# Supplementary material for: Leveraging platinum-protein interactions to overcome chemoresistance
Source: Nat Commun. 2025 Oct 20;16:9263. doi: 10.1038/s41467-025-64295-0 (PMC12537935; doi:10.1038/s41467-025-64295-0)
Supplement: Supplementary file 1 — Supplementary Information [file 41467_2025_64295_MOESM1_ESM.pdf]

Supplementary Information

Leveraging platinum-protein interactions to overcome chemoresistance

Fang Wang,[1,2,3,#,*] Jonathan Braverman,[2,4,#,*] George Eng,[2,5] Ozen Leylek,[2] Nicholas L. Petrone,[3] Daniel S. Honeycut,[3] Shinya Imada,[2] Brian Pallares,[6] Daiyao Zhang,[2] Jason M. Mrosla,[6] Camellia S. Huang,[2] Anna A. Griadunova,[4] William K. McCarthy,[6] Jacob M. Goldberg,[6] Michael T. Hemann,[2] Stephen J. Lippard,[1,*] Ömer H. Yilmaz[2,5,*]

[1] Department of Chemistry, Massachusetts Institute of Technology, Cambridge, MA 02139, United States
[2] Department of Biology, The David H. Koch Institute for Integrative Cancer Research at MIT, Massachusetts Institute of Technology, Cambridge, MA 02139, USA
[3] Department of Chemistry, University of Rhode Island, Kingston, RI 02881, USA
[4] Innovative Genomics Institute, University of California, Berkeley, Berkeley, CA 94720, USA
[5] Department of Pathology, Massachusetts General Hospital and Harvard Medical School, Boston, MA 02114, USA
[6] Department of Chemistry, Colgate University, Hamilton, NY 13346, USA

[#] These authors contributed equally to this work.
*e-mail: fangwang@uri.edu, braverman@berkeley.edu, lippard@mit.edu, ohyilmaz@mit.edu

Contents

S1. General. ***2***
S2. Synthetic procedures. ***5***
S3. Stability of DoxPt. ***16***
S4. Reactivity of Oxaliplatin and DoxPt with different sulfur-containing small molecules. ***21***
S5. Reactivity of DoxPt4 and DoxPt5 with different sulfur-containing small molecules. ***31***
S6. Reactivity of DoxPt with proteins. ***35***
S5. Intracellular Pt content analysis. ***37***
S6. In vitro toxicity determination with conventional cancer cell lines. ***45***
S7. Colorectal cancer organoid generation and related cytotoxicity studies. ***67***
S8. Resistant colorectal cancer organoid generation and related cytotoxicity studies. ***69***
S9. Imaging studies. ***72***
S10. Characterization of DoxPt with RNAi-based competition assay. ***84***
S11. Mechanisms of action of DoxPt study with the NCI60 screening panel. ***89***
S12. Investigation of the inhibitory activity of DoxPt to human Top2α using decatenation assay ***93***
S13. Investigation of the mode of action of DoxPt with human Top2α cleavage assay ***95***
S14. Investigation of the mode of action of DoxPt with wheat germ Top1 intercalation (unwinding) assay .. ***97***
S15. HPLC Chromatograms of purified compounds. ***99***
S16. NMR Spectra. ***102***
S17. References. ***173***

S1. General.

Materials and methods for chemistry.

Reagents were purchased from commercial sources and used as received. Doxorubicin hydrochloride (98%) was purchased from A Chemtek Inc (catalog # 0104-018277), and the purity was examined by ^{1}H NMR spectroscopy and analytical HPLC. Anhydrous solvents were saturated with argon and purified by passage through two columns of activated alumina. Air-sensitive reactions and compounds were handled with standard Schlenk techniques or in an MBraun dry box. Column chromatography was performed on silica gel (230–400 mesh, 60 Å). Milli-Q purified water with resistivity of at least 18 MΩ·cm^{-1} was used to prepare aqueous solutions for analytical chemistry. For Pt content analysis, ultrapure grade HNO_3 (VWR catalog # 87003-226) was used.

Platinum compounds doxaliplatin (DoxPt, **2**) and doxaliplatin3 (DoxPt3, **4**) were purified using a reverse phase Biotage® SNAP Ultra C18 column (12 g) with H_2O/MeOH as the mobile phase prior to HPLC purification. Compound **1** ($DoxNH_2NH_2$), doxaliplatin, and doxaliplatin3 were purified using Agilent 1260 Series HPLC systems fitted with multi-wavelength detectors using a C18 reverse stationary phase (Zorbax-SB C18 column: 7 µm, 21.2 × 250 mm). The purity of compound **1**, DoxPt, doxaliplatin2 (DoxPt2, **3**), and DoxPt3 was examined using an Agilent 1200 Series HPLC system fitted with multi-wavelength detectors using a C18 reverse stationary phase (Zorbax-SB C18 column: 5 µm, 4.6 × 250 mm). The mobile phase was composed of two solvents. For compound **1**, the mobile phase was composed of A: H_2O + 0.1% (v/v) CF_3CO_2H; B: CH_3CN + 0.1% (v/v) CF_3CO_2H. For platinum compounds, the mobile phase was composed of A: H_2O; B: CH_3OH.

NMR spectra were acquired on a 400 MHz Bruker AVANCE-400 spectrometer or a Varian Inova-500 NMR spectrometer. ^{1}H NMR and $^{13}C\{^{1}H\}$ NMR chemical shifts are reported in ppm relative to that of $SiMe_4$ (δ = 0.00) and were referenced internally to residual solvent peaks.[1] ^{195}Pt NMR chemical shifts are reported in ppm relative to that of K_2PtCl_6 (δ = 0.00). Low-resolution electrospray mass spectra were acquired on an Agilent 1100 Series LC/MSD Trap spectrometer or on a Shimadzu LCMS-2020 spectrometer. High-resolution mass spectra were acquired on an Agilent 6510 Series Quadrupole Time-Of-Flights spectrometer at the MIT Center for Environmental Health Sciences or on an AB SCIEX TripleTOF 4600 mass spectrometer equipped with a DuoSpray™ ion source at the RI-INBRE Centralized Research Core Facility at the University of Rhode Island. Tandem MS spectra were acquired on the AB SCIEX TripleTOF 4600 mass spectrometer equipped with a DuoSpray™ ion source.

Materials and methods for biology.

Pharmaceutical-grade cisplatin (catalog # PHR1624), oxaliplatin (catalog # PHR1528), carboplatin (catalog # PHR3417), and doxorubicin hydrochloride (catalog # D1515) were purchased from Sigma-Aldrich. Doxorubicin stock solutions were prepared by dissolving doxorubicin hydrochloride in H_2O and stored at –20 °C. The stock solution of doxorubicin was used and discarded after each experiment. Oxaliplatin and carboplatin stock solutions were prepared by dissolving the corresponding platinum agent in Milli-Q water (18 $M\Omega \cdot cm^{-1}$) at rt and stored at –20 °C. Cisplatin stock solutions were prepared by dissolving cisplatin in 0.9 wt % sodium chloride solution at rt and stored at –20 °C. DoxPt and DoxPt3 stock solutions were prepared by dissolving the corresponding compound in a glycerol/water mixture (1:1 v/v). The suspension was heated at 60 °C for about 5 min to facilitate solubilization. The stock solutions were stored at –20 °C and heated at 60 °C for 1–2 min to dissolve the precipitated compound before use. DoxPt2 solutions were prepared in glycerol. The suspension was heated at 60 °C for about 5 min to facilitate solubilization. This solution was then diluted with an equal volume of 0.9 wt % sodium chloride solution. This solution was stored at –20 °C and heated at 60 °C for 1–2 min to dissolve the precipitated compound before use. All platinum agent stock solutions were filtered through a sterile 0.2 μm regenerated cellulose syringe filter after preparation. The concentration of the filtrate was determined by atomic absorption spectrometry. Verapamil (Sigma-Aldrich catalog # V4629) was prepared as a 10 mM stock solution in 100% EtOH. The individual dose-response curve of each biological replicate was fitted with Dr. Fit.[2] The average IC_{50} values were calculated from at least three biological replicates.

Cells and organoids were maintained at 37 °C and under a humidified 5% CO_2. Cell culture media were prepared with Advanced DMEM/F-12 (ThermoFisher catalog # 12634010), supplemented with fetal bovine serum (FBS, 5 vol %, HyClone™, Cytiva catalog # SH30910.03), 2 mM GlutaMAX™ (1 vol %, ThermoFisher catalog # 35050061), and 100 U/mL penicillin-streptomycin (1 vol %, 10,000 U/mL, ThermoFisher catalog # 15140122). Matrigel® (Corning catalog # 356231) was used as a 67 vol % mixture with cell culture media. Resazurin was purchased from ThermoFisher (catalog # R12204).

Unless stated otherwise, cell lines were obtained from the Robert A. Swanson (1969) Biotechnology Center at the Massachusetts Institute of Technology. Cell lines, including A2780ADR (catalog # 93112520) MES-SA (catalog # 95051030), and MES-SA/Dx5 (catalog # 95051031), were purchased from Sigma-Aldrich. Drug-sensitive and -resistant cell line pairs, including HL-60 (Catalog # CRL-240) and HL-60/MX2 (Catalog # CRL-2257), H69 (Catalog # HTB-119) and H69AR (Catalog # CRL-11351), and CEM (Catalog # CCL-119) and CEM/C2 (Catalog # CRL-2264), were purchased from ATCC. The oxaliplatin-resistant OVCAR8 cell line (OVCAR8/OxR) was generated by treating the OVCAR8 human ovarian cancer cell line repeatedly with increasing concentrations of oxaliplatin until at least a 10-fold increase in IC_{50} was observed. The doxorubicin-resistant OVCAR8 cell line (OVCAR8/DoxR) was generated by treating the OVCAR8 human ovarian cancer cell line repeatedly with increasing concentrations of doxorubicin until at least a 50-fold increase in IC_{50} was observed.

For *in vivo* mouse experiments, metastatic AKPS-TdT colon cancer organoid lines were used. Trypsinized organoids (2.0×10^5 cells) were seeded in the peritoneum of C57BL/6 mice. For *in vivo* dosing, DoxPt was dissolved in glycerol and water (50:50 vol %). DoxPt treatment was initiated three days after tumor engraftment and dosed weekly thereafter via intraperitoneal injection. For the survival study, doxaliplatin was initially dosed at 1 mg/kg on Day 3 after tumor engraftment, and the mice were subsequently dosed at 0.5 mg/kg weekly. For the tumor burden reduction study, doxaliplatin was initially dosed at 2 mg/kg on Day 3 after tumor engraftment, and the mice were subsequently dosed at 1 mg/kg on Day 10 and Day 17. The mice were euthanized on Day 23, and tumors were dissected and weighed. All experiments involving mice were carried out using young adult female mice (3 to 5 months old). Mice were never allowed to bear a tumour burden exceeding the maximal tumour size of 1 cm in diameter, as approved by MIT's Committee on Animal Care.

S2. Synthetic procedures.

***N*-Trifluoroacetyl doxorubicin (6)**

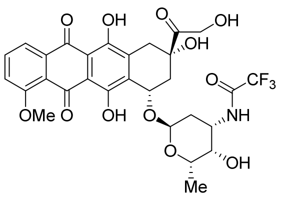

The titled compound was synthesized based on a reported procedure.[3] In a Schlenk flask containing doxorubicin hydrochloride salt (1.16 g, 2.0 mmol), anhydrous pyridine (60 mL) was added at −30 °C. The reaction was stirred at this temperature for 15 min. A mixture of trifluoroacetic anhydride (3.78 g, 2.54 mL, 18.0 mmol) in Et_2O (12 mL) was added to the flask dropwise at the same temperature. The reaction was gradually warmed to 0 °C over a period of 3 h, during which doxorubicin dissolved to give a slightly brown solution. The reaction was diluted with water (100 mL) and stirred for 20 min to hydrolyze excess trifluoroacetic anhydride. The reaction mixture was extracted with ethyl acetate (100 mL × 3). The combined organic phase was washed with water (100 mL × 3) and dried over $MgSO_4$. The solvent was removed under vacuum. The crude product was suspended in a mixture of butanone and hexanes (80 mL + 320 mL, respectively) and stored at −40 °C for 3 h. The dark red solid was collected by suction filtration, washed with hexanes, and dried under vacuum (1.20 g, 94% yield). This product was used directed in the next step without further purification. Analytical pure sample was obtained by column chromatography (silica gel, dichloromethane:methanol = 98:2 to 96:4). δ ^{1}H NMR (400 MHz, $CDCl_3$-CD_3OD 6:1 *v*:*v*) δ 13.88 (s, 1H, exchangeable proton, 0.05 H was observed), 13.18 (s, 1H, exchangeable proton, 0.05 H was observed), 7.94 (dd, *J* = 7.7, 0.7 Hz, 1H), 7.90 (br, 1H, exchangeable proton, 0.10 H was observed), 7.88 (br, 1H, exchangeable proton, 0.12 H was observed), 7.74 (t, *J* = 8.1 Hz, 1H), 7.36 (d, *J* = 8.4 Hz, 1H), 5.44 (d, *J* = 3.5 Hz, 1H), 5.19 (dd, *J* = 2.0, 1.9 Hz, 1H), 4.71 (s, 2H), 4.14 – 4.09 (m, 2H), 4.02 (s, 3H), 3.58 (d, *J* = 1.6 Hz, 1H), 3.16 (dd, *J* = 18.8, 1.5 Hz, 1H), 2.92 (d, *J* = 18.8 Hz, 1H), 2.31 (pseudo d, *J* = 14.7 Hz, 1H), 2.12 (dd, *J* = 14.7, 4.3 Hz, 1H), 1.93 (td, *J* = 13.2, 4.0 Hz, 1H), 1.76 (dd, *J* = 13.2, 4.8 Hz, 1H), 1.24 (d, *J* = 6.6 Hz, 3H). The data are consistent with reported values.[4] ^{19}F{^{1}H} NMR (396 MHz, $CDCl_3$-CD_3OD 6:1 *v*:*v*) −76.19 (s, 1.45F), −76.22 (s, 1.55F). ^{19}F{^{1}H} NMR spectrum acquired 30 min after sample preparation indicated that, presumably due to the slow amide bond rotation, two conformers were observed in a ratio of 1.45:1.55. The ratio of two conformers decreased to 0.17:2.83 after 18 h at rt, likely indicating a slow conversion of the conformer at −76.19 ppm to a more stable conformer at −76.22 ppm. ESI-MS(−) m/z calcd for $[M-H]^-$ 638.1, found 638.0.

***N*-Trifluoroacetyl doxorubicin 9,14-cyclic methyl orthoester (7)**

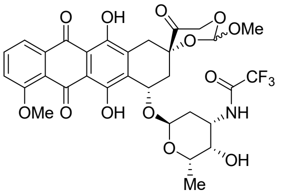

To a solution of **6** (1.06 g, 1.66 mmol) and methyl orthoformate (80 mL) in THF (280 mL) was added (1*S*)-(+)-10-camphorsulfonic acid (102 mg, 0.44 mmol) in one portion. The reaction was stirred at room temperature for 3 h. The reaction was then quenched with 5 wt % $NaHCO_3$ aq. solution (400 mL). The mixture was extracted with ethyl acetate (100 mL × 3). The combined organic phase was washed with 5 wt % $NaHCO_3$ aq. solution (200 mL) and water (200 mL). The organic phase was dried over $MgSO_4$. The solvent was removed under vacuum. The crude product was purified by column chromatography (silica gel, ethyl acetate:hexanes = 3:1 to 6:1) to afford a dark orange solid (362 mg, 32% yield). ESI-MS(−) m/z calcd for $[M-H]^-$ 680.2, found 680.1. ^{19}F NMR spectrum of **7** in acetone-

d6 displayed four major signals with a ratio of 70:18:7:5. These signals presumably corresponded to two diastereomers of the cyclic methyl orthoester, each of which has two conformers due to the slow amide bond rotation. Because of the complexity of the ^{1}H NMR spectrum, little structural information was obtained.

N-Trifluoroacetyl (4'S)-triflate doxorubicin 9,14-cyclic methyl orthoester (8)

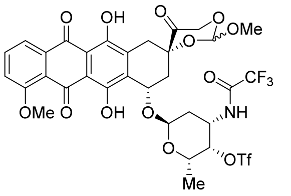

To a Schlenk flask containing **7** (301 mg, 0.44 mmol) in CH_2Cl_2 (10 mL), pyridine (280 mg, 285 μL, 3.54 mmol) was added dropwise at −40 °C. Triflic anhydride (499 mg, 297 μL, 1.77 mmol) in CH_2Cl_2 (5 mL) was then added dropwise to the flask at the same temperature. The reaction was stirred for 2 h, during which time the reaction was gradually warmed to 0 °C. The completion of the reaction was confirmed by thin layer chromatography (ethyl acetate:hexanes = 3:1). The reaction was diluted with ice water (40 mL). The mixture was extracted with cold Et_2O (40 mL×3). The combined organic phase was washed with cold NaOAc aq. solution (10 wt %, 80 mL) and then cold water (80 mL). The organic phase was dried over $MgSO_4$. The solvent was removed under vacuum. The crude product (346 mg) was used in the next step without further purification.

N-Trifluoroacetyl (4'R)-azido doxorubicin 9,14-cyclic methyl orthoester (9)

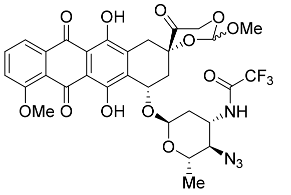

In a glovebox, **8** (crude, 346 mg, approximately 0.43 mmol) was dissolved in anhydrous THF (15 mL). To this solution, $n\mathrm{Bu_4NN_3}$ (121 mg, 0.43 mmol) was added. A dark red solution formed immediately. The reaction was stirred at room temperature for 12 h. The solvent was removed under vacuum. The crude product was purified by column chromatography (ethyl acetate:hexanes = 2:1). An orange solid was obtained (199 mg, 64% yield based on **8**). ESI-MS(−) m/z calcd for $[M-H]^-$ 705.2, found 705.1. ^{1}H NMR (400 MHz, $CDCl_3$) δ 14.07 (s, 1H), 13.26 (s, 1H), 8.01 (dd, *J* = 7.7, 0.9 Hz, 1H), 7.77 (t, *J* = 8.1 Hz, 1H), 7.38 (dd, *J* = 8.5, 0.7 Hz, 1H), 6.23 (d, *J* = 8.8 Hz, 1H), 5.73 (s, 1H), 5.56 (d, *J* = 3.1 Hz, 1H), 5.06 (dd, *J* = 3.8, 1.3 Hz, 1H), 4.55 (d, *J* = 17.4 Hz, 1H), 4.30 (d, *J* = 17.3 Hz, 1H), 4.26 (m, 1H), 4.11 (dq, *J* = 9.7, 6.0 Hz, 1H), 4.07 (s, 3H), 3.46 (dd, *J* = 11.9, 1.9 Hz, 1H), 3.43 (s, 3H), 3.04 (t, *J* = 18.8 Hz, 1H), 3.03 d, *J* = 18.8 Hz, 1H), 2.61 (dt, *J* = 13.1, 1.7 Hz, 1H), 2.19 (ddd, *J* = 12.6, 4.2, 0.5 Hz, 1H), 2.06 (dd, *J* = 14.9, 5.4 Hz, 1H), 1.90 (td, *J* = 12.9, 3.8 Hz, 1H), 1.41 (d, *J* = 6.2 Hz, 3H). $^{13}C\{^{1}H\}$ NMR (101 MHz, $CDCl_3$) δ 207.55, 187.32, 186.71, 161.13, 157.11 (q, *J* = 37.6 Hz) 156.92, 155.73, 135.86, 135.65, 134.24, 134.22, 120.99, 119.94, 118.49, 115.72 (q, *J* = 288.4 Hz) 111.52, 111.25, 109.59, 99.84, 78.39, 69.52, 67.82, 66.90, 65.17, 56.80, 53.94, 48.15, 35.86, 35.60, 30.88, 18.79. ^{19}F NMR (376 MHz, $CDCl_3$) δ −75.93.

(4'R)-Azido doxorubicin 9,14-cyclic methyl orthoester (10)

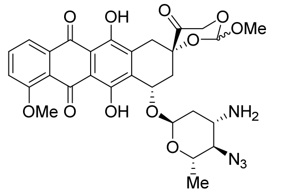

To a stirred solution of **9** (199 mg, 0.28 mmol) in $CHCl_3$ (140 mL) was added NaOH in sat. brine (0.5 M, 14.0 mL). The reaction was stirred vigorously (1200 rpm) at room temperature for 24 h. The completion of the reaction was confirmed by ESI-MS. The organic phase was isolated. The aqueous phase was extracted with CH_2Cl_2 (50 mL × 2). The combined organic phase was washed with brine (50 mL × 2) and water (50 mL × 2). The organic phase was dried over $MgSO_4$. The solvent was removed under vacuum. The crude product was used in the next step without

further purification (80 mg, 47% yield). ESI-MS(−) m/z calcd for $[M-H]^-$ 609.2, found 609.2. ESI-MS(+) m/z calcd for $[M+H]^+$ 611.2, found 611.2.

(4’*R*)-Dehydroxyamino doxorubicin (DoxNH$_2$NH$_2$, 1)

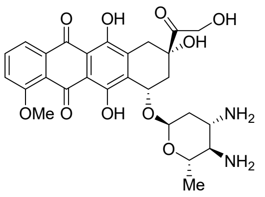

To the crude product **10** (80 mg, 0.13 mmol) in THF (52 mL) was added triphenylphosphine (102 mg, 0.39 mmol) in small portions. The reaction was stirred at room temperature for 48 h. The completion of the reaction was confirmed by ESI-MS. ESI-MS(+) m/z calcd for the iminophosphorane $[M+H]^+$ 845.3, found 845.5. This reaction mixture was combined with water (5.2 mL) and stirred at room temperature. The progress of the hydrolysis of the iminophosphorane was monitored by ESI-MS, which was complete after 24 h. ESI-MS(+) m/z calcd for (4’*R*)-dehydroxyamino doxorubicin 9,14-cyclic methyl orthoester (**11**) $[M+H]^+$ 585.2, found 585.1; and $[M+Ph_3PO+H]^+$ 863.3, found 863.3. To the same reaction mixture, an aliquot of aq. HCl (0.25 M, 1.04 mL, 0.26 mmol) was added. The progress of the reaction was monitored by ESI-MS. The reaction was complete after 24 h. The solvents were removed under vacuum. The reaction mixture was dissolved in MeCN/water (1:1, v/v, 20 mL). The mixture was suction filtered. The filtrate was then passed through a 0.2 μm PTFE syringe filter. The crude product was purified by preparative HPLC. Preparative HPLC used a (A) water (0.1% v/v CF_3CO_2H) / (B) CH_3CN (0.1% v/v CF_3CO_2H) solvent system, according to the following protocol: constant flow rate 15.0 mL·min^{-1}; 0.0-3.0 min, linear gradient 20-27% B; 3.0-13.0 min, linear gradient 27-37% B; 13.0-15.0 min, linear gradient 37-100% B; 15.0-18.0 min, 100% B; 18.0-20.0 min, linear gradient 100-20% B; 20.0-21.0 min, 20% B. Fractions containing the desired product (T_R = 11.8 min) were combined and lyophilized to give an orange solid as a bis(trifluoroacetic acid) salt (48 mg, 48% yield based on **11**). The purity of **1** was examined by analytical HPLC (T_R = 17.2 min). Analytical HPLC used a (A) water (0.1% v/v CF_3CO_2H) / (B) CH_3CN (0.1% v/v CF_3CO_2H) solvent system, according to the following protocol: constant flow rate 1.0 mL·min^{-1}; 0.0-5.0 min, 10% B; 5.0-30.0 min, linear gradient 10-100% B; 30.0-33.0 min, 100% B; 33.0-36.0 min, linear gradient 100-10% B; 36.0-40.0 min, 10% B. The diacetate salt of **1** for *in vitro* experiments was obtained by passing the bis(trifluoroacetic acid) salt through a preparative HPLC with a (A) water (0.1% v/v CH_3CO_2H) / (B) CH_3CN (0.1% v/v CH_3CO_2H) solvent system. UV-Vis (50 mM PIPES, 100 mM KCl, pH 7.0) ε_{490} =18,300 M^{-1}·cm^{-1}. The ^{1}H NMR spectrum of **1** in DMSO-d_6 displayed multiple sets of signals, a feature indicating the presence of multiple species. In contrast, the ^{1}H NMR spectrum of **1** in CD_3CN/D_2O (3:1, v/v) only showed one set of signals. This observation suggested that compound **1** underwent slow conformational interconversions on the NMR timescale in DMSO-d_6. ^{1}H NMR (400 MHz, CD_3CN/D_2O 3:1, v/v) δ 7.76 – 7.64 (m, 2H), 7.41 (d, *J* = 9.0 Hz, 1H), 5.45 (d, *J* = 2.8 Hz, 1H), 4.94 (d, *J* = 2.7 Hz, 1H), 4.69 (d, *J* = 20 Hz, 2H, AB system), 4.16 (dq, *J* = 10.0, 6.3 Hz, 1H), 3.91 (s, 3H), 3.67 (ddd, *J* = 12.0, 10.5, 4.6 Hz, 1H), 3.15 (t, *J* = 10.0 Hz, 1H), 3.00 (d, *J* = 19.2 Hz, 1H, part of an AB system), 2.74 (d, *J* = 18.7 Hz, 1H, part of an AB system), 2.32 – 2.04 (m, 3H), 1.94 (1H, overlap with CD_2HCN), 1.36 (d, *J* = 6.3 Hz, 3H). ^{13}C{^{1}H} NMR (101 MHz, CD_3CN/D_2O 3:1, v/v) δ 214.8, 187.7, 187.6, 161.9, 156.7, 155.4, 137.3, 135.6, 134.7(2), 134.7(0), 120.8, 120.4, 120.3, 112.2, 112.0, 99.3, 76.7, 70.4, 66.3, 65.5, 57.4, 55.6, 47.6, 36.8, 34.1, 33.2, 17.9.

ESI-MS(+) m/z calcd for $DoxNH_2NH_2$ (**1**) $[M+H]^+$ 543.2, found 543.4. ESI-MS(−) m/z calcd $[M-H]^-$ 541.2, found 541.2. HRMS (ESI/Q-TOF) m/z: $[M+H]^+$ Calcd for $C_{27}H_{31}N_2O_{10}^+$ 543.1974; Found 543.1947.

Doxaliplatin (DoxPt, 2)

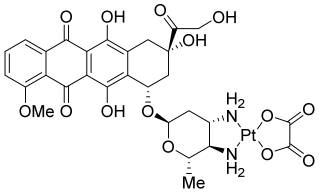

A solution of **1** bis(trifluoroacetic acid) salt (77.0 mg, 0.100 mmol) in water (9 mL) was added in one portion to a stirred solution of K_2PtCl_4 (41.5 mg, 0.100 mmol) in water (30 mL). A red precipitate formed. The reaction was stirred at room temperature for 24 h. To the same solution, $AgNO_3$ aq. solution (0.10 M, 4.0 mL) was added. The reaction was stirred at room temperature for 24 h. An aq. solution of $K_2C_2O_4$ (0.20 M, 0.50 mL) was then added. The reaction was stirred at room temperature for 24 h. The reaction mixture was lyophilized together with a small amount of C18-reversed phase silica gel. The crude product was purified using a reverse phase Biotage® SNAP Ultra C18 column (12 g). The purification used a (A) water / (B) MeOH solvent system, according to the following protocol: constant flow rate 12.0 $mL \cdot min^{-1}$; 0.0-3.0 column volume (CV), 0% B; 3.0-10.0 CV, linear gradient 0-80% B; 10.0-20.0 CV, 80% B. Fractions from 13-15 CV were collected and filtered through a 0.2 μm PTFE syringe filter. The combined fractions were further purified by preparative HPLC. Preparative HPLC used a (A) water / (B) MeOH solvent system, according to the following protocol: constant flow rate 15.0 $mL \cdot min^{-1}$; 0.0-3.0 min, linear gradient 25-62% B; 3.0-13.0 min, linear gradient 62-72% B; 13.0-15.0 min, linear gradient 72-100% B; 15.0-17.0 min, 100-25% B; 17.0-18.0 min, 25% B. Fractions containing the desired product (T_R = 12.8 min) were combined and lyophilized to give a bright orange solid (24.0 mg, 29% yield based on **1**). The purity of **2** was examined by analytical HPLC (T_R = 26.5 min). Analytical HPLC used a (A) water / (B) MeOH solvent system, according to the following protocol: constant flow rate 1.0 $mL \cdot min^{-1}$; 0.0-5.0 min, 10% B; 5.0-30.0 min, linear gradient 10-100% B; 30.0-33.0 min, 100% B; 33.0-36.0 min, linear gradient 100-10% B; 36.0-40.0 min, 10% B. UV-Vis (water) ε_{478} =16,500 $M^{-1} \cdot cm^{-1}$. The ^1H NMR spectrum of **2** in DMF-d_7 showed a set of signals with significant line broadening, which indicated that the observed species underwent a slow exchange reaction. The low solubility of **2** in DMF, however, impeded the characterization of the compound. The stability and the relatively high solubility[5] of **2** in DMSO-d_6 allowed us to further characterize the compound. In contrast to the ^1H NMR spectrum in DMF-d_7, the ^1H NMR spectrum of **2** in DMSO-d_6 revealed two sets of signals, presumably due to the presence of two conformers (Figure S147). The multiplicity of some ^1H NMR signals was not determined due to overlap. Conformer A (the population is approximately 45%): ^1H NMR (500 MHz, DMSO-d_6, water suppression using presaturation) δ 7.878 (1H), 7.637 (1H), 7.187 (d, J = 7.5 Hz, 1H), 6.215 (br, 1H), 5.759 (br, 1H), 5.713 (br, 1H), 5.334 (m, 1H), 5.241 (m, 1H), 4.918 (br, 1H), 4.759 (1H), 4.156 (d, J = 14.6 Hz, 1H, part of an AB system), 4.068 (d, J = 14.6 Hz, 1H, part of an AB system), 3.795 (1H), 3.770 (3H), 3.192 (br, 1H), 2.705 (d, J = 18.9 Hz, 1H), 2.121 (1H), 2.040 (1H), 1.869 (1H), 1.663 (1H), 1.592 (1H), 1.120 (d, J = 5.6 Hz, 3H). Conformer B (the population is approximately 55%): ^1H NMR (500 MHz, DMSO-d_6, water suppression using presaturation) δ 7.878 (1H), 7.637-7.609 (2H), 6.339 (br, 1H), 6.139 (d, J = 4.9 Hz, 1H), 5.676 (1H), 5.411 (pseudo t, J = 10.3 Hz, 1H), 5.103 (br, 1H), 4.893 (1H), 4.628 (d, J = 19.7 Hz, 1H, part of an AB

system), 4.544 (d, J = 20.0 Hz, 1H, part of an AB system), 3.966 (3H), 3.947 (1H), 2.908 (pseudo t, J = 19.2 Hz, 2H), 2.461 (1H), 2.143 (1H), 2.023 (1H), 1.942 (d, J = 9.5 Hz, 1H), 1.869 (1H), 1.619 (1H), 1.183 (d, J = 6.0 Hz, 3H). Due to the relatively low concentration of **2** in DMSO-d_6, only part of the $^{13}C\{^1H\}$ NMR signals were detected through ^{13}C-^1H correlation experiments. The $^{13}C\{^1H\}$ NMR signals of Conformer A detected by ^1H-^{13}C HSQC and ^1H-^{13}C HMBC spectroscopy: $^{13}C\{^1H\}$ NMR (101 MHz, DMSO-d_6) δ 16.98, 31.68, 34.80, 37.89, 55.85, 56.35, 64.07, 65.27, 66.70, 68.15, 98.84, 118.56, 118.86, 134.84, 159.78. The $^{13}C\{^1H\}$ NMR signals of Conformer B detected by ^1H-^{13}C HSQC and ^1H-^{13}C HMBC spectroscopy: $^{13}C\{^1H\}$ NMR (101 MHz, DMSO-d_6) δ 17.24, 32.01, 36.73, 37.27, 56.13, 56.31, 63.56, 64.07, 68.53, 69.85, 99.05, 118.68, 118.84, 135.99, 160.62. ^{195}Pt NMR (86 MHz, DMSO-d_6) δ −1989 (br). ESI-MS(+) m/z calcd $[M+H]^+$ 826.1, found 826.2. HRMS (ESI/Q-TOF) m/z: $[M+Na]^+$ Calcd for $C_{29}H_{30}N_2NaO_{14}Pt^+$ 848.1237; Found 848.1187. In the MS/MS experiment, the fragments of molecular ion ($M+Na^+$) of m/z = 848.1 confirm the structure of DoxPt. Anal. Calcd for $C_{29}H_{30}N_2O_{14}Pt{\cdot}(H_2O)_8$ C, 35.92; H, 4.78; N, 2.89. Found: C, 35.63; H, 4.22; N, 2.77%.

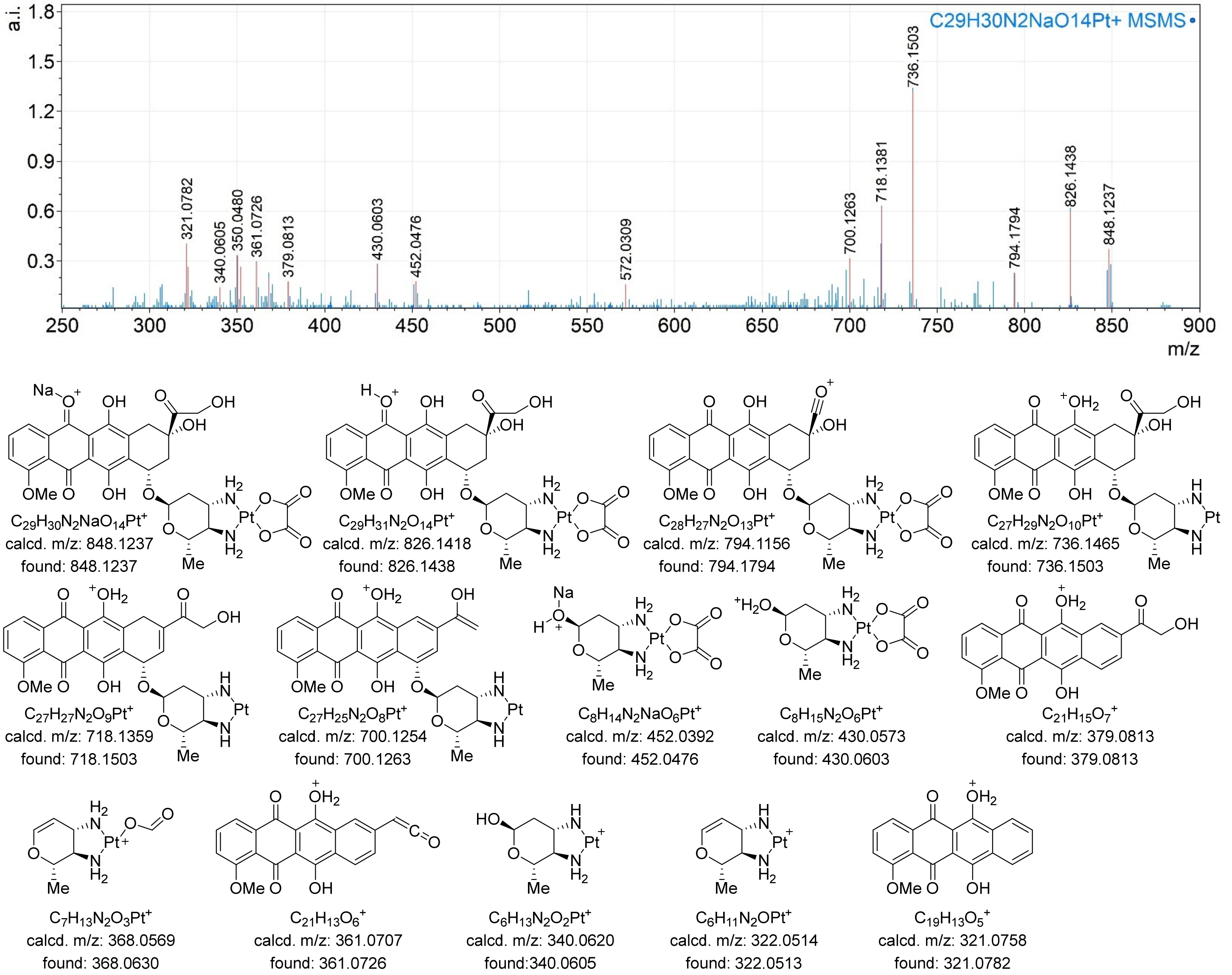

Figure S1. Tandem mass spectrum of DoxPt and the fragmentation analysis.

Doxaliplatin2 (DoxPt2, 3)

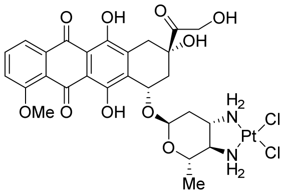

A solution of **1** bis(trifluoroacetic acid) salt (7.7 mg, 0.0100 mmol) in water (1.0 mL) was added in one portion to a stirred solution of K_2PtCl_4 (4.2 mg, 0.0100 mmol) in water (3.0 mL). A red precipitate formed. The reaction was stirred at room temperature for 24 h. The reaction mixture was centrifuged at 4000 rpm for 3 min. The aqueous supernatant was discarded. The solid was suspended in MeOH (1 mL), to which Et_2O (8 mL) was then added. The mixture was centrifuged at 4000 rpm for 3 min. The supernatant was discarded. This process was repeated two more times. The remaining solid was dried under vacuum to give a bright orange solid (7.0 mg, 58% yield based on **1**). The purity of **3** was examined by analytical HPLC (T_R = 26.6 min). Analytical HPLC used a (A) water / (B) MeOH solvent system, according to the following protocol: constant flow rate 1.0 mL·min^{-1}; 0.0-5.0 min, 10% B; 5.0-30.0 min, linear gradient 10-100% B; 30.0-33.0 min, 100% B; 33.0-36.0 min, linear gradient 100-10% B; 36.0-40.0 min, 10% B. The very low solubility of Compound **3** in commonly used deuterated solvents did not permit characterization by NMR spectroscopy. A freshly prepared solution of **3** in MeOH was characterized by mass spectrometry. ESI-MS(+) m/z calcd $[M+Na]^+$ 831.1, found 831.1. HRMS (ESI/Q-TOF) m/z: $[M+Na]^+$ Calcd for $C_{27}H_{30}Cl_2N_2NaO_{10}Pt^+$ 831.0820; Found 831.0749; $[M-Cl+OMe+H]^+$ Calcd for $C_{28}H_{34}ClN_2O_{11}Pt^+$ 805.1495; Found 805.1453.

Doxaliplatin3 (DoxPt3, 4)

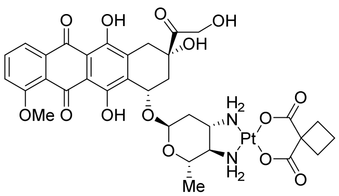

A solution of **1** bis(trifluoroacetic acid) salt (6.23 mg, 0.015 mmol) in water (1.5 mL) was added in one portion to a stirred solution of K_2PtCl_4 (11.6 mg, 0.015 mmol) in water (4.5 mL). A red precipitate formed. To the same solution, $AgNO_3$ aq. solution (0.10 M, 0.60 mL) was added. The reaction was stirred at room temperature for 24 h. An aq. solution of cyclobutane-1,1-dicarboxylic acid dipotassium salt (0.10 M, 0.50 mL, 150 μL, prepared by mixing KOH aq. solution with cyclobutane-1,1-dicarboxylic acid aq. solution) was then added to this mixture. The reaction was stirred at room temperature for 24 h. The reaction mixture was loaded on a reverse phase Biotage® SNAP Ultra C18 column (12 g). The purification used a (A) water / (B) MeOH solvent system, according to the following protocol: constant flow rate 12.0 mL·min^{-1}; 0.0-5.0 column volume (CV), 0% B; 5.0-20.0 CV, linear gradient 0-100% B; 20.0-25.0 CV, 100% B. Fractions from CV17 to CV18 were collected and filtered through a 0.2 μm PTFE syringe filter. The combined fractions were further purified by preparative HPLC. Preparative HPLC used a (A) water / (B) MeOH solvent system, according to the following protocol: constant flow rate 15.0 mL·min^{-1}; 0.0-3.0 min, linear gradient 40-50% B; 3.0-24.0 min, linear gradient 50-71% B; 24.0-25.0 min, linear gradient 71-100% B; 25.0-27.0 min, 100-40% B. Fractions containing the desired product (T_R = 22.4 min) were combined and lyophilized to give a bright orange solid (3.8 mg, 29% yield based on **1**). The purity of **4** was examined by analytical HPLC (T_R = 27.0 min). Analytical HPLC used a (A) water / (B) MeOH solvent system, according to the following protocol: constant flow rate 1.0 mL·min^{-1}; 0.0-5.0 min, 10% B; 5.0-30.0 min, linear gradient 10-100% B; 30.0-33.0 min, 100% B; 33.0-36.0 min, linear gradient 100-10% B; 36.0-40.0 min,

10% B. The very low solubility of Compound **4** in commonly used deuterated solvents did not permit characterization by NMR spectroscopy. A freshly prepared solution of **4** in MeOH was characterized by mass spectrometry. ESI-MS(+) m/z calcd $[M+Na]^+$ 902.3, found 902.3. HRMS (ESI/Q-TOF) m/z: $[M+H]^+$ Calcd for $C_{33}H_{37}N_2O_{14}Pt^+$ 880.1888; Found 880.1824. In the MS/MS experiment, the fragments of molecular ion ($M+H^+$) of m/z = 880.2 confirm the structure of DoxPt3.

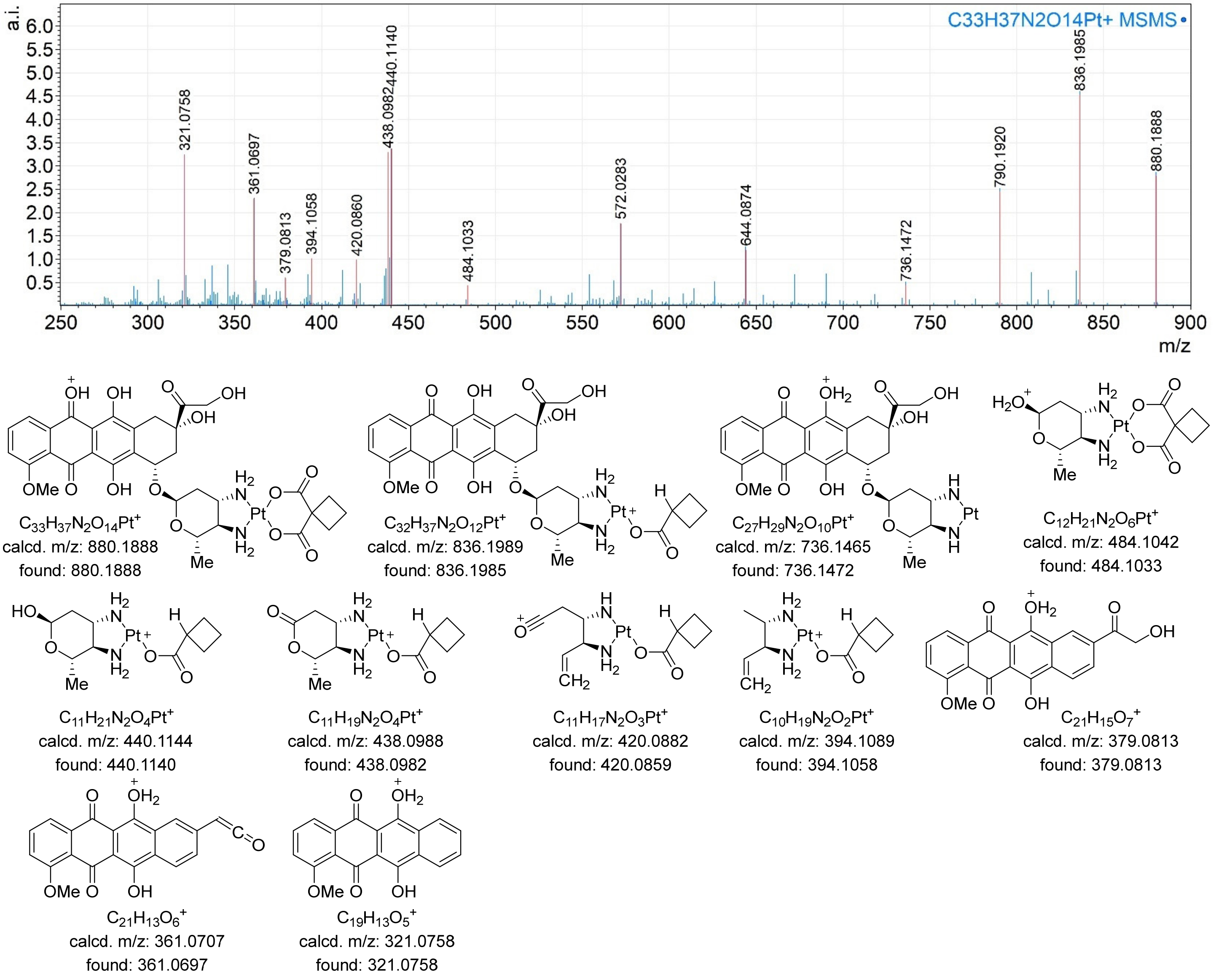

Figure S2. Tandem mass spectrum of DoxPt3 and the fragmentation analysis.

Doxaliplatin4 (DoxPt4, 5) and Doxaliplatin5 (DoxPt5, 12)

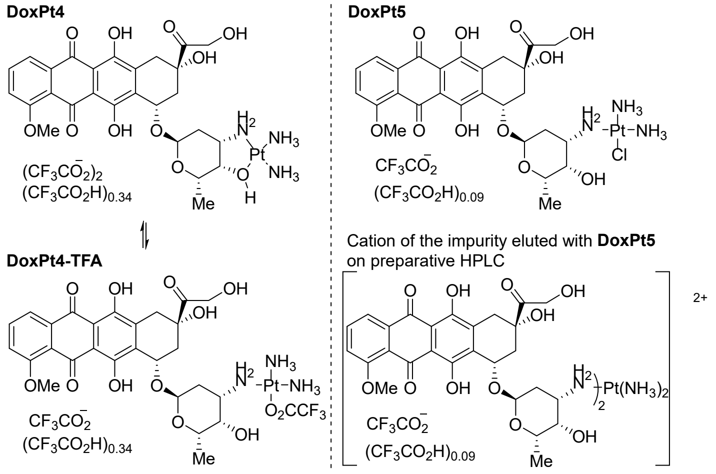

Cisplatin (45.0 mg, 0.10 mmol) was dissolved in DMF (1.0 mL) at 25 °C. A portion of $AgNO_3$ (25.5 mg, 0.15 mmol) was added. The reaction was stirred in the dark at rt for 16 h. The mixture was centrifuged (12000 rpm, 5 min) to remove the solid byproduct. The supernatant was passed through a 0.20 μm PTFE syringe filter. The filtrate was collected. In a separate reaction vessel, doxorubicin hydrochloride (58.0 mg, 0.10 mmol) in DMF (1.0 mL) was mixed with $AgNO_3$ (17.0 mg, 0.10 mmol). The white precipitate was removed by centrifugation (12000 rpm, 5 min). The supernatant was passed through a 0.20 μm PTFE syringe filter. The doxorubicin filtrate was neutralized with NaOH aq. solution (1 M, 0.10 mmol, 100 μL), resulting in a dark red mixture with a small amount of brown precipitate. This mixture was then added dropwise to the platinum agent-containing filtrate. The reaction mixture was stirred in the dark at rt for 24 h. DMF was removed under vacuum on a rotary evaporator with the water bath temperature at 40 °C. The crude product was suspended in water (0.5 mL), and the suspension was centrifuged (12000 rpm, 5 min). The supernatant was passed through a 0.20 μm nylon syringe filter. The crude product was loaded on a reverse phase BUCHI EcoFlex flash chromatography C18 cartridge (12 g). The purification used a (A) water / (B) MeOH solvent system, according to the following protocol: constant flow rate 12.0 $mL{\cdot}min^{-1}$; 0.0-3.0 column volume (CV), 0% B; 3.0-13.0 CV, linear gradient 0-100% B; 13.0-20.0 CV, 100% B. Fractions from CV4.5 to CV10 were collected and filtered through a 0.2 μm PTFE syringe filter. The combined fractions were further purified by preparative HPLC. Preparative HPLC used a (A) water (0.1% v/v CF_3CO_2H) / (B) CH_3CN (0.1% v/v CF_3CO_2H) solvent system, according to the following protocol: constant flow rate 15.0 $mL{\cdot}min^{-1}$; 0.0-2.0 min, 30% B; 2.0-13.0 min, linear gradient 30-41% B; 13.0-14.0 min, linear gradient 41-100% B; 14.0-15.0 min, 100% B; 15.0-17.0 min, linear gradient 100-30% B; 17.0-18.0 min, 30% B. Fractions containing DoxPt4 (T_R = 8.3 min) and DoxPt5 (T_R = 11.3 min) were collected separately and lyophilized to give DoxPt4 as a bright orange solid (26.9 mg, a trifluoroacetate salt, 29% yield) and DoxPt5 as a bright orange solid (18.1 mg, a trifluoroacetate salt, 17% yield). Compound DoxPt5 obtained from preparative HPLC contained a small amount of impurity (about 10%) corresponding to a $[Dox_2Pt(NH_3)_2]^{2+}$-containing salt, as indicated by MS studies (ESI-MS(+) m/z calcd $[M-H]^+$ 1314.4, found 1314.3). Analytically pure DoxPt5 was isolated with a semipreparative HPLC (T_R = 9.9 min) using a (A) water (0.1% v/v CF_3CO_2H) / (B) CH_3CN (0.1% v/v CF_3CO_2H) solvent system, according to the following protocol: constant flow rate 3.0 $mL{\cdot}min^{-1}$; 0.0-3.0 min, 10% B; 3.0-5.0 min, linear gradient 10-40% B; 13.0-14.0 min, linear gradient 41-100% B; 14.0-15.0 min, 100% B; 15.0-17.0 min, linear gradient 100-30% B; 17.0-18.0 min, 30% B. The purity of DoxPt4 and DoxPt5 was examined by analytical HPLC (T_R = 16.8 min for DoxPt4 and T_R = 18.1 min for DoxPt5). Analytical HPLC used a (A) water / (B) MeOH solvent system, according to the

following protocol: constant flow rate 1.0 mL·min^{-1}; 0.0-5.0 min, 10% B; 5.0-30.0 min, linear gradient 10-100% B; 30.0-33.0 min, 100% B; 33.0-36.0 min, linear gradient 100-10% B; 36.0-40.0 min, 10% B.

^{1}H NMR spectroscopy of DoxPt4 in DMF-d_7 shows two sets of signals, presumably corresponding to two species, with at least one as an adduct with a trifluoroacetate anion. Upon the addition of water (5 vol %) in DMF-d_7, the two sets of signals coalesced. Because of ^{1}H NMR signal broadening in D_2O, we were unable to obtain interpretable information. The ^{19}F NMR spectrum of DoxPt4 in D_2O showed only one major signal, instead of two, as observed in DMF-d_7. We tentatively attribute this observation to the dissociation of trifluoroacetate from platinum in D_2O. As revealed by HPLC analysis, DoxPt5 underwent slow aquation in water at rt to form DoxPt4 (Figure S30). Given the labile nature of DoxPt5 in water and the relatively simple speciation of DoxPt4 in water and ease of purification, DoxPt4 was used in the *in vitro* anticancer activity investigations.

Characterization of DoxPt4

^{1}H NMR (400 MHz, DMF-d_7) δ 14.30 – 14.24 (m, 1H), 13.39 – 13.32 (m, 1H), 8.01 – 7.94 (m, 2H), 7.79 – 7.70 (m, 1H), 5.78 – 5.23 (m, 5H, NH_2 and NH_3, partially overlapping with $H_{1'}$), 5.43 – 5.39 (m, 1H, partially overlapping with NH_2 and NH_3), 5.12 (s, 1H), 4.95 (br, 3H, NH_3), 4.85 – 4.68 (m, 3H), 4.56 (br, 1H), 4.40 – 4.20 (m, 1H), 4.17 – 4.10 (partially overlapping with $H_{7'}$, 1H), 4.11 (two s signals, 3H), 3.37 – 3.22 (m, 1H), 3.21 – 2.94 (m, 2H), 2.45 – 2.19 (m, 2H), 2.18 – 1.83 (m, 2H), 1.31 – 1.23 (m, 3H). Proton 28 was not observed in DMF-d_7. Two sets of signals were observed in DMF-d_7, which coalesced in DMF-d_7:D_2O (95:5, v/v). ^{13}C{^{1}H} NMR (101 MHz, DMF-d_7) δ 215.06, 214.84, 188.04, 187.96, 162.38, 160.20 (q, J = 33.0 Hz).157.62, 157.60, 156.13, 156.11, 137.38, 137.33, 136.68, 136.60, 136.20, 135.40, 121.42, 120.70, 120.17, 118.57 (q, J = 289.9 Hz), 112.16, 112.06, 112.01, 101.27, 100.98, 76.82, 76.66, 71.15, 70.43, 70.12, 69.96, 68.07, 67.97, 65.62, 65.48, 57.43, 57.42, 53.47, 52.26, 37.83, 37.40, 33.81, 33.73, 32.13, 32.01, 17.60, 17.51. Aside from the ^{13}C NMR signals of trifluoroacetate, only 45 carbon signals were observed, presumably due to partial overlap between signals from two species. ^{195}Pt NMR (86 MHz, DMF-d_7) δ −2139.85. ^{19}F NMR (377 MHz, DMF-d_7) δ −74.71 (br), −74.74. ^{1}H and ^{19}F quantitative nuclear magnetic resonance (qNMR) studies indicate that the compound was isolated as a trifluoroacetate salt with a doxorubicin backbone:trifluoroacetate ratio of 1.00:2.34. Mass spectrometry studies were performed using a stock solution of DoxPt4 freshly prepared in water. ESI-MS(+) m/z calcd [M]$^{+}$ 771.2, found 771.1. HRMS (ESI/Q-TOF) m/z: [M]$^{+}$ Calcd for $C_{27}H_{34}N_3O_{11}Pt^{+}$ 771.1836; Found 771.1884. In the MS/MS experiment, the fragments of molecular ion (M^{+}) of m/z = 771.2 confirm the structure of DoxPt4.

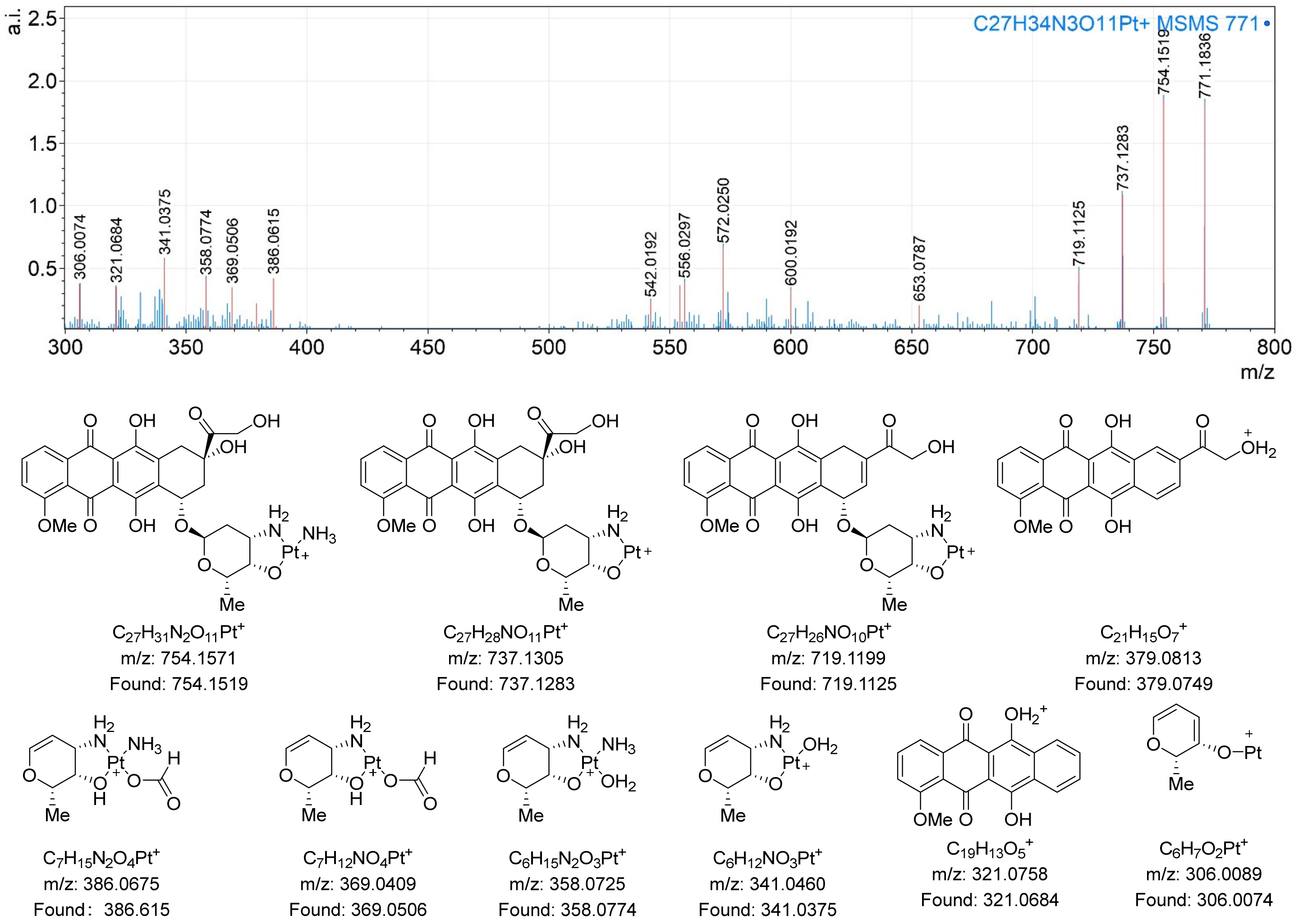

Figure S3. Tandem mass spectrum of DoxPt4 and the fragmentation analysis.

Characterization of DoxPt5

^{1}H NMR (400 MHz, DMF-d_7) δ 14.24 (s, 1H), 13.35 (s, 1H), 7.97 – 7.95 (m, 2H), 7.72 (pseudo t, J = 4.8 Hz, 1H), 5.53 (s, 1H), 5.35 (d, J = 3.3 Hz, 1H), 5.30 (d, J = 4.1 Hz, 1H), 5.08 (dd, J = 4.4, 3.0 Hz, 1H), 5.01 (d, J = 6.3 Hz, 2H), 4.77 (s, 6H), 4.30 (q, J = 6.8 Hz, 1H), 4.25 (s, 1H), 4.20 (s, 3H), 4.09 (s, 3H), 3.40 – 3.21 (m, 1H), 3.06 (ABq, $\Delta\delta_{AB}$ = 0.10, J_{AB} = 18.3 Hz, 2H), 2.38 (d, J = 13.4 Hz, 1H), 2.25 (dd, J = 14.5, 5.4 Hz, 1H), 2.13 (dd, J = 13.1, 4.8 Hz, 1H), 1.88 (dt, J = 12.9, 3.8 Hz, 1H), 1.27 (d, J = 6.5 Hz, 3H). ^{13}C{^{1}H} NMR (101 MHz, DMF-d_7) δ 215.06, 188.05, 187.98, 162.38 (q, J = 37.3 Hz, partially overlapping with DMF-d_7 signal and C4), 162.38, 157.69, 156.16, 137.33, 136.63, 136.24, 135.41, 121.45, 120.69, 120.18, 118.87 (q, J = 292.4 Hz), 112.15, 112.05, 101.68, 76.75, 71.42, 70.09, 67.90, 65.59, 57.43, 52.83, 37.75, 33.69, 32.60, 17.64. ^{19}F NMR (377 MHz, DMF-d_7) δ −74.80. ^{195}Pt NMR (86 MHz, DMF-d_7) δ −2422. ^{1}H and ^{19}F quantitative nuclear magnetic resonance (qNMR) studies indicate that the compound was isolated as a trifluoroacetate salt with a doxorubicin backbone: trifluoroacetate ratio of 1.00:1.09. Mass spectrometry studies were performed using a stock solution of DoxPt5 freshly prepared in water. ESI-MS(+) m/z calcd [M]$^+$ 808.2, found 808.0. HRMS (ESI/Q-TOF) m/z: [M]$^+$ Calcd

for $C_{27}H_{35}ClN_3O_{11}Pt^+$, 807.1603; Found: 807.1632. In the MS/MS experiment, the fragments of molecular ion (M^+) of m/z = 808.2 confirm the structure of DoxPt5.

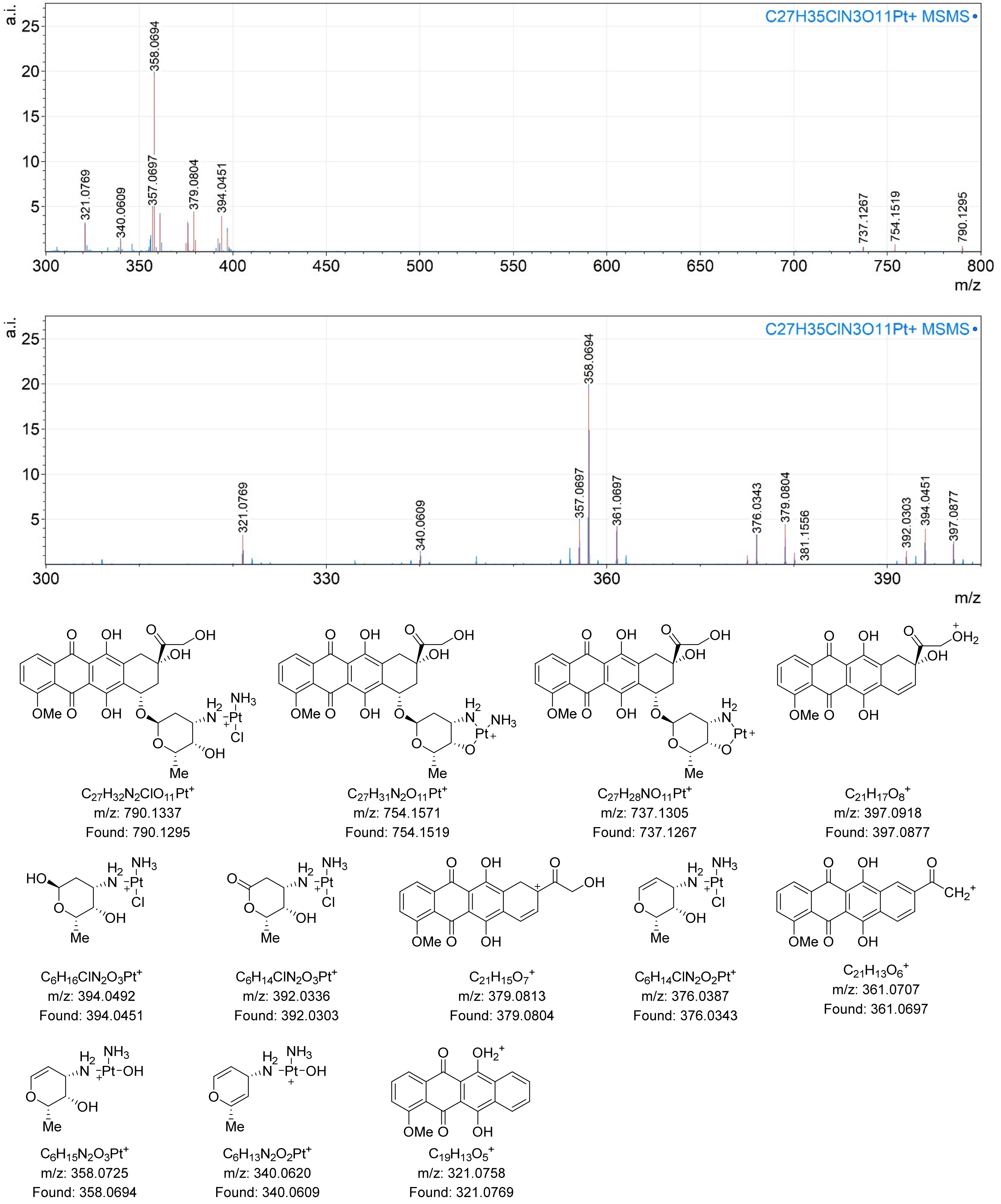

Figure S4. Tandem mass spectrum of DoxPt5 and the fragmentation analysis.

S3. Stability of DoxPt.

Stability of DoxPt in pH 7.4 HEPES buffer

An aliquot (100 µL) of a stock solution of DoxPt in EtOH-water (50:50, v:v, 1 mM) was mixed with HEPES buffer (10 mM, pH = 7.4, NaCl 10 mM, 900 µM, final conc. of DoxPt = 100 µM). This solution was stored at rt in the dark. For analytical HPLC studies, an aliquot (50 µL) of the diluted solution was analyzed by HPLC at the indicated times and eluted with a (A) water / (B) MeOH solvent system, according to the following protocol: constant flow rate 1.0 mL·min^{-1}; 0.0-5.0 min, 10% B; 5.0-30.0 min, linear gradient 10-100% B; 30.0-33.0 min, 100% B; 33.0-36.0 min, linear gradient 100-10% B; 36.0-40.0 min, 10% B. After 24 h, a red precipitate formed. Little DoxPt remained in the buffer. The red precipitate was dissolved in MeOH and analyzed with HPLC. Both HPLC and LC-MS analyses suggested that the precipitate was intact DoxPt. This observation is consistent with the reported slow aquation of oxaliplatin in pH 7.4 HEPES buffer (half-life ≈ 160 h at 37 °C).[6]

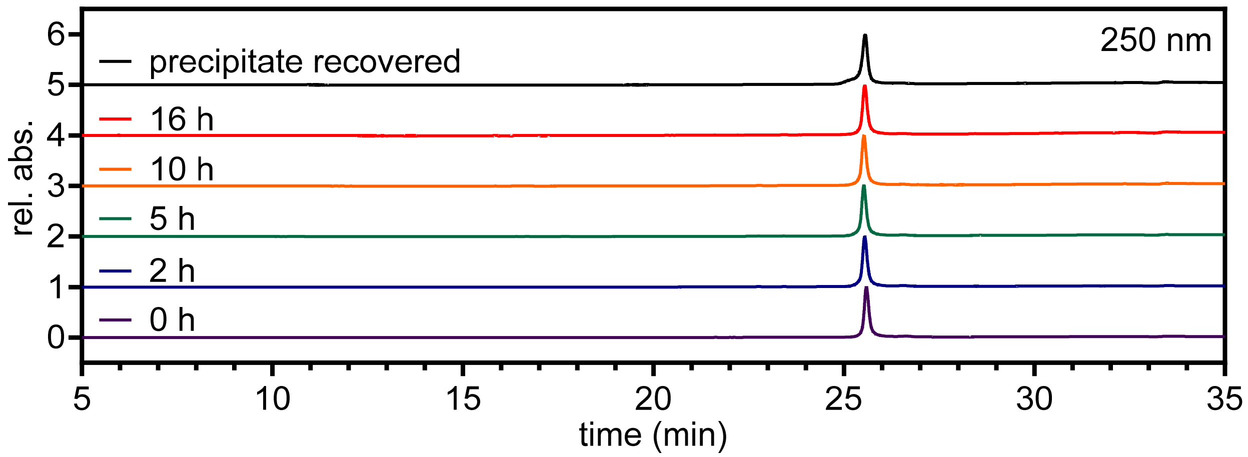

Figure S5. Analytical HPLC chromatograms of DoxPt in pH 7.4 HEPES buffer at rt analyzed over time. Solvent gradient is given in the text.

Stability of DoxPt in a 5:95 EtOH-water mixture

An aliquot (30 µL) of a stock solution of DoxPt in EtOH-water (50:50, v:v, 1 mM) was mixed with MilliQ water (300 µL, final conc. of DoxPt ≈ 100 µM). This solution was stored at rt in the dark. For analytical HPLC studies, an aliquot (30 µL) of the diluted solution was analyzed by HPLC at the indicated times and eluted with a (A) water / (B) MeOH solvent system, according to the following protocol: constant flow rate 1.0 mL·min^{-1}; 0.0-5.0 min, 10% B; 5.0-30.0 min, linear gradient 10-100% B; 30.0-33.0 min, 100% B; 33.0-36.0 min, linear gradient 100-10% B; 36.0-40.0 min, 10% B.

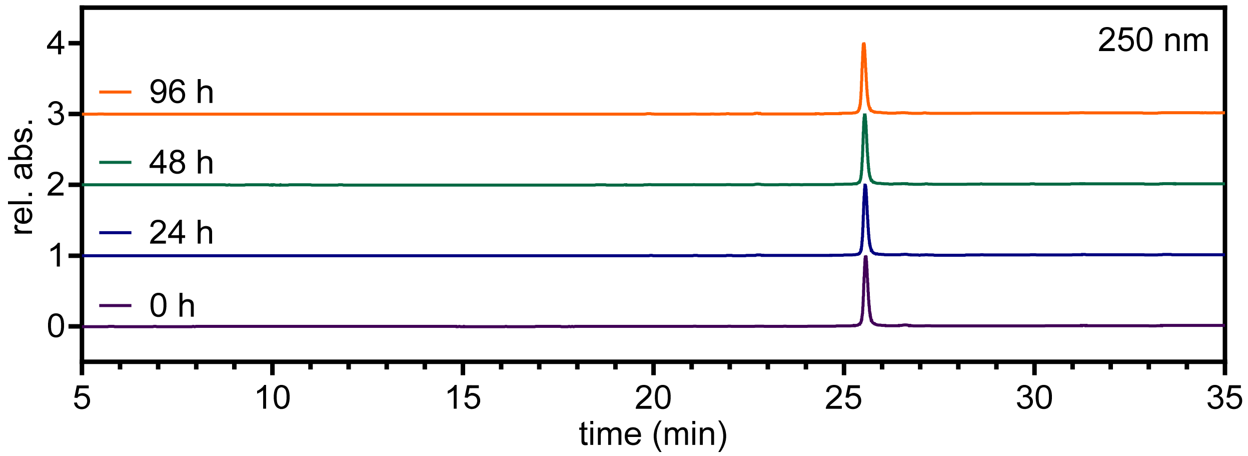

Figure S6. Analytical HPLC chromatograms of DoxPt in an EtOH-water mixture (5:95, v:v) at rt analyzed over time. Solvent gradient is given in the text.

Stability of DoxPt in DMF

A portion of DoxPt (0.5 mg) was dissolved in DMF (500 μL). This solution was stored at rt in the dark. For analytical HPLC studies, an aliquot (30 μL) of the diluted solution was analyzed by HPLC at the indicated times and eluted with a (A) water / (B) MeOH solvent system, according to the following protocol: constant flow rate 1.0 mL·min^{-1}; 0.0-5.0 min, 10% B; 5.0-30.0 min, linear gradient 10-100% B; 30.0-33.0 min, 100% B; 33.0-36.0 min, linear gradient 100-10% B; 36.0-40.0 min, 10% B.

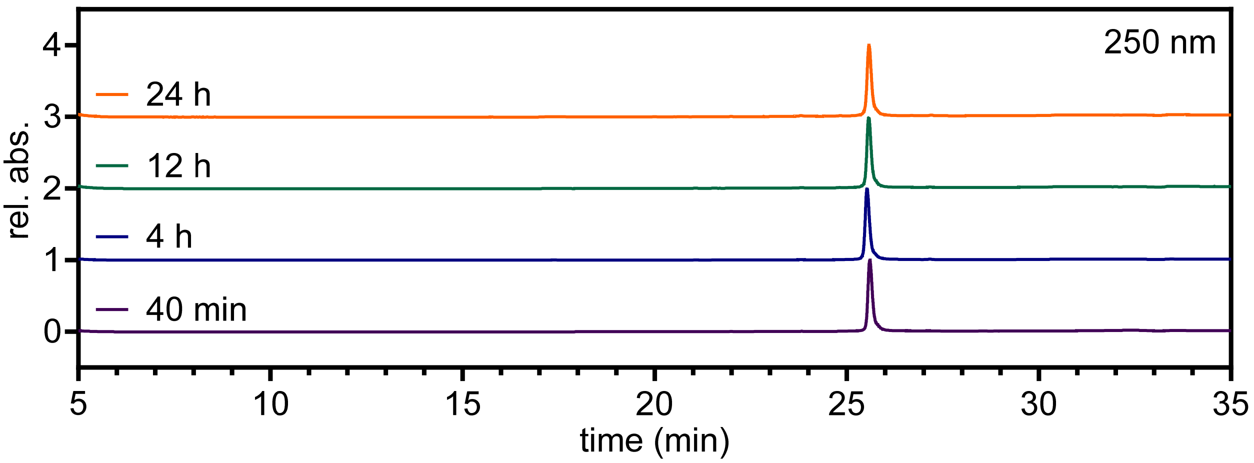

Figure S7. Analytical HPLC chromatograms of DoxPt in DMF at rt analyzed over time. Solvent gradient is given in the text.

Stability of DoxPt in DMSO

A portion of DoxPt (0.5 mg) was dissolved in dry DMSO (500 μL). This solution was stored at rt in the dark. For analytical HPLC studies, an aliquot (30 μL) of the diluted solution was analyzed by HPLC at the indicated times and eluted with a (A) water / (B) MeOH solvent system, according to the following protocol: constant flow rate 1.0 mL·min^{-1}; 0.0-5.0 min, 10% B; 5.0-30.0 min, linear gradient 10-100% B; 30.0-33.0 min, 100% B; 33.0-36.0 min, linear gradient 100-10% B; 36.0-40.0 min, 10% B.

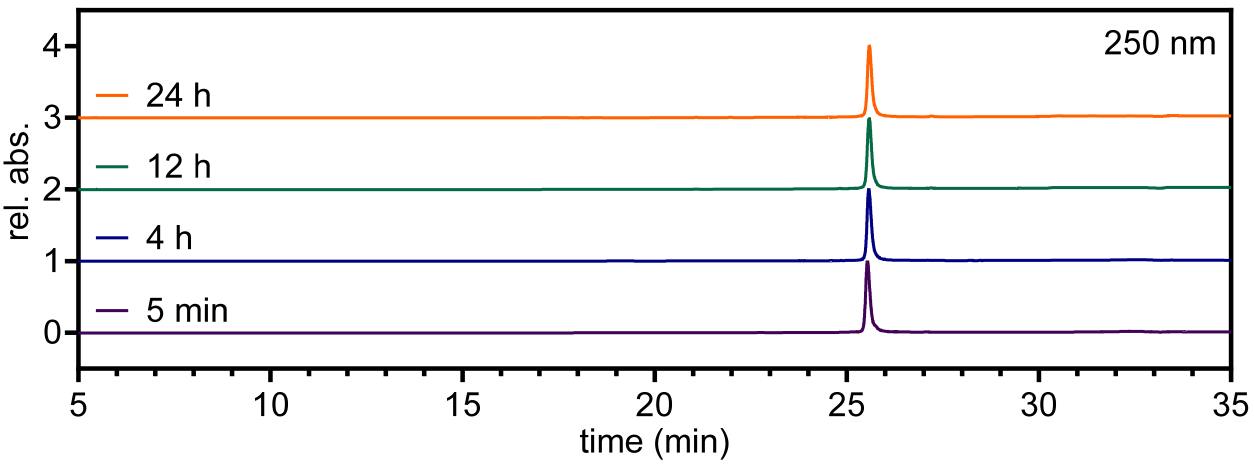

Figure S8. Analytical HPLC chromatograms of DoxPt in DMSO at rt analyzed over time. Solvent gradient is given in the text.

Analysis of DoxPt precipitate in glycerol:water (1:1 v:v) mixture stored at −20 °C

At about 1 mM, DoxPt is fully soluble in glycerol:water (1:1 v:v) at 65 °C but precipitates at −20 °C, which dissolves at 65 °C. To confirm that the precipitate is intact DoxPt, a mixture of DoxPt in glycerol:water (1:1 v:v, 1.12 mM) was heated at 65 °C to generate a solution and then stored at −20 °C for 12 h. The mixture was centrifuged (12000 rpm, 5 min). The supernatant was removed. The red solid was dissolved in MeOH and analyzed by HPLC a the (A) water / (B) MeOH solvent system, according to the following protocol: constant flow rate 1.0 mL·min−1; 0.0-5.0 min, 10% B; 5.0-30.0 min, linear gradient 10-100% B; 30.0-33.0 min, 100% B; 33.0-36.0 min, linear gradient 100-10% B; 36.0-40.0 min, 10% B. This solution was also analyzed by ESI-LCMS and confirmed to be intact DoxPt.

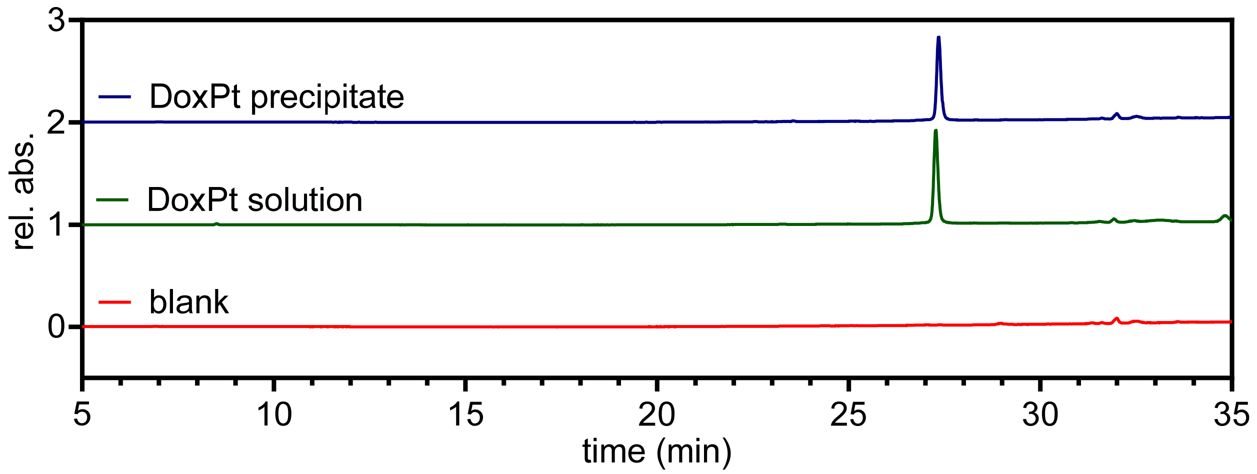

Figure S9. Analytical HPLC chromatograms of DoxPt precipitate in glycerol:water (1:1 *v:v*). Solvent gradient is given in the text.

Thermostability of DoxPt in glycerol:water (1:1 v:v) after heating-freezing cycles

A mixture of DoxPt in glycerol:water (1:1 v:v, 1.12 mM) was heated at 65 °C for 5 min in the dark and frozen at −20 °C for 5 min in the dark. An aliquot of this solution (5.0 µL) was diluted in MeOH (95 µL) and analyzed by HPLC. For analytical HPLC studies, an aliquot (15 µL) of the diluted solution was analyzed by HPLC at the indicated times and eluted with a (A) water / (B) MeOH solvent system, according to the following protocol: constant flow rate 1.0 mL·min^{-1}; 0.0-5.0 min, 10% B; 5.0-30.0 min, linear gradient 10-100% B; 30.0-33.0 min, 100% B; 33.0-36.0 min, linear gradient 100-10% B; 36.0-40.0 min, 10% B. This experiment was repeated in the same fashion, and the solution was analyzed after 10 and 20 heating-freezing cycles. The solution was also analyzed by ESI-LCMS after 20 heating-freezing cycles. No decomposition was observed.

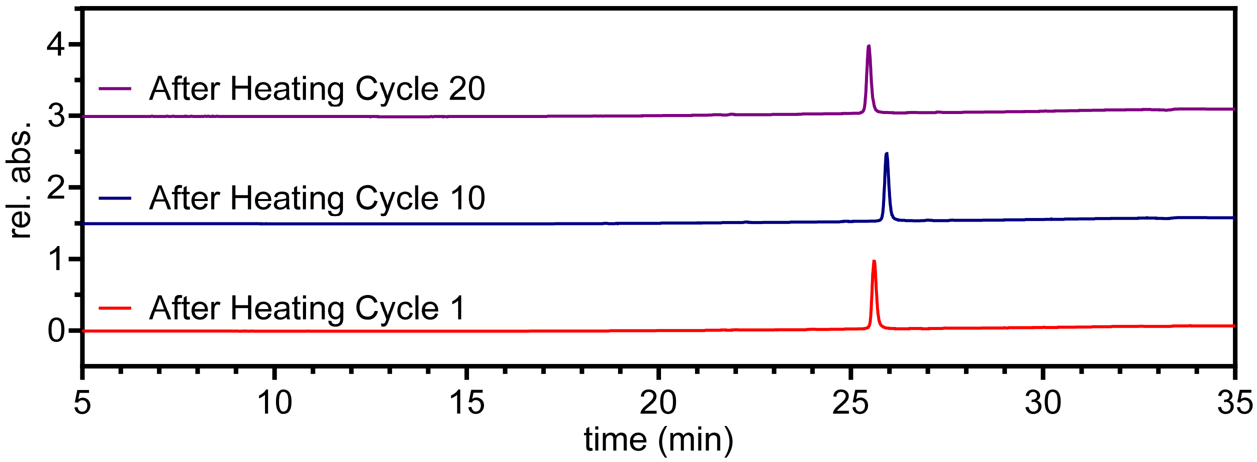

Figure S10. Analytical HPLC chromatograms of DoxPt in glycerol:water (1:1 *v:v*) after heating-freezing cycles. Solvent gradient is given in the text.

S4. Reactivity of Oxaliplatin and DoxPt with different sulfur-containing small molecules.

Monitoring the reaction of 50 µM DoxPt with sulfur-containing biomolecules at 37 °C by HPLC with H_2O/MeOH mobile phase.

Typical procedure for the reaction of 50 µM DoxPt with 100 µM sulfur-containing biomolecules

An aliquot of DoxPt in glycerol:water (1:1 v:v, 1.12 mM, 8.38 µL) and an aqueous solution of sulfur-containing biomolecule (2.00 mM, 9.38 µL) or water (as the control, 9.38 µL) were added to pH 7.0, 50 mM PIPES buffer (151.0 µL) containing 100 mM KCl and MeOH (18.75 µL), which was used to improve the solubility of DoxPt in the reaction mixture. This mixture was heated at 37 °C in the dark. The progress of the reaction was monitored by analytical HPLC using a (A) water / (B) MeOH solvent system, according to the following protocol: constant flow rate 1.0 mL·min^{-1}; 0.0-3.0 min, 0% B; 3.0-7.0 min, linear gradient 0-60% B; 7.0-21.0 min, linear gradient 60-74% B; 21.0-22.0 min, 100% B; 22.0-23.0 min, 100% B; 23.0-27.0 min, linear gradient 100-0% B; 27.0-30.0 min, 0% B.

These studies suggested that DoxPt is relatively stable in the presence of sulfur-containing species at low concentrations and retains its speciation as a conjugate of platinum and $DoxNH_2NH_2$ backbone.

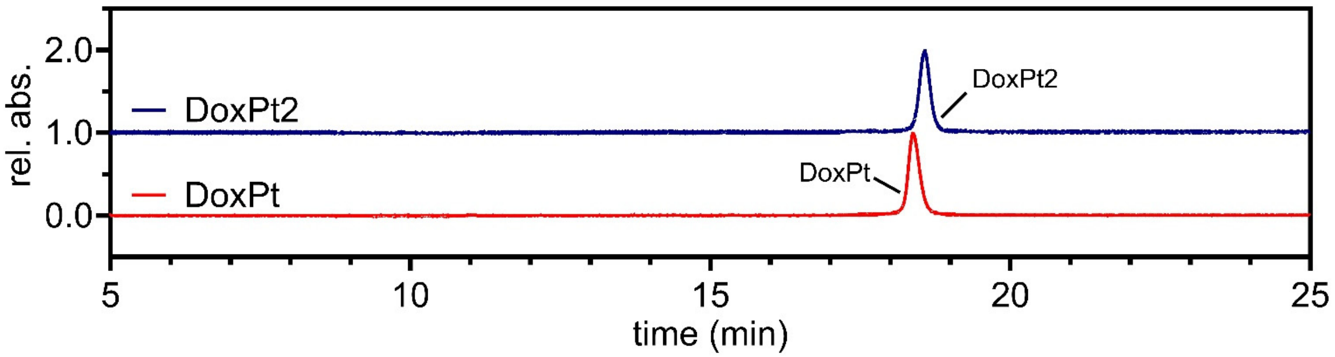

Figure S11. HPLC chromatograms (250 nm) showing the retention time of DoxPt and DoxPt2. Solvent gradient is given in the text.

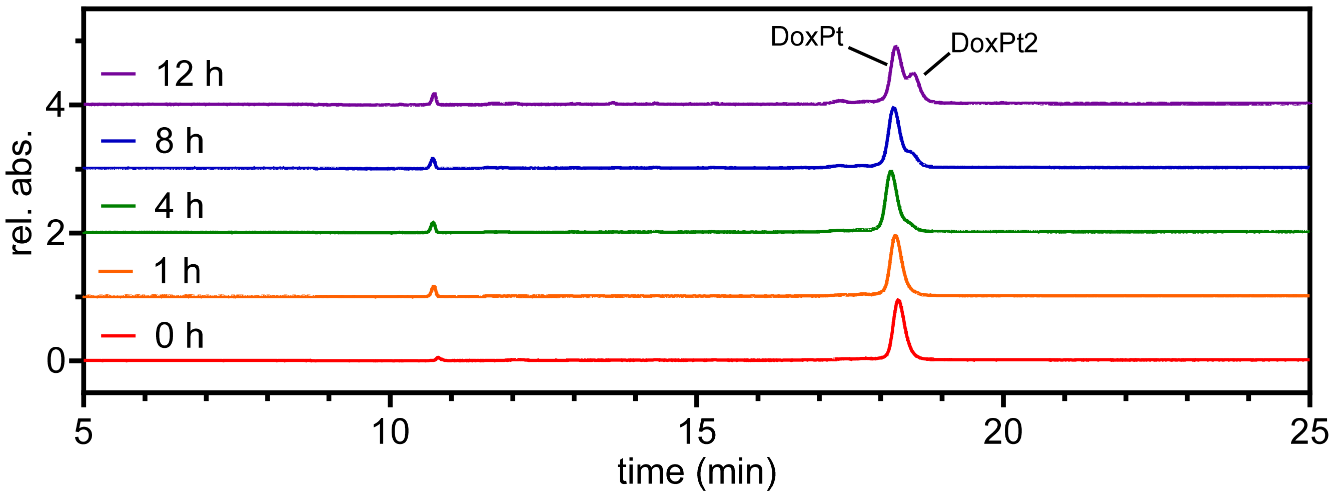

Figure S12. HPLC chromatograms (250 nm) showing the slow conversion of DoxPt (50 µM) to DoxPt2 in pH 7.0 PIPES buffer containing 100 mM KCl and 10 vol % MeOH at 37 °C. Solvent gradient is given in the text.

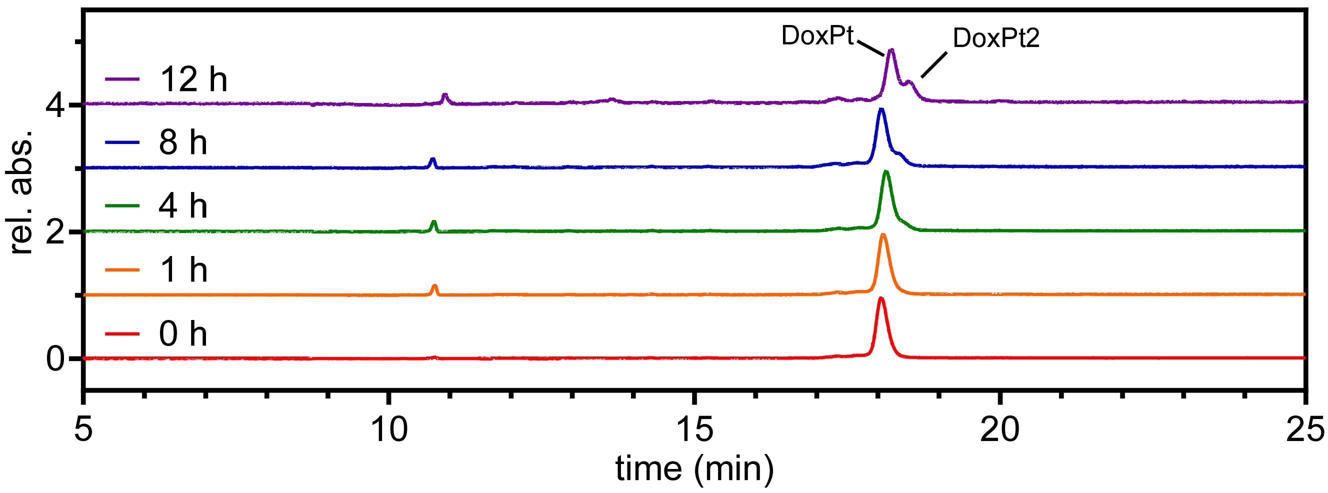

Figure S13. HPLC chromatograms (250 nm) showing the conversion of DoxPt (50 µM) in the presence of GSH (100 µM) in pH 7.0 PIPES buffer containing 100 mM KCl and 10 vol % MeOH at 37 °C. Solvent gradient is given in the text.

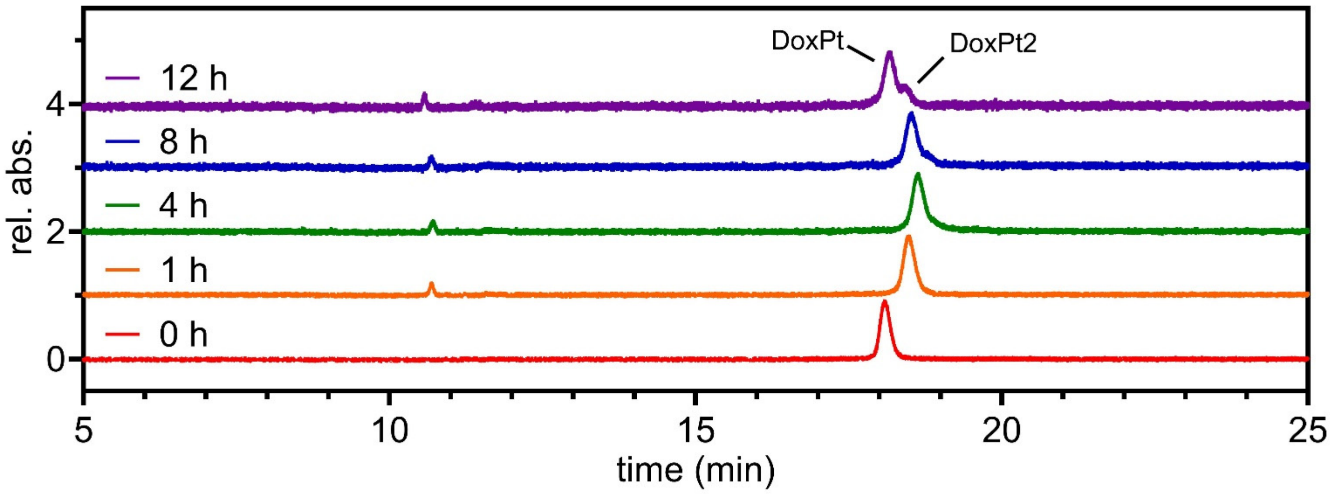

Figure S14. HPLC chromatograms (250 nm) showing the conversion of DoxPt (50 µM) in the presence of (L)-cysteine (100 µM) in pH 7.0 PIPES buffer containing 100 mM KCl and 10 vol % MeOH at 37 °C. Solvent gradient is in the text.

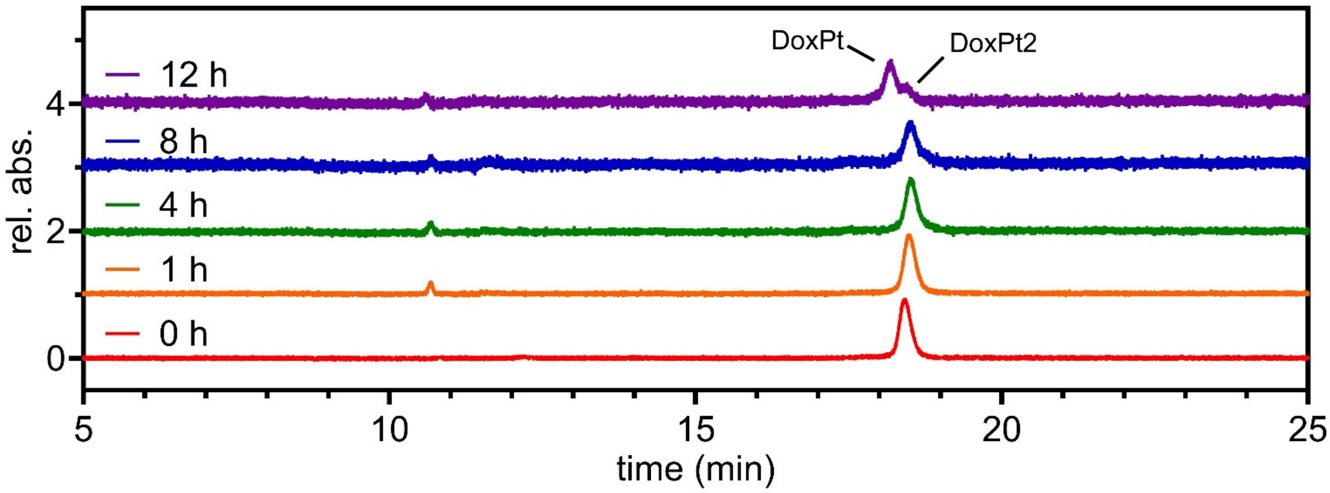

Figure S15. HPLC chromatograms (250 nm) showing the conversion of DoxPt (50 µM) in the presence of (L)-cysteine (200 µM) in pH 7.0 PIPES buffer containing 100 mM KCl and 10 vol % MeOH at 37 °C. Solvent gradient is in the text.

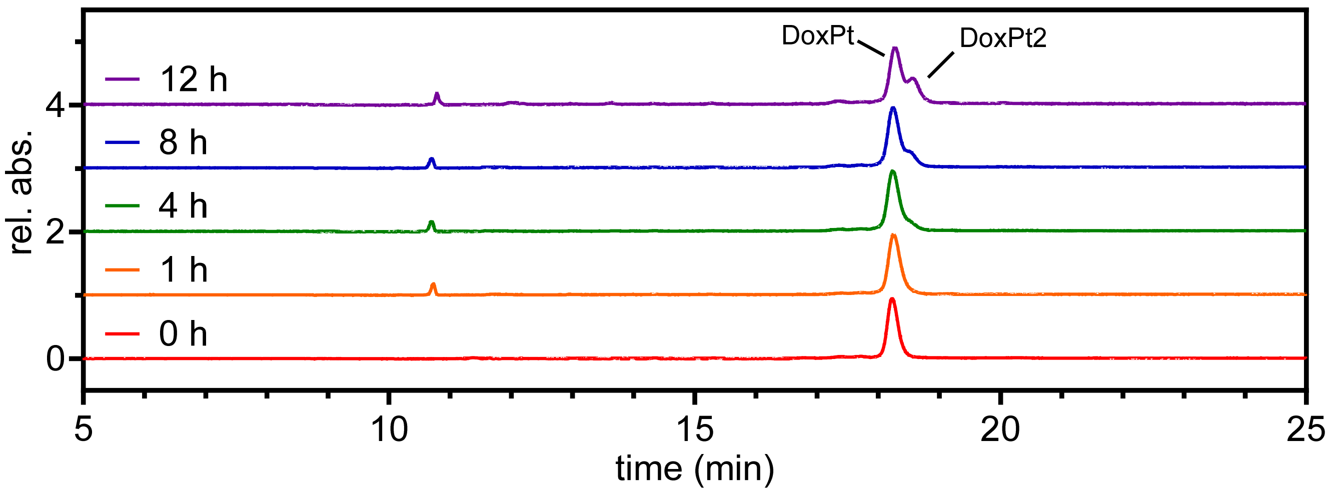

Figure S16. HPLC chromatograms (250 nm) showing the conversion of DoxPt (50 μM) in the presence of GSSG (100 μM) in pH 7.0 PIPES buffer containing 100 mM KCl and 10 vol % MeOH at 37 °C. Solvent gradient is given in the text.

Monitoring the reaction of 50 µM oxaliplatin with (L)-cysteine at 37 °C by HPLC with H_2O/MeOH mobile phase.

Typical procedure for the reaction of 50 µM oxaliplatin with 100 µM (L)-cysteine

An aliquot of oxaliplatin (Ox) in water (10.0 mM, 10.00 µL) and an aqueous solution of (L)-cysteine (20.0 mM, 10.00 µL) were added to pH 7.0, 50 mM PIPES buffer (1780 µL) containing 100 mM KCl and MeOH (200.0 µL). This mixture was heated at 37 °C in the dark. The progress of the reaction was monitored by analytical HPLC using a (A) water / (B) MeOH solvent system, according to the following protocol: constant flow rate 1.0 mL·min^{-1}; 0.0-3.0 min, 0% B; 3.0-7.0 min, linear gradient 0-25% B; 7.0-12.0 min, linear gradient 25-100% B; 12.0-13.0 min, 100% B; 13.0-16.0 min, linear gradient 100-0% B; 16.0-20.0 min, 0% B.

In these studies, oxaliplatin exhibited a reactivity similar to that of DoxPt.

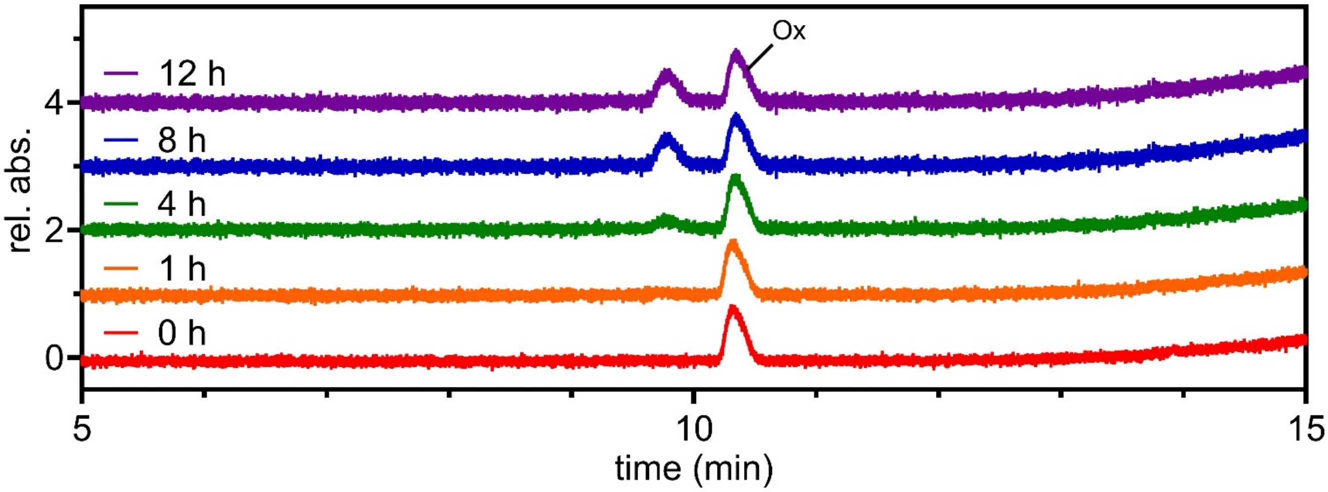

Figure S17. HPLC chromatograms (220 nm) showing the conversion of Ox (50 µM) in pH 7.0 PIPES buffer containing 100 mM KCl and 10 vol % MeOH at 37 °C. Solvent gradient is in the text.

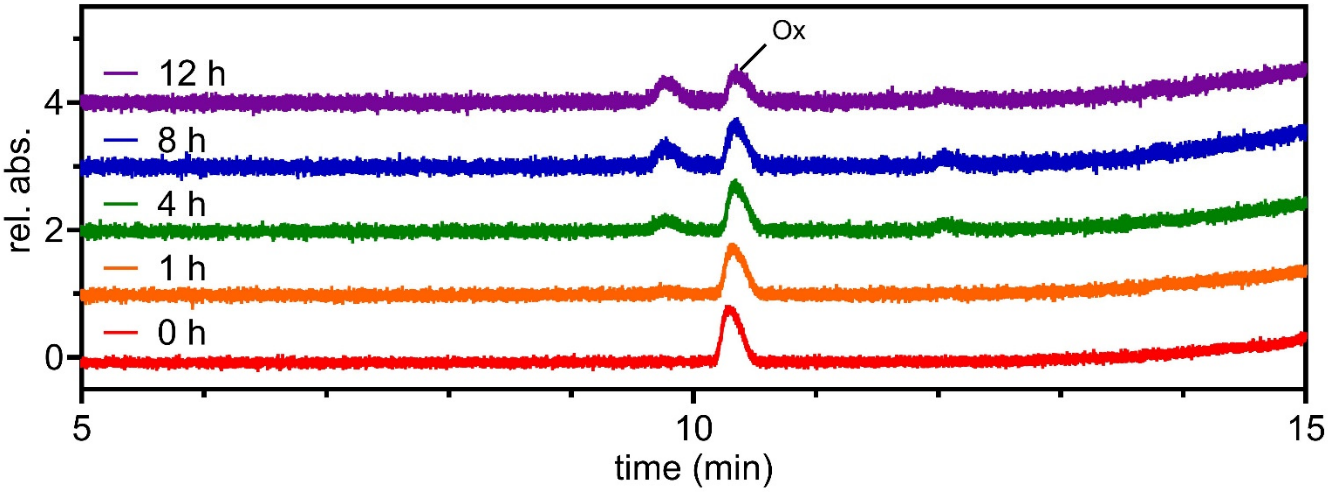

Figure S18. HPLC chromatograms (220 nm) showing the conversion of Ox (50 µM) in the presence of (L)-cysteine (100 µM) in pH 7.0 PIPES buffer containing 100 mM KCl and 10 vol % MeOH at 37 °C. Solvent gradient is in the text.

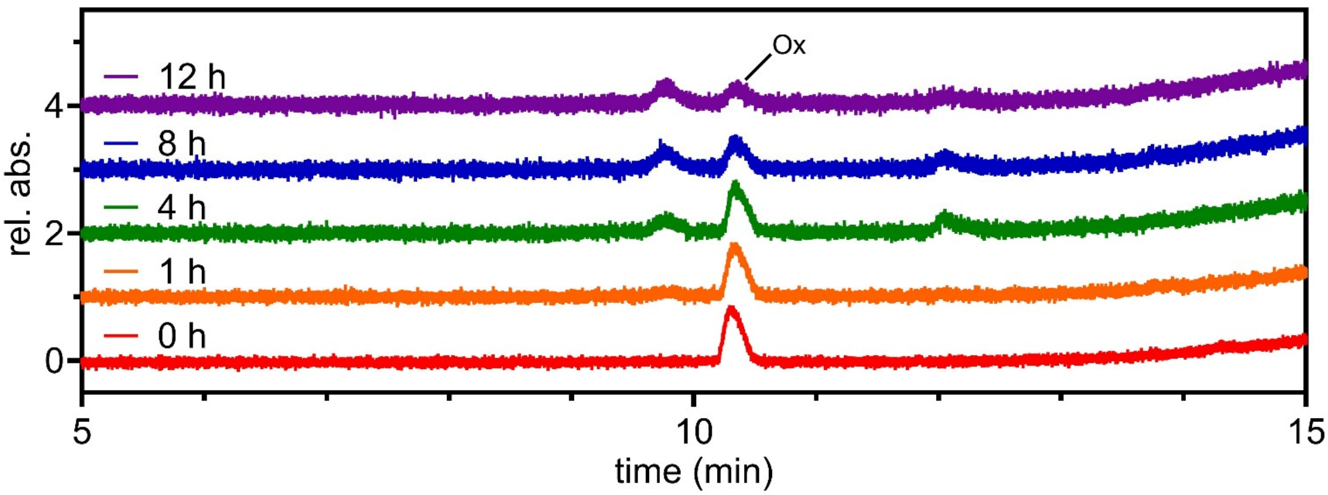

Figure S19. HPLC chromatograms (220 nm) showing the conversion of Ox (50 µM) in the presence of (L)-cysteine (200 µM) in pH 7.0 PIPES buffer containing 100 mM KCl and 10 vol % MeOH at 37 °C. Solvent gradient is in the text.

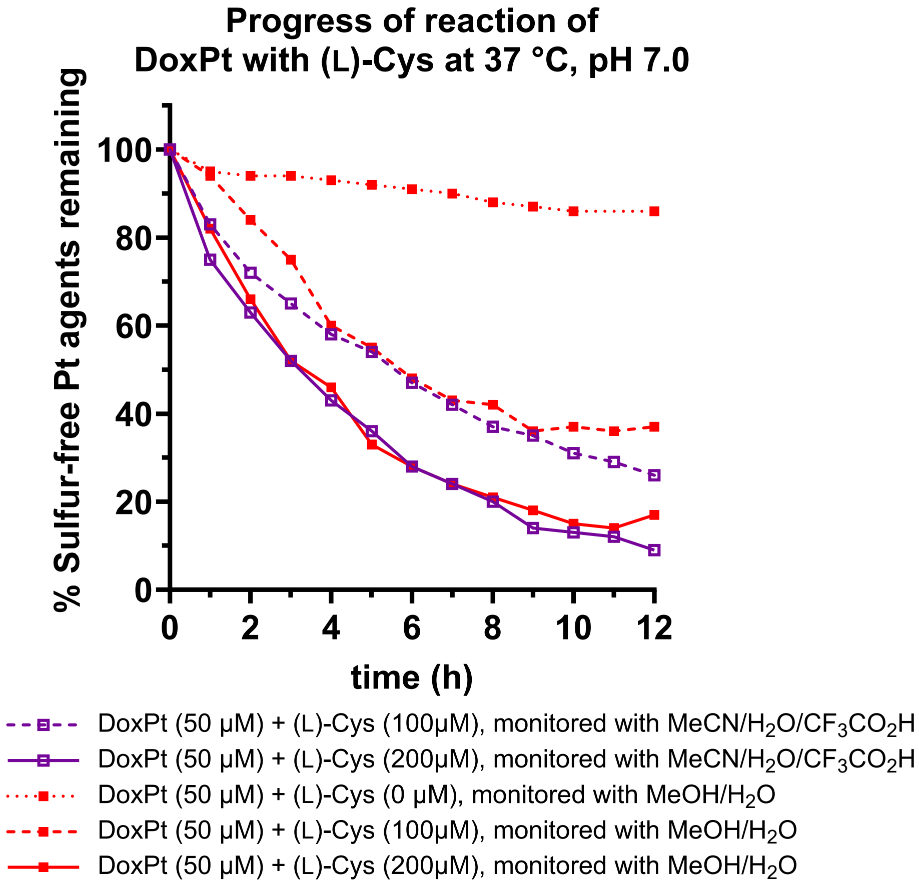

Figure S20. The progress of the reaction of DoxPt with 0 µM, 100 µM, and 200 µM (L)-cysteine. Solvent gradients are in the text.

Monitoring the reaction of 50 μM DoxPt with sulfur-containing small molecules at 37 °C by HPLC with water/CH_3CN/0.1% v/v CF_3CO_2H mobile phase.

Typical procedure for the reaction of 50 μM DoxPt with 200 μM sulfur-containing biomolecules

To further confirm the strong covalent binding between the $DoxNH_2NH_2$ backbone and the platinum pharmacophore, we monitored the reaction between DoxPt and sulfur-containing species at biologically or physiologically relevant concentrations (200 μM and 1.0 mM) by HPLC. We used a water/CH_3CN/0.1% v/v CF_3CO_2H mobile phase, in which $DoxNH_2NH_2$ is better separated. We tested reactions with various sulfur-containing species, including GSH, GSSG, (L)-cystine, and (L)-cysteine, which are commonly found in cell culture media, in the cell, or as model thiol-containing biomolecules.

Experimentally, for the reaction with GSH, GSSG, or (L)-cysteine at 200 μM, an aliquot of DoxPt in glycerol:water (1:1 v:v, 1.12 mM, 11.17 μL) and an aqueous solution of sulfur-containing compounds (2.00 mM, 25 μL) were added to pH 7.0, 50 mM PIPES buffer (188.8 μL) containing 100 mM KCl and MeOH (18.75 μL). For the reaction with (L)-cystine at 200 μM, an aliquot of DoxPt in glycerol:water (1:1 v:v, 1.12 mM, 11.17 μL) and a solution of (L)-cystine in pH 7.0 PIPES buffer with 100 mM KCl (400 μM, 125 μL) were added to pH 7.0, 50 mM PIPES buffer (88.8 μL) containing 100 mM KCl and MeOH (25.0 μL). For the reaction with GSH, GSSG, or (L)-cysteine at 1.00 mM, an aliquot of DoxPt in glycerol:water (1:1 v:v, 1.12 mM, 11.17 μL) and an aqueous solution of sulfur-containing compounds (200 mM, 1.25 μL) were added to pH 7.0, 50 mM PIPES buffer (212.6 μL) containing 100 mM KCl and MeOH (25.0 μL). The reaction mixture was heated at 37 °C in the dark. The progress of the reaction was monitored by analytical HPLC using a (A) water (0.1% v/v CF_3CO_2H) / (B) CH_3CN (0.1% v/v CF_3CO_2H) solvent system, according to the following protocol: constant flow rate 1.0 mL·min^{-1}; 0.0-3.0 min, 10% B; 3.0-23.0 min, linear gradient 10-60% B; 23.0-25.0 min, linear gradient 60-100% B; 25.0-26.0 min, 100% B; 26.0-28.0 min, 10% B; 28.0-30.0 min, 0% B.

As shown in **Figure S26**, DoxPt slowly reacted with thiol- and disulfide-containing compounds, particularly at a higher concentration of 1.0 mM, leading to a mixture of multiple products. This result is also consistent with our protein binding studies (Section 6). Nonetheless, the dissociation of $DoxNH_2NH_2$ ligand and the platinum was not observed or was negligible, supporting the design that the platinum pharmacophore is incorporated in a covalent, non-cleavable fashion.

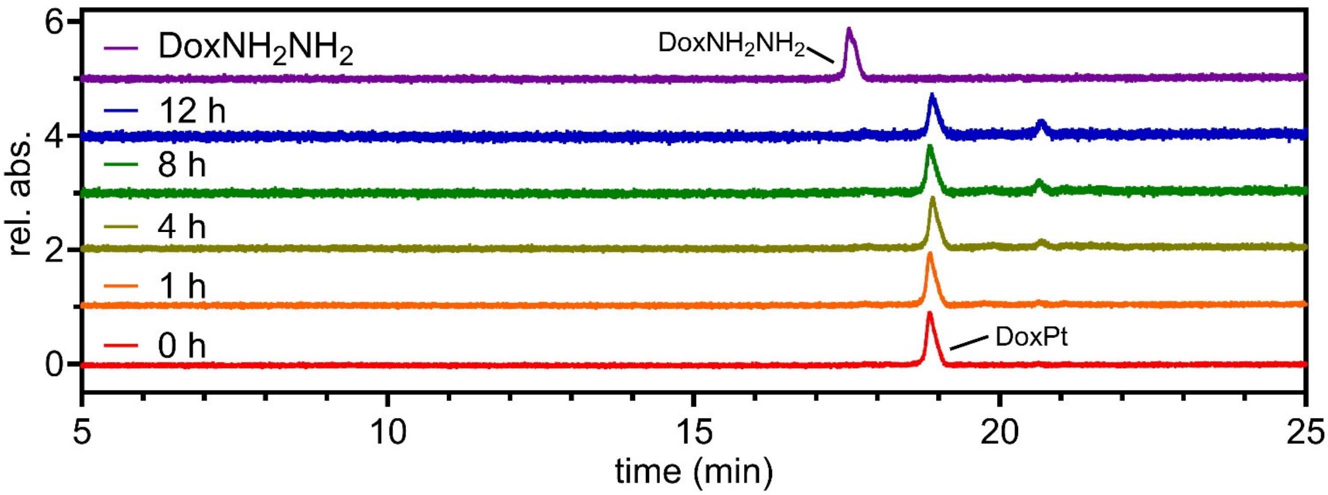

Figure S21. HPLC chromatograms (250 nm) showing the transformation of DoxPt (50 μM) in the presence of (L)-cysteine (100 μM) in pH 7.0 PIPES buffer containing 100 mM KCl and 10 vol % MeOH at 37 °C. Solvent gradient is in the text.

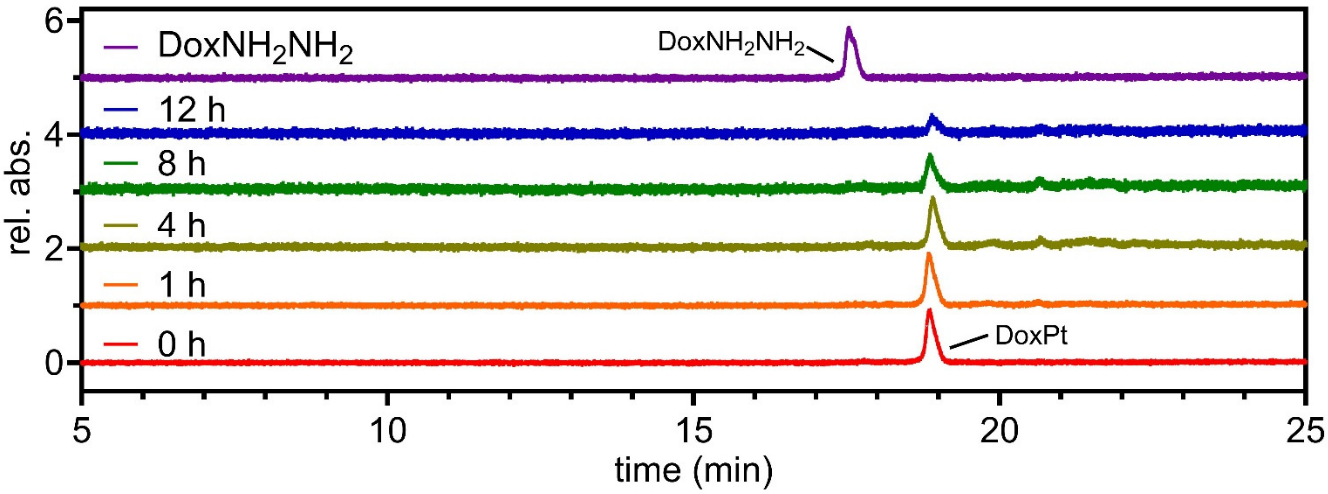

Figure S22. HPLC chromatograms (250 nm) showing the transformation of DoxPt (50 μM) in the presence of (L)-cysteine (200 μM) in pH 7.0 PIPES buffer containing 100 mM KCl and 10 vol % MeOH at 37 °C. Solvent gradient is in the text.

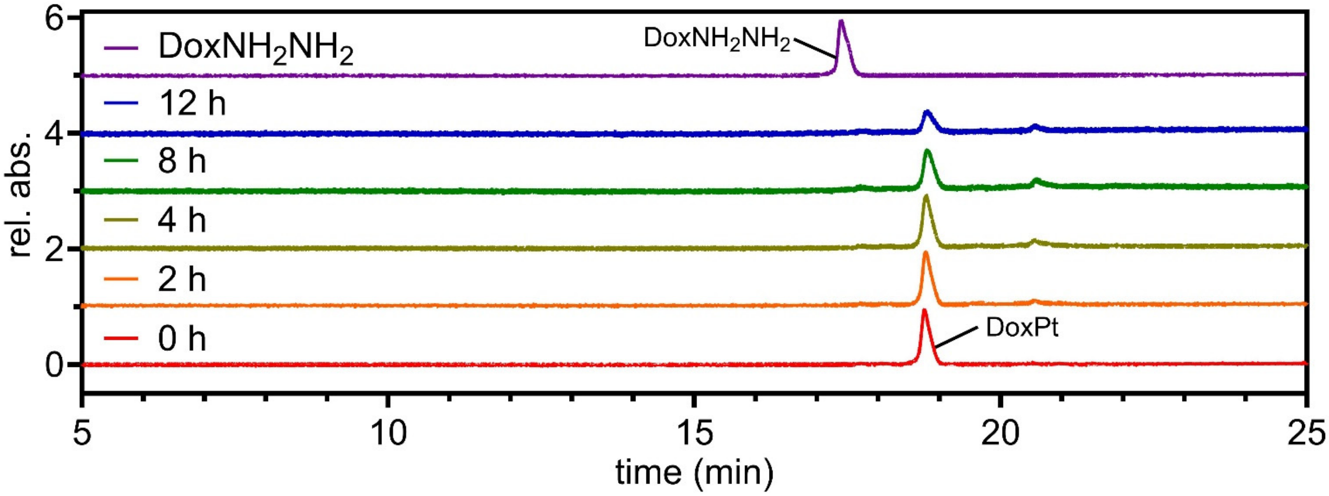

Figure S23. HPLC chromatograms (250 nm) showing the transformation of DoxPt (50 μM) in the presence of GSH (200 μM) in pH 7.0 PIPES buffer containing 100 mM KCl and 10 vol % MeOH at 37 °C. Solvent gradient is given in the text.

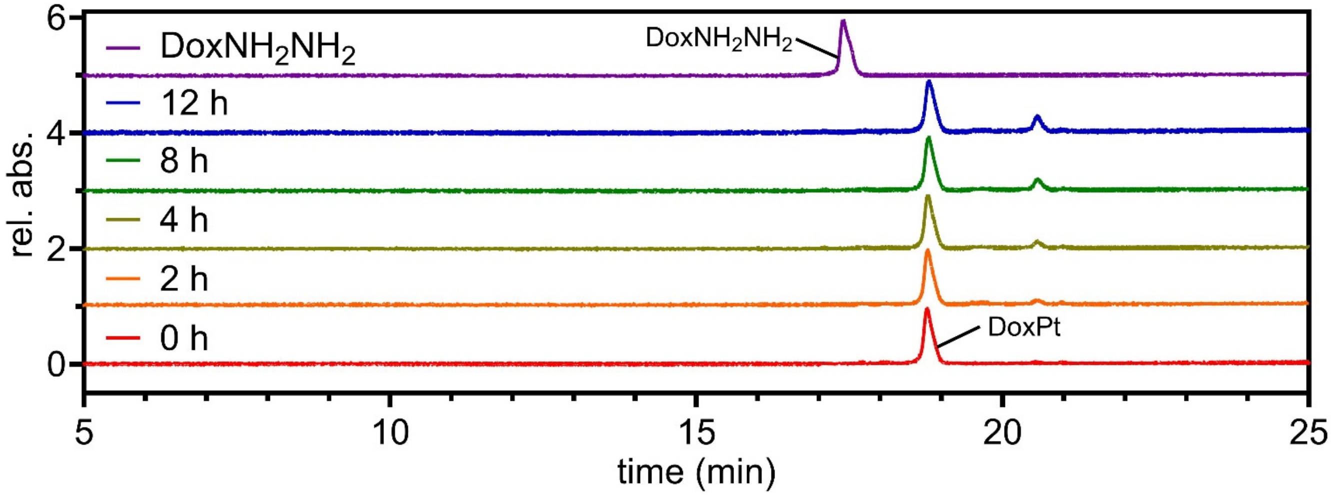

Figure S24. HPLC chromatograms (250 nm) showing the transformation of DoxPt (50 μM) in the presence of GSSG (200 μM) in pH 7.0 PIPES buffer containing 100 mM KCl and 10 vol % MeOH at 37 °C. Solvent gradient is given in the text.

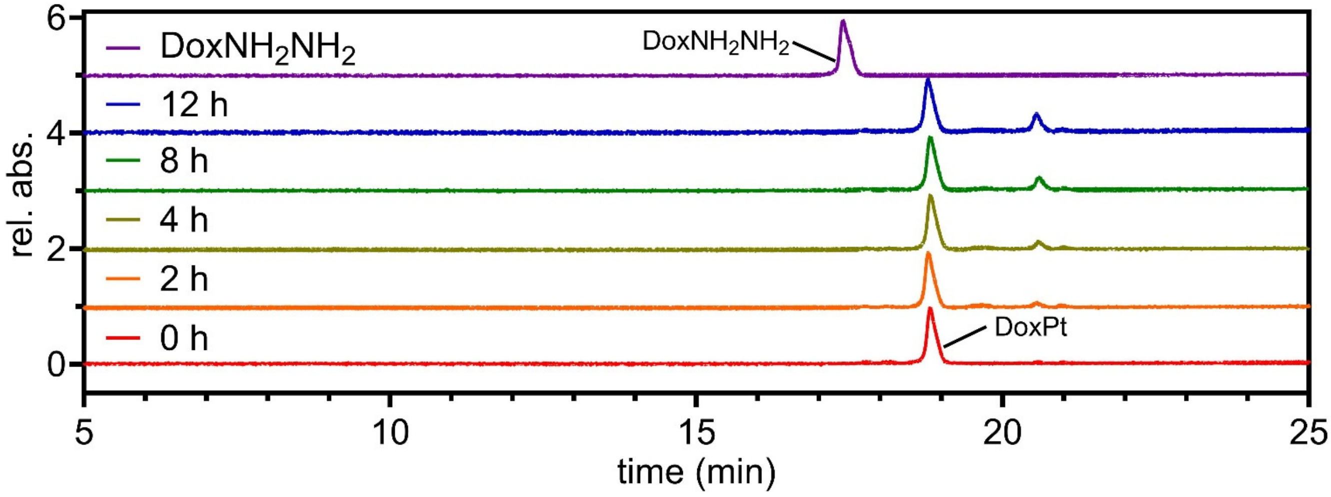

Figure S25. HPLC chromatograms (250 nm) showing the transformation of DoxPt (50 μM) in the presence of (L)-cystine (200 μM) in pH 7.0 PIPES buffer containing 100 mM KCl and 10 vol % MeOH at 37 °C. Solvent gradient is given in text.

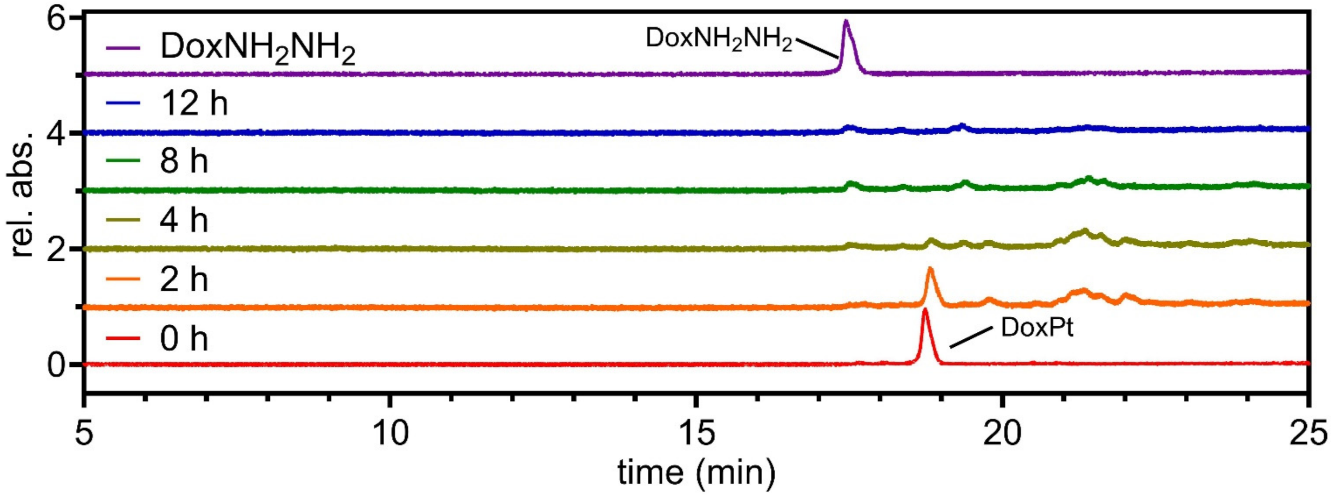

Figure S26. HPLC chromatograms (250 nm) showing the transformation of DoxPt (50 μM) in the presence of (L)-cysteine (1.00 mM) in pH 7.0 PIPES buffer containing 100 mM KCl and 10 vol % MeOH at 37 °C. Fraction area integration indicates about 6% $DoxNH_2NH_2$ was released from DoxPt after 12 h. Solvent gradient is in the text.

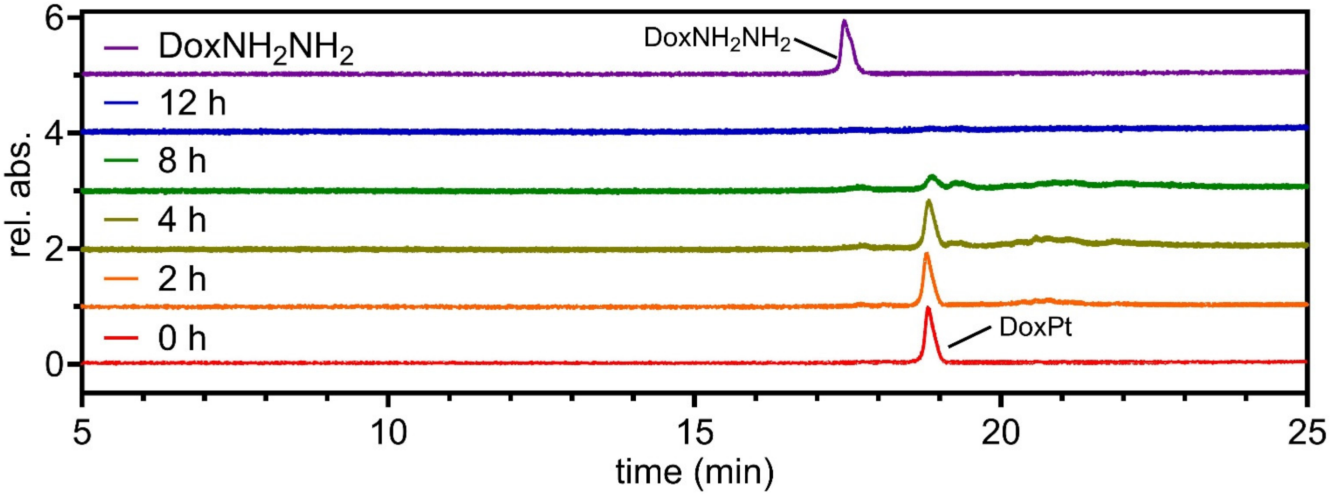

Figure S27. HPLC chromatograms (250 nm) showing the transformation of DoxPt (50 μM) in the presence of GSH (1.00 mM) in pH 7.0 PIPES buffer containing 100 mM KCl and 10 vol % MeOH at 37 °C. Solvent gradient is given in the text.

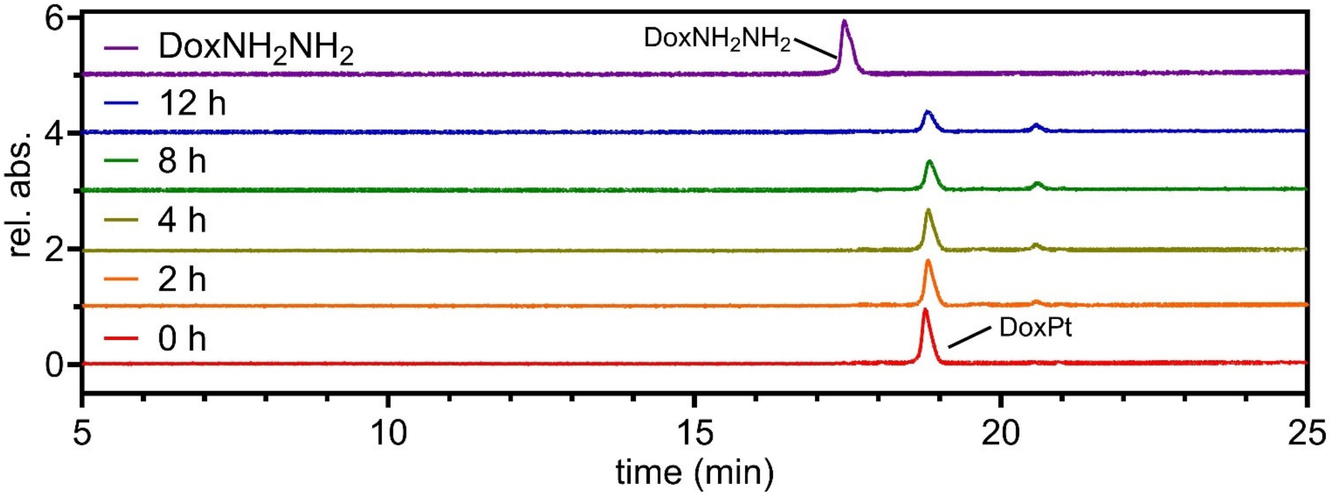

Figure S28. HPLC chromatograms (250 nm) showing the transformation of DoxPt (50 μM) in the presence of GSSG (1.00 mM) in pH 7.0 PIPES buffer containing 100 mM KCl and 10 vol % MeOH at 37 °C. Solvent gradient is given in the text.

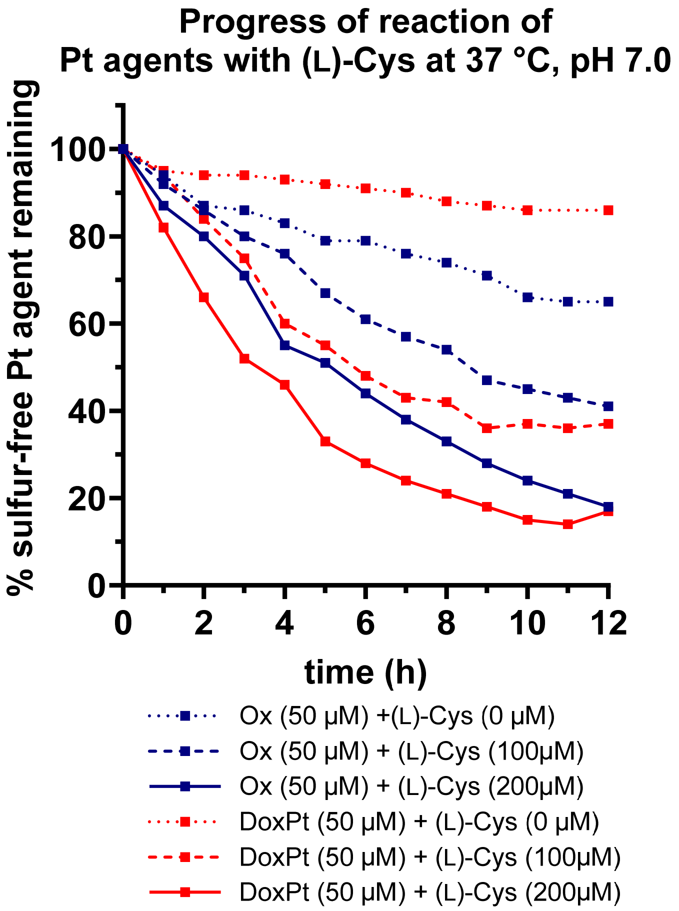

Figure S29. The progress of the reaction of DoxPt and oxaliplatin with 0 µM, 100 µM, and 200 µM (L)-cysteine. Solvent gradients are in the text.

S5. Reactivity of DoxPt4 and DoxPt5 with different sulfur-containing small molecules.

To study the strength of covalent binding between the doxorubicin backbone and the platinum pharmacophore, we monitored the reaction between DoxPt5 and sulfur-containing species at biological or physiological concentrations (200 μM and 1.0 mM) by HPLC. We used a water/CH_3CN/0.1% v/v CF_3CO_2H mobile phase, in which doxorubicin is better separated. We tested reactions with various sulfur-containing species, including GSH, GSSG, (L)-cystine, and (L)-cysteine, which are commonly found in cell culture media, in the cell, or as model thiol-containing biomolecules.

Experimentally, for the reaction with GSH, GSSG, or (L)-cysteine at 200 μM, an aliquot of DoxPt5 in water (2.00 mM, 6.25 μL) and an aqueous solution of sulfur-containing compounds (2.00 mM, 6.25 μL) were added to pH 7.0, 50 mM PIPES buffer (237.5 μL) containing 100 mM KCl. For the reaction with (L)-cystine at 200 μM, DoxPt5 in water (2.00 mM, 6.25 μL) and a solution of (L)-cystine in pH 7.0 PIPES buffer with 100 mM KCl (400 μM, 125 μL) were added to pH 7.0, 50 mM PIPES buffer (118.8 μL) containing 100 mM KCl. For the reaction with GSH, GSSG, or (L)-cysteine at 1.00 mM, DoxPt5 in water (2.00 mM, 6.25 μL) and an aqueous solution of sulfur-containing compounds (200 mM, 1.25 μL) were added to pH 7.0, 50 mM PIPES buffer (242.5 μL) containing 100 mM KCl. The reaction mixture was heated at 37 °C in the dark.

The reaction of DoxPt4 (50 μM) with GSH (1.00 mM) was conducted similarly. The progress of the reaction was monitored by analytical HPLC using a (A) water (0.1% v/v CF_3CO_2H) / (B) CH_3CN (0.1% v/v CF_3CO_2H) solvent system, according to the following protocol: constant flow rate 1.0 $mL \cdot min^{-1}$; 0.0-3.0 min, 10% B; 3.0-23.0 min, linear gradient 10-60% B; 23.0-25.0 min, linear gradient 60-100% B; 25.0-26.0 min, 100% B; 26.0-28.0 min, 10% B; 28.0-30.0 min, 0% B.

As shown in Figure S30, DoxPt5 underwent relatively rapid aquation in pH 7.0 PIPES. In the presence of 200 μM GSH or (L)-cysteine, a few new species, along with a trace amount of doxorubicin, formed after 12 h (Figure S32 and Figure S33). When 1.00 mM thiol-containing compounds, such as GSH and (L)-cysteine, were applied, the reaction with DoxPt5 resulted in more species and led to increased dissociation of doxorubicin from platinum, even though some intact DoxPt4 remained. In contrast, no significant reaction was observed between DoxPt4 or DoxPt5 with disulfide-containing compounds. Overall, these results indicate that both DoxPt4 and DoxPt5 are generally stable in cell culture media. Once entering the cell, these platinum agents can bind nucleophilic biomolecules and potentially release doxorubicin at slow rates.

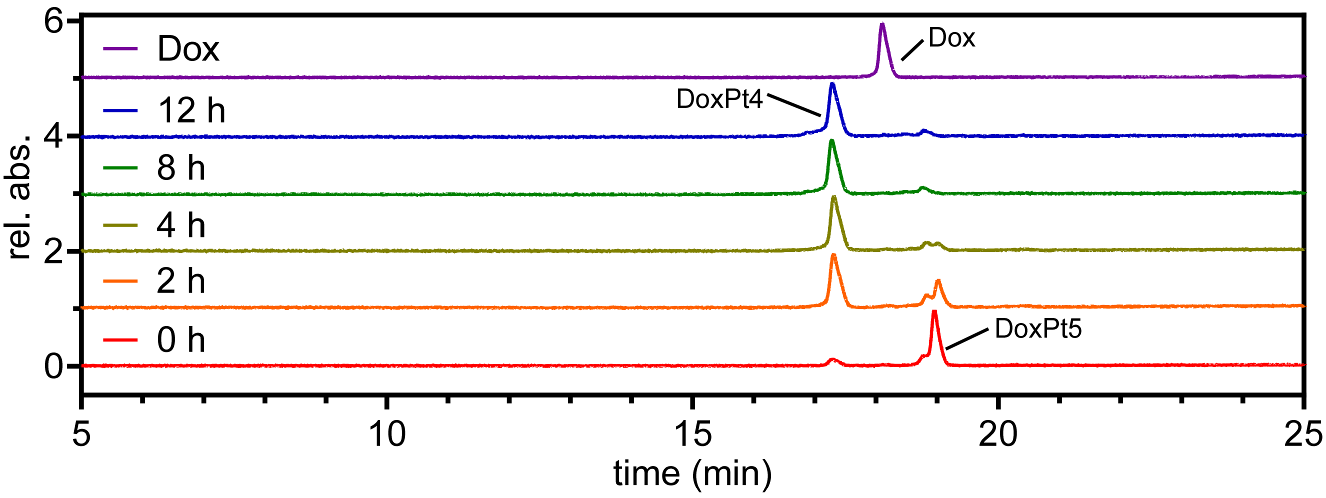

Figure S30. HPLC chromatograms (250 nm) showing the transformation of DoxPt5 (50 μM) in pH 7.0 PIPES buffer containing 100 mM KCl at 37 °C. Solvent gradient is given in the text.

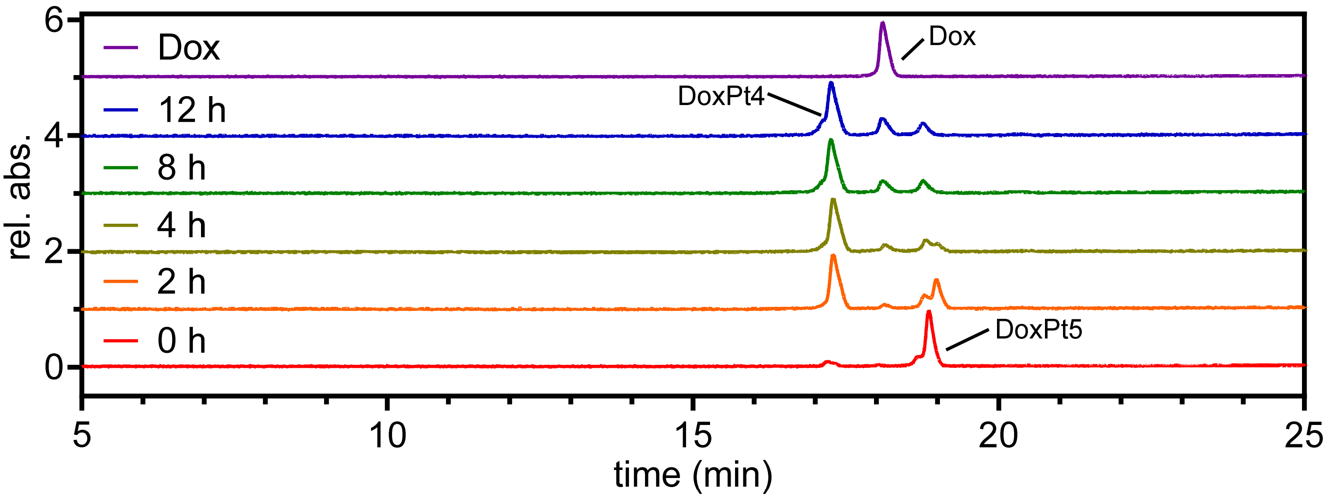

Figure S31. HPLC chromatograms (250 nm) showing the transformation of DoxPt4 (50 μM) in the presence of (L)-cysteine (200 μM) in pH 7.0 PIPES buffer containing 100 mM KCl at 37 °C. Solvent gradient is given in the text.

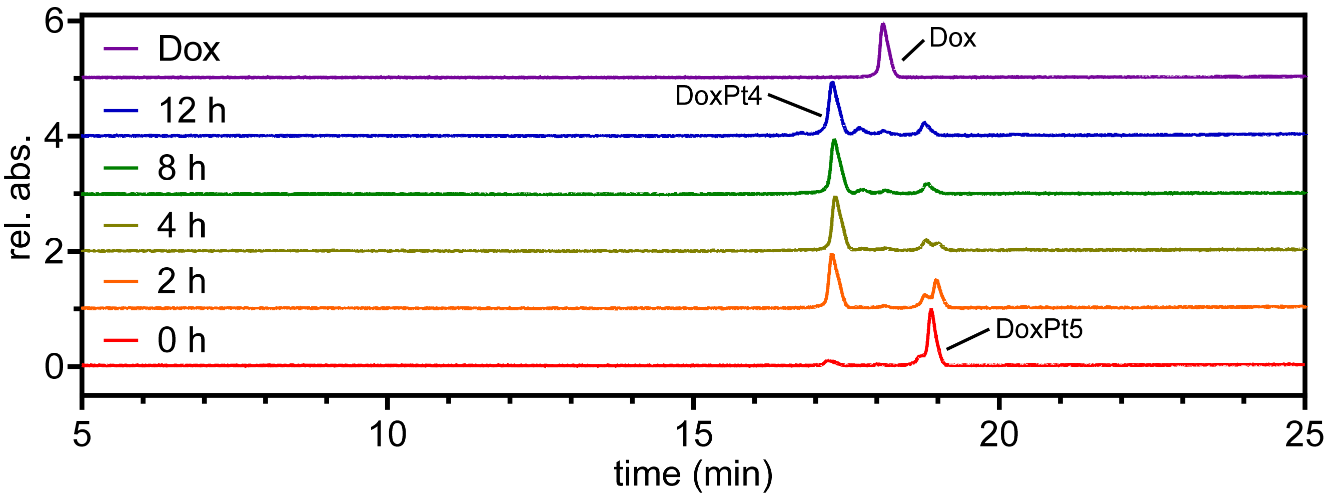

Figure S32. HPLC chromatograms (250 nm) showing the transformation of DoxPt5 (50 μM) in the presence of GSH (200 μM) in pH 7.0 PIPES buffer containing 100 mM KCl at 37 °C. Solvent gradient is given in the text.

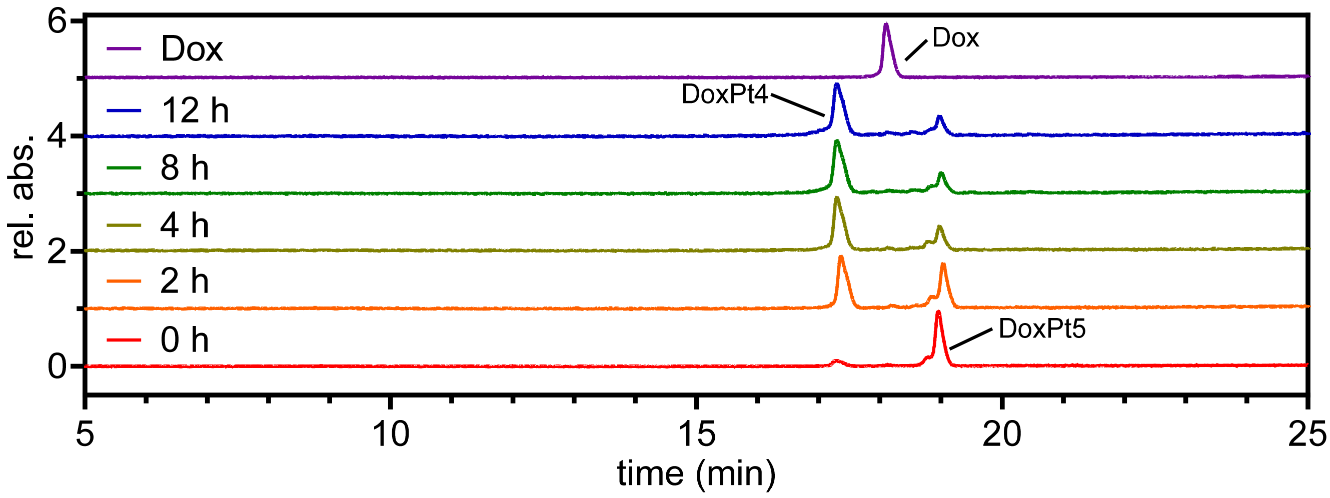

Figure S33. HPLC chromatograms (250 nm) showing the transformation of DoxPt5 (50 µM) in the presence of (L)-cystine (200 µM) in pH 7.0 PIPES buffer containing 100 mM KCl at 37 °C. Solvent gradient is given in the text.

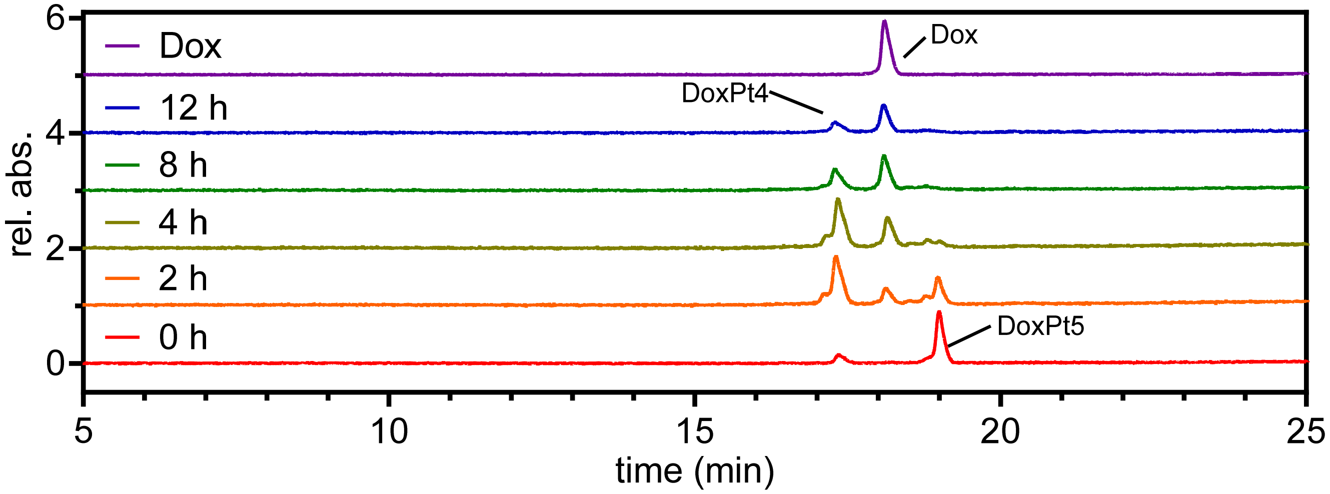

Figure S34. HPLC chromatograms (250 nm) showing the transformation of DoxPt5 (50 µM) in the presence of (L)-cysteine (1.00 mM) in pH 7.0 PIPES buffer containing 100 mM KCl at 37 °C. Solvent gradient is given in the text.

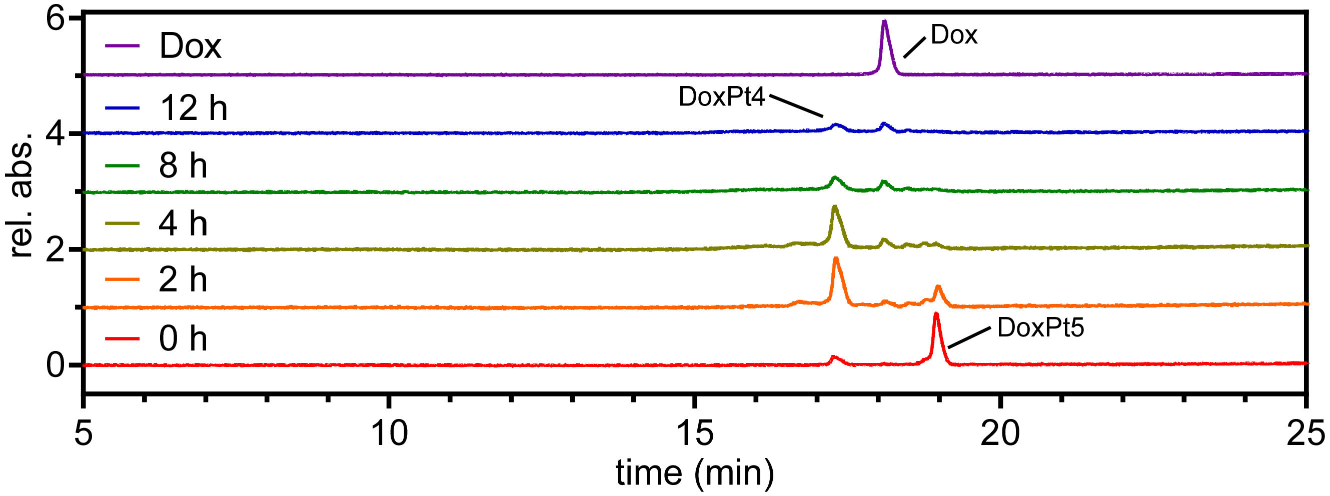

Figure S35. HPLC chromatograms (250 nm) showing the transformation of DoxPt5 (50 µM) in the presence of GSH (1.00 mM) in pH 7.0 PIPES buffer containing 100 mM KCl at 37 °C. Solvent gradient is given in the text.

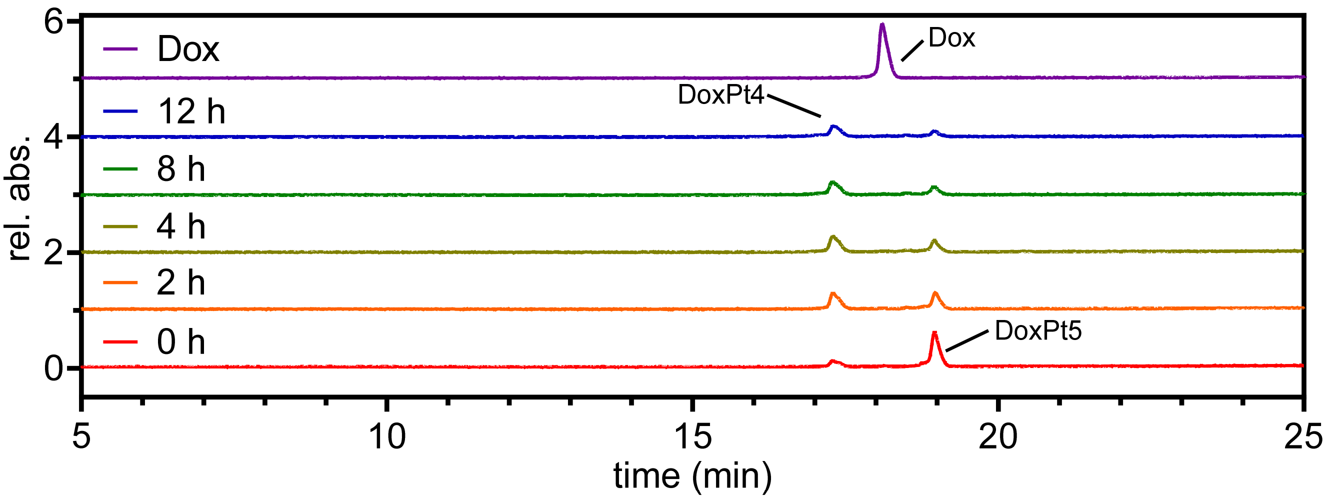

Figure S36. HPLC chromatograms (250 nm) showing the transformation of DoxPt5 (50 μM) in the presence of GSSG (1.00 mM) in pH 7.0 PIPES buffer containing 100 mM KCl at 37 °C. Solvent gradient is given in the text.

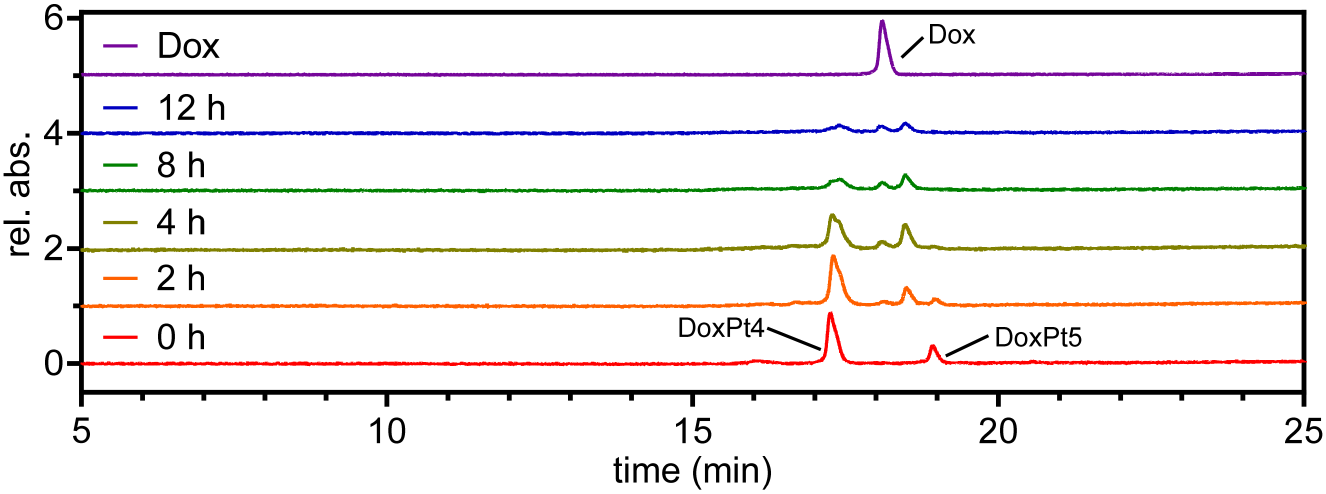

Figure S37. HPLC chromatograms (250 nm) showing the transformation of DoxPt4 (50 μM) in the presence of GSH (1.00 mM) in pH 7.0 PIPES buffer containing 100 mM KCl at 37 °C. Solvent gradient is given in the text.

S6. Reactivity of DoxPt with proteins.

Binding of DoxPt with bovine serum albumin (BSA) as a model protein

The reaction between the model protein BSA and different anticancer agents was studied by gel electrophreosis. The samples were prepared by mixing an aliquot of a BSA stock solution (1.0 mM) with an aliquot of indicated anticancer agents (10 mM for Dox, Ox, and $DoxNH_2NH_2$ in water; 1.16 mM for DoxPt in glycerol:water, 50:50, v/v) in PIPES (pH 7.0, 50 mM) containing 100 mM KCl. The final concentrations of BSA and anticancer agents are as indicated. The reaction mixtures were incubated in the dark at 37 °C for 24 h with shaking at 250 rpm. Upon completion, an aliquot of each reaction mixture (5 µL) was combined with an equal volume of 2× Laemmli sample buffer (Bio-Rad catalog # 1610737), which was loaded directly to the gel without heating. The ladder standard (Precision Plus Protein™ Kaleidoscope™ Prestained Protein Standards, Bio-Rad catalog # 1610375) was also loaded to the gel. After electrophoresis, the gel was visualized on a Bio-Rad GelDoc Go Gel Imaging System using the SYBR® Green settings (Figure S38). The gel was then stained with Coomassie blue and imaged (Figure S39).

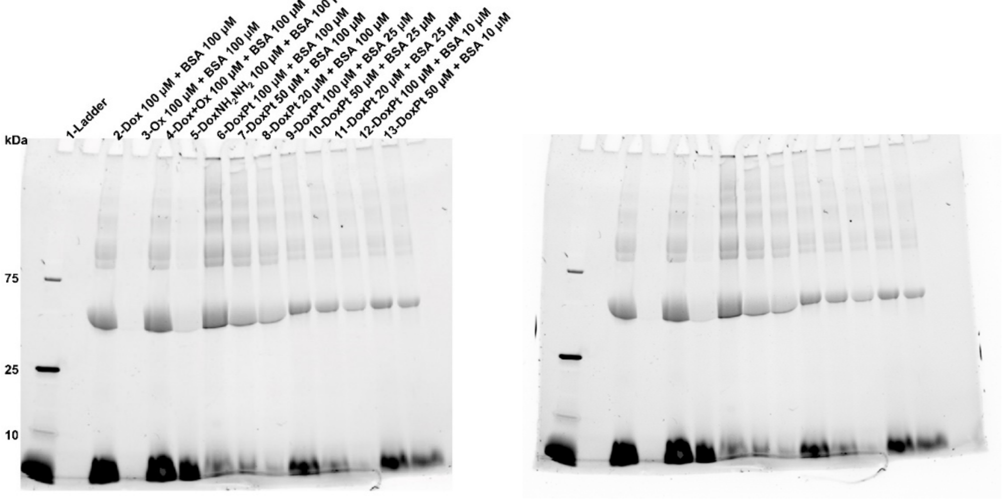

Figure S38. Fluorescence gel image of BSA treated with indicated compounds.

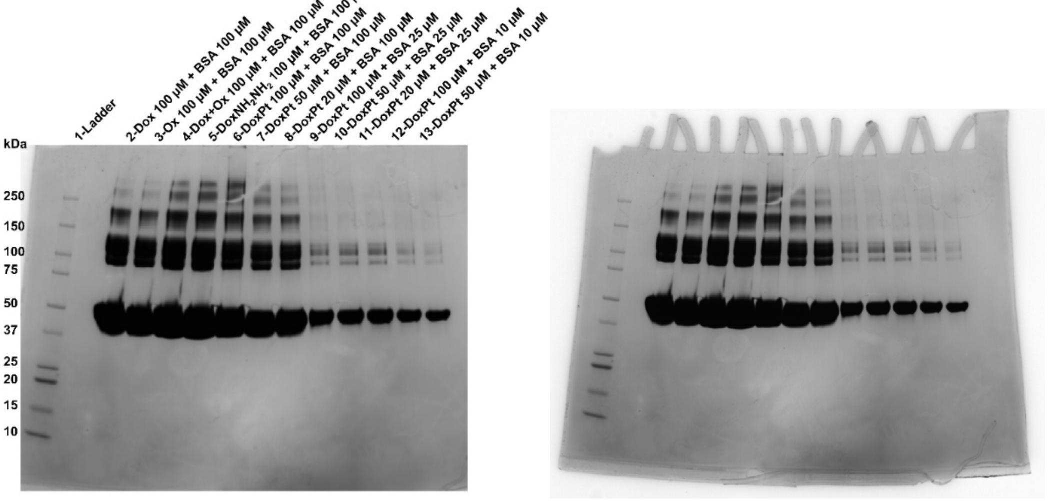

Figure S39. Coomassie-stained gel image of BSA treated with indicated compounds.

Binding of DoxPt with cell lysate

MES-SA cells were grown in T25 flasks to 100% confluency. The cells were harvested with trypsin and scraping. The suspension of cells from four flasks was combined and centrifuged (200 ×*g*). The supernatant was removed. The cell pellet was suspended in PBS and transferred to an Eppendorf tube, which was centrifuged (13,000 ×*g*). The PBS was removed. The cell pellet was frozen in liquid nitrogen for 5 min and thawed at rt, followed by the addition of MilliQ water (200 µL). The freeze-thaw process was repeated three additional times. This mixture was then centrifuged (13,000 ×*g*) for 15 min. An aliquot of supernatant (25 µL) was mixed with the indicated anticancer agent to reach a final concentration of 500 µM. The reaction mixtures were incubated in the dark at 37 °C for 24 h with shaking at 300 rpm. Upon completion, an aliquot of each reaction mixture (10 µL) was combined with an equal volume of 2× Laemmli sample buffer (Bio-Rad catalog # 1610737), which was loaded directly to the gel without heating. The ladder standard (Precision Plus Protein™ Kaleidoscope™ Prestained Protein Standards, Bio-Rad catalog # 1610375) was also loaded to the gel. After electrophoresis, the gel was visualized on a Bio-Rad GelDoc Go Gel Imaging System using the SYBR® Green settings (Figure S40). The gel was then stained with Coomassie blue and imaged (Figure S41).

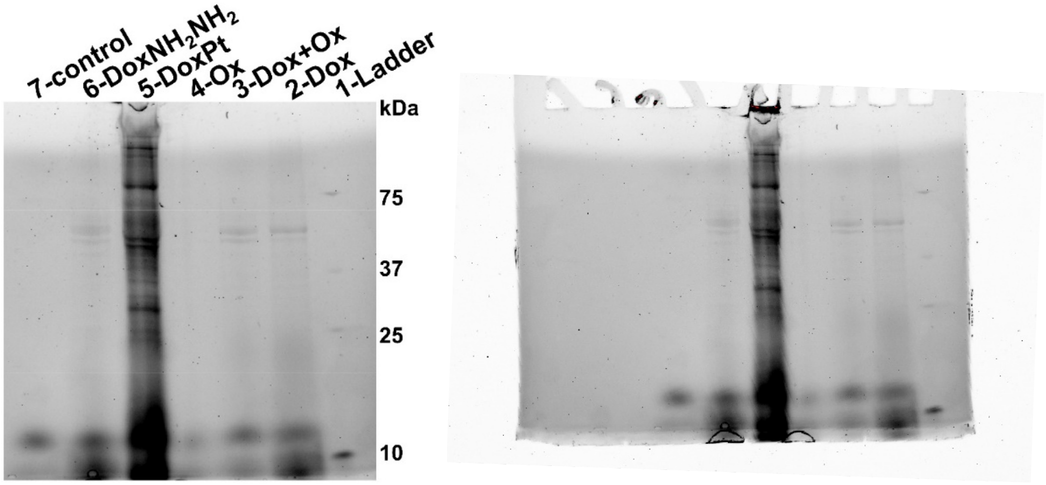

Figure S40. Fluorescence gel image of MES-SA whole-cell lysate treated with indicated compounds.

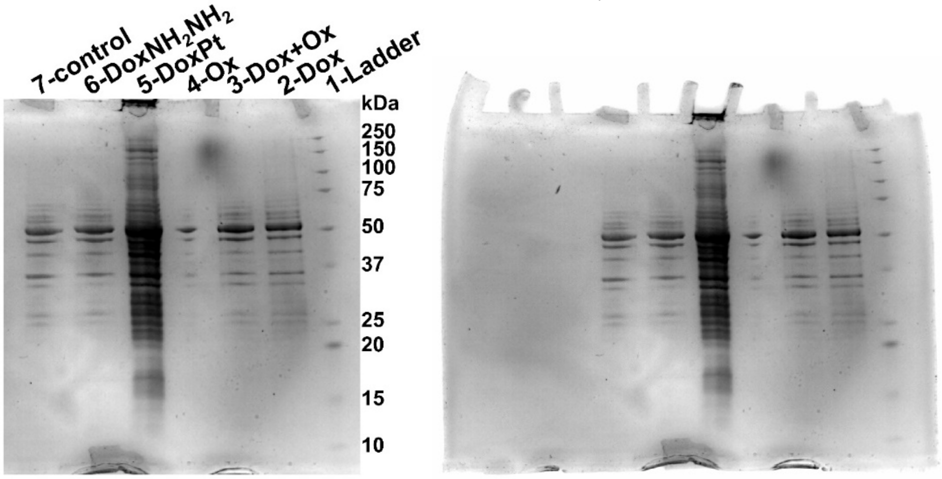

Figure S41. Coomassie-stained gel image of MES-SA whole-cell lysate treated with indicated compounds.

***S5. Intracellular Pt content analysis*.**

Time-dependent platinum clearance.

MES-SA and MES-SA/Dx5 cells were grown in six-well plates in an incubator until reaching a population of about 10^6 cells. The cells were then treated with oxaliplatin (10.0 μM) or doxaliplatin (1.25 μM).

For the t = 0 h time point, cells were treated with the corresponding drug for 12 h. To harvest, the drug-containing media were removed, and the cells were washed twice with PBS containing 100 μM EDTA (2.0 mL). The cells were then lysed in MilliQ water (1.0 mL) for 20 min at rt. A portion of the resulting cell lysates (0.90 mL) was transferred to an Eppendorf tube and stored at −80 °C.

For cells harvested at later time points, drug-containing media were removed after 12 h of treatment. Cells were then washed twice with PBS (2.0 mL) and further incubated in drug-free media. After an additional incubation time (4, 8, and 24 h), the media were removed, and the cells were washed twice with PBS containing 100 μM EDTA (2.0 mL). The cells were then lysed in MilliQ water (1.0 mL) for 20 min at rt. A portion of the resulting cell lysates (0.90 mL) were transferred to an Eppendorf tube and stored at −80 °C.

To ensure complete cell lysis, all samples were thawed at rt and frozen at −80 °C. This procedure was repeated three times. The insoluble portion of the lysate was pelleted by centrifugation (10,000 g for 10 min). The soluble protein concentration was determined by mixing an aliquot of the lysate (14.2 μL) with Pierce™ 660 nm Protein assay reagent (ThermoFisher catalog # 22660, 200 μL) in a 96-well plate.

The samples, including the insoluble lysate, were lyophilized and then treated with conc. HNO_3 (200 μL) at 70 °C for 1 h. The digested samples (180 μL) were then diluted with MilliQ water (320 μL). The platinum concentrations of the resulting samples were determined by atomic absorption spectrometry (AAS). The total platinum content of each sample was normalized to the total mass of soluble protein.

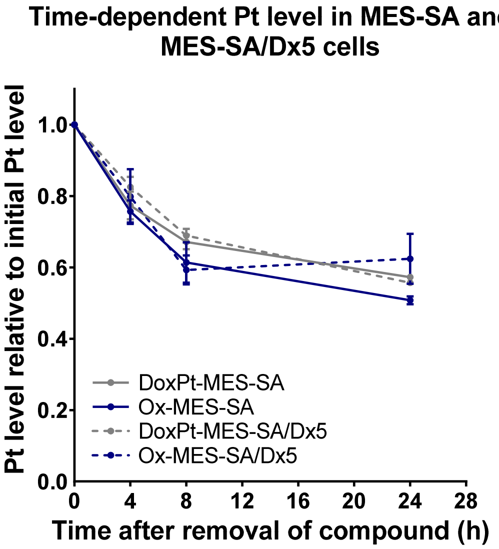

Time after drug removal (h)	Pt level in Ox-treated MES-SA cells (nmol Pt/mg protein)	Pt level in Ox-treated MES-SA/Dx5 cells (nmol Pt/mg protein)	Pt level in DoxPt-treated MES-SA cells (nmol Pt/mg protein)	Pt level in DoxPt-treated MES-SA/Dx5 cells (nmol Pt/mg protein)
0	0.311±0.008	0.217±0.020	0.663±0.041	1.42±0.02
4	0.235±0.009	0.174±0.017	0.514±0.026	1.17±0.04
8	0.190±0.018	0.129±0.009	0.445±0.014	0.977±0.027
24	0.155±0.003	0.133±0.016	0.374±0.002	0.778±0.010

Figure S42. Time-dependent Pt clearance from MES-SA and MES-SA/Dx5 cells treated with oxaliplatin (Ox, 10.0 μM) or doxaliplatin (DoxPt, 1.25 μM). The horizontal axis indicates the time after the cells were switched to drug-free media. The vertical axis indicates the Pt level relative to the initial Pt level under respective conditions. Error bars represent standard deviations.

Effect of verapamil on platinum clearance.

MES-SA and MES-SA/Dx5 cells were grown in six-well plates in an incubator until reaching a population of about 10^6 cells. The cells were treated with oxaliplatin (10.0 μM) or doxaliplatin (1.25 μM) and incubated in the presence or absence of verapamil (10 μM) for 12 h. The drug-containing media were then removed.

For cells harvested immediately after the 12 h drug treatment (Figure S43A), the cells were washed twice with PBS containing 100 μM EDTA (2.0 mL) and lysed in MilliQ water (1.0 mL, 18.2 MΩ cm) for 20 min at rt. A portion of the resulting cell lysate (0.90 mL) was transferred to an Eppendorf tube and stored at −80 °C.

For the second set of samples (Figure S43B), the cells were washed twice with PBS (2.0 mL) and incubated for an additional 24 h in drug-free media. The media were then removed. Cells were washed twice with PBS containing 100 μM EDTA (2.0 mL) and lysed in MilliQ water (1.0 mL) for 20 min at rt. A portion of the resulting cell lysate (0.90 mL) was transferred to an Eppendorf tube and stored at −80 °C.

To ensure complete cell lysis, all samples were thawed at rt and frozen at −80 °C. This procedure was repeated three times. The insoluble lysate was pelleted by centrifugation (10,000 g for 10 min). The soluble protein concentration was determined by mixing an aliquot of the lysate (14.2 μL) with Pierce™ 660 nm Protein assay reagent (ThermoFisher catalog # 22660, 200 μL) in a 96-well plate. These samples, including the insoluble lysate, were lyophilized and then treated with conc. HNO_3 (200 μL) at 70 °C for 1 h. The digested samples (180 μL) were then diluted with MilliQ water (320 μL). The platinum concentrations of the resulting samples were determined by AAS. The total platinum content of each sample was normalized to the total mass of soluble protein.

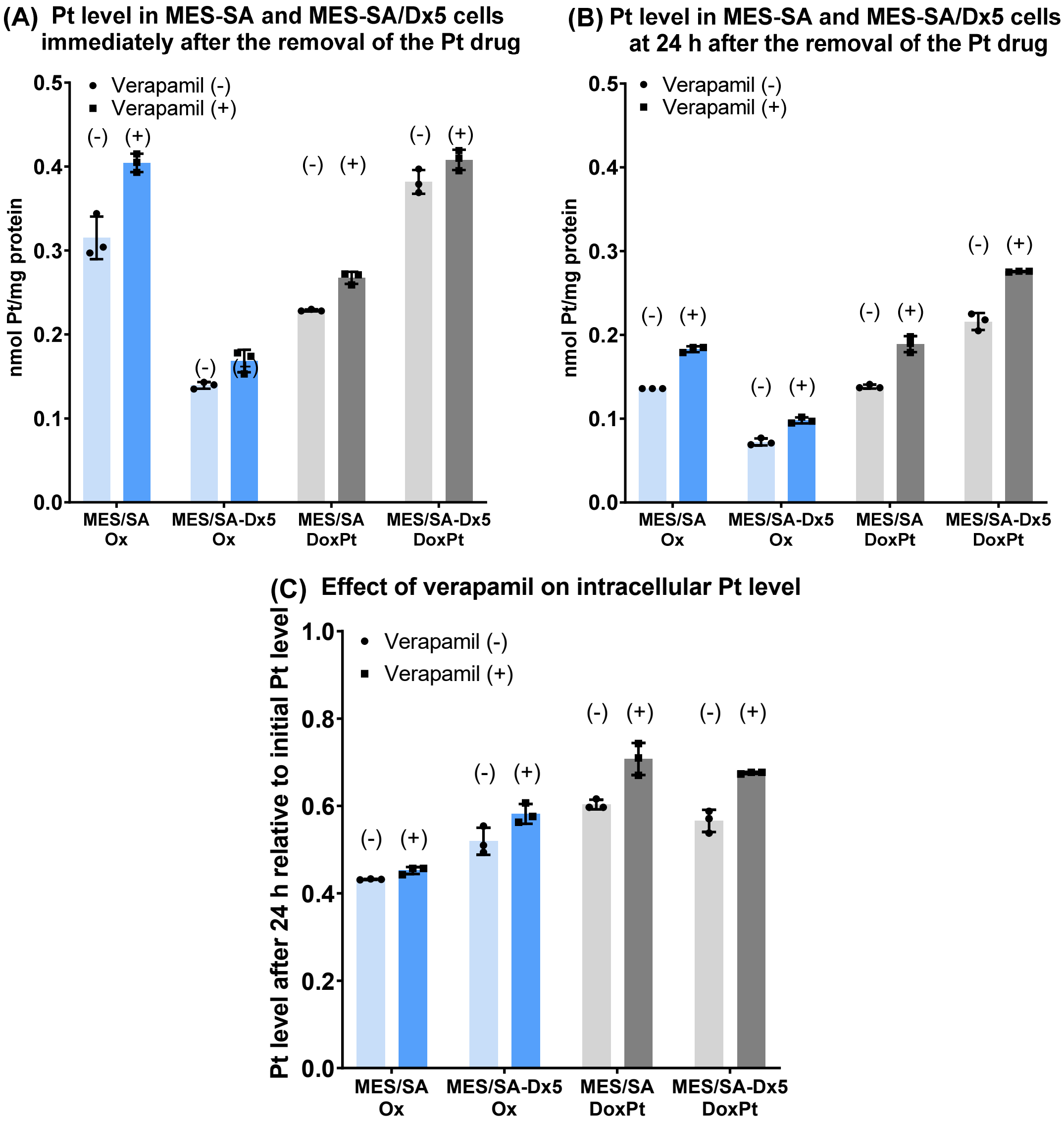

Figure S43. (A) Pt level in MES-SA and MES-SA/Dx5 cells immediately after the removal of the Pt agent. (B) Pt level in MES-SA and MES-SA/Dx5 cells 24 h after the removal of the Pt agent. (C) Comparison of relative Pt levels in MES-SA and MES-SA/Dx5 cells in the presence or in the absence of verapamil 24 h after the removal of the Pt agent. Verapamil has a minimal effect on Pt clearance. Error bars represent standard deviations.

Subcellular platinum distribution.

MES-SA and MES-SA/Dx5 cells were grown in six-well plates until reaching a population of about 10^6 cells. The cells were treated with oxaliplatin (10.0 μM) or DoxPt (1.25 μM) and incubated for 14 h. The drug-containing media were then removed. The cells were washed twice with PBS containing 100 μM EDTA (2.0 mL) and harvested with trypsin-EDTA. The nuclear and cytoplastic contents of these cells were extracted with NE-PER™ nuclear and cytoplasmic extraction reagents (ThermoFisher catalog # 78833) following the manufacturer's instructions. These extracts, along with the insoluble fractions, were lyophilized. The cytoplasmic, nuclear, and insoluble fractions were digested with 1000 μL, 200 μL, and 175 μL of conc. HNO_3 respectively at 70 °C for 1 h. For the cytoplasmic and nuclear fractions, 180 μL of the digested samples was diluted with MilliQ water (320 μL). For the insoluble fractions, 170 μL of the digested sample was mixed with conc. HNO_3 (10 μL) and MilliQ water (320 μL). The platinum concentrations of the resulting samples were determined by AAS.

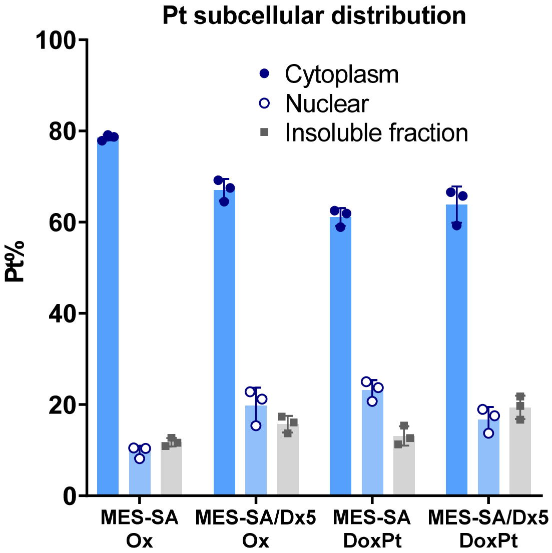

	Pt content in cytoplasmic fraction (ng)	cytoplasmic platinum percentage (%)	Pt content in nuclear fraction (ng)	nuclear platinum percentage (%)	Pt content in insoluble fraction (ng)	Insoluble fraction platinum percentage (%)
Ox-treated MES-SA cells	67.1±5.2	78.6±0.6	8.3±1.8	9.7±1.3	10.0±0.3	11.8±0.9
Ox-treated MES-SA/Dx5 cells	33.3±3.3	67.1±2.4	9.9±2.3	19.8±3.9	6.5±0.7	13.1±2.1
DoxPt-treated MES-SA cells	51.3±7.5	61.1±2.0	19.2±0.6	23.2±2.2	13.2±2.8	15.7±1.9
DoxPt-treated MES-SA/Dx5 cells	59.0±5.7	64.9±4.0	59.0±1.9	16.7±2.7	17.9±2.4	19.4±2.6

Figure S44. Subcellular Pt distribution in MES-SA and MES-SA/Dx5 cells treated with oxaliplatin (Ox, 10.0 μM) or DoxPt (1.25 μM). The absolute Pt content value in each fraction is shown in the table. Error bars represent standard deviations.

Platinum distribution on nucleic acids and proteins.

(a) Whole-cell Pt accumulation measurement.

MES-SA cells were grown in six-well plates until reaching a population of about 3×10^6 cells per well. The cells were treated with oxaliplatin (25.0 μM) or DoxPt (5.00 μM) for 18 h. The drug-containing media were then removed. The cells were washed twice with PBS containing 100 μM EDTA (2.0 mL) and lysed in MilliQ water (1.0 mL) for 20 min at rt. A portion of the resulting cell lysate (0.90 mL) was transferred to an Eppendorf tube and lyophilized. The resulting material was treated with conc. HNO_3 (200 μL) at 70 °C for 1 h. The digested sample (90 μL) was then mixed with conc. HNO_3 (10 μL) and MilliQ water (400 μL). The platinum concentrations of the resulting samples were determined by AAS.

To confirm the viability of cells in the presence of anticancer agents at high concentrations, we treated MES-SA and MES-SA/Dx5 cells with oxaliplatin (25.0 μM) or DoxPt (5.00 μM) for 18 h. Our experiments indicate that >75% of cells were viable under these conditions (Figure S46).

(b) Determination of platinum content on nucleic acids.

MES-SA cells were grown in six-well plates until reaching a population of about 3×10^6 cells per well. For each sample, cells from five wells were pooled (about 1.5×10^7 cells total). The cells were treated with oxaliplatin (25.0 μM) or DoxPt (5.00 μM) for 18 h. The drug-containing media were then removed. Cells were washed twice with PBS containing 100 μM EDTA (2.0 mL). Cells were harvested with TrypLE™ Express (Gibco™ catalog # 12604013, 1.0 mL) for 5 min at 37 °C. The resulting cell suspension (0.90 mL each) was pooled from five wells and collected by centrifugation. The cell pellet was then suspended in PBS (50 μL) followed by the addition of 0.3 M sodium acetate aq. solution (150 μL). A mixture of phenol:chloroform:isoamyl alcohol (25:24:1 *v/v*, ThermoFisher catalog # 15593031, 200 μL) was then added. The mixture was shaken vigorously and centrifuged at 16,000 ×*g* for 5 min at rt. The top nucleic acid-containing aqueous layer (ca. 150 μL) was carefully removed and transferred to an Eppendorf tube. A portion of 100% EtOH (450 μL) was added to the Eppendorf tube to precipitate the nucleic acid. The tube was inverted gently and stored at −80 °C for 3 min. The tube was then centrifuged at 16,000 ×*g* for 5 min at rt. The supernatant was removed. The nucleic acid pellet was suspended in ice-cold 75% EtOH (1.0 mL) and centrifuged at 16,000 ×*g* for 5 min at rt. The supernatant was removed. The remaining pellet was dried under air before lyophilization. The resulting material was treated with conc. HNO_3 (200 μL) at 70 °C for 1 h. The digested sample (90 μL) was then mixed with conc. HNO_3 (10 μL) and MilliQ water (400 μL). The platinum content of the resulting samples was determined by AAS. The percentage platinum content on nucleic acids was calculated by dividing the nucleic acid platinum content by the total platinum content determined in the whole cell platinum accumulation experiment.

(c) Determination of platinum content on proteins.

MES-SA cells were grown in six-well plates until reaching a population of about 3×10^6 cells. The cells were treated with oxaliplatin (25.0 μM) or DoxPt (5.00 μM) for 18 h. The drug-containing media were then removed. The cells were washed twice with PBS containing 100 μM EDTA (2.0 mL) and lysed in MilliQ water (1.0 mL)

for 20 min at rt. A portion of the resulting cell lysate (0.90 mL) was transferred to an Eppendorf tube. The lysate was frozen at −80 °C and thawed at rt. This procedure was repeated three times. Trichloroacetic acid (Sigma-Aldrich catalog # T0699, 100 µL) was added to the tube. The tube was vortexed and then placed on ice for 30 min to allow for protein precipitation. The mixture was centrifuged at 10,000 ×*g* for 10 min at 4 °C. The supernatant was removed. The pellet was washed with ice-cold acetone (0.5 mL) to remove residual trichloroacetic acid. The mixture was centrifuged at 10,000 ×*g* for 5 min at 4 °C. This procedure was repeated one more time. The resulting pellet was dried under air before lyophilization. The resulting material was treated with conc. HNO_3 (200 µL) at 70 °C for 1 h. The digested sample (90 µL) was then mixed with conc. HNO_3 (10 µL) and MilliQ water (400 µL). The platinum concentrations of the resulting samples were determined by AAS. The percentage platinum content on protein was calculated by dividing the protein platinum content by the total platinum content determined in the whole cell platinum accumulation experiment.

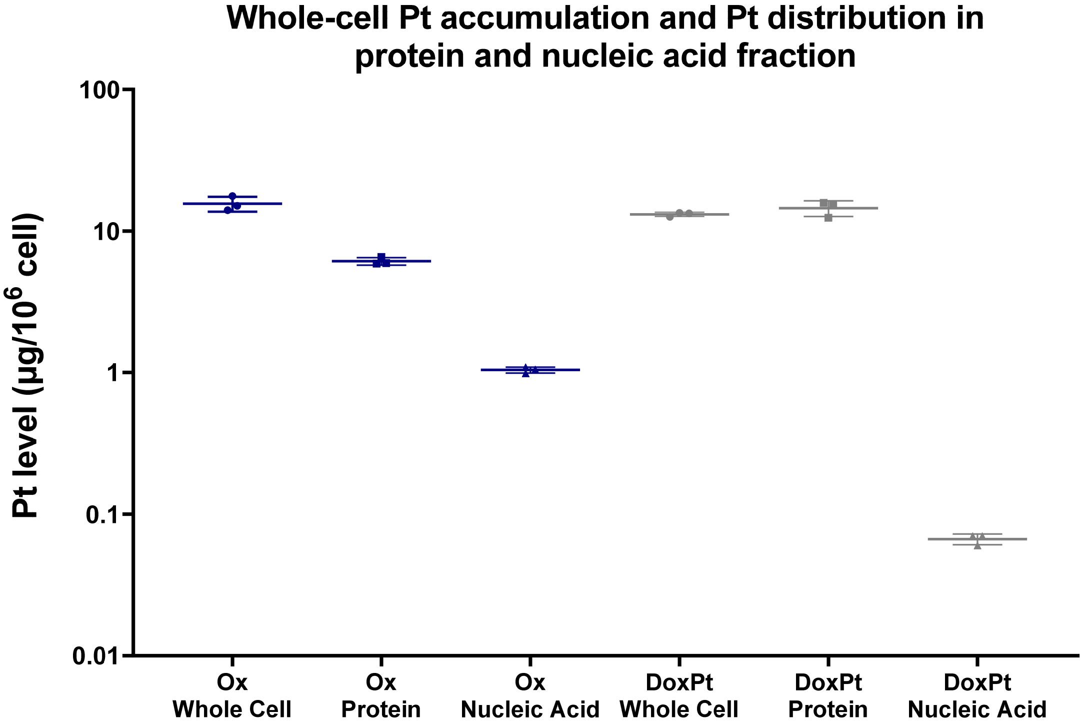

Pt agent	Pt content in whole cell (µg/10^6 cells)	Pt content in nucleic acid fraction (µg/10^6 cells)	Pt content in protein fraction (µg/10^6 cells)
Ox	15.6±1.9	1.04±0.05	6.12±0.37
DoxPt	13.1±0.5	0.065±0.003	14.5±1.8

Figure S45. Pt distribution on nucleic acid and protein in MES-SA cells treated with oxaliplatin (25.0 µM) or DoxPt (5.00 µM). The absolute Pt content value in each fraction is shown in the table. Error bars represent standard deviations.

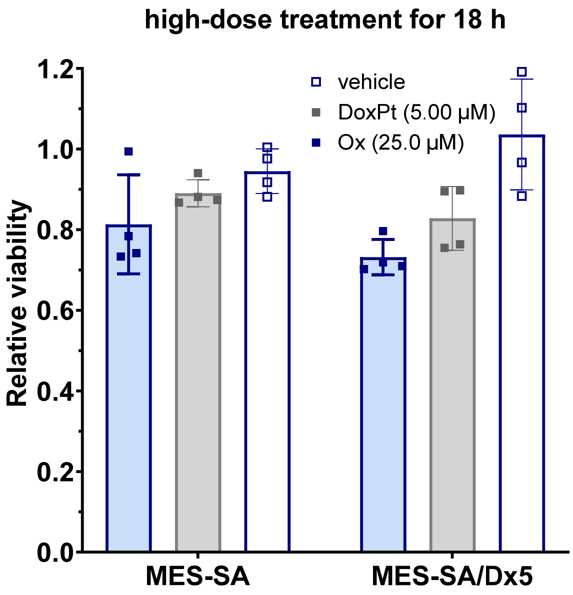

Figure S46. Relative cell viability of MES-SA and MES-SA/Dx5 cells treated with oxaliplatin (25.0 μM) or DoxPt (5.00 μM) for 18 h. Error bars represent standard deviations.

S6. In vitro toxicity determination with conventional cancer cell lines.

Typical procedure

Cells were grown in media (10 mL) in 10 cm tissue culture plates. The cells were trypsinized and seeded into 96-well plates with 5×10^3 cells per well. Cells were treated with cytotoxic drug-containing media (200 µL) at the time of plating. For experiments with P-gp inhibition, verapamil was applied at the indicated concentration at the time of cell seeding along with the cytotoxic agent. The cells were then incubated for 72 h following the drug treatment. Cell growth was measured by a resazurin assay. In brief, at the end of 72 h drug treatment, resazurin was added to the cells at a final concentration of 50 µg/mL. The baseline fluorescence was determined immediately. The cells were then incubated at 37 °C for 2 h. The fluorescence of the resorufin product was measured at 560/590 nm and baseline corrected. The cell growth at each drug concentration was normalized to the untreated control.

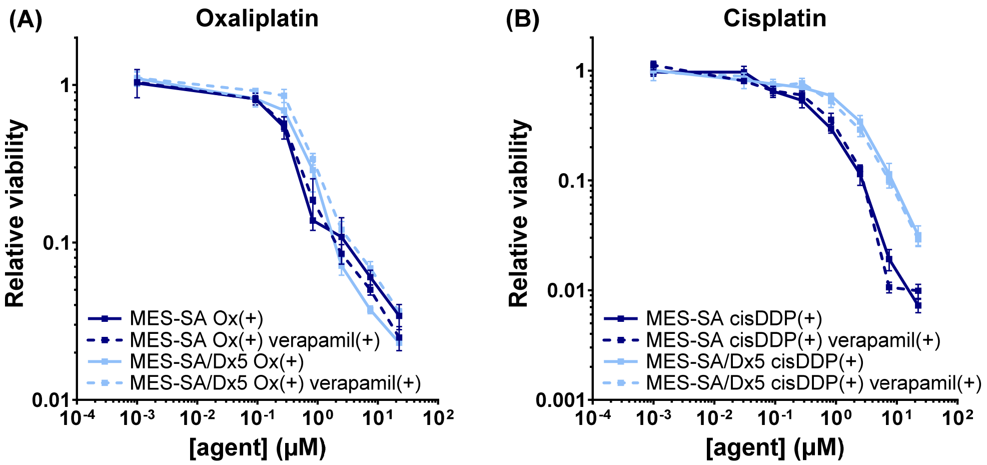

IC_{50} (µM)	MES-SA	MES-SA/Dx5	Fold Resistance
cisDDP	0.29±0.05	1.3±0.1	4.6
Ox	0.29±0.02	0.53±0.07	1.8

IC_{50} (µM) with MES-SA	(−) verapamil	(+) verapamil (10 µM)	Fold Sensitization
cisDDP	0.29±0.05	0.45±0.06	0.65
Ox	0.29±0.02	0.33±0.05	0.86

IC_{50} (µM) with MES-SA/Dx5	(−) verapamil	(+) verapamil (10 µM)	Fold Sensitization
cisDDP	1.3±0.1	1.0±0.1	1.3
Ox	0.53±0.07	0.62±0.04	0.85

Figure S47. Dose-response curves showing the effect of oxaliplatin (Ox) and cisplatin (cisDDP) on the viability of MES-SA human uterine sarcoma cell line (A, dark blue) and MES-SA/Dx5 doxorubicin-resistant human uterine sarcoma cell line (B, light blue) after a 72-hour exposure. The solid line indicates the effect of drugs in the absence of P-gp inhibitor verapamil. The dashed line indicates the effect of drug in the presence of verapamil. Error bars represent standard deviations.

Table S1. Summary of IC_{80} and IC_{50} values of anticancer agents against various human cancer cell lines.

IC_{80} (µM)	Origin	Dox	DoxPt	Ox
HCT116	Colon	0.087±0.003	0.54±0.06	0.96±0.11
HCT15	Colon	0.23±0.04	0.22±0.06	3.9±0.6
HT29	Colon	0.41±0.06	0.65±0.15	1.8±0.6
COLO205	Colon	0.049±0.005	0.71±0.10	3.3±1.5
A549	Lung	0.39±0.13	1.6±0.5	10±7
H1299	Lung	0.22±0.01	0.39±0.04	3.7±1.1
HS 578T	Breast	0.51±0.11	0.61±0.08	>67.5
MDA-MB-231	Breast	0.21±0.02	0.71±0.06	21±11
BT549	Breast	0.29±0.00	0.76±0.02	32±4
T-47D	Breast	0.48±0.08	1.3±0.2	53±9
SKOV3	Ovarian	0.29±0.18	0.85±0.07	66±4
OVCAR3	Ovarian	0.072±0.005	0.46±0.18	1.1±0.2
OVCAR4	Ovarian	0.53±0.06	1.1±0.1	19±6
OVCAR8	Ovarian	0.18±0.02	0.97±0.14	33±21
LNCAP	Prostate	0.092±0.008	0.23±0.05	1.3±0.4
22RV1	Prostate	0.13±0.01	0.39±0.06	15±11
DU 145	Prostate	0.093±0.005	0.27±0.05	6.5±2.1
PC3	Prostate	0.28±0.03	0.76±0.05	27±7
LOX IMVI	Melanoma	0.15±0.02	0.14±0.03	3.2±0.7

IC_{50} (µM)	Origin	Dox	DoxPt	Ox
HCT116	Colon	0.022±0.001	0.22±0.04	0.36±0.04
HCT15	Colon	0.029±0.001	0.067±0.019	0.85±0.09
HT29	Colon	0.036±0.007	0.14±0.03	0.32±0.06
COLO205	Colon	0.019±0.001	0.24±0.09	0.54±0.09
A549	Lung	0.0093±0.0003	0.16±0.03	0.63±0.05
H1299	Lung	0.12±0.04	0.095±0.022	0.66±0.14
HS 578T	Breast	0.0060±0.0006	0.14±0.06	2.0±0.6
MDA-MB-231	Breast	0.021±0.007	0.11±0.03	0.64±0.32
BT549	Breast	0.014±0.002	0.19±0.09	2.1±0.7
T-47D	Breast	0.15±0.04	0.49±0.09	2.1±1.1
SKOV3	Ovarian	0.032±0.011	0.16±0.01	6.2±1.2
OVCAR3	Ovarian	0.015±0.001	0.072±0.023	0.46±0.06
OVCAR4	Ovarian	0.20±0.03	0.30±0.08	1.4±0.7
OVCAR8	Ovarian	0.028±0.002	0.28±0.03	0.79±0.09
LNCAP	Prostate	0.017±0.003	0.045±0.017	0.17±0.01
22RV1	Prostate	0.012±0.003	0.076±0.009	0.91±0.24
DU 145	Prostate	0.020±0.001	0.091±0.014	0.80±0.35
PC3	Prostate	0.046±0.003	0.21±0.04	1.4±0.4
LOX IMVI	Melanoma	0.0055±0.0005	0.034±0.010	1.3±0.3

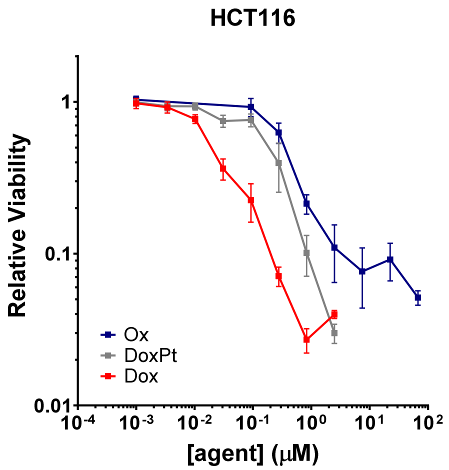

Figure S48. Dose-response curves showing the effect of oxaliplatin (Ox, blue), doxorubicin (Dox, red), and doxaliplatin (DoxPt, gray) on the viability of HCT116 human colon cancer cell line after a 72-hour exposure. Error bars represent standard deviations.

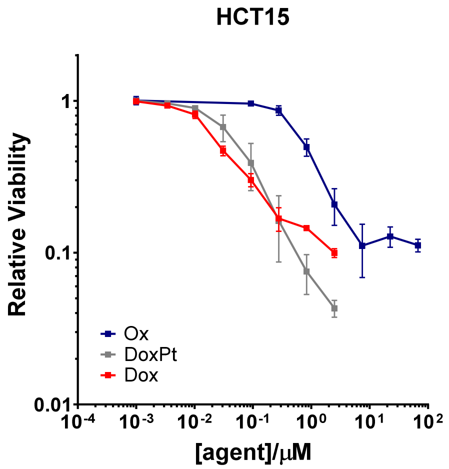

Figure S49. Dose-response curves showing the effect of oxaliplatin (Ox, blue), doxorubicin (Dox, red), and doxaliplatin (DoxPt, gray) on the viability of HCT15 human colon cancer cell line after a 72-hour exposure. Error bars represent standard deviations.

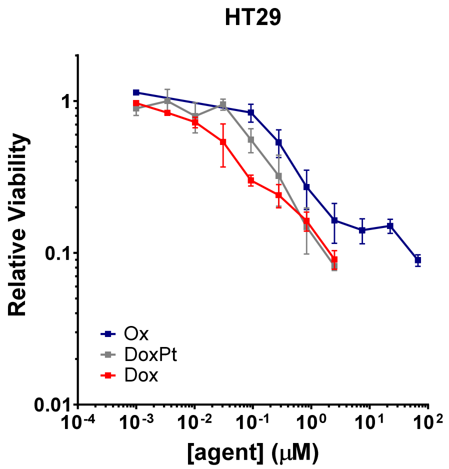

Figure S50. Dose-response curves showing the effect of oxaliplatin (Ox, blue), doxorubicin (Dox, red), and doxaliplatin (DoxPt, gray) on the viability of HT29 human colon cancer cell line after a 72-hour exposure. Error bars represent standard deviations.

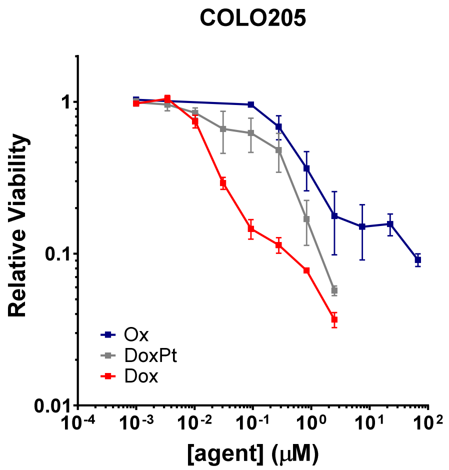

Figure S51. Dose-response curves showing the effect of oxaliplatin (Ox, blue), doxorubicin (Dox, red), and doxaliplatin (DoxPt, gray) on the viability of COLO205 human colon cancer cell line after a 72-hour exposure. Error bars represent standard deviations.

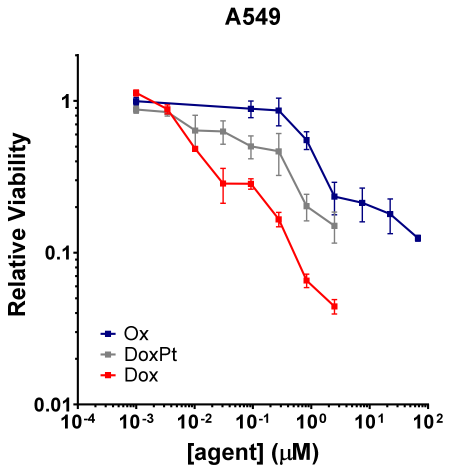

Figure S52. Dose-response curves showing the effect of oxaliplatin (Ox, blue), doxorubicin (Dox, red), and doxaliplatin (DoxPt, gray) on the viability of A549 human lung cancer cell line after a 72-hour exposure. Error bars represent standard deviations.

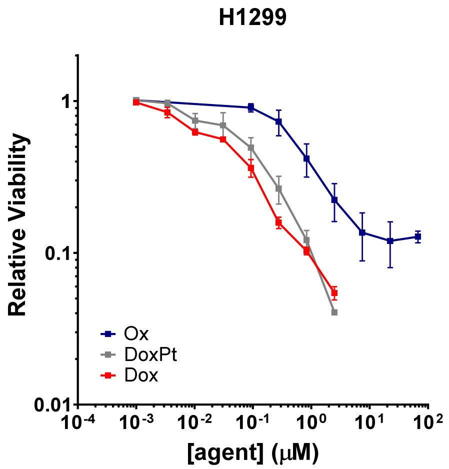

Figure S53. Dose-response curves showing the effect of oxaliplatin (Ox, blue), doxorubicin (Dox, red), and doxaliplatin (DoxPt, gray) on the viability of H1299 human lung cancer cell line after a 72-hour exposure. Error bars represent standard deviations.

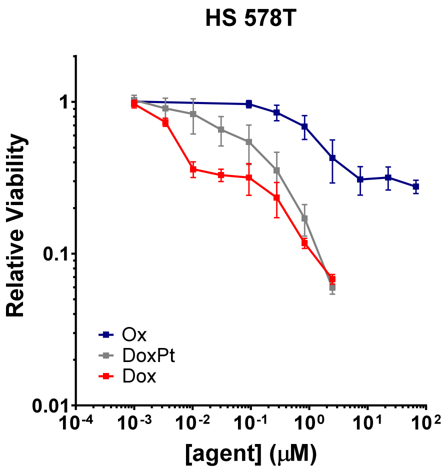

Figure S54. Dose-response curves showing the effect of oxaliplatin (Ox, blue), doxorubicin (Dox, red), and doxaliplatin (DoxPt, gray) on the viability of HS 578T human breast cancer cell line after a 72-hour exposure. Error bars represent standard deviations.

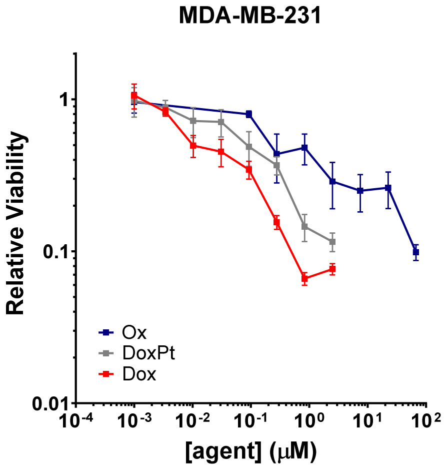

Figure S55. Dose-response curves showing the effect of oxaliplatin (Ox, blue), doxorubicin (Dox, red), and doxaliplatin (DoxPt, gray) on the viability of MDA-MB-231 human breast cancer cell line after a 72-hour exposure. Error bars represent standard deviations.

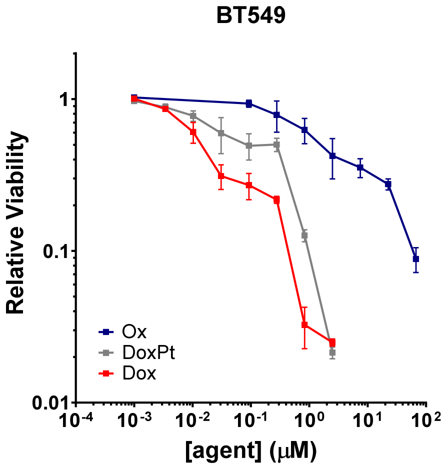

Figure S56. Dose-response curves showing the effect of oxaliplatin (Ox, blue), doxorubicin (Dox, red), and doxaliplatin (DoxPt, gray) on the viability of BT549 human breast cancer cell line after a 72-hour exposure. Error bars represent standard deviations.

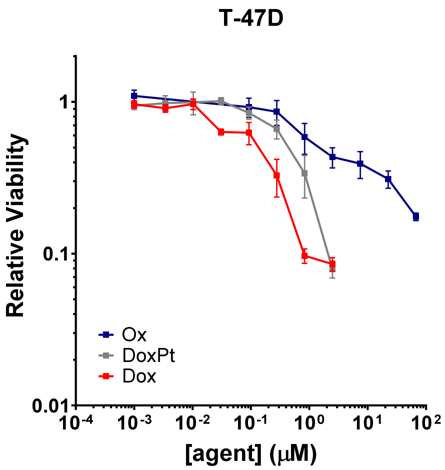

Figure S57. Dose-response curves showing the effect of oxaliplatin (Ox, blue), doxorubicin (Dox, red), and doxaliplatin (DoxPt, gray) on the viability of T-47D human breast cancer cell line after a 72-hour exposure. Error bars represent standard deviations.

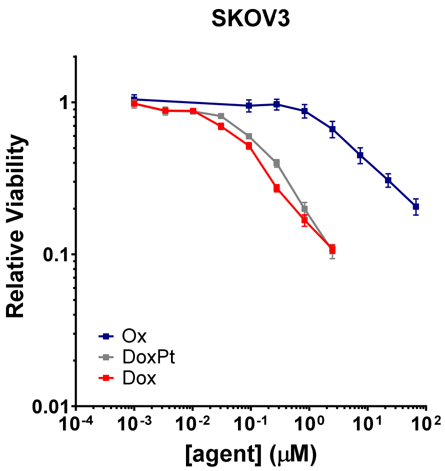

Figure S58. Dose-response curves showing the effect of oxaliplatin (Ox, blue), doxorubicin (Dox, red), and doxaliplatin (DoxPt, gray) on the viability of SKOV3 human ovarian cancer cell line after a 72-hour exposure. Error bars represent standard deviations.

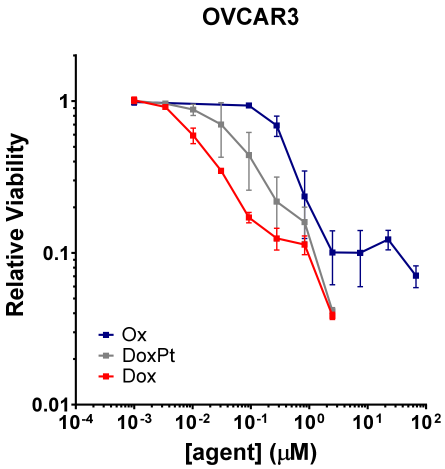

Figure S59. Dose-response curves showing the effect of oxaliplatin (Ox, blue), doxorubicin (Dox, red), and doxaliplatin (DoxPt, gray) on the viability of OVCAR3 human ovarian cancer cell line after a 72-hour exposure. Error bars represent standard deviations.

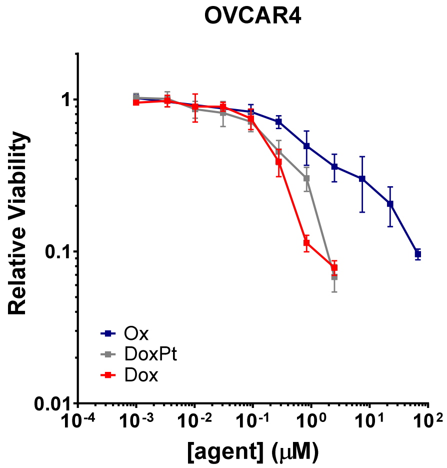

Figure S60. Dose-response curves showing the effect of oxaliplatin (Ox, blue), doxorubicin (Dox, red), and doxaliplatin (DoxPt, gray) on the viability of OVCAR4 human ovarian cancer cell line after a 72-hour exposure. Error bars represent standard deviations.

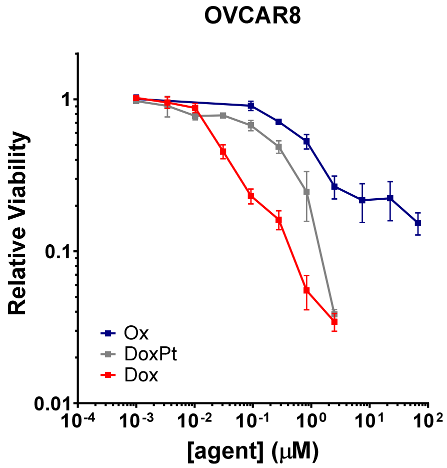

Figure S61. Dose-response curves showing the effect of oxaliplatin (Ox, blue), doxorubicin (Dox, red), and doxaliplatin (DoxPt, gray) on the viability of OVCAR8 human ovarian cancer cell line after a 72-hour exposure. Error bars represent standard deviations.

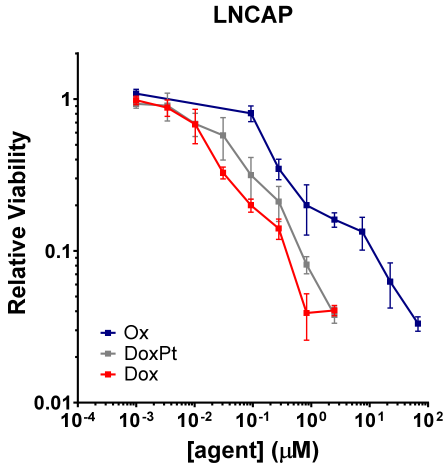

Figure S62. Dose-response curves showing the effect of oxaliplatin (Ox, blue), doxorubicin (Dox, red), and doxaliplatin (DoxPt, gray) on the viability of LNCAP human prostate cancer cell line after a 72-hour exposure. Error bars represent standard deviations.

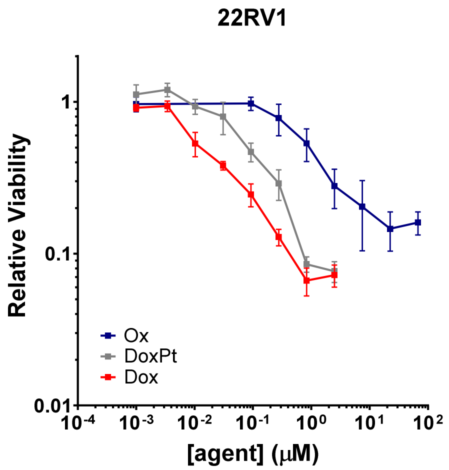

Figure S63. Dose-response curves showing the effect of oxaliplatin (Ox, blue), doxorubicin (Dox, red), and doxaliplatin (DoxPt, gray) on the viability of 22RV1 human prostate cancer cell line after a 72-hour exposure. Error bars represent standard deviations.

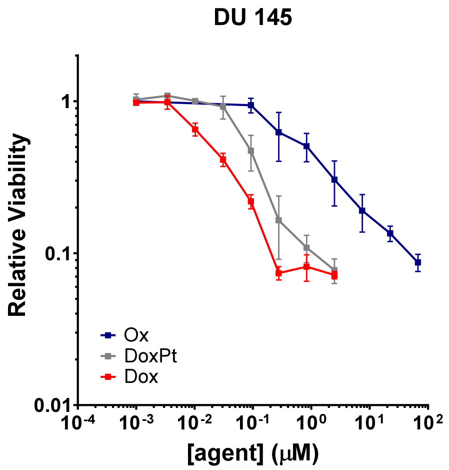

Figure S64. Dose-response curves showing the effect of oxaliplatin (Ox, blue), doxorubicin (Dox, red), and doxaliplatin (DoxPt, gray) on the viability of DU 145 human prostate cancer cell line after a 72-hour exposure. Error bars represent standard deviations.

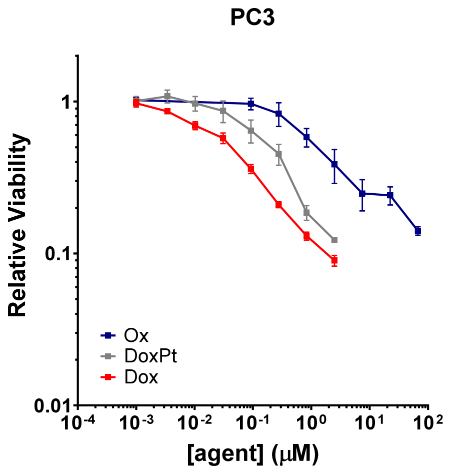

Figure S65. Dose-response curves showing the effect of oxaliplatin (Ox, blue), doxorubicin (Dox, red), and doxaliplatin (DoxPt, gray) on the viability of PC3 human prostate cancer cell line after a 72-hour exposure. Error bars represent standard deviations.

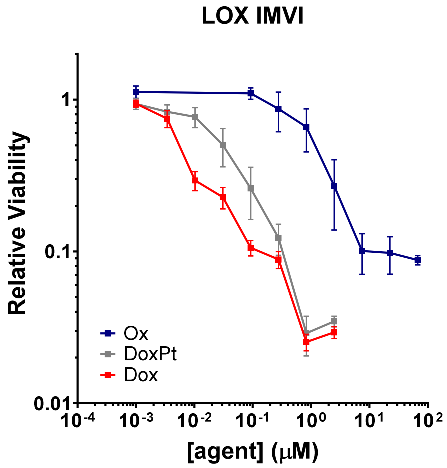

Figure S66. Dose-response curves showing the effect of oxaliplatin (Ox, blue), doxorubicin (Dox, red), and doxaliplatin (DoxPt, gray) on the viability of LOX IMVI human melanoma cancer cell line after a 72-hour exposure. Error bars represent standard deviations.

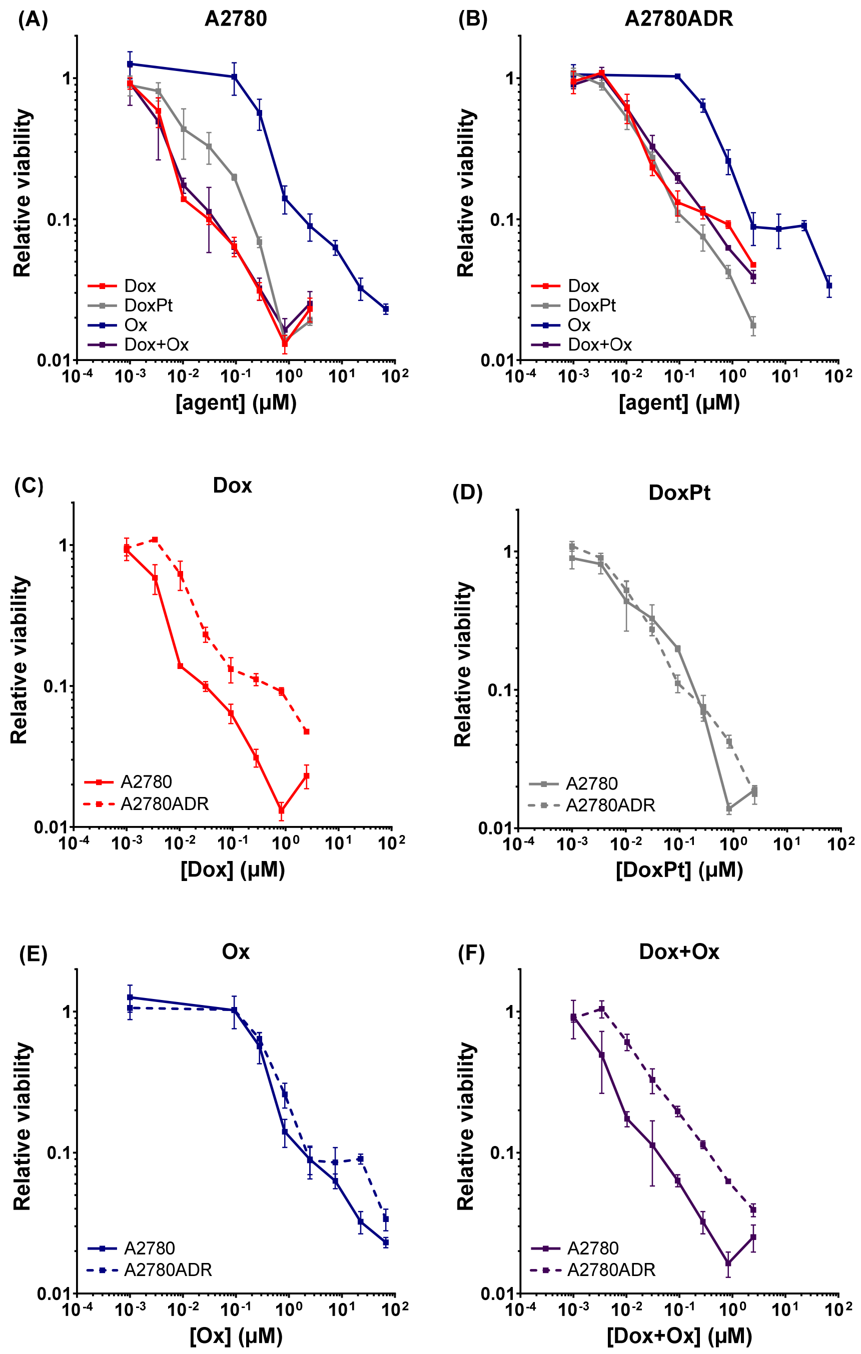

IC_{80} (μM)	A2780	A2780ADR	Fold Resistance
Dox	0.041±0.004	0.14±0.02	3.4
DoxPt	0.33±0.04	0.18±0.02	0.55
Ox	0.80±0.06	1.3±0.1	1.6
Dox+Ox (1:1 molar ratio)	0.056±0.028	0.29±0.09	5.2
Combination index [7]	1.4	2.3	

Figure S67. Dose-response curves showing the effect of oxaliplatin (Ox, blue), doxorubicin (Dox, red), doxaliplatin (DoxPt, gray), and a mixture of Dox and Ox (Dox+Ox, purple) on the viability of A2780 human ovarian cancer cell line (A) and A2780ADR doxorubicin-resistant human ovarian cancer cell line (B) after a 72-hour exposure. Dose-response curves showing the effect of Dox (C), DoxPt (D), Ox (E), and Dox+Ox (F) on A2780 (solid line) and A2780ADR (dashed line) cell lines. Error bars represent standard deviations.

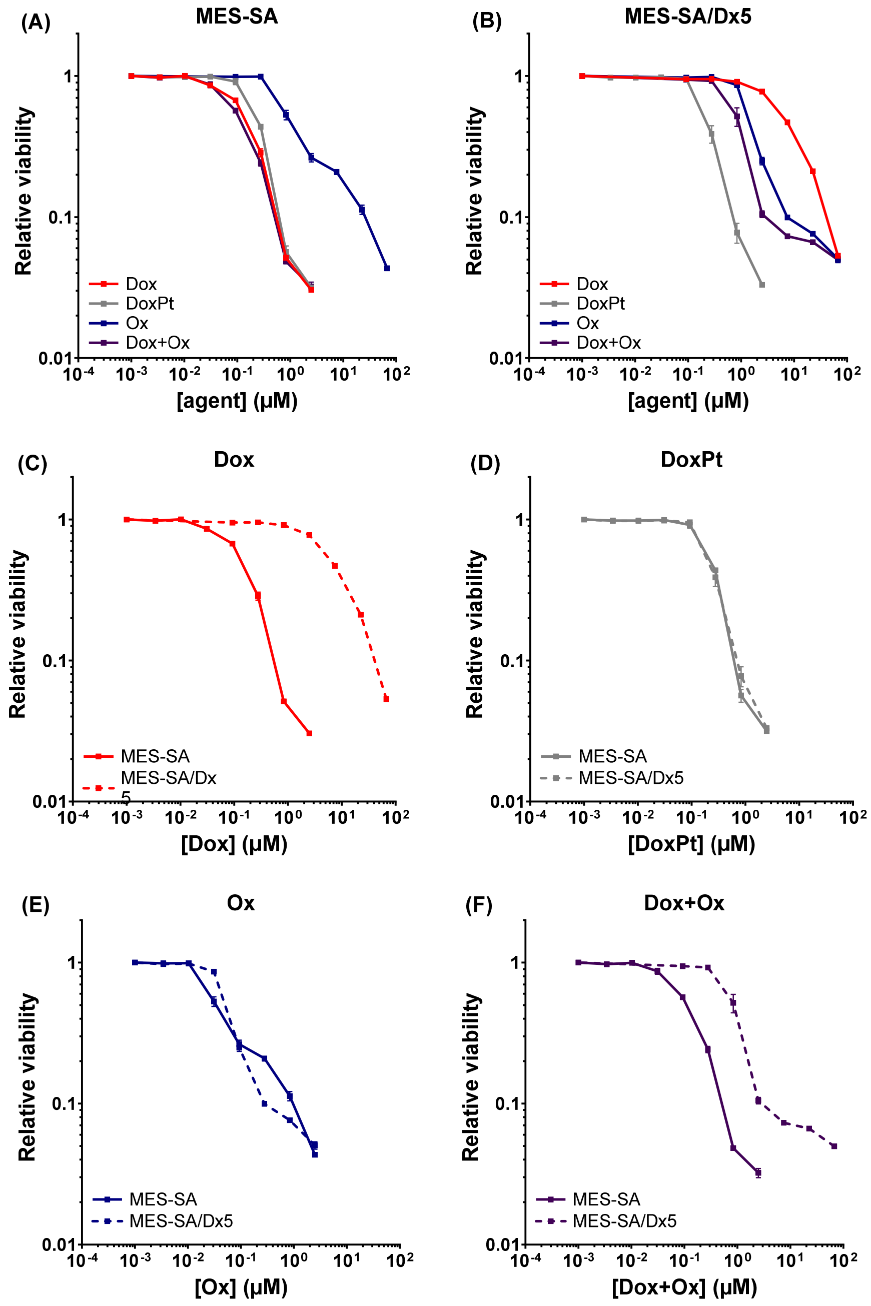

IC_{80} (μM)	MES-SA	MES-SA/Dx5	Fold Resistance
Dox	0.37±0.00	23±0.5	63
Ox	7.6±0.8	2.9±0.1	0.39
DoxPt	0.45±0.02	0.40±0.03	0.89
Dox+Ox (1:1 molar ratio)	0.31±0.02	1.7±0.1	5.4
Combination index	0.89	0.65	

Figure S68. Dose-response curves showing the effect of oxaliplatin (Ox, blue), doxorubicin (Dox, red), doxaliplatin (DoxPt, gray), and a mixture of Dox and Ox (Dox+Ox, purple) on the viability of MES-SA human uterine sarcoma cell line (A) and MES-SA/Dx5 doxorubicin-resistant human uterine sarcoma cell line (B) after a 72-hour exposure. Dose-response curves showing the effect of Dox (C), DoxPt (D), Ox (E), and Dox+Ox (F) on MES-SA (solid line) and MES-SA/Dx5 (dashed line) cell lines. Error bars represent standard deviations.

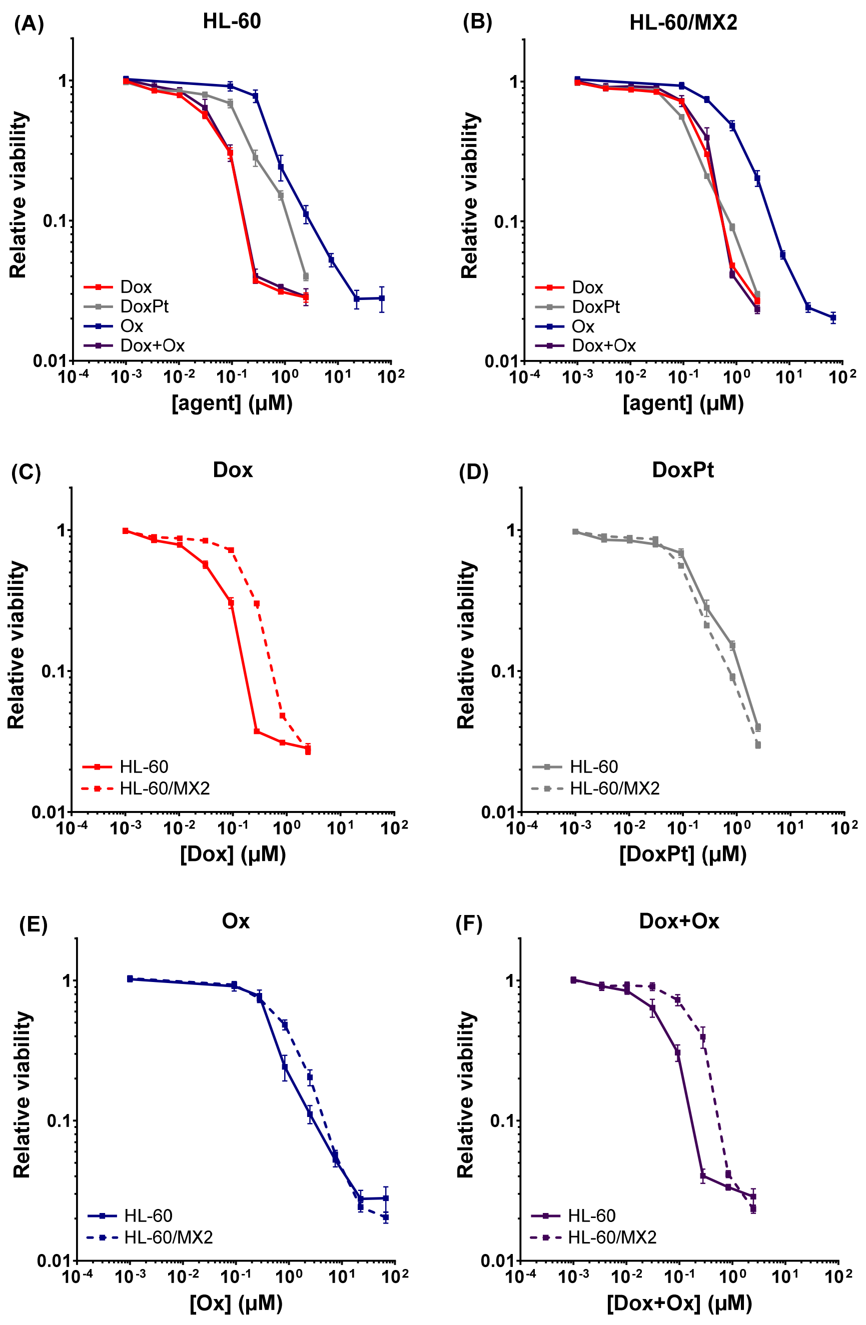

IC_{80} (μM)	HL-60	HL-60/MX2	Fold Resistance
Dox	0.13±0.01	0.37±0.01	2.9
DoxPt	0.56±0.05	0.30±0.00	0.54
Ox	0.99±0.10	2.5±0.2	2.6
Dox+Ox (1:1)	0.11±0.00	0.44±0.03	3.8
Combination index	1.0	1.4	

Figure S69. Dose-response curves showing the effect of oxaliplatin (Ox, blue), doxorubicin (Dox, red), doxaliplatin (DoxPt, gray), and a mixture of Dox and Ox (Dox+Ox, purple) on the viability of HL-60 human acute promyelocytic leukemia cell line (A) and HL-60/MX2 mitoxantrone-resistant human acute promyelocytic leukemia cell line (B) after a 72-hour exposure. Dose-response curves showing the effect of Dox (C), DoxPt (D), Ox (E), and Dox+Ox (F) on HL-60 (solid line) and HL-60/MX2 (dashed line) cell lines. Error bars represent standard deviations.

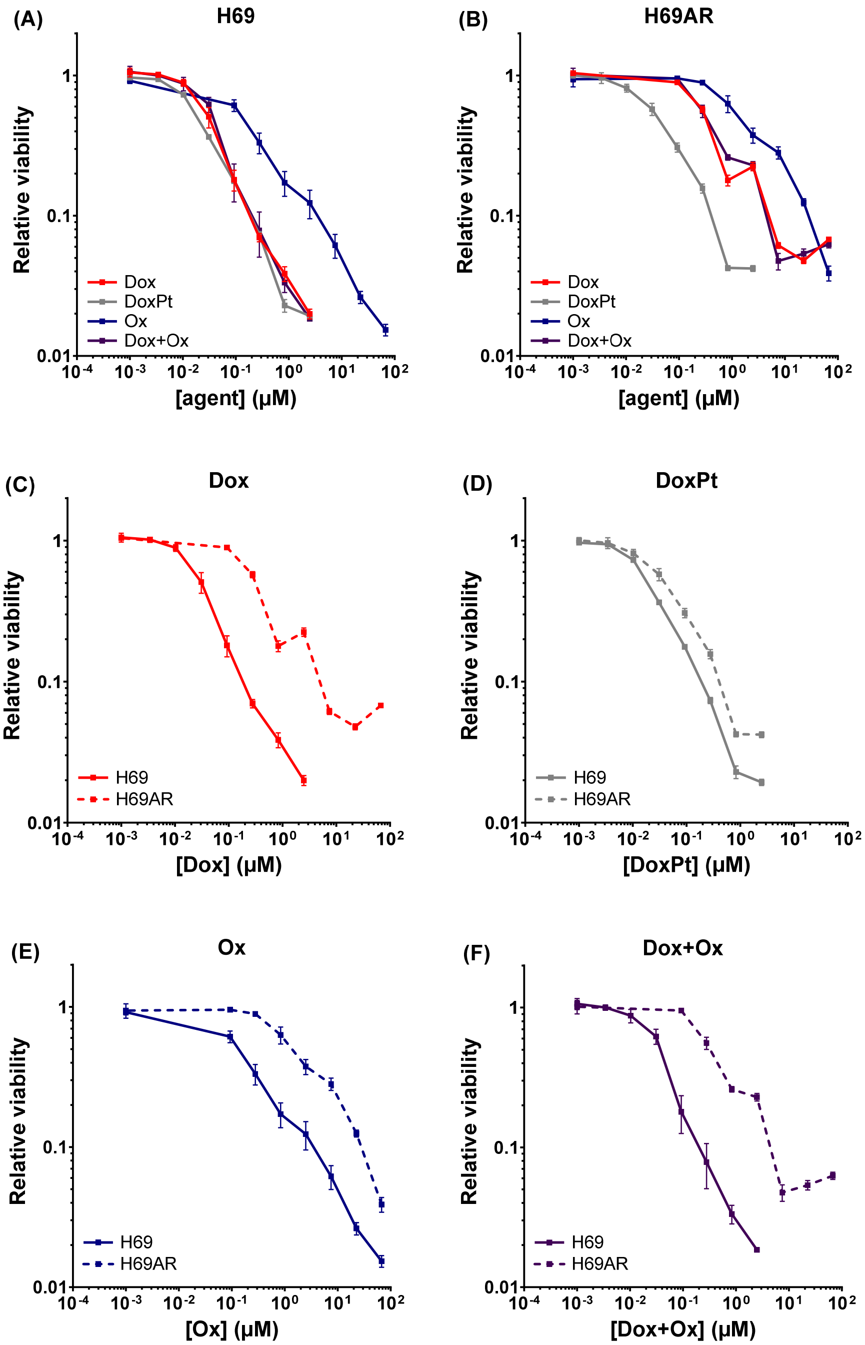

IC_{80} (μM)	H69	H69AR	Fold Resistance
Dox	0.077±0.008	0.92±0.05	12
DoxPt	0.067±0.003	0.16±0.00	2.5
Ox	0.79±0.16	8.7±1.5	11
Dox+Ox (1:1)	0.093±0.009	1.2±0.1	13
Combination index	1.3	1.4	

Figure S70. Dose-response curves showing the effect of oxaliplatin (Ox, blue), doxorubicin (Dox, red), doxaliplatin (DoxPt, gray), and a mixture of Dox and Ox (Dox+Ox, purple) on the viability of H69 human lung cancer cell line (A) and H69AR doxorubicin-resistant human lung cancer cell line (B) after a 72-hour exposure. Dose-response curves showing the effect of Dox (C), DoxPt (D), Ox (E), and Dox+Ox (F) on H69 (solid line) and H69AR (dashed line) cell lines. Error bars represent standard deviations.

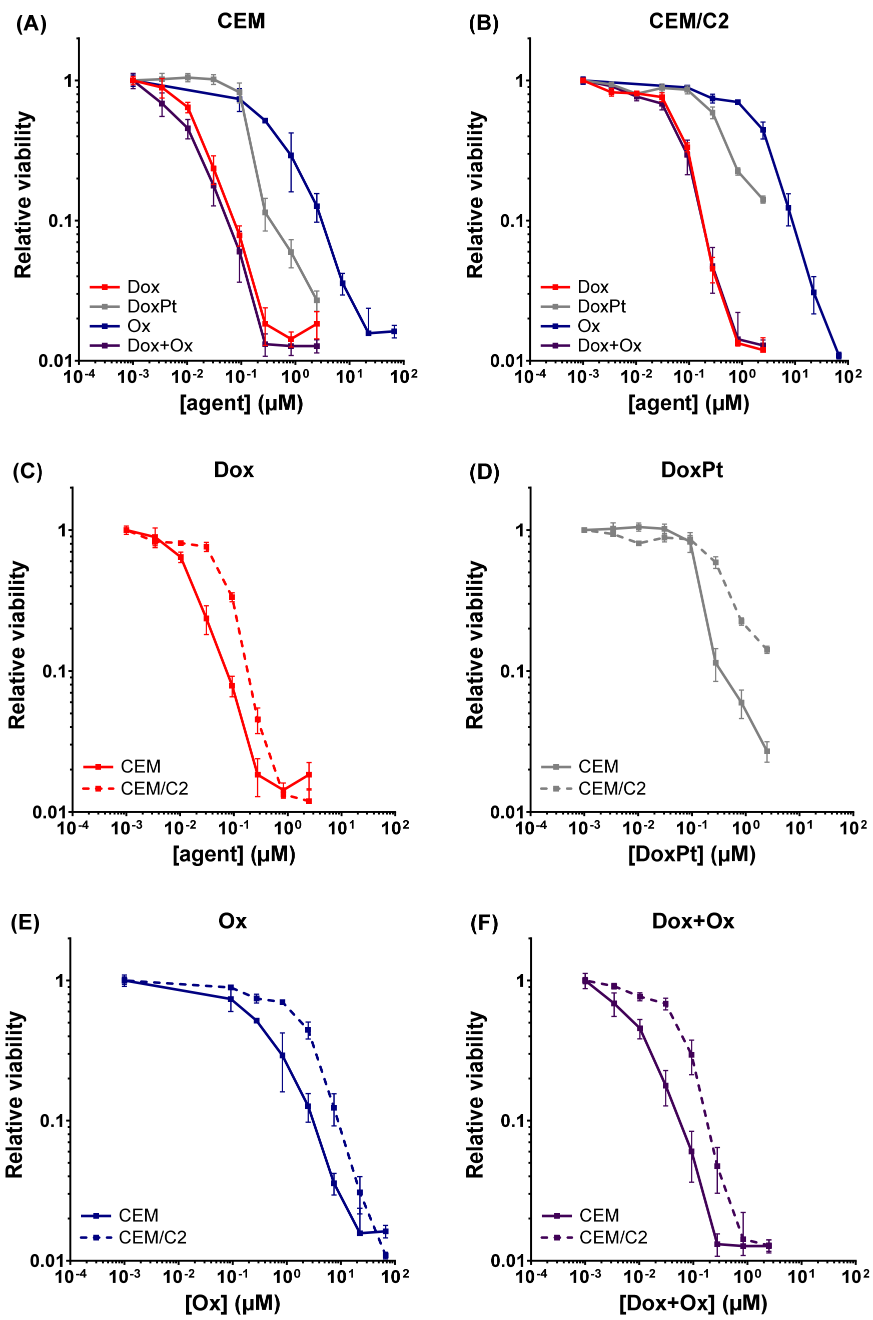

IC_{80} (μM)	CEM	CEM/C2	Fold Resistance
Dox	0.038±0.005	0.14±0.01	3.6
DoxPt	0.22±0.01	1.0±0.1	4.6
Ox	1.4±0.3	5.3±0.6	3.6
Dox+Ox (1:1)	0.029±0.005	0.12±0.02	4.2
Combination index	0.78	0.89	

Figure S71. Dose-response curves showing the effect of oxaliplatin (Ox, blue), doxorubicin (Dox, red), doxaliplatin (DoxPt, gray), and a mixture of Dox and Ox (Dox+Ox, purple) on the viability of CEM human acute lymphoblastic leukemia cell line (A) and CEM/C2 camptothecin-resistant human acute lymphoblastic leukemia cell line (B) after a 72-hour exposure. Dose-response curves showing the effect of Dox (C), DoxPt (D), Ox (E), and Dox+Ox (F) on CEM (solid line) and CEM/C2 (dashed line) cell lines. Error bars represent standard deviations.

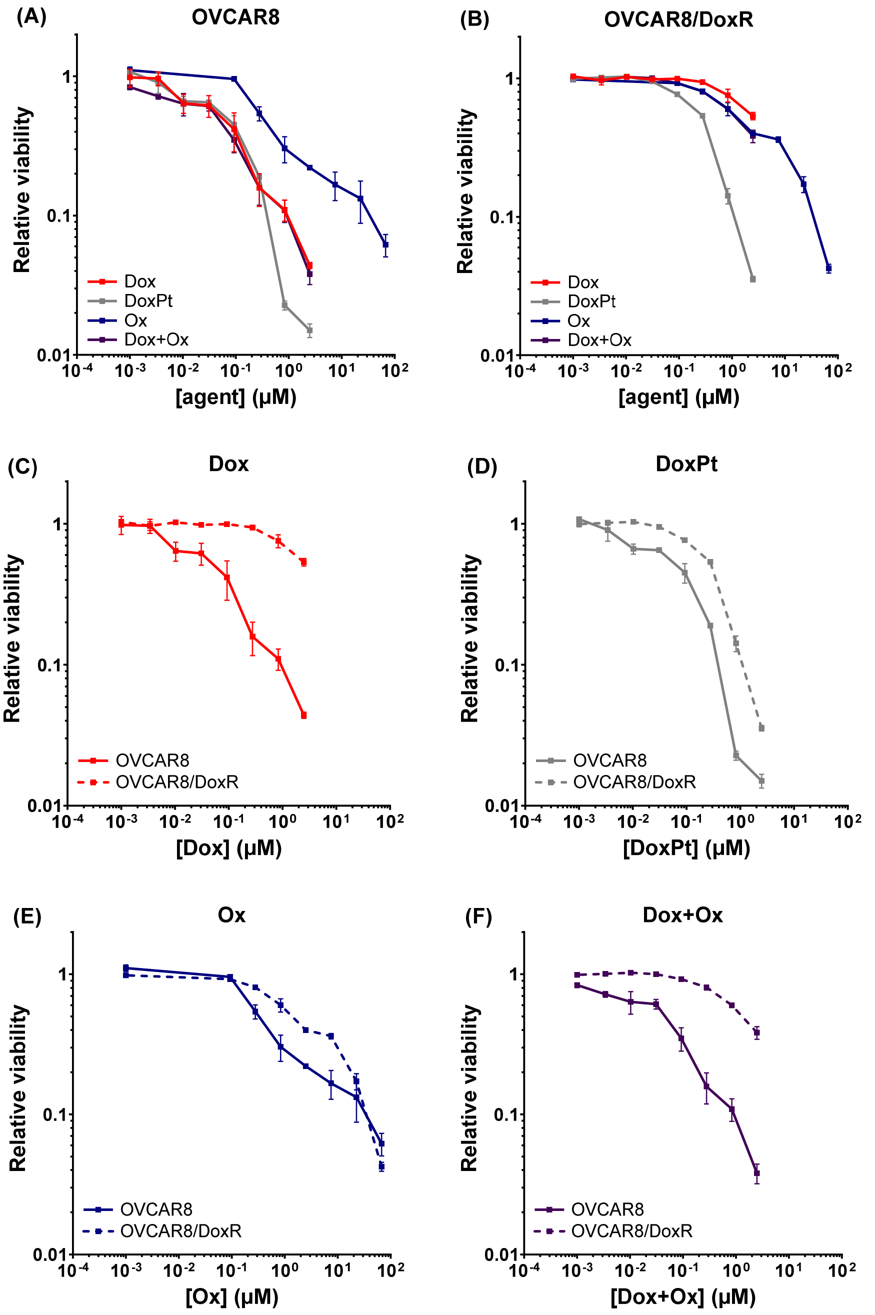

IC_{80} (μM)	OVCAR8	OVCAR8/DoxR	Fold Resistance
Dox	0.23±0.03	9.6	42
DoxPt	0.25±0.01	0.69±0.04	2.8
Ox	4.6±1.5	16±1	3.5
Dox+Ox (1:1)	0.21±0.05	27±20	128
Combination index	1.0	4.5	

Figure S72. Dose-response curves showing the effect of oxaliplatin (Ox, blue), doxorubicin (Dox, red), doxaliplatin (DoxPt, gray), and a mixture of Dox and Ox (Dox+Ox, purple) on the viability of OVCAR8 human ovarian cancer cell line (A) and OVCAR8/DoxR doxorubicin-resistant human ovarian cancer cell line (B) after a 72-hour exposure. Dose-response curves showing the effect of Dox (C), DoxPt (D), Ox (E), and Dox+Ox (F) on OVCAR8 (solid line) and OVCAR8/DoxR (dashed line) cell lines. Error bars represent standard deviations.

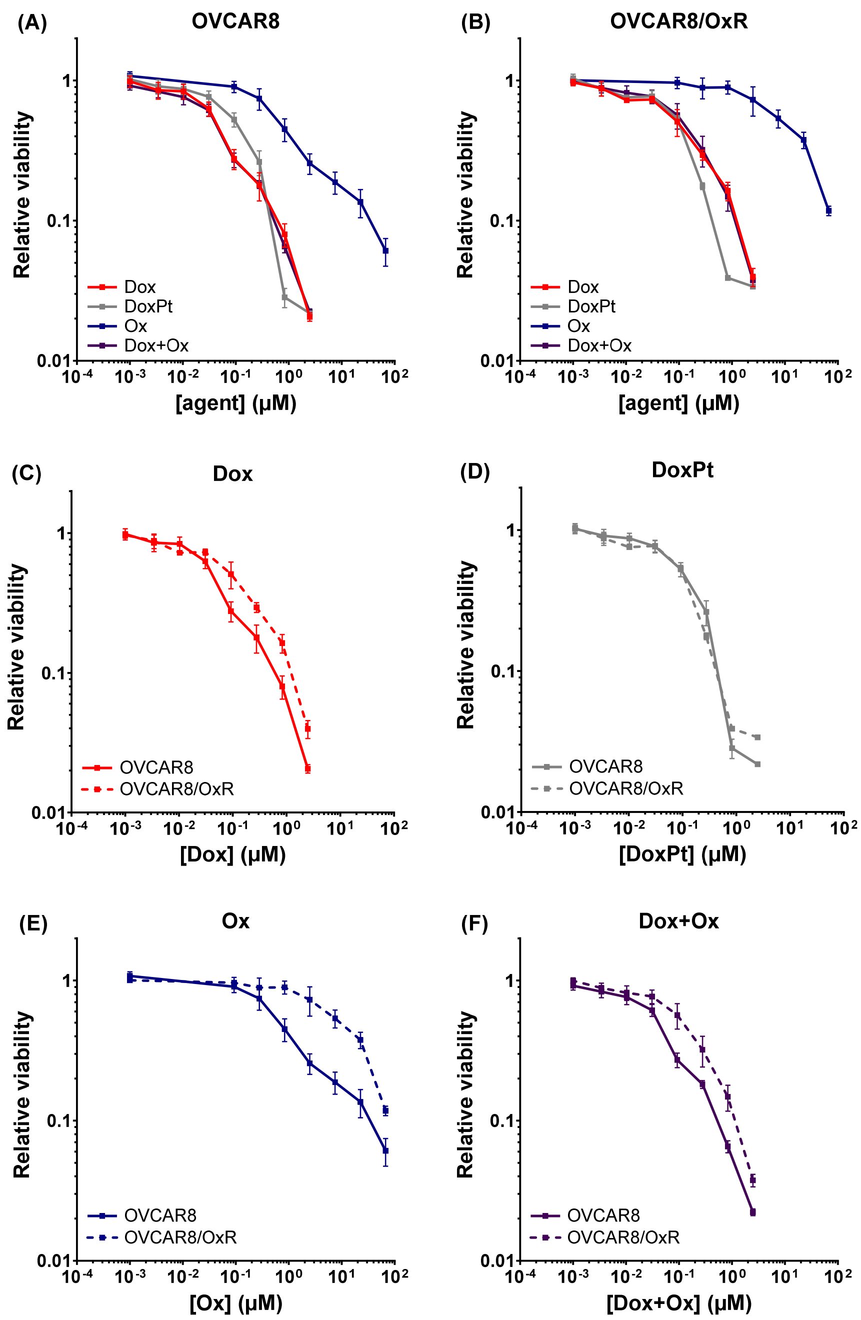

IC_{80} (μM)	OVCAR8	OVCAR8/OxR	Fold Resistance
Dox	0.41±0.02	0.84±0.01	2.1
DoxPt	0.37±0.02	0.31±0.01	0.84
Ox	12±3	38±5	3.2
Dox+Ox (1:1)	0.41±0.02	0.81±0.05	2.0
Combination index	1.0	0.98	

Figure S73. Dose-response curves showing the effect of oxaliplatin (Ox, blue), doxorubicin (Dox, red), doxaliplatin (DoxPt, gray), and a mixture of Dox and Ox (Dox+Ox, purple) on the viability of OVCAR8 human ovarian cancer cell line (A) and OVCAR8/OxR oxaliplatin-resistant human ovarian cancer cell line (B) after a 72-hour exposure. Dose-response curves showing the effect of Dox (C), DoxPt (D), Ox (E), and Dox+Ox (F) on OVCAR8 (solid line) and OVCAR8/OxR (dashed line) cell lines. Error bars represent standard deviations.

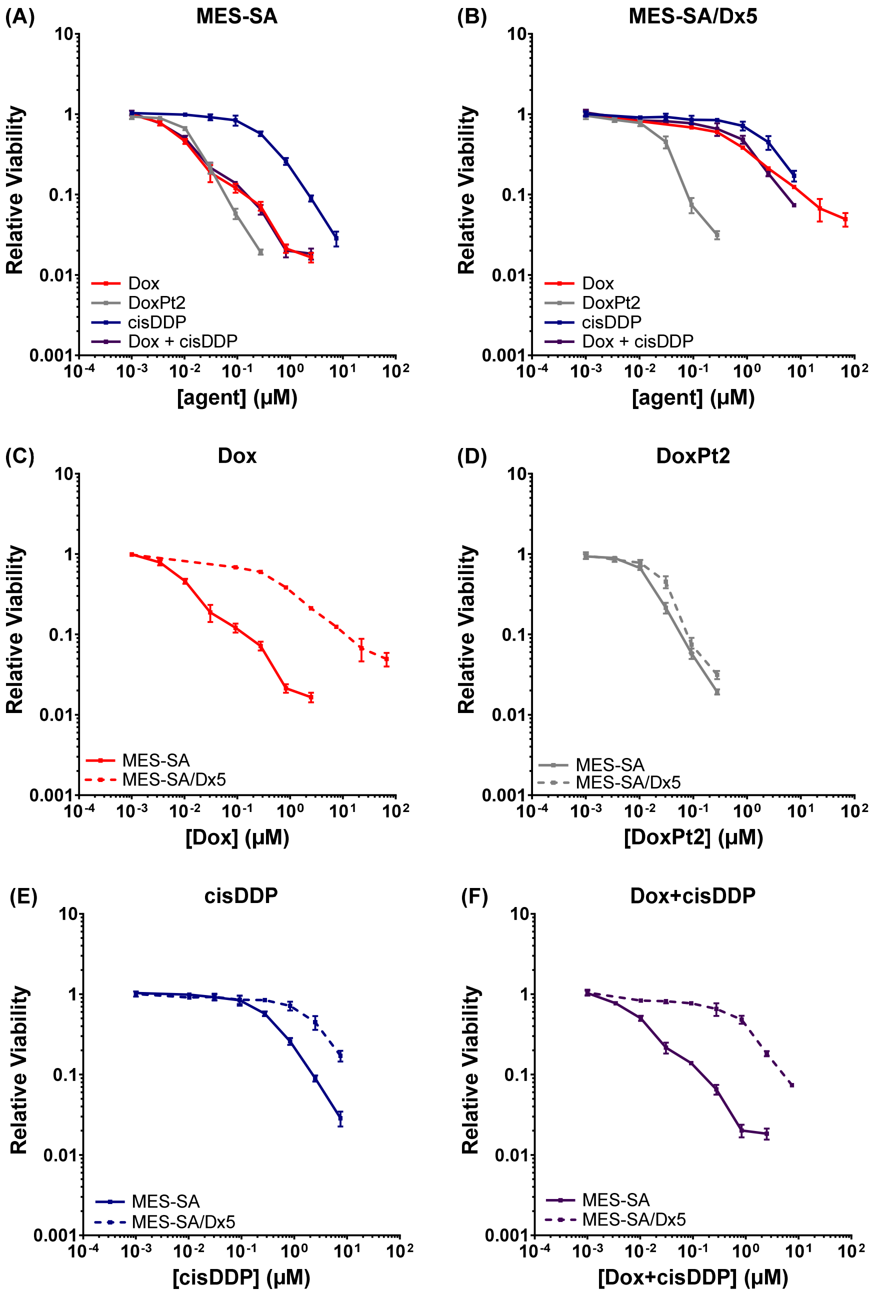

IC_{80} (μM)	MES-SA	MES-SA/Dx5	Fold Resistance
Dox	0.030±0.006	2.8±0.5	91
cisDDP	1.0±0.1	6.3±0.8	6.1
DoxPt2	0.032±0.002	0.052±0.004	1.6
Dox+cisDDP (1:1 molar ratio)	0.039±0.003	2.4±0.1	61
Combination index	1.3	1.2	

Figure S74. Dose-response curves showing the effect of cisplatin (cisDDP, blue), doxorubicin (Dox, red), doxaliplatin2 (DoxPt2, gray), and a mixture of Dox and cisDDP (Dox+cisDDP, purple) on the viability of MES-SA human uterine sarcoma cell line (A) and MES-SA/Dx5 doxorubicin-resistant human uterine sarcoma cell line (B) after a 72-hour exposure. Dose-response curves showing the effect of Dox (C), DoxPt2 (D), cisDDP (E), and Dox+cisDDP (F) on MES-SA (solid line) and MES-SA/Dx5 (dashed line) cell lines. Error bars represent standard deviations.

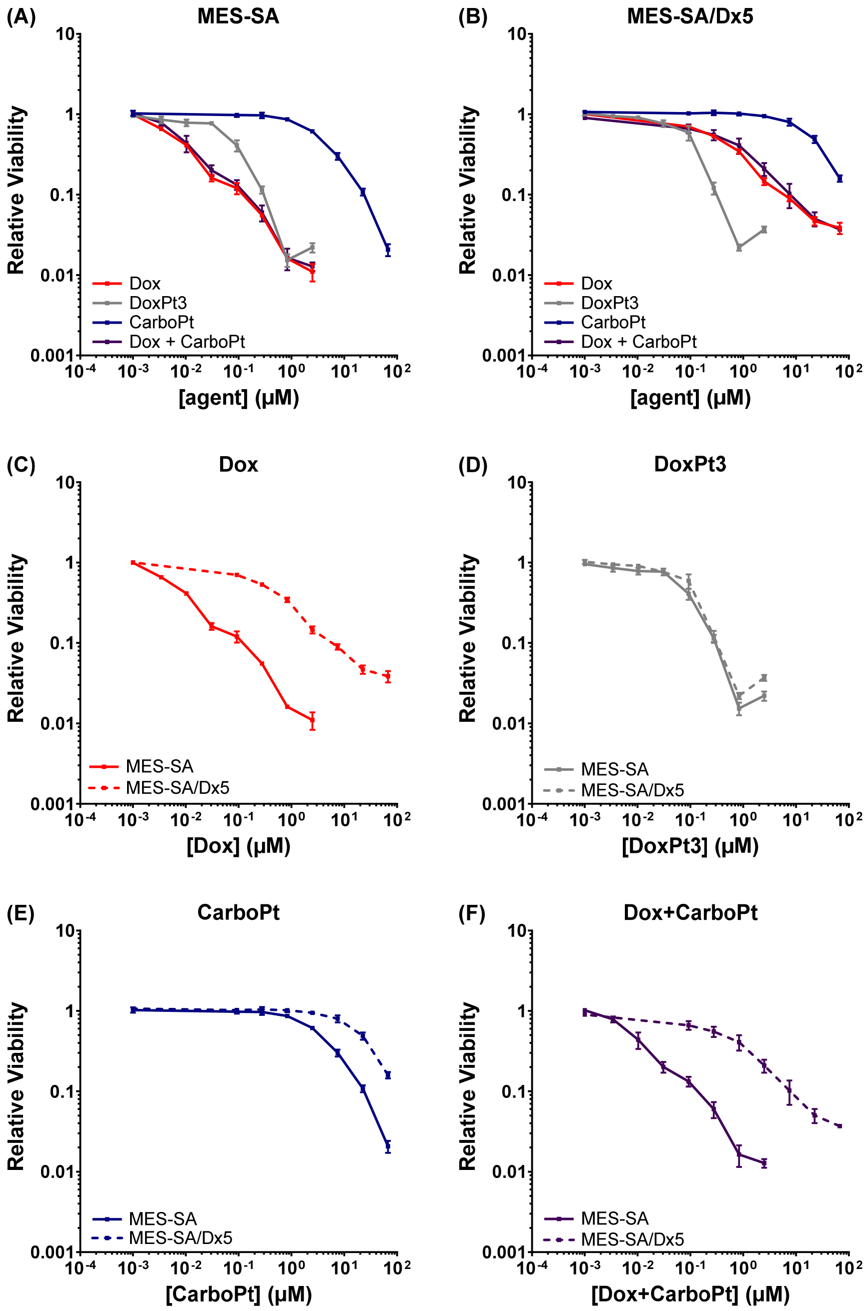

IC_{80} (μM)	MES-SA	MES-SA/Dx5	Fold Resistance
Dox	0.027±0.003	1.9±0.1	70
CarboPt	12±1	57±3	4.8
DoxPt3	0.18±0.02	0.24±0.02	1.3
Dox+ CarboPt (1:1 molar ratio)	0.033±0.005	2.7±0.5	82
Combination index	1.3	1.5	

Figure S75. Dose-response curves showing the effect of carboplatin (CarboPt, blue), doxorubicin (Dox, red), doxaliplatin3 (DoxPt3, gray), and a mixture of Dox and CarboPt (Dox+CarboPt, purple) on the viability of MES-SA human uterine sarcoma cell line (A) and MES-SA/Dx5 doxorubicin-resistant human uterine sarcoma cell line (B) after a 72-hour exposure. Dose-response curves showing the effect of Dox (C), DoxPt2 (D), CarboPt (E), and Dox+ CarboPt (F) on MES-SA (solid line) and MES-SA/Dx5 (dashed line) cell lines. Error bars represent standard deviations.

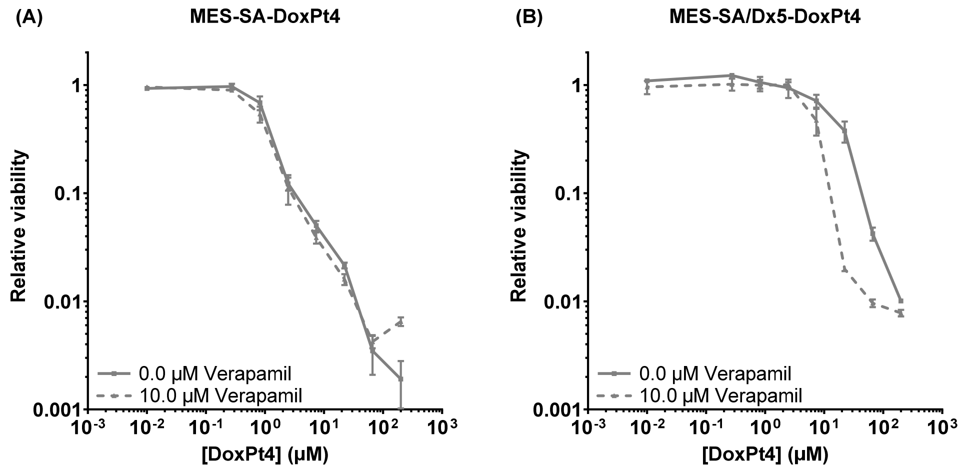

IC80 (μM)	MES-SA	MES-SA/Dx5	Fold Resistance
+ 0.0 μM verapamil	2.0±0.0	33±3	17
+ 10 μM verapamil	1.8±0.2	10±1	5.5

Figure S76. Dose-response curves showing the effect of doxaliplatin4 (DoxPt4, gray) on the viability of MES-SA human uterine sarcoma cell line (A) and MES-SA/Dx5 doxorubicin-resistant human uterine sarcoma cell line (B) after a 72-hour exposure. Dashed lines show the effect of verapamil (10 μM) on the activity of DoxPt4. Error bars represent standard deviations.

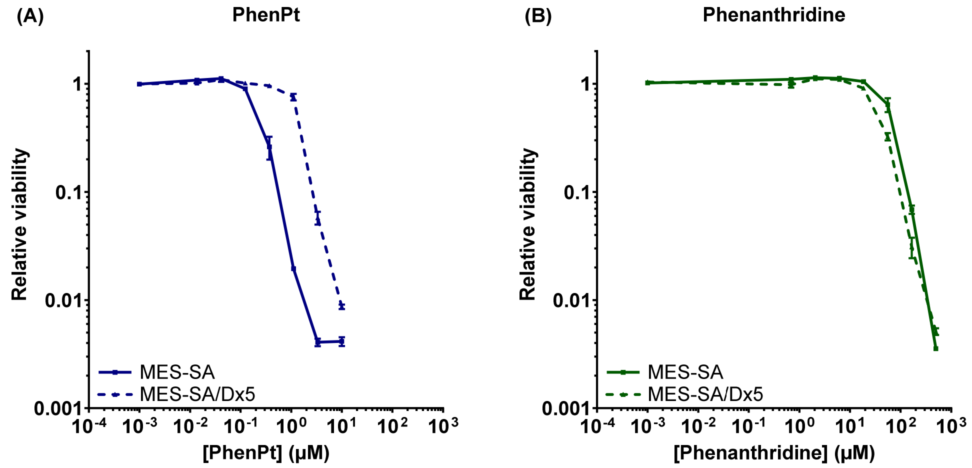

IC80 (μM)	MES-SA	MES-SA/Dx5	Fold Resistance
Phenanthridine	111±5	72±3	0.7
PhenPt	0.43±0.03	2.3±0.1	5.4

Figure S77. Dose-response curves showing the effect of phenanthriplatin (PhenPt) (A) and phenanthridine (B) on the viability of MES-SA human uterine sarcoma cell line (solid line) and MES-SA/Dx5 doxorubicin-resistant human uterine sarcoma cell line (dashed line). Error bars represent standard deviations.

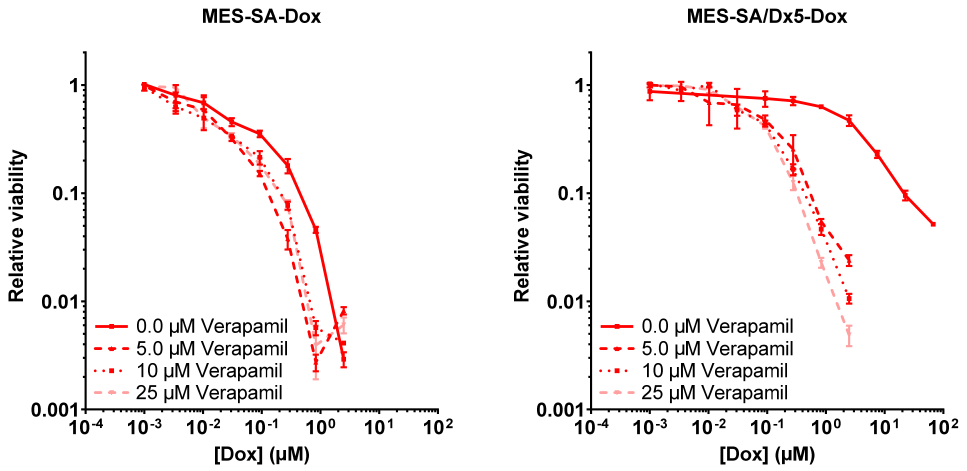

IC80 (μM)	MES-SA	MES-SA/Dx5	Fold Resistance
+ 0.0 μM verapamil	0.24±0.03	9.0±0.6	38
+ 5.0 μM verapamil	0.071±0.004	0.34±0.06	4.8
+ 10 μM verapamil	0.093±0.008	0.24±0.01	2.6
+ 25 μM verapamil	0.095±0.016	0.20±0.01	2.1

Figure S78. Dose-response curves showing the effect of verapamil at different concentrations on the activity of doxorubicin against the viability of MES-SA human uterine sarcoma cell line (left) and MES-SA/Dx5 doxorubicin-resistant human uterine sarcoma cell line (right) after a 72-hour exposure. Error bars represent standard deviations.

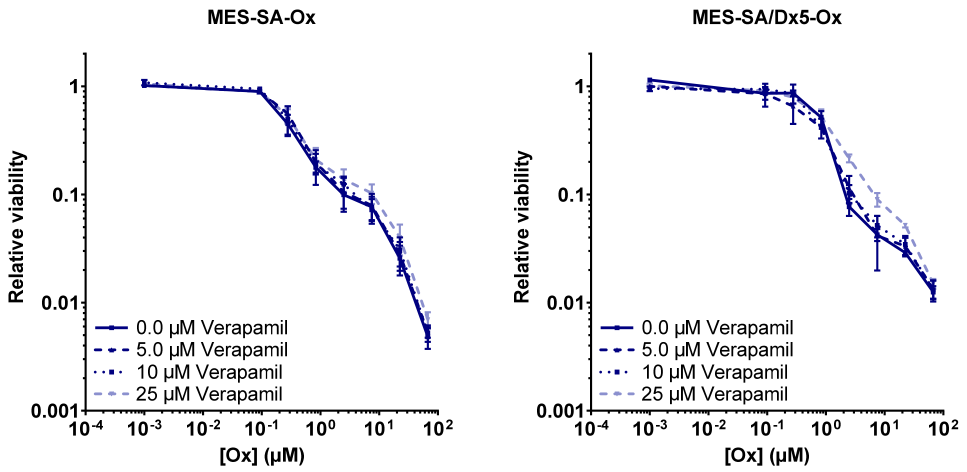

IC80 (μM)	MES-SA	MES-SA/Dx5	Fold Resistance
+ 0.0 μM verapamil	0.65±0.12	1.5±0.1	2.3
+ 5.0 μM verapamil	0.88±0.19	1.8±0.2	2.0
+ 10 μM verapamil	0.87±0.17	1.5±0.1	1.8
+ 25 μM verapamil	1.0±0.4	3.0±0.2	2.9

Figure S79. Dose-response curves showing the effect of verapamil at different concentrations on the activity of oxaliplatin against the viability of MES-SA human uterine sarcoma cell line (left) and MES-SA/Dx5 doxorubicin-resistant human uterine sarcoma cell line (right) after a 72-hour exposure. Error bars represent standard deviations.

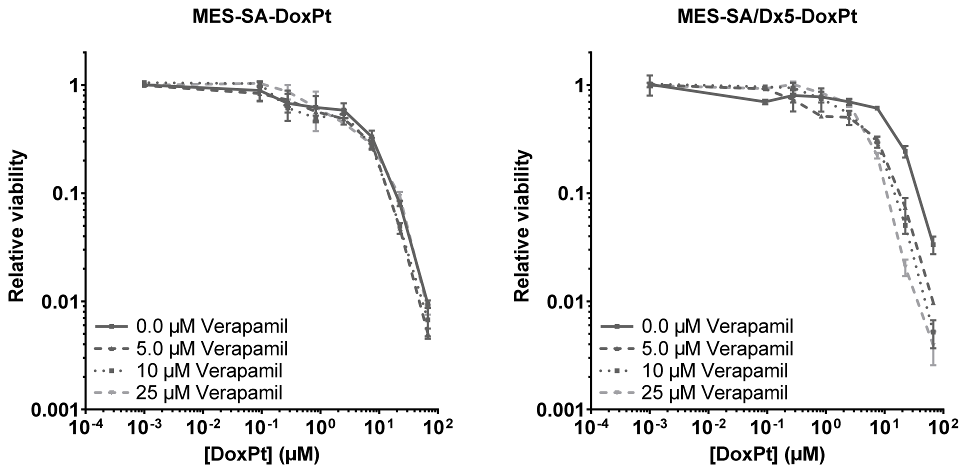

IC$_{80}$ (μM)	MES-SA	MES-SA/Dx5	Fold Resistance
+ 0.0 μM verapamil	0.44±0.03	0.95±0.05	2.2
+ 5.0 μM verapamil	0.37±0.03	0.41±0.01	1.1
+ 10 μM verapamil	0.36±0.01	0.44±0.07	1.2
+ 25 μM verapamil	0.43±0.03	0.30±0.00	0.7

Figure S80. Dose-response curves showing the effect of verapamil at different concentrations on the activity of DoxPt against the viability of MES-SA human uterine sarcoma cell line (left) and MES-SA/Dx5 doxorubicin-resistant human uterine sarcoma cell line (right) after a 72-hour exposure. Error bars represent standard deviations.

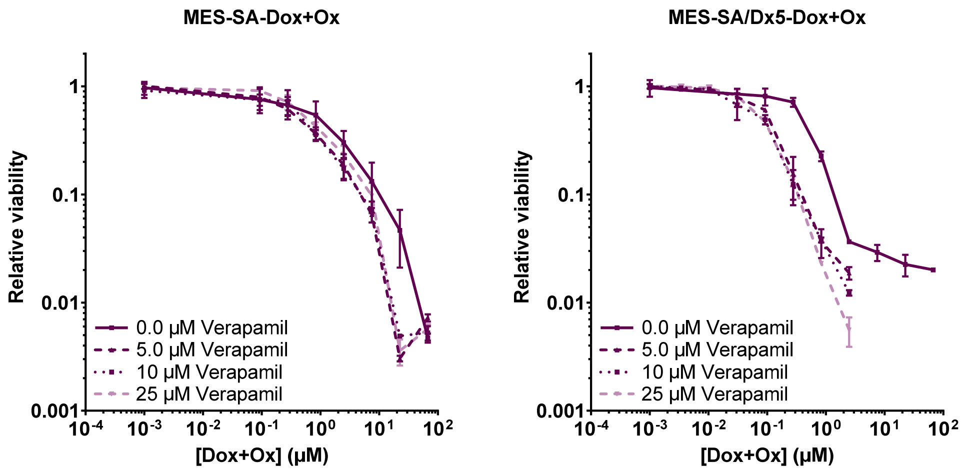

IC$_{80}$ (μM)	MES-SA	MES-SA/Dx5	Fold Resistance
+ 0.0 μM verapamil	0.16±0.03	1.0±0.0	6.4
+ 5.0 μM verapamil	0.084±0.001	0.25±0.03	3.0
+ 10 μM verapamil	0.079±0.006	0.21±0.02	2.6
+ 25 μM verapamil	0.11±0.02	0.20±0.01	1.8

Figure S81. Dose-response curves showing the effect of verapamil at different concentrations on the activity of a physical mixture of doxorubicin and oxaliplatin (Dox+Ox, 1:1 molar ratio) against the viability of MES-SA human uterine sarcoma cell line (left) and MES-SA/Dx5 doxorubicin-resistant human uterine sarcoma cell line (right) after a 72-hour exposure. Error bars represent standard deviations.

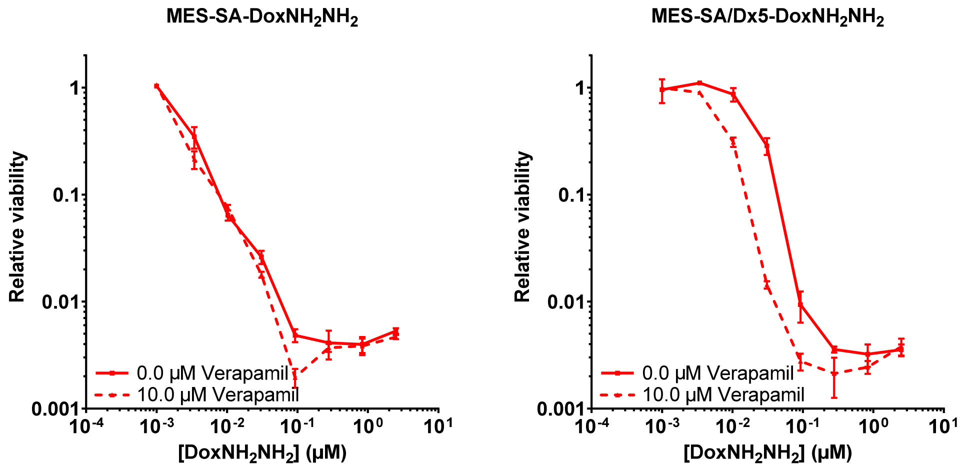

IC₈₀ (μM)	MES-SA	MES-SA/Dx5	Fold Resistance
+ 0.0 μM verapamil	0.0055±0.0007	0.033±0.006	6.0
+ 10 μM verapamil	0.0037±0.0002	0.013±0.000	3.5

Figure S82. Dose-response curves showing the effect of $DoxNH_2NH_2$ on the viability of MES-SA human uterine sarcoma cell line (left) and MES-SA/Dx5 doxorubicin-resistant human uterine sarcoma cell line (right) after a 72-hour exposure. Dashed lines show the effect of verapamil (10 μM) on the activity of $DoxNH_2NH_2$. Error bars represent standard deviations.

S7. Colorectal cancer organoid generation and related cytotoxicity studies.

Colonic crypts were isolated and colon organoids were generated from the following mouse genotypes on a C57Bl/6 background: **1.** Rosa-LSL-TdTomato (hereafter LSL-TdT), **2.** $P53^{fl/fl}$; LSL- TdT, and **3.** LSL-$KRAS^{G12D}$; $p53^{fl/fl}$; LSL-TdT. Subsequently, the pSECC-APC plasmid (carrying Cre, Cas9 and sgAPC)[8] was transfected using Lipofectamine™ 2000 (ThermoFisher catalog # 11668019) into each of the above three genotypes of colon organoids. This procedure generated in a single step **1.** $APC^{-/-}$; TdT^{+} (A-TdT), **2.** $APC^{-/-}$; $p53^{-/-}$; TdT^{+} (AP-TdT), and **3.** $APC^{-/-}$; $KRAS^{G12D/+}$; $p53^{-/-}$; TdT^{+} (AKP-TdT) colorectal cancer organoids, which were selected with Wnt withdrawal. The $APC^{-/-}$; $KRAS^{G12D/+}$; $p53^{-/-}$; $SMAD4^{-/-}$; TdT^{+} (AKPS-TdT) organoids were generated from the AKP-TdT organoids with CRISPR deletion of SMAD4 and subsequent TGF-β1 selection (PeproTech catalog #: 100-21). The $APC^{-/-}$; $KRAS^{G12D/+}$; $p53^{-/-}$; $ZsGreen^{+}$ (AKP-ZsG) colorectal cancer organoids were generated in a similar manner using pSECC-APC starting with LSL-$Kras^{G12D}$; $p53^{fl/fl}$; LSL-ZsG organoids.

To determine IC_{50} values for cytotoxic agents, trypsinized organoids were seeded in 10 μL drops containing 67 v/v% Matrigel® in 48-well plates and incubated at 37 °C for 15 min. Drug-containing media (300 μL) were then added. For experiments with P-gp inhibition, verapamil was applied at the indicated concentration at the time of the cytotoxic agent treatment. The organoids were incubated for 72-96 h following the treatment. Organoid growth was measured by a resazurin assay. In brief, at the end of drug treatment, resazurin was added to the organoid-containing wells at a final concentration of 50 μg/mL. The baseline fluorescence was determined immediately. The organoids were then incubated at 37 °C for 2 h. The fluorescence of the resorufin product was measured at 560/590 nm and baseline corrected. The growth at each drug concentration was normalized to the untreated control.

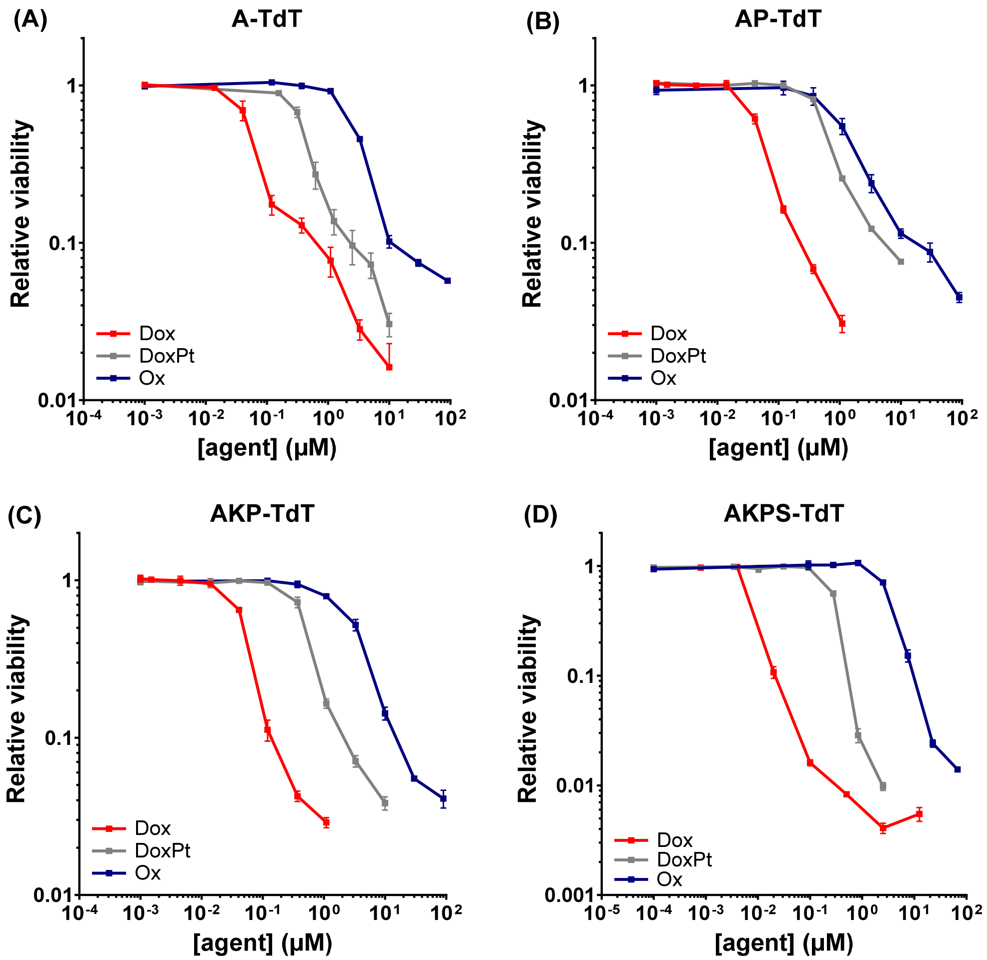

IC_{50} (µM)	A-TdT	AP-TdT	AKP-TdT	AKPS-TdT
Dox	0.058±0.006	0.096±0.005	0.052±0.001	0.011±0.000
DoxPt	0.42±0.03	1.3±0.0	0.54±0.02	0.30±0.00
Ox	3.1±0.0	4.1±0.1	3.2±0.2	3.6±0.1

IC_{80} (µM)	A-TdT	AP-TdT	AKP-TdT	AKPS-TdT
Dox	0.11±0.01	0.052±0.001	0.091±0.002	0.016±0.000
DoxPt	0.83±0.11	0.67±0.01	1.0±0.0	0.46±0.02
Ox	5.9±0.2	1.3±0.2	8.9±0.8	6.2±0.2

Figure S83. Representative dose-response curves showing the effect of oxaliplatin (Ox, blue), doxorubicin (Dox, red), and doxaliplatin (DoxPt, gray) on the viability of murine colorectal cancer organoids with different genetic background after a 72-hour exposure. (A) Dose-response curves for $APC^{-/-}$; TdT^{+} (A-TdT) colorectal cancer organoids. (B) Dose-response curves for $APC^{-/-}$; $p53^{-/-}$; TdT^{+} (AP-TdT) colorectal cancer organoids. (C) $APC^{-/-}$; $KRAS^{G12D/+}$; $p53^{-/-}$; TdT^{+} (AKP-TdT) colorectal cancer organoids. (D) $APC^{-/-}$; $KRAS^{G12D}$; $P53^{-/-}$; $SMAD4^{-/-}$; TdT^{+} (AKPS-TdT) colorectal cancer organoids. Error bars represent standard deviations.

S8. Resistant colorectal cancer organoid generation and related cytotoxicity studies.

Drug-resistant AKP-TdT organoid lines were generated via iterative rounds of drug selection with escalating concentrations. Briefly, organoids were seeded in seven Matrigel® droplets (67 v/v%, 10 µL) per well on a 12-well plate. The organoids were treated with cytotoxic agents at three different concentrations (1×, 2×, and 4× of the IC_{80} values determined from non-resistant organoids). After one week of drug treatment, organoids from the well with the highest drug concentration permitting outgrowth were collected, trypsinized, and seeded into four wells on a 12-well plate with seven Matrigel® droplets per well. Media containing the cytotoxic agent were added to wells at three concentrations (0.5×, 1×, and 2× of the previous highest outgrowth concentration). The fourth well was left drug-free as a backup. This procedure was repeated for 20 passages. The highest concentration permitting outgrowth was recorded at each passage. This experiment was performed in three independent replicate lines for each drug. Drug-resistant AKPS-TdT organoid lines were generated in a similar manner.

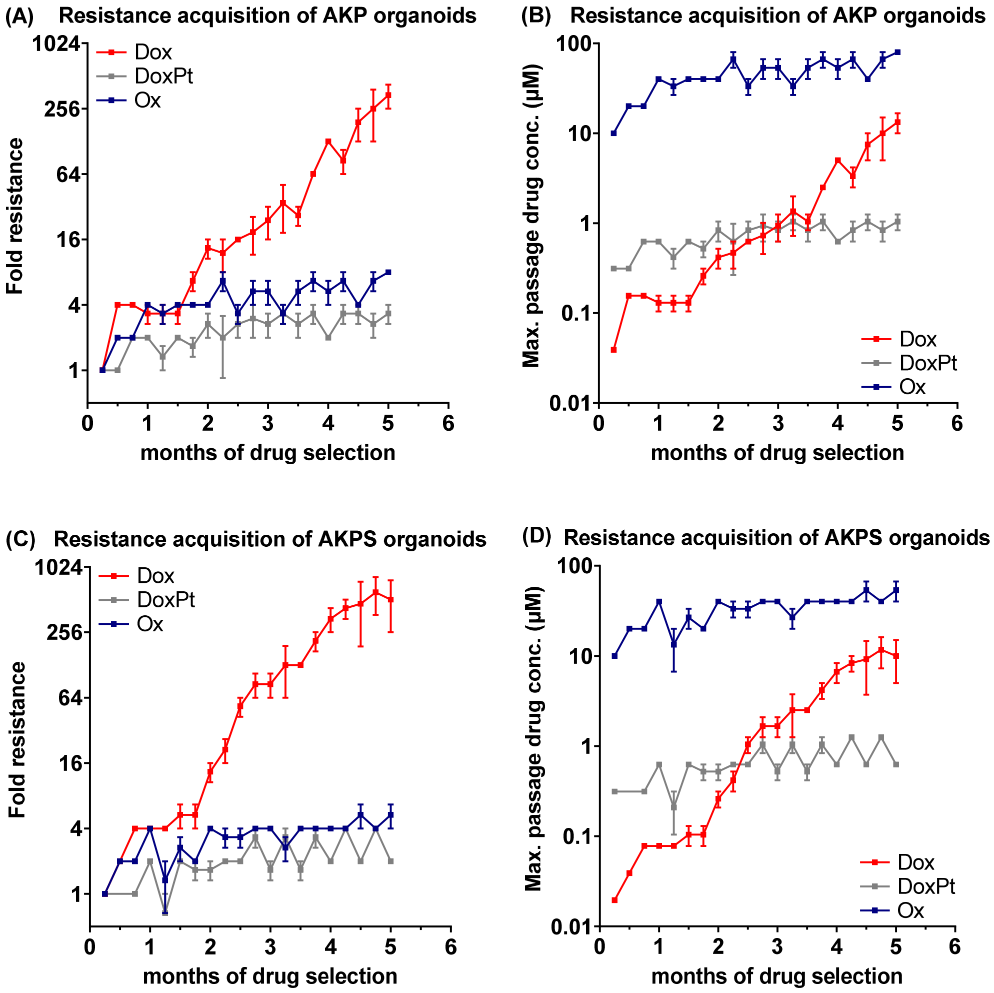

Figure S84. (A) Fold change in resistance of AKP murine colorectal cancer organoids during five months of drug selection (doxorubicin, red; doxaliplatin, gray; oxaliplatin, blue). (B) Highest drug concentration allowing for AKP organoid outgrowth across five months of drug selection. (C) Fold change in resistance of AKPS murine colorectal cancer organoids during five months of drug selection (doxorubicin, red; doxaliplatin, gray; oxaliplatin, blue). (D) Highest drug concentration allowing for AKPS organoid outgrowth across five months of drug selection. Error bars represent standard errors of the mean.

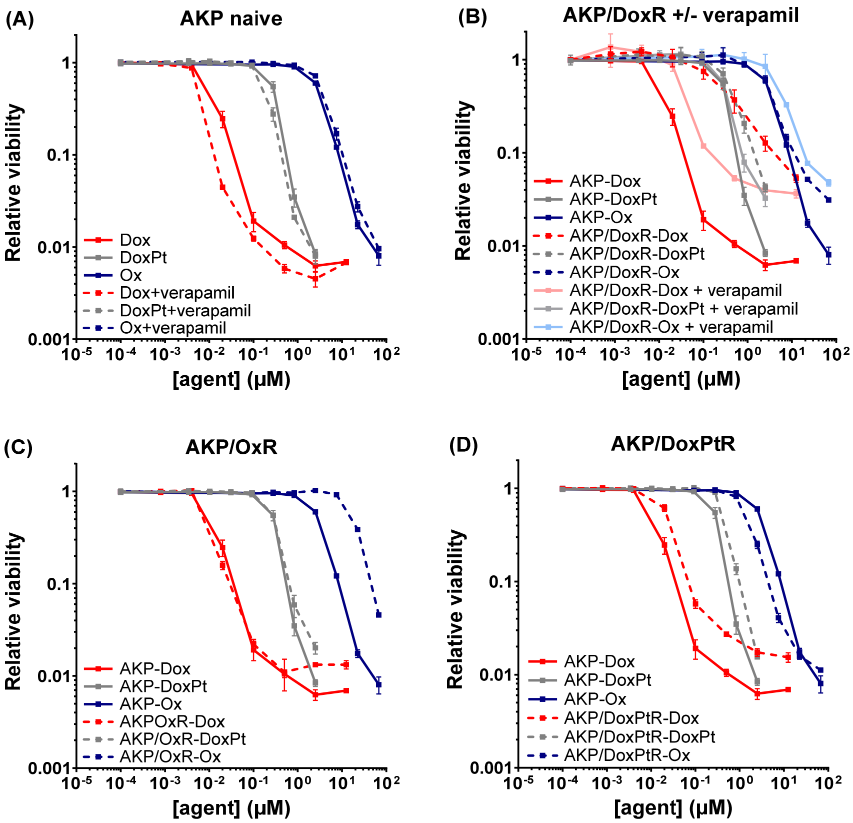

IC_{80} (μM)	AKP	AKP/DoxR	Fold Resistance
Dox	0.022±0.002	1.0±0.2	46
DoxPt	0.49±0.02	0.82±0.05	1.6
Ox	6.0±0.3	6.4±0.5	1.1

IC_{80} (μM)	AKP	AKP/OxR	Fold Resistance
Dox	0.022±0.002	0.019±0.000	0.8
DoxPt	0.49±0.02	0.48±0.01	1.0
Ox	6.0±0.3	32±1	5.3

IC_{80} (μM)	AKP	AKP/DoxPtR	Fold Resistance
Dox	0.022±0.002	0.046±0.004	2.1
DoxPt	0.49±0.02	0.73±0.02	1.5
Ox	6.0±0.3	2.9±0.1	0.50

IC_{80} (μM) with AKP	(−) verapamil	(+) verapamil (10 μM)	Fold Sensitization
Dox	0.022±0.002	0.011±0.000	2.0
DoxPt	0.49±0.02	0.32±0.01	1.6
Ox	6.0±0.3	7.0±0.3	0.86

IC_{80} (μM) with AKP/DoxR	(−) verapamil	(+) verapamil (10 μM)	Fold Sensitization
Dox	1.0±0.2	0.076±0.003	13.8
DoxPt	0.82±0.05	0.44±0.05	1.9
Ox	6.4±0.5	10±2	0.61

Figure S85. Dose-response curves showing the effect of oxaliplatin (Ox, blue), doxorubicin (Dox, red), and doxaliplatin (DoxPt, gray) on the viability of $APC^{-/-}$; $KRAS^{G12D/+}$; $p53^{-/-}$ (AKP) colorectal cancer organoid variants after a 72-hour exposure. (A) Dose-response curves for naïve AKP colorectal cancer organoids. (B) Dose-response curves for doxorubicin-resistant AKP/DoxR colorectal cancer organoids. (C) Dose-response curves for oxaliplatin-resistant AKP/OxR colorectal cancer organoids. (D) Dose-response curves for doxaliplatin-resistant AKP/DoxPtR colorectal cancer organoids. Error bars represent standard deviations.

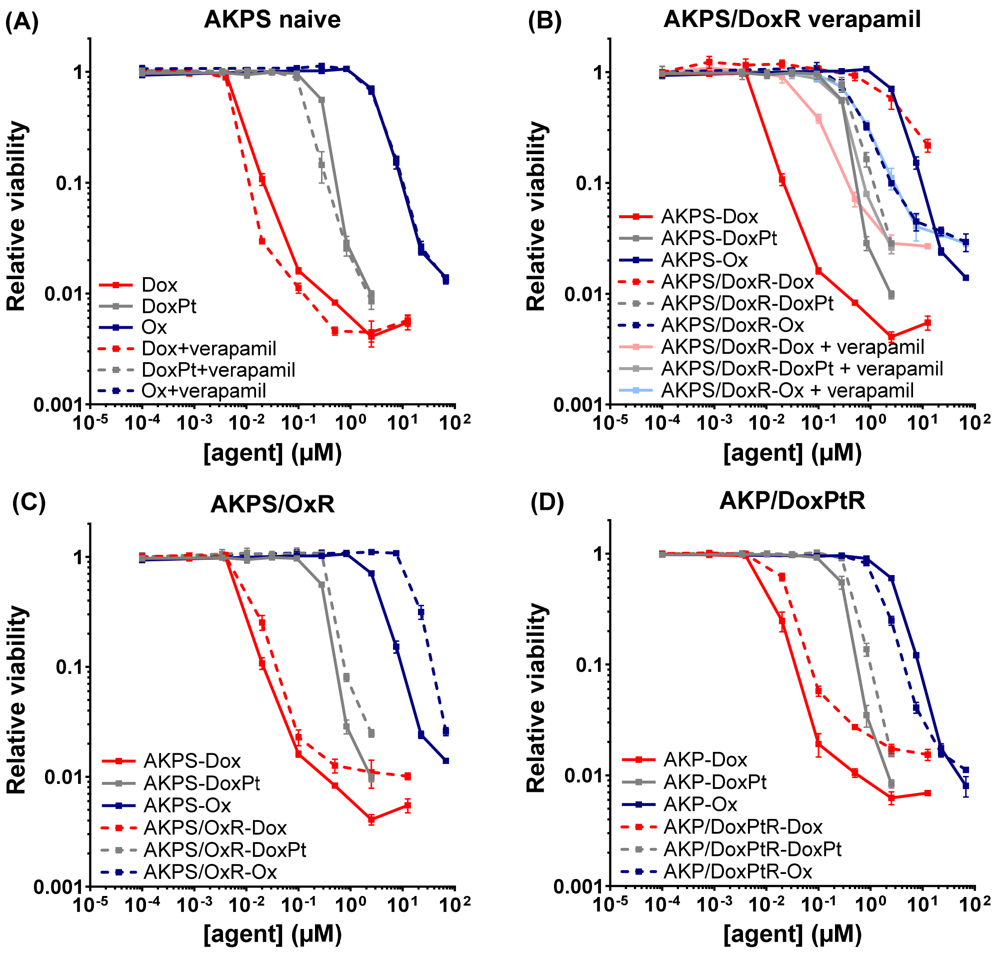

IC_{80} (μM)	AKPS	AKPS/DoxR	Fold Resistance
Dox	0.016±0.000	15±2	918
DoxPt	0.46±0.02	0.78±0.01	1.7
Ox	6.2±0.2	1.2±0.1	0.19

IC_{80} (μM)	AKPS	AKPS/OxR	Fold Resistance
Dox	0.016±0.000	0.031±0.003	1.9
DoxPt	0.46±0.02	0.68±0.01	1.5
Ox	6.2±0.2	33±2	5.3

IC_{80} (μM)	AKPS	AKPS/DoxPtR	Fold Resistance
Dox	0.016±0.000	0.040±0.004	2.4
DoxPt	0.46±0.02	0.90±0.06	2.0
Ox	6.2±0.2	5.2±0.0	0.84

IC_{80} (μM) with AKPS	(−) verapamil	(+) verapamil (10 μM)	Fold Sensitization
Dox	0.016±0.000	0.011±0.000	1.5
DoxPt	0.46±0.02	0.25±0.02	1.9
Ox	6.2±0.2	6.1±0.1	1.0

IC_{80} (μM) with AKPS/DoxR	(−) verapamil	(+) verapamil (10 μM)	Fold Sensitization
Dox	15±2	0.60±0.02	82
DoxPt	0.78±0.01	0.18±0.01	1.3
Ox	1.2±0.1	1.4±0.0	0.98

Figure S86. Dose-response curves showing the effect of oxaliplatin (Ox, blue), doxorubicin (Dox, red), and doxaliplatin (DoxPt, gray) on the viability of $APC^{-/-}$; $KRAS^{G12D}$; $P53^{-/-}$; $SMAD4^{-/-}$ (AKPS) colorectal cancer organoid variants after a 72-hour exposure. (A) Dose-response curves for naïve AKPS colorectal cancer organoids. (B) Dose-response curves for doxorubicin-resistant AKPS/DoxR colorectal cancer organoids. (C) Dose-response curves for oxaliplatin-resistant

AKPS/OxR colorectal cancer organoids. (D) Dose-response curves for doxaliplatin-resistant AKPS-DoxPtR colorectal cancer organoids. Error bars represent standard deviations.

S9. Imaging studies.

Experimental procedure

Imaging was performed using a Nikon Eclipse Ti-S/L100 inverted phase contrast fluorescent microscope fitted with an Andor Zyla sCMOS VCS digital camera. The light source was a Lumencor SOLA Light Engine solid-state illumination source (SOLA-SMS-LCR-SA). Nikon DAPI filter set (excitation BP 405/20 nm; emission BP 460/50 nm) was used to visualize Hoechst-33342. Nikon TRITC filter set (excitation BP 545/30 nm; emission BP 620/60 nm) was used to visualize doxorubicin and DoxPt. The microscope was operated with Nikon NIS Elements (5.02.01, build 1270).

Regions of interest (ROI) were identified using phase contrast microscopy. To measure the accumulation and localization of doxorubicin or DoxPt, fluorescence images were acquired using the TRITC standard red channel filter set with a 2000 ms exposure time at gain level 4. To locate the Hoechst-33342-stained nuclear region, fluorescence images were acquired using the DAPI standard blue channel filter set. All images were acquired with a 40× objective lens.

In brief, MES-SA or MES-SA/Dx5 cells were seeded onto 35 mm glass bottom dish with 20 mm micro-well #1.5 cover glass (Cellvis catalog # D35-20-1.5-N). The cells were cultured until reaching 70% confluency. The media were replaced with media containing doxorubicin (2.5 μM) or doxaliplatin (2.5 μM). For conditions where verapamil was co-administered, it was applied at 10 μM along with the cytotoxic agent. The cells were treated for 18 h. The media were then replaced with FluoroBrite^{TM} DMEM (ThermoFisher catalog # A1896701) containing Hoechst-33342 (2 μg/mL). The cells were incubated for 30 min before imaging. The fluorescence intensities in whole cell, cytoplasm, and nucleus were quantified using ImageJ (version 1.50i). The background fluorescence of each image was calculated by averaging the fluorescence intensity of five approximately cell-sized regions, in which cells or debris were absent. This value was then used to correct the fluorescence intensity measured for the whole cell, cytoplasm, and nucleus in the corresponding image. For all experiments, the quantification was accomplished based on a minimum number of 130 cells.

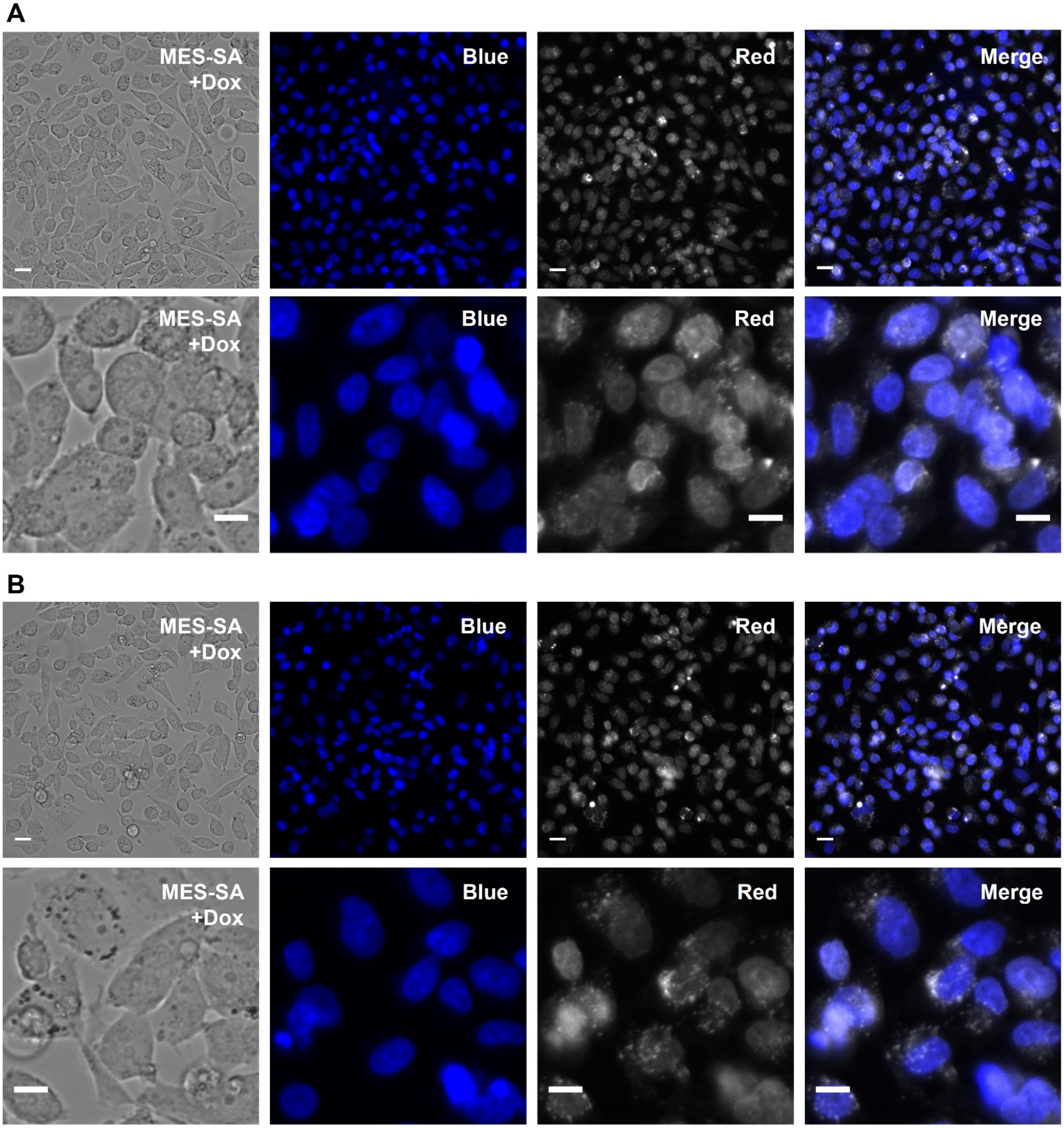

Figure S87. Images from fluorescence microscopy studies showing drug accumulation and localization in MES-SA cells treated with doxorubicin (Dox, 2.5 μM) for 18 h. Image Sets A and B are two representative regions. For each set of images, the top row shows the microscope field of view (scale bar = 20 μm) and the bottom row shows the expanded view of a selected region of the image (scale bar = 10 μm).

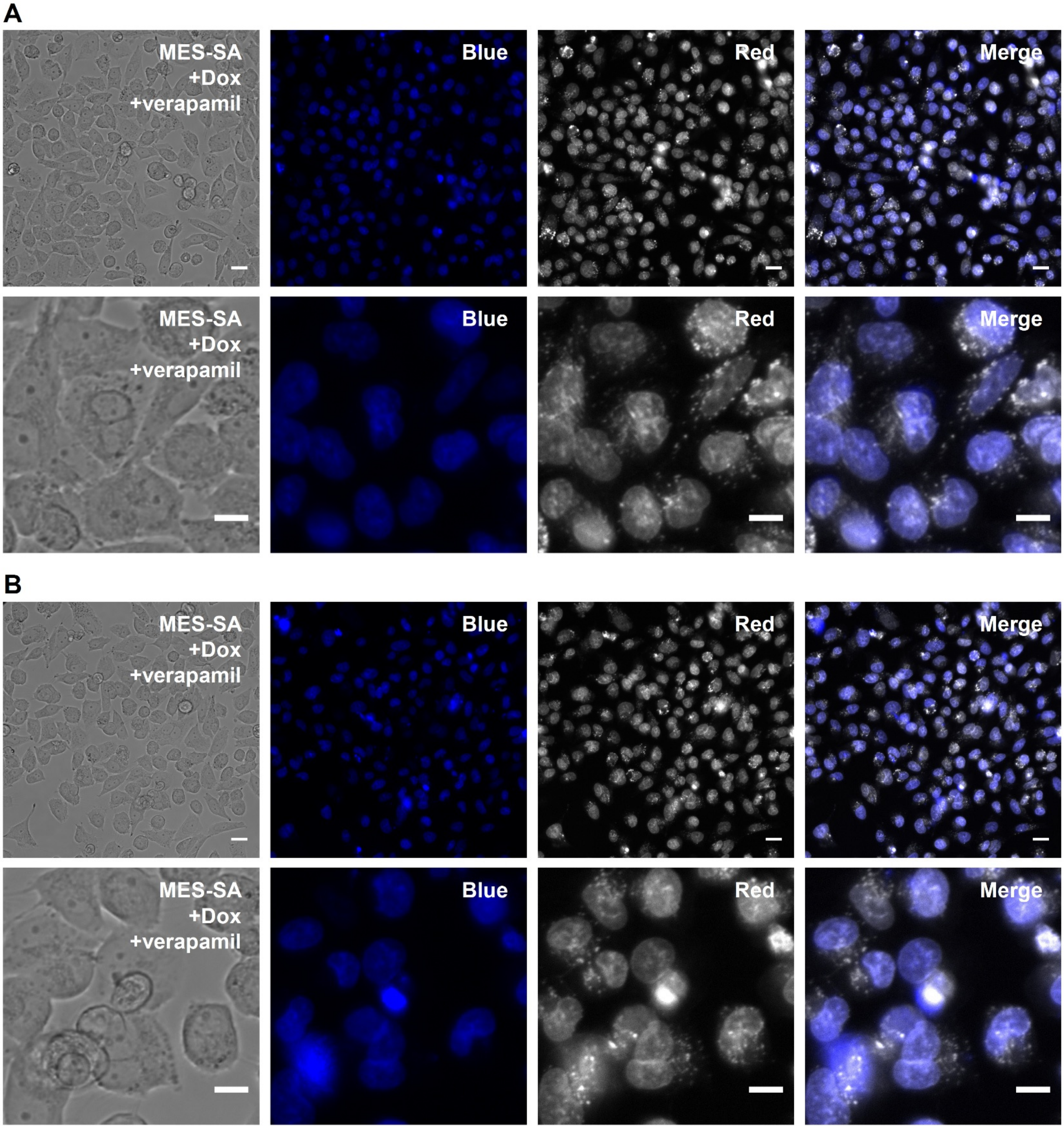

Figure S88. Images from fluorescence microscopy studies showing drug accumulation and localization in MES-SA cells treated with doxorubicin (Dox, 2.5 μM) in the presence of verapamil (10 μM) for 18 h. Image Sets A and B are two representative regions. For each set of images, the top row shows the microscope field of view (scale bar = 20 μm) and the bottom row shows the expanded view of a selected region of the image (scale bar = 10 μm).

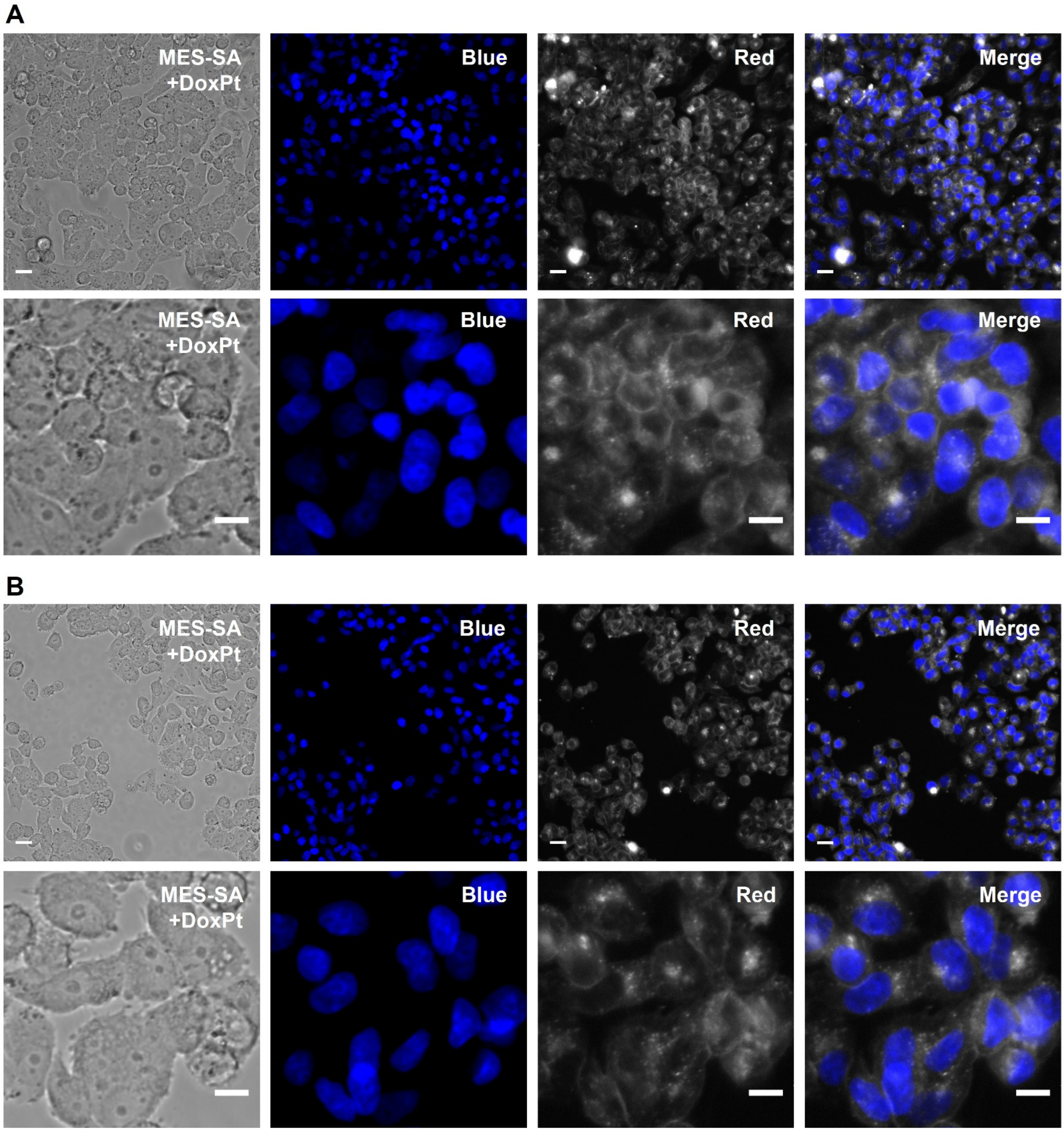

Figure S89. Images from fluorescence microscopy studies showing drug accumulation and localization in MES-SA cells treated with doxaliplatin (DoxPt, 2.5 μM) for 18 h. Image Sets A and B are two representative regions. For each set of images, the top row shows the microscope field of view (scale bar = 20 μm) and the bottom row shows the expanded view of a selected region of the image (scale bar = 10 μm).

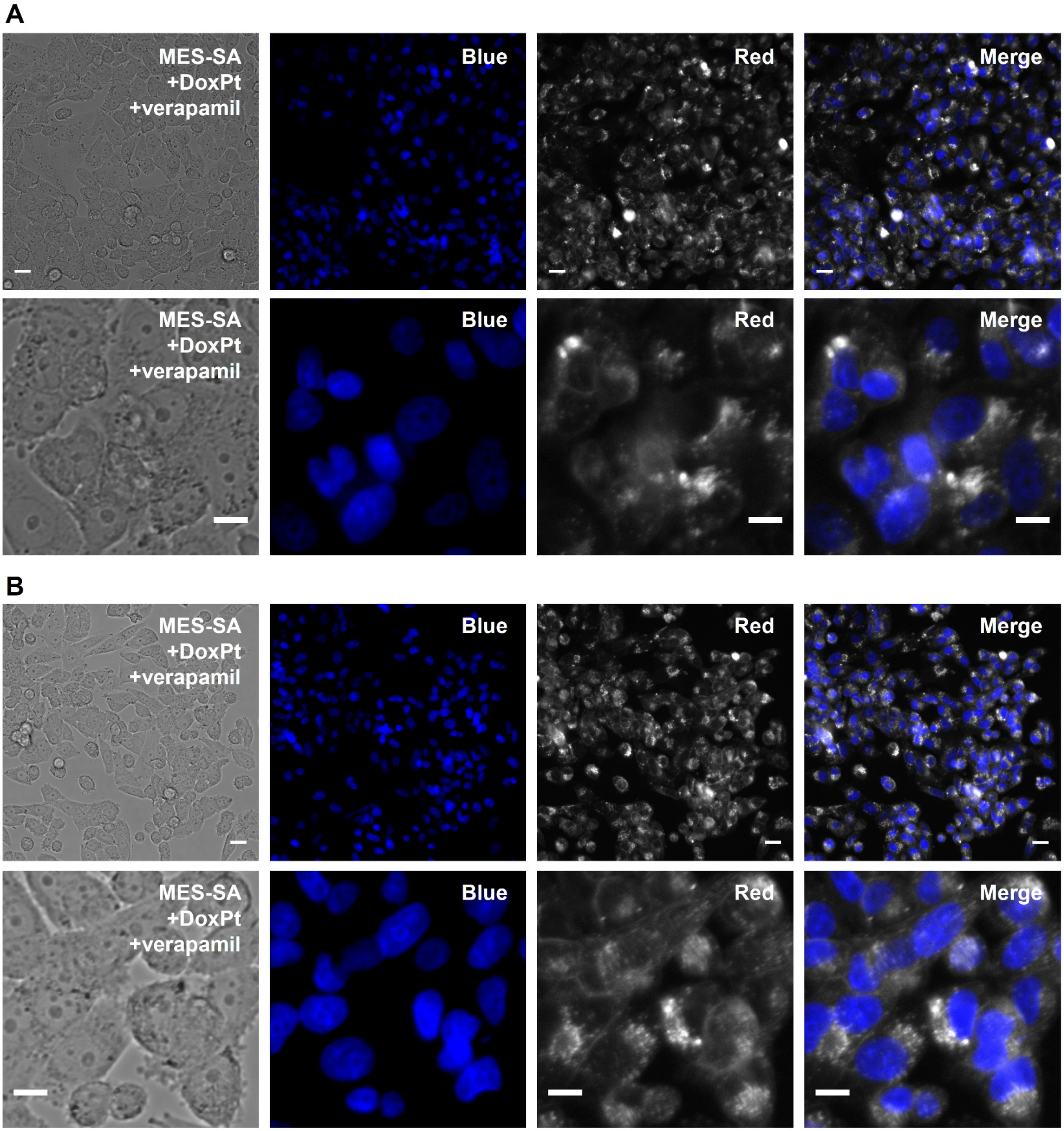

Figure S90. Images from fluorescence microscopy studies showing drug accumulation and localization in MES-SA cells treated with doxaliplatin (DoxPt, 2.5 μM) in the presence of verapamil (10 μM) for 18 h. Image Sets A and B are two representative regions. For each set of images, the top row shows the microscope field of view (scale bar = 20 μm) and the bottom row shows the expanded view of a selected region of the image (scale bar = 10 μm).

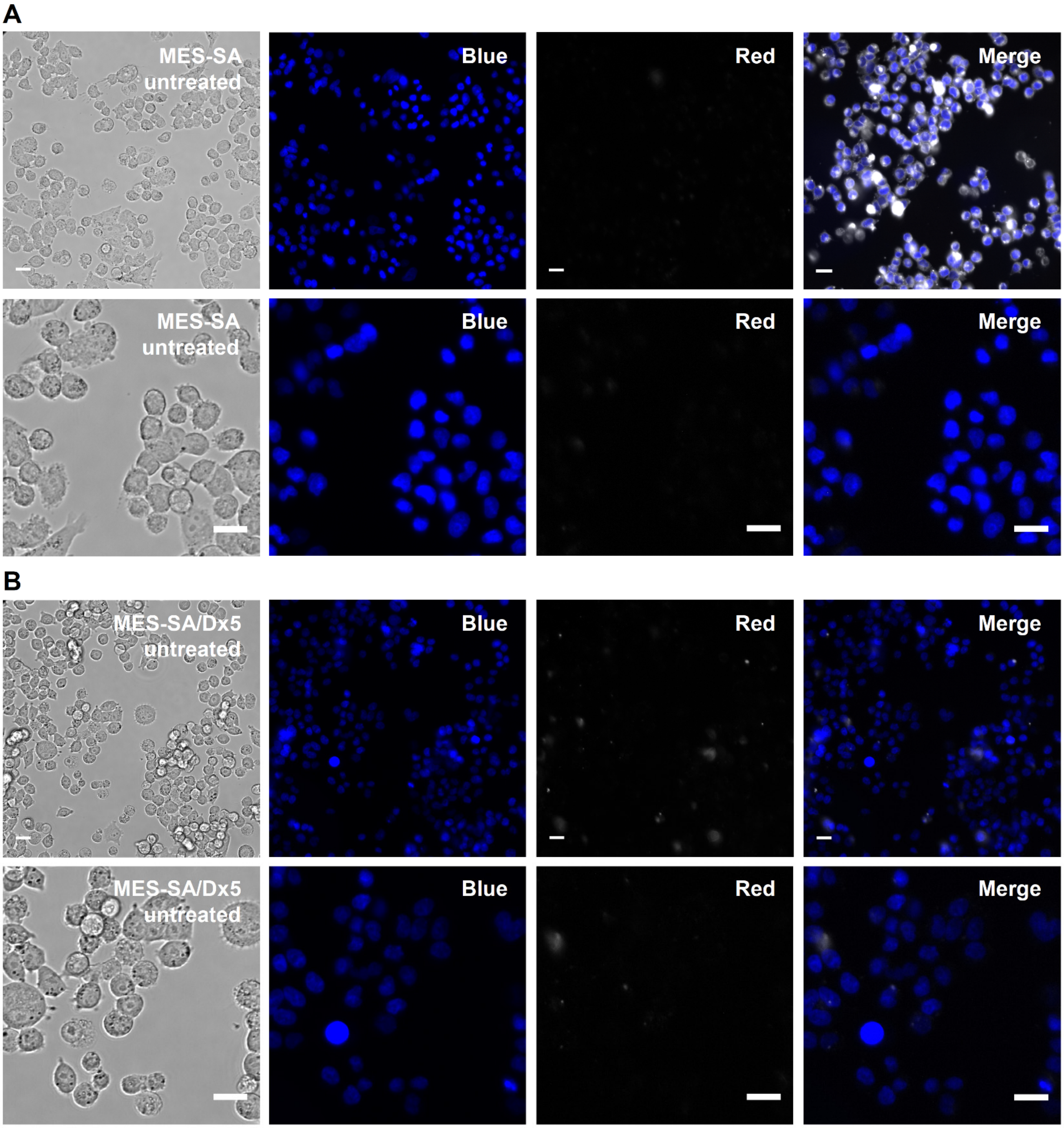

Figure S91. Images from fluorescence microscopy studies of untreated MES-SA and MES-SA/Dx5 cells. Image Sets A and B are representative regions for MES-SA and MES-SA/Dx5 cells, respectively. For each set of images, the top row shows the microscope field of view (scale bar = 20 μm) and the bottom row shows the expanded view of a selected region of the image (scale bar = 10 μm).

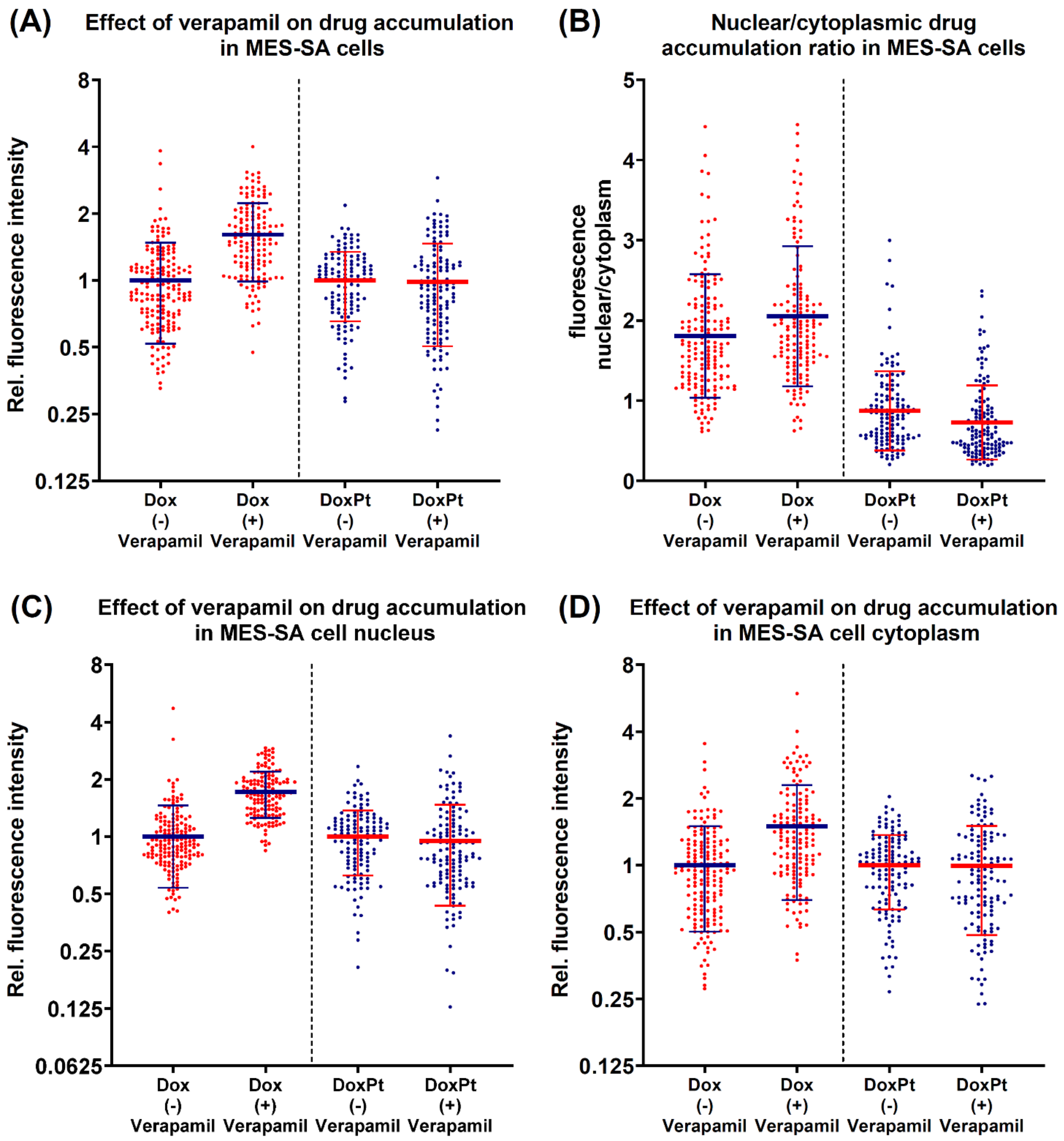

Figure S92. (A) Quantification of fluorescence intensity of MES-SA cells treated with doxorubicin (Dox, 2.5 µM) or doxaliplatin (DoxPt 2.5 µM) for 18 h in the absence or presence of verapamil. Fluorescence intensity is normalized to the average absolute fluorescence intensity of cells treated with the respective drug in the absence of verapamil. The results are consistent across three independent experiments. One experiment was chosen for quantification (n = 169 cells for Dox (−) verapamil, n = 148 cells for Dox (+) verapamil, n = 127 cells for DoxPt (−) verapamil, n = 133 cells for Dox (+) verapamil). (B) Ratio of nuclear and cytoplasmatic fluorescence intensity of MES-SA cells treated with Dox (2.5 µM) or DoxPt (2.5 µM) for 18 h in the absence or presence of verapamil. (n = 169 cells for Dox (−) verapamil, n = 148 cells for Dox (+) verapamil, n = 127 cells for DoxPt (−) verapamil, n = 133 cells for Dox (+) verapamil). (C) Relative nuclear fluorescence intensity of MES-SA cells treated with Dox (2.5 µM) or DoxPt (2.5 µM) for 18 h in the absence or presence of verapamil. Fluorescence intensity is normalized to the average absolute nuclear fluorescence intensity of cells treated with the respective drug in the absence of verapamil. (n = 169 cells for Dox (−) verapamil, n = 148 cells for Dox (+) verapamil, n = 127 cells for DoxPt (−) verapamil, n = 133 cells for Dox (+) verapamil). (D) Relative cytoplasmatic fluorescence intensity of MES-SA cells treated with Dox (2.5 µM) or DoxPt (2.5 µM) for 18 h in the absence or presence of verapamil. (n = 169 cells for Dox (−) verapamil, n = 148 cells for Dox (+) verapamil, n = 127 cells for DoxPt (−) verapamil, n = 133 cells for Dox (+) verapamil). Fluorescence intensity is normalized to the average absolute cytoplasmatic fluorescence intensity of cells treated with the respective drug in the absence of verapamil. Error bars represent standard deviations.

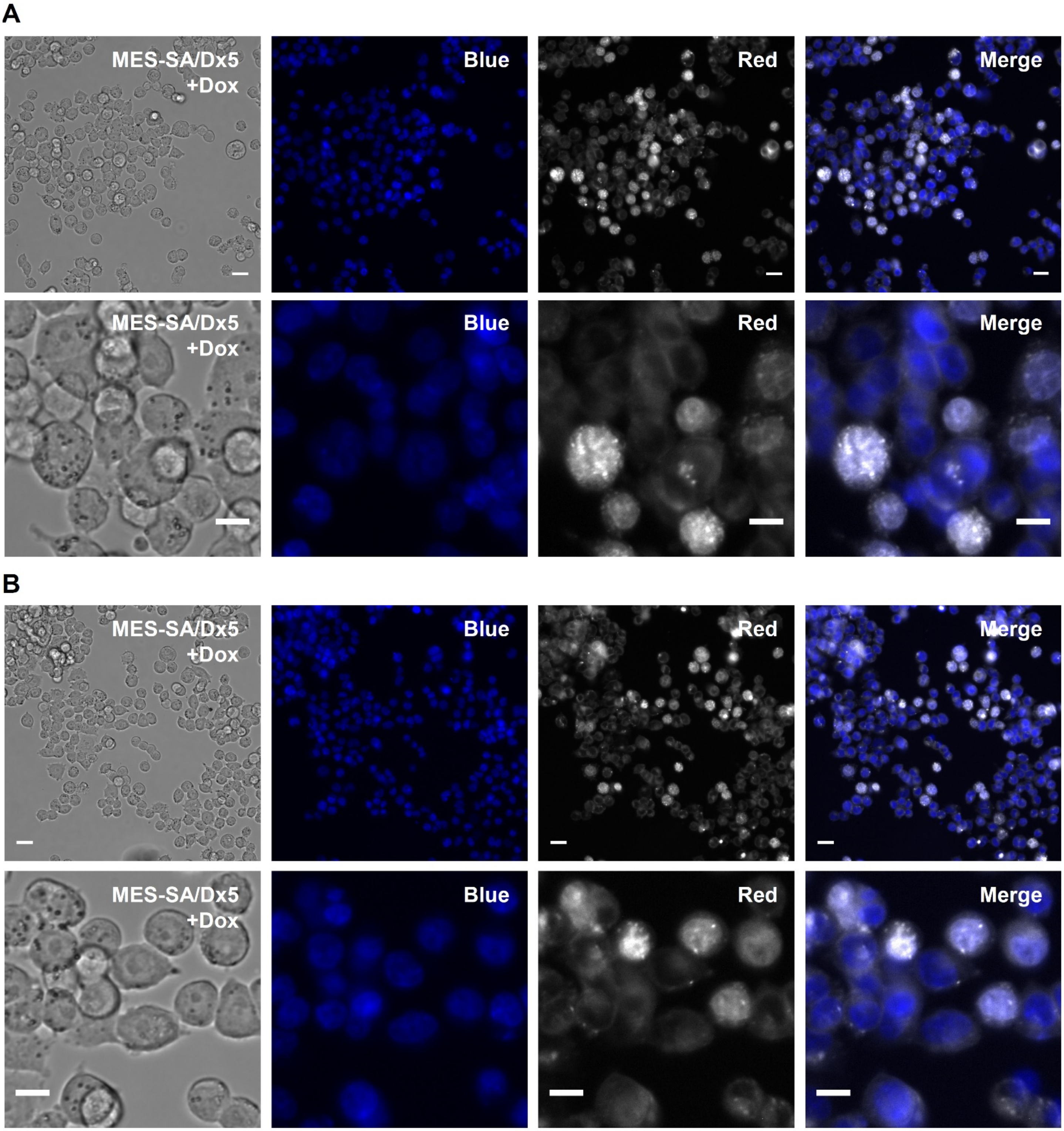

Figure S93. Images from fluorescence microscopy studies showing drug accumulation and localization in MES-SA/Dx5 cells treated with doxorubicin (Dox, 2.5 µM) for 18 h. Image Sets A and B are two representative regions. For each set of images, the top row shows the microscope field of view (scale bar = 20 µm) and the bottom row shows the expanded view of a selected region of the image (scale bar = 10 µm).

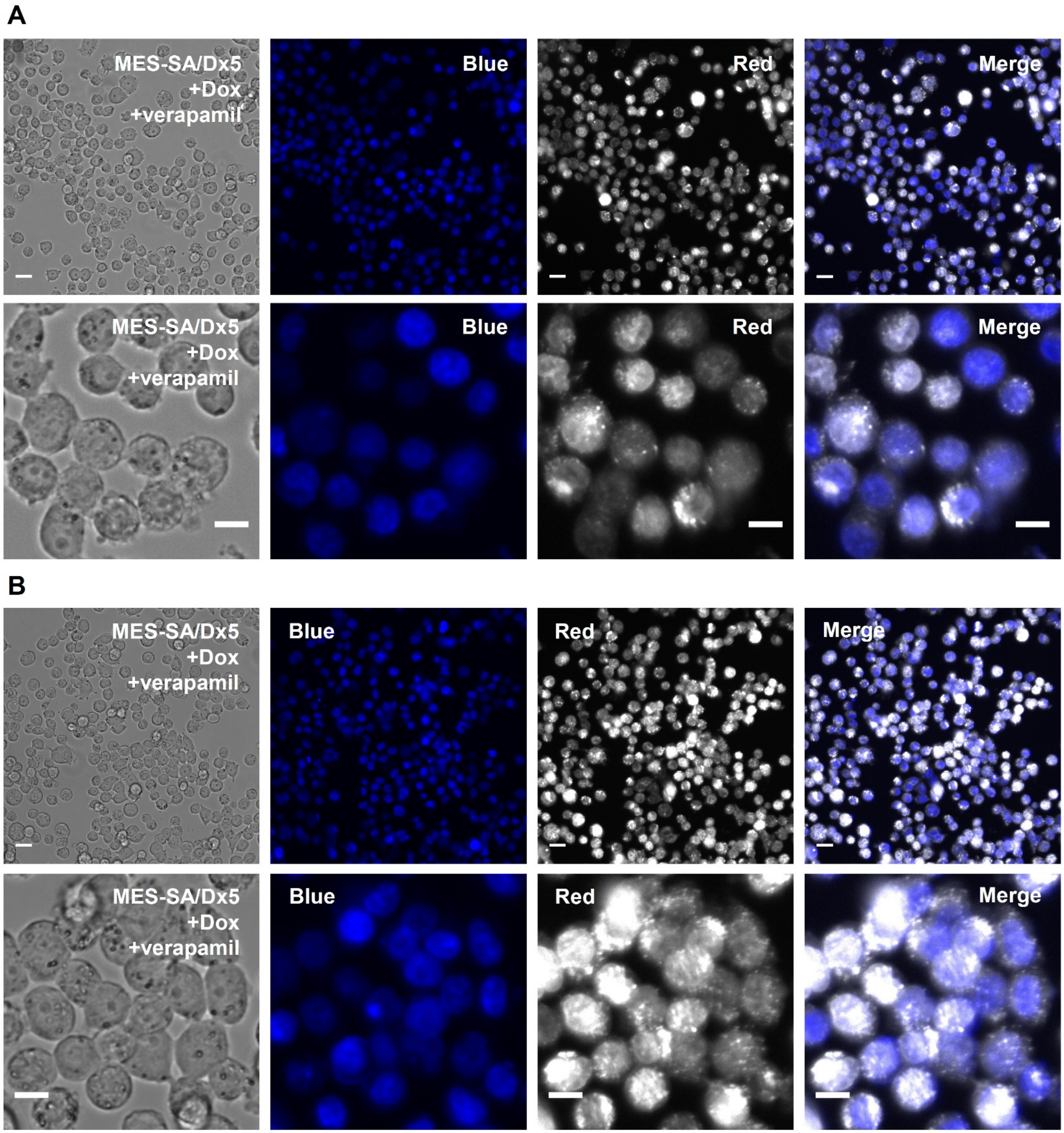

Figure S94. Images from fluorescence microscopy studies showing drug accumulation and localization in MES-SA/Dx5 cells treated with doxorubicin (Dox, 2.5 μM) in the presence of verapamil (10 μM) for 18 h. Image Sets A and B are two representative regions. For each set of images, the top row shows the microscope field of view (scale bar = 20 μm) and the bottom row shows the expanded view of a selected region of the image (scale bar = 10 μm).

A

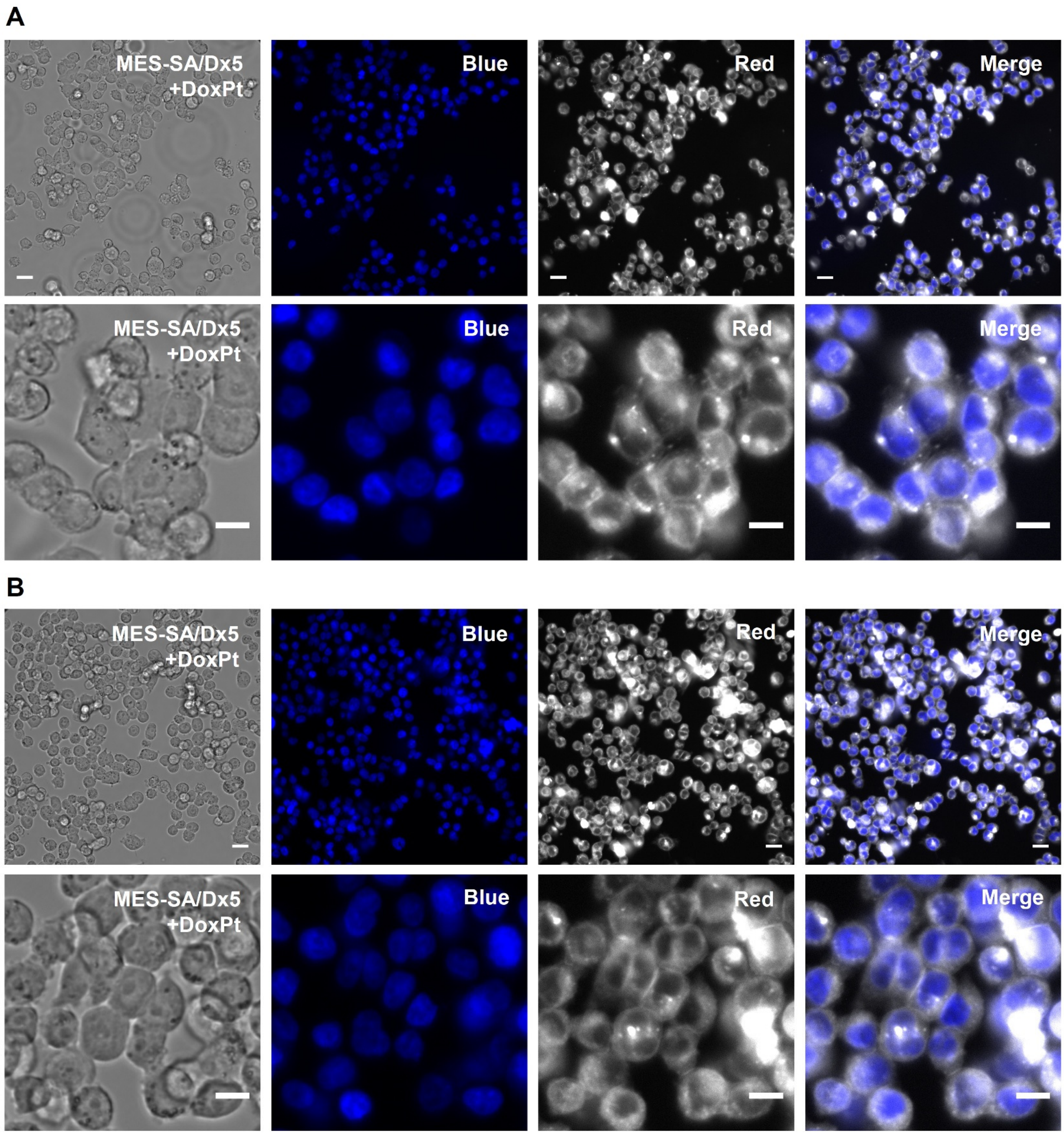

Figure S95. Images from fluorescence microscopy studies showing drug accumulation and localization in MES-SA/Dx5 cells treated with doxaliplatin (DoxPt, 2.5 μM) for 18 h. Image Sets A and B are two representative regions. For each set of images, the top row shows the microscope field of view (scale bar = 20 μm) and the bottom row shows the expanded view of a selected region of the image (scale bar = 10 μm).

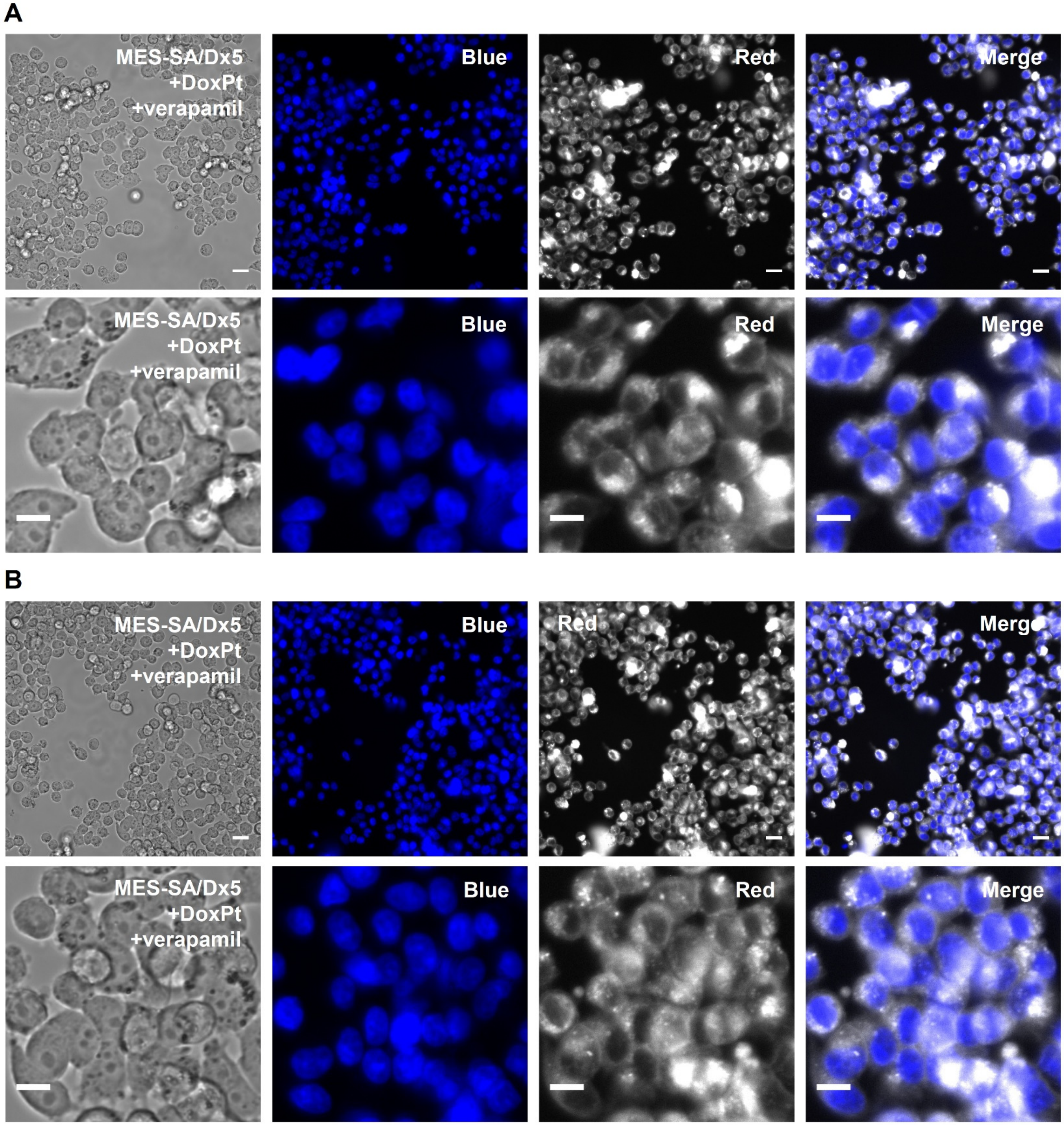

Figure S96. Images from fluorescence microscopy studies showing drug accumulation and localization in MES-SA/Dx5 cells treated with doxaliplatin (DoxPt, 2.5 μM) in the presence of verapamil (10 μM) for 18 h. Image Sets A and B are two representative regions. For each set of images, the top row shows the microscope field of view (scale bar = 20 μm) and the bottom row shows the expanded view of a selected region of the image (scale bar = 10 μm).

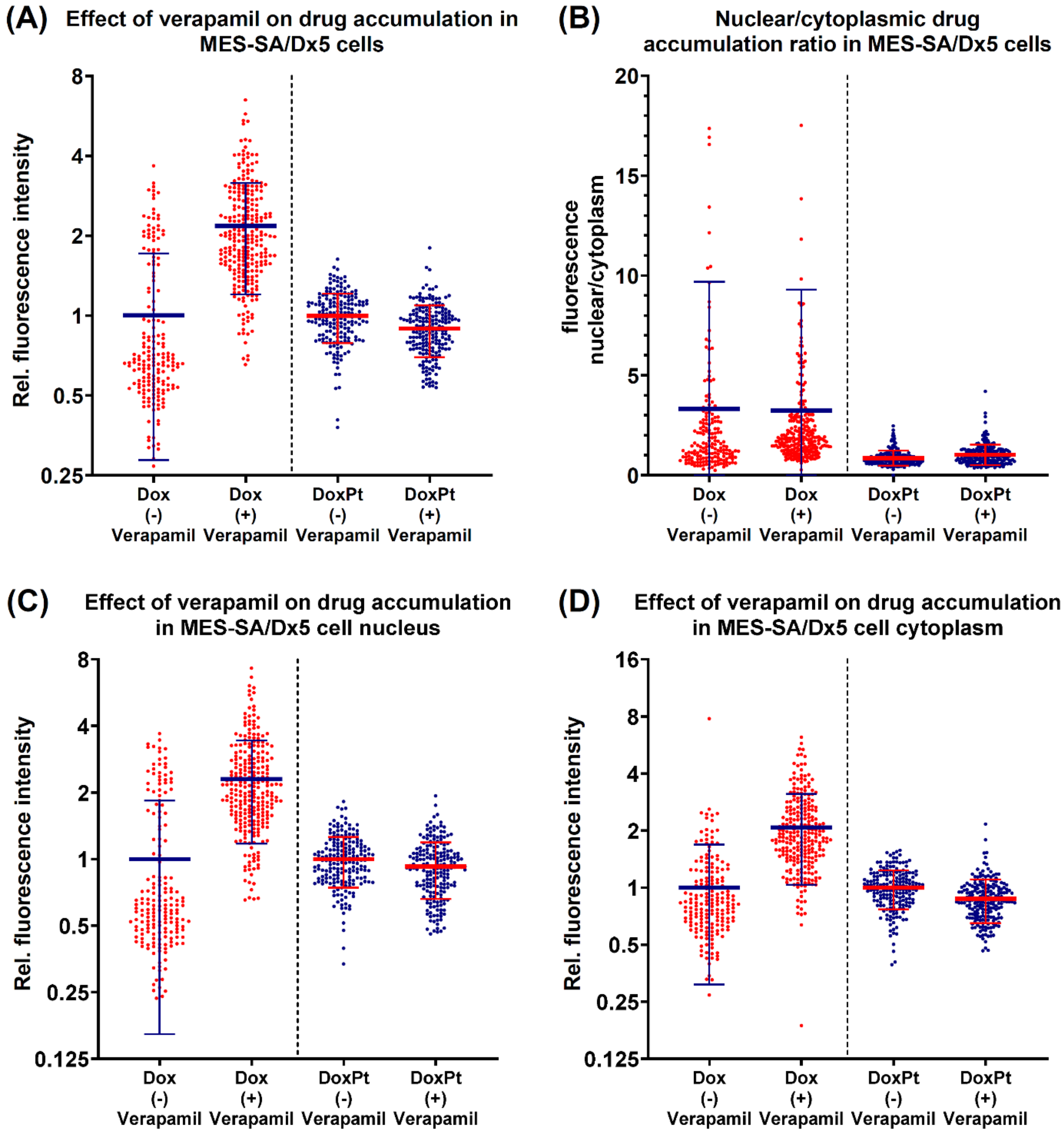

Figure S97. (A) Quantification of fluorescence intensity of MES-SA/Dx5 cells treated with doxorubicin (Dox, 2.5 μM) or doxaliplatin (DoxPt, 2.5 μM) for 18 h in the absence or presence of verapamil. Fluorescence intensity is normalized to the average absolute fluorescence intensity of cells treated with the respective drug in the absence of verapamil. The results are consistent across three independent experiments. One experiment was chosen for quantification (n = 183 cells for Dox (−) verapamil, n = 262 cells for Dox (+) verapamil, n = 192 cells for DoxPt (−) verapamil, n = 205 cells for Dox (+) verapamil). (B) Ratio of nuclear and cytoplasmatic fluorescence intensity of MES-SA/Dx5 cells treated with Dox (2.5 μM) or DoxPt (2.5 μM) for 18 h in the absence or presence of verapamil. (n = 183 cells for Dox (−) verapamil, n = 261 cells for Dox (+) verapamil, n = 192 cells for DoxPt (−) verapamil, n = 205 cells for Dox (+) verapamil). (C) Relative nuclear fluorescence intensity of MES-SA/Dx5 cells treated with Dox (2.5 μM) or DoxPt (2.5 μM) for 18 h in the absence or presence of verapamil. (n = 183 cells for Dox (−) verapamil, n = 262 cells for Dox (+) verapamil, n = 192 cells for DoxPt (−) verapamil, n = 205 cells for Dox (+) verapamil). Fluorescence intensity is normalized to the average absolute nuclear Fluorescence intensity of cells treated with the respective drug in the absence of verapamil. (D) Relative cytoplasmatic fluorescence intensity of MES-SA/Dx5 cells treated with Dox (2.5 μM) or DoxPt (2.5 μM) for 18 h in the absence or presence of verapamil. (n = 183 cells for Dox (−) verapamil, n = 261 cells for Dox (+) verapamil, n = 192 cells for DoxPt (−) verapamil, n = 205 cells for Dox (+) verapamil). Fluorescence intensity is normalized to the average absolute cytoplasmatic fluorescence intensity of cells treated with the respective drug in the absence of verapamil. Error bars represent standard deviations.

S10. Characterization of DoxPt with RNAi-based competition assay.

A panel of eight previously validated shRNAs[9] (Table S2) were delivered to Eμ-Myc Cdkn2a$^{Arf-/-}$ lymphoma cells using the pMSCV-LTR-miR30-SV40-GFP (MLS) retroviral vector.[10,11] The lymphoma cells were grown in B-cell media (BCM), which was composed of Dulbecco's Modification of Eagle's Medium (DMEM, Corning catalog # 45000-306) and Iscove's Modification of DMEM (Corning catalog # 45000-366) supplemented with 10% fetal bovine serum (Gibco catalog # A2720803), 1% penicillin/streptomycin (Corning catalog # 45000-652), and 0.1% 2-mercaptoethanol (Gibco catalog # 21985023). The cells were infected with each of the GFP-tagged shRNA constructs at 25-30% infection rate as previously described.[48] Cells expressing each shRNA were seeded into 24-well plates with 250 μL BCM (125,000 cells/well for treatment wells and 65,000 cells/well for control wells). Drug-containing media (250 μL) was then added to the cells. For control wells, 300 μL of media was removed and replaced with 300 μL of fresh BCM media after 24 h. An additional 500 μL of fresh BCM media was then added to all wells. After 48 h of drug treatment, the GFP percentage of treatment and control wells was determined by flow cytometry using a BD FACSCelesta™ cell analyzer, with live cells quantified based on DAPI exclusion. The experiment was performed independently three times, each time with three technical replicates. For each drug, the resistance index (RI) was calculated for cells expressing each shRNA as previously defined.[9] The RI values for generating the signature of each treatment in the heat map (Fig. 3k) are listed in Table S3. Subsequently, each signature was compared to an established reference set of drugs (Table S4) using the modified K-nearest neighbors (K-NN) algorithm with reported parameters and code.[9,12] This algorithm classifies the drug of interest into the nearest category in the reference set using Euclidean K-NN analysis. The linkage ratio (LR) is then calculated by dividing the pairwise distances of the new drug-containing category by the category without the new drug. Next, the LR values for all out-of-category drugs are calculated assuming that these drugs are members of that specific category, which provides a background distribution of negative-control LRs. Lastly, the p value was obtained by comparing the LR of the drug of interest to the distribution of negative-control LRs. When the p value is greater than 0.05, the drug of interest is considered to belong to a "new drug class" with a mechanism of action not represented in the reference set.[45] Additionally, principle component analysis (PCA) using the "pca" function in MATLAB was also performed to visualize the drug classification (Table S4, Figure S98, and Figure S99).[13]

The LR analysis indicated that DoxPt may kill cancer cells through a mode of action distinct from that of either Dox or Ox. Additionally, DoxPt was classified separately from any cytotoxic agents present in the reference set (p value = 0.4732). A signature for the physical mixture of equipotent doses of Dox and Ox was also generated. The Dox-Ox mixture also exhibited a signature differing from the categories available from the reference set (p value = 0.1425, LR = 1.1). Although the signatures of DoxPt are similar to those of the Dox-Ox physical mixture (Fig. 3k), statistically significant differences in the RIs of these two treatments indicate that DoxPt may be mechanistically distinct from the physical mixture of Dox and Ox (Figure S99).

Table S2. Target sequence of employed shRNAs.

shRNA	Target sequence
p53	CCACTACAAGTACATGTGTAA
Chk2	CAGAAACACATAATCATTAAA
ATR	ACCCATGTTCTTGACATTGAA
Chk1	CAGGAATATTCTGATTGGAAA
ATX	CAGGATAGCAATAAAGATGAA
DNAPK	CAGGCCTATACTTACAGTTAA
Bok	CTGGCCTCTGTGACTGCTCTA
Bim	TAGGAACAGAGAAATATGCAA

Table S3. Resistance index (RI) of drugs with Eμ-Myc Cdkn2aArf$^{-/-}$ lymphoma cells bearing different shRNAs.

Treatment	p53	Chk2	ATR	Chk1	ATX	DNAPK	Bok	Bim
DoxPt	1.932	2.278	0.794	−0.708	−1.366	0.745	−0.965	1.146
Dox	2.563	2.354	0.980	−0.711	−1.416	−0.745	−2.022	1.621
Ox	2.325	1.363	−0.122	0.656	−0.909	−0.165	1.017	0.631
Dox+Ox	2.722	2.239	0.372	0.444	−1.228	−0.448	0.025	1.180
Etoposide	3.105	3.510	0.754	−0.894	−1.532	−0.352	−0.840	1.582
Epirubicin	4.003	3.884	1.434	−0.611	−1.342	−1.529	−1.442	1.486
Daunuribicin	3.982	3.773	1.158	−1.246	−1.170	−1.487	−1.964	1.664
Mitoxantrone	3.938	3.581	1.403	−0.672	−1.778	−1.571	−0.847	1.295
Rapamycin	2.194	0.033	0.188	−1.029	−1.518	−0.007	1.368	1.257
Flavopiridol	2.995	0.065	0.592	−0.585	−1.419	0.252	−0.097	1.164
BMH-21	2.980	−0.139	0.780	−0.587	−0.954	−0.081	−0.429	1.556
Actinomycin D	3.094	0.361	0.976	-0.197	−1.619	0.395	0.160	1.079
Gemcitabine	1.678	0.962	−0.886	−1.460	0.660	1.010	0.917	0.856
Pemetrexed	1.748	1.101	−0.006	−1.605	0.035	0.137	0.367	1.053
Methotrexate	1.345	0.875	−0.829	−2.229	0.899	−0.172	−0.901	0.909
5-Fluorouracil	2.430	0.964	−0.348	−2.036	0.247	0.613	1.506	1.259
Hydroxyurea	3.218	−0.009	−0.250	−2.375	−0.131	0.809	−0.115	0.796

Table S4. Reference set drugs and classes.

Category	Drug	Category	Drug	Category	Drug
Top2 poisons	Doxorubicin	Single-strand alkylating agents	Lomustine	Transcription–translation inhibitors	Oxaliplatin
	Etoposide		Carmustine		Rapamycin
	Epirubicin		Temozolomide		Flavopiridol
	Daunorubicin		Streptozocin		BMH-21
	Mitoxantrone	Anti-metabolites	Gemcitabine		Actinomycin D
DNA cross-linkers	Chlorambucil		Pemetrexed	HDAC inhibitors	Scriptaid
	Satraplatin		Methothrexate		SAHA
	Carboplatin		5-Fluorouracil		JQ1
	Thiotepa		Hydroxyurea		LBH589
	Mitomycin C	Spindle Destabilizers	Vinblastine	DNMT inhibitors	Zebularine
Top1 poisons	Camptothecin		Vinorelbine		5-Aza-cytidine
	Irinotecan		Vincristine		Decitabine
	SN-38		Colchicine	HSP90 inhibitors	17-AAG
	Topotecan	Spindle stabilizer	Docetaxel		VER-50589
PARP inhibitors	BMN-673		Cabazitaxel		
	Veliparib		Taxol		
	Olaparib				

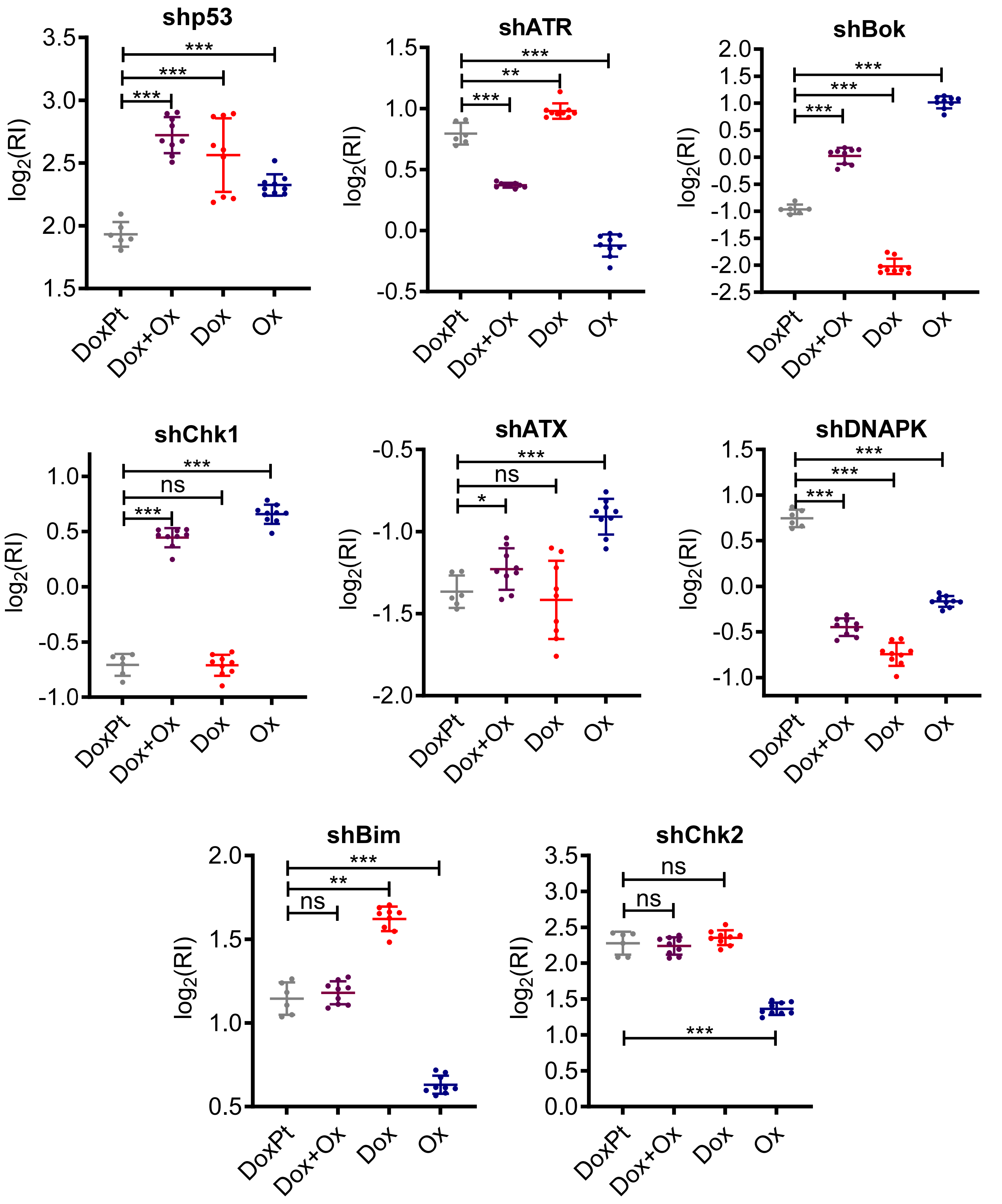

Figure S98. Comparison of resistance indexes (RIs) for Eμ-Myc Cdkn2aArf$^{-/-}$ cells infected with each shRNA and treated with either doxaliplatin (DoxPt) or an equipotent doxorubicin (Dox) and oxaliplatin (Ox) physical mixture. Welch's *t* test results are annotated as: *** for $p < 0.001$, ** for $0.001 < p < 0.01$, * for $p = 0.035$, and ns for not significant. The data suggest that, statistically, the mode of action of DoxPt is significantly different from those of Dox, Ox, and Dox+Ox in pathways involving p53, ATR, Bok, and DNAPK. DoxPt exhibits activity profiles only partially overlapping with those of Dox or Dox+Ox, as indicated by the ns difference with Chk1, ATX, Bim, and Chk2. Overall, DoxPt is classified separately from these reagents. Error bars represent standard deviations.

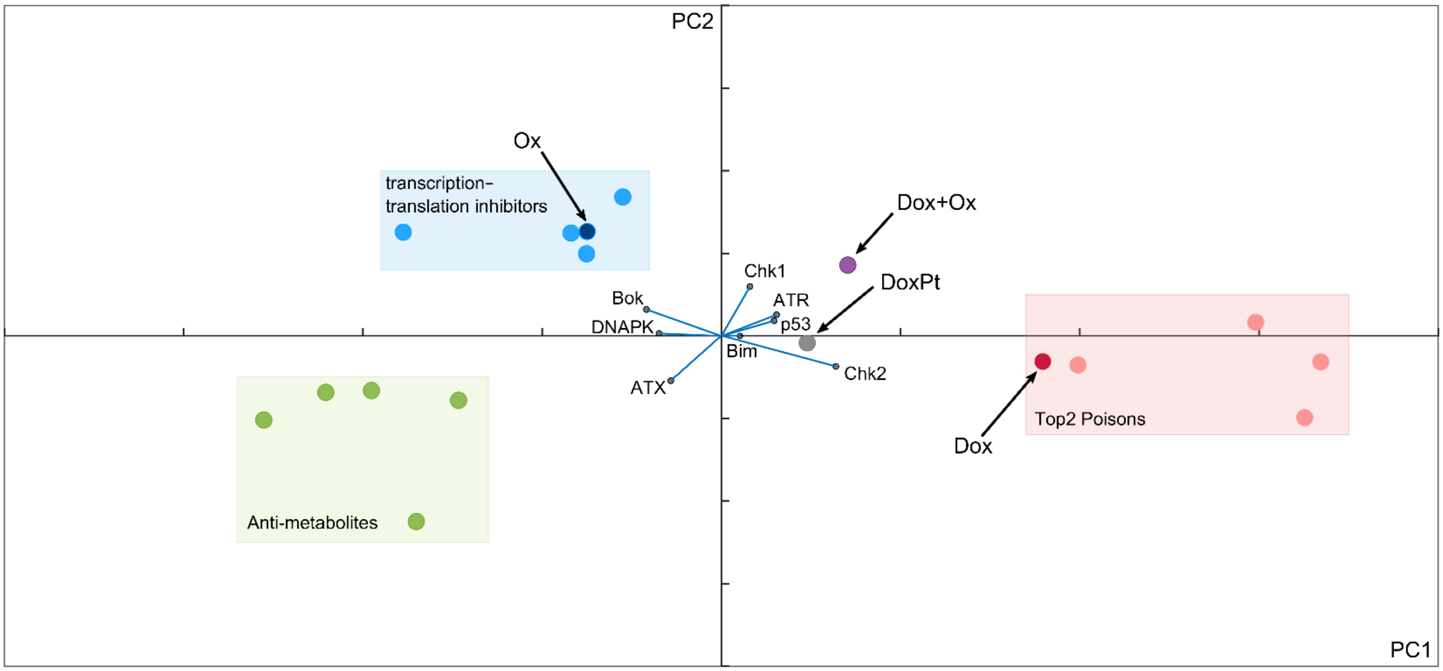

Principle Component	1	2
% variance explained	61.2	17.4

Principle Component 1 Loadings							
p53	Chk2	ATR	Chk1	ATX	DNAPK	Bok	Bim
0.29	0.64	0.31	0.16	−0.28	−0.35	−0.42	0.10

Figure S99. Principal-component analysis of doxaliplatin (DoxPt) and equipotent doxorubicin-oxaliplatin (Dox+Ox) physical mixture, Top2 poisons, transcription–translation inhibitors, and anti-metabolites. Tables show the percentage variance explained by each principal component, as well as the Principal Component 1 (PC1) loadings. Shaded boxes represent the approximate space the category occupies in the PCA and are meant only to aid visualization, not to be used for category classification.

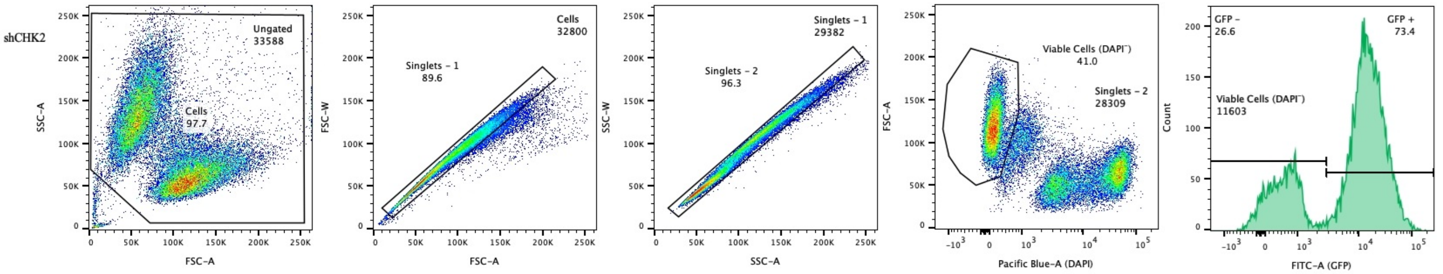

Figure S100. Representative data showing gating strategies for flow cytometry experiments. Eμ-Myc cells were first selected based on forward and side scatter characteristics (FSC-A vs SSC-A) to identify the lymphocyte population. Sequential doublet discrimination was performed using pulse geometry gating (FSC-A vs FSC-W followed by SSC-A vs SSC-W) to ensure analysis of single cells only. Dead cells were excluded by gating on DAPI-negative events (DAPI-A vs FSC-A). GFP expression was quantified as the percentage of GFP-positive cells within the live, single-cell population.

S11. Mechanisms of action of DoxPt study with the NCI60 screening panel.

The mechanisms of action (MOA) of DoxPt were investigated with the NCI60 screening panel[14] and a pattern comparison using the COMPARE algorithm.[15] As shown in Table S5, the analysis indicates that DoxPt acts both as a TOP2 inhibitor, including anthracycline drugs (highlighted in red) and an N^7-alkylating agent, like classical platinum anticancer drugs (highlighted in blue). This result is consistent with the RNAi-based competition assay. In addition, the analysis also reveals that DoxPt may also target Top1 (highlighted in yellow, Table S5). This founding is confirmed by the wheat germ Top1 unwinding assay, which shows moderate Top1 inhibition by DoxPt.

Table S5. Correlation between DoxPt and commonly used anticancer agents analyzed with the COMPARE algorithm based on the NCI60 Screening Panel.

NSC	Name	MOA	Correlations
854109	Doxaliplatin (DoxPt)	-	1.000
3088	Chlorambucil	A7|AlkAg	0.758
8806	Melphalan	A7|AlkAg	0.753
141540	Etoposide	TOP2	0.75
34462	Uracil mustard	A7|AlkAg	0.742
122819	Teniposide	TOP2	0.728
9706	Triethylenemelamine	A7|AlkAg	0.719
409962	Carmustine	A7|AlkAg	0.716
25154	Pipobroman	A7|AlkAg	0.709
757804	Etoposide	TOP2	0.704
757098	Melphalan	A7|AlkAg	0.717
6396	Thiotepa	A7|AlkAg	0.688
758667	Teniposide	TOP2	0.683
760764	Voreloxin	TOP2	0.677
249992	M-AMSA	TOP2	0.666
256942	Epirubicin	TOP2	0.677
246131	Valrubicin	TOP2	0.67
758255	Teniposide	TOP2	0.654
301739	Mitoxantrone	TOP2	0.641
758423	M-AMSA	TOP2	0.639
141549	M-AMSA	TOP2	0.633
32065	Hydroxyurea	AM|Dr	0.631
79037	Lomustine	A6|AlkAg	0.629
82151	Daunorubicin	TOP2	0.625
154948	M-AMSA	TOP2	0.631
616348	Irinotecan	TOP1	0.619
241240	Carboplatin	A7|AlkAg	0.606
759878	Irinotecan	TOP1	0.605
279836	Mitoxantrone	TOP2	0.601
762	Nitrogen mustard	A7|AlkAg	0.597
256439	Idarubicin	TOP2	0.597
757087	Nitrogen mustard	A7|AlkAg	0.595
756717	Daunorubicin	TOP2	0.589
759195	Epirubicin	TOP2	0.58
765396	BMN-673	PARP1|PARP2	0.566
119875	Cisplatin	A7|AlkAg	0.559
156303	M-AMSA	TOP2	0.559
26980	Mitomycin	A2|AlkAg	0.54
109724	Ifosfamide	A7|AlkAg	0.566
609699	Topotecan	TOP1	0.529

767125	BMN-673	PARP1|PARP2	**0.532**
123127	Doxorubicin	TOP2	**0.516**
83142	Daunorubicin	TOP2	**0.515**
639186	Raltitrexed	Ds|DHFR|TYMS	**0.514**
290193	ciclosporin	-	**0.552**
75520	tfdu	TYMS	**0.504**
138783	Bendamustine	A2|A6|AlkAg	**0.521**
706363	Arsenic trioxide	Apo	**0.503**
759155	Doxorubicin	TOP2	**0.498**
63878	Cytarabine	Ds	**0.494**
755605	Enzalutamide	Ho|AR	**0.489**
758612	Bleomycin	Db	**0.48**
759263	Topotecan	TOP1	**0.477**
94600	Camptothecin	TOP1	**0.477**
92859	Arsenic trioxide	Apo	**0.479**
100880	Camptothecin	TOP1	**0.469**
728073	Irinotecan	TOP1	**0.483**
4375	Hydroxychloroquine Sulfate	Immunomodulatory	**0.494**
169780	Dexrazoxane	TOP2|NonCan:Cardioprotective	**0.457**
45388	Dacarbazine	A7|AlkAg	**0.453**
755880	Mitomycin	A2|AlkAg	**0.451**
673596	7-Ethyl-10-hydroxycamptothecin	TOP1	**0.447**
613327	Gemcitabine	Ds	**0.441**
321521	auranofin	-	**0.476**
755986	Vismodegib	SMO|PK:YK	**0.434**
125066	Bleomycin	Db	**0.433**
266046	Oxaliplatin	A7|AlkAg	**0.452**
606170	Zalcitabine	Ds	**0.47**
27640	Floxuridine	Ds|TYMS	**0.416**
113926	rifa	-	**0.447**
134727	Mitomycin	A2|AlkAg	**0.407**
656576	Midostaurin	PK:PRKCA,STK,FLT3	**0.402**
105024	Mithramycin	Db|Rs	**0.405**
67574	Vincristine	TUBB|Tu-frag	**0.407**
287459	Cytarabine	Ds	**0.398**
755400	Masitinib	PK:YK,PDGFR,FGFR, KIT,FGFR3	**0.396**
109229	Asparaginase	AM|Pi	**0.393**
752	6-Thioguanine	Ds|IMPDH2|IMPDH	**0.392**
145668	Ancitabine hydrochloride	Ds	**0.392**
3053	Actinomycin D	Db	**0.39**
797937	Sulfatinib	PK:YK,FGFR,KDR	**0.395**
753686	Olaparib	PARP|PARP1	**0.383**
759857	Clofarabine	Ds	**0.378**
12198	Dromostanolone Propionate	Ho	**0.376**
665970	Artemether	NonCan:Mal	**0.381**
4291	Masoprocol	-	**0.366**
755984	Pralatrexate	Df|CTNNB1|DHFR	**0.365**
755985	Nelarabine	Ds	**0.365**
606698	Rapamycin	PK:STK,MTOR	**0.368**
226080	Rapamycin	PK:STK,MTOR	**0.356**
759274	Arsenic trioxide	Apo	**0.353**
3061	malacid	DHFR	**0.353**
801082	BLU-285	-	**0.41**
759817	artesunate	-	**0.349**
683864	Temsirolimus	PK:STK,MTOR	**0.356**
698037	Pemetrexed	Df|AM|GARTF|DHFR| TYMS	**0.35**
141633	Homoharringtonine	Apo|Ang|Pi	**0.38**
694501	BN-2629	Db	**0.674**

724998	LMP-400	TOP1	**0.617**
697887	XK-469	TOP2	**0.657**
320846	Batracylin	TOP2|TOP1	**0.566**
698215	XK-469	TOP2	**0.555**
656889	XK-469	TOP2	**0.559**
663249	Triapine	Dr|CC	**0.512**
783916	pyridoclax	MCL1	**0.504**
374551	Fenretinide	Ds	**0.503**
807055	AZD-5991	-	**0.503**
798846	S-63845	-	**0.496**
793679	CNDAC	-	**0.48**
784722	Aldoxorubicin	TOP2	**0.479**
697726	RH1	PK:ROS1	**0.474**
760419	Fenretinide	Ds	**0.474**
759096	Triapine	Dr|CC	**0.473**
36405	Chelerythrine	BCL2|PK:PRKCA,STK	**0.496**
791785	Sapacitabine	Ds	**0.466**
809971	S-64315	-	**0.458**
771858	TAK-441	SMO	**0.464**
714597	Imexon	Apo	**0.454**
773230	AZD-2461	PARP1|PARP2	**0.453**
764755	Cerulenin	FAS|FASN|HMGCS1/2	**0.451**
264137	Elliptinium Acetate	TOP2	**0.458**
761192	Pevonedistat	NFkB|NEDD8	**0.453**
782164	CX-5461	RNA polymerase I	**0.449**
772886	Apatinib	PK:VEGFR	**0.435**
782125	JNJ-54302833	FASN	**0.426**
774901	AZD-2858	Wnt|PK:GSK3A	**0.422**
780163	SOMCL-12-81	PK:ALK	**0.421**
804041	AMG-176	-	**0.418**
757444	Barasertib	PK:STK,AURK	**0.404**
638646	7-Hydroxystaurosporine	PK:STK,CDK1,CDK2,<br>CDK4,CDK6	**0.422**
792955	GSK-2194069	FAS|FASN	**0.399**
788794	A-911	PK:CDK,CDK9	**0.391**
365798	Fostamatinib	Ds|Rs|PK:YK,SYK	**0.426**
784591	CCT-251545	Wnt|PK:CDK19	**0.39**
697912	5-Fluoro deoxy uridine 10mer	Ds|AM	**0.385**
794443	KPT-8602	XPO1	**0.38**
762153	ST-3595	HDAC	**0.375**
308847	AMONAFIDE	TOP2	**0.378**
787025	BAY-1161909	IDUA	**0.373**
725776	LMP776	TOP1	**0.376**
764659	SCH-900776	PK:STK,CHEK,CHEK1	**0.372**
708298	AR-67	TOP1	**0.371**
761070	VE-821	PK:STK,ATR	**0.371**
765775	Gemcitabine elaidate	Ds	**0.373**
761693	Vertex ATR inhibitor Cpd 45	PK:STK,ATR	**0.369**
805746	CC-671	PK:TTK	**0.367**
756660	Quercetin	TOP2|AO|NonCan:COMT	**0.367**
802100	ON-123300	PK:CDK,RET	**0.365**
649890	Alvocidib	CCNT1|PK:STK,CDK,CDK1,CDK2,<br>CDK4,CDK6,CDK7,CDK8,CDK9	**0.361**
771531	RG-7112	MDM2	**0.364**
759498	CYC-116	PK:STK,AURK	**0.374**
766824	CEP-14083	PK:ALK	**0.362**
779404	RG-7388	MDM2	**0.358**
756662	sr 13654	BIRC5|PK:YK	**0.355**
758485	BX-912	PK:STK,PDK1	**0.354**
809100	RG-7112	MDM2	**0.355**

781009	MI-773	MDM2	**0.352**
802450	BAY-1251152	-	**0.348**
764481	Brivanib	PK:YK,FGFR,VEGFR,FGFR1, FGFR2,FGFR3,KDR	**0.345**
765889	ABT-348	PK:AURK,VEGFR	**0.351**
813862	Lapachone	TOP2|TOP1	**-0.357**
668814	Kahalide F	-	**-0.467**

S12. Investigation of the inhibitory activity of DoxPt to human Top2α using decatenation assay

The inhibition of human Top2α by DoxPt was investigated with a decatenation assay (Inspiralis Limited), which uses kinetoplast DNA (kDNA) from *Crithidia fasciculata*. This substrate comprises a network of minicircles (2.3 Kb) with some maxicircles. Top2α releases minicircles from the network. During gel electrophoresis, the maxicircles, not always visible, remain in the wells, but the released minicircles migrate through the gel, where they can be visualized with ethidium bromide staining. All experiments were conducted according to the following procedure provided by Inspiralis Limited.

Enzyme activity was determined prior to the testing of the compounds. Here, one unit (U) was defined as the amount of enzyme required to completely release minicircles from 200 ng of kDNA by full decatenation. This amount of enzyme was initially used to determine the activity of doxorubicin as a control inhibitor. The experiments with DoxPt and doxorubicin were performed in duplicate. For all assays, the final glycerol in the reaction mix was 6 vol %. For the experiments with doxorubicin, the DMSO concentration was 2 vol %, as shown, or 0% otherwise.

Specifically, human Top2α (1 U) was incubated with 200 ng of kDNA in a 30 μL reaction mixture at 37 °C and pH 7.5 for 30 min. The reaction media contained 50 mM Tris·HCl, 125 mM NaCl, 10 mM $MgCl_2$, 5 mM dithiothreitol (DTT), and 100 μg/mL albumin. Each reaction was stopped by extracting with a sat. aq. solution of *n*-butanol (50 μl) before addition of chloroform/iso-amyl alcohol (24:1, 30 μL) and 30 μL Stop Dye (pH 7.5, 40 w/v % sucrose, 100 mM Tris·HCl, 10 mM EDTA, 0.5 μg/mL bromophenol blue). The samples were then loaded on a 1.0% Tris Acetate EDTA (TAE) gel. Electrophoresis was conducted at 90 V for about 1 h. Upon completion, bands were visualized by ethidium staining for 10 minutes, de-stained for 10 minutes in water, and analyzed by gel documentation equipment. The quantification was performed using Syngene GeneTools software. Fluorescent band volumes from raw gel data were normalized as a percentage intensity relative to the fully decatenated DNA band control (set at 100%) obtained without the inhibitor. Fluorescence exceeding 100% of decatenation was adjusted to 100%. The percentage intensity for each well was then converted to percentage inhibition.

The master mix for determining the CC_{50} value of doxorubicin (16 samples) contained water (316.8 μL), 10× assay buffer (48 μL), kDNA (100 ng/μL, 32 μL), an ATP solution (30 mM, 16 μL), and a glycerol/water mixture (1:1, v/v, 9.6 μL).

The master mix for determining the CC_{50} value of DoxPt (16 samples) contained water (316.8 μL), 10× assay buffer (48 μL), kDNA (100 ng/μL, 32 μL), and an ATP solution (30 mM, 16 μL).

Table S6. IC_{50} values of Dox and DoxPt determined in the human Top2α decatenation assay.

Compound	IC_{50} (μM) Replicate 1	IC_{50} (μM) Replicate 2	Average IC_{50} (μM)
Dox	2.48	2.46	2.47
DoxPt	9.96	6.44	8.20

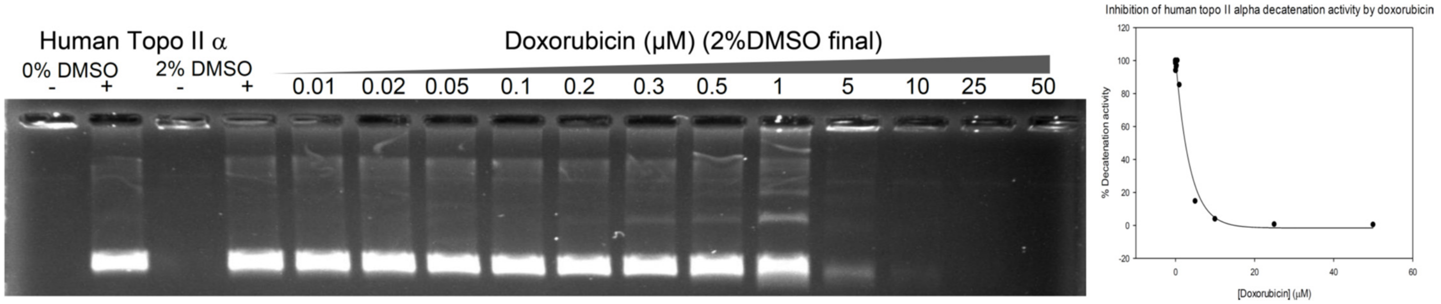

Figure S101. Representative gel image of human Top2α decatenation assay with doxorubicin as the inhibitor and the corresponding quantification (the uncropped gel can be found in Source Data file).

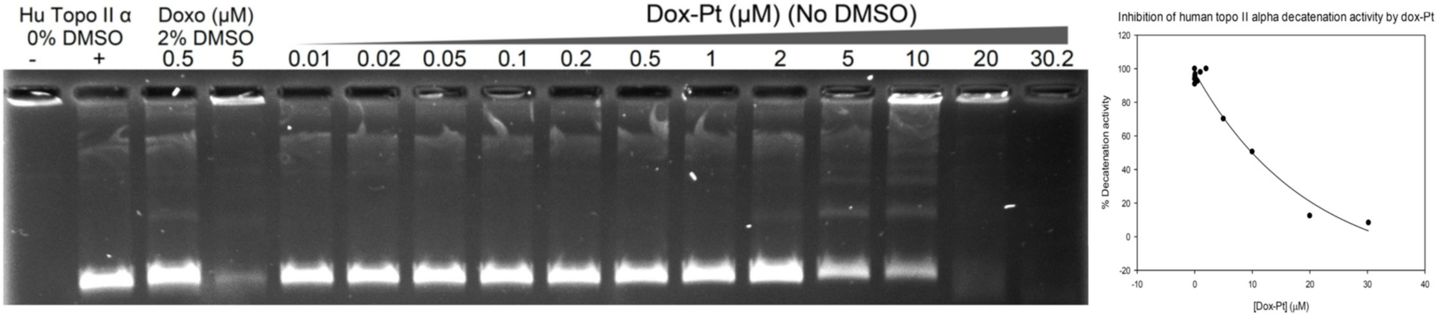

Figure S102. Representative gel image of human Top2α decatenation assay with DoxPt as the inhibitor and the corresponding quantification (the uncropped gel can be found in Source Data file).

S13. Investigation of the mode of action of DoxPt with human Top2α cleavage assay

The mode of action of DoxPt was studied with a human Top2α cleavage assay (Inspiralis Limited). For this assay, 1 U of human Top2α was incubated with supercoiled plasmid DNA (pBR322, 0.5 μg) at pH 7.5 and 37 °C for 30 min in a 30 μL reaction mixture containing 20 mM Tris·HCl, 200 mM NaCl, 0.25 mM EDTA, 2 vol % DMSO (except for the reactions with DoxPt) and 5 vol % glycerol. The reaction was further incubated for 30 min with 0.2 % SDS and 0.5 μg/μl proteinase K. All experiments were conducted according to the following procedure provided by Inspiralis Limited.

Each reaction was stopped by extracting the reaction with a sat. aq. solution of *n*-butanol (50 μL) to remove the anticancer agent. The samples were vortexed for 10 s and centrifuged for 30 s. The *n*-butanol-containing top phase was removed. The reactions were further treated with chloroform/isoamyl alcohol (24:1 , 30 μL) and 30 μL Stop Dye (pH 7.5, 40% sucrose (w/v), 100 mM Tris·HCl, 10 mM EDTA, 0.5 μg/mL bromophenol blue). The aqueous phase of the samples (20 μL) was then loaded on a 1% TAE gel. Electrophoresis was conducted at 90 V for 1.5 h in the presence of ethidium bromide (0.5 μg/mL) unless mentioned otherwise. Upon completion, the gels were scanned with documentation equipment. Cleavage levels were quantified from band data obtained using gel scanning software (GeneTools, Syngene, Cambridge, UK).

The master mix for determining the CC_{50} value of doxorubicin (16 samples) contained water (340.8 μL), 10× assay buffer (48 μL), supercoiled DNA - pBR322 (1 μg/μL, 8 μL), ATP solution (30 mM, 16 μL), and a glycerol/water mixture (1:1, v/v, 9.6 μL).

The master mix for determining the CC_{50} value of etoposide (16 samples) contained water (340.8 μL), 10× assay buffer (48 μL), supercoiled DNA - pBR322 (1 μg/μL, 8 μL), ATP solution (30 mM, 16 μL), and a glycerol/water mixture (1:1, v/v, 9.6 μL).

The master mix for determining the CC_{50} value of DoxPt (16 samples) contained water (340.8 μL), 10× assay buffer (48 μL), supercoiled DNA - pBR322 (1 μg/μL, 8 μL), and an ATP solution (30 mM, 16 μL).

Table S7. CC_{50} values of etoposide, Dox, and DoxPt determined in the human Top2α cleavage assay.

Compound	CC_{50} (μM) Replicate 1	CC_{50} (μM) Replicate 2	Average CC_{50} (μM)
Etoposide	56.5	60.3	58.4
Dox[a]	–	–	–
DoxPt[a]	–	–	–

[a]Not determined. Under the tested conditions, cleavage complex stabilization was not observed with Dox and DoxPt.

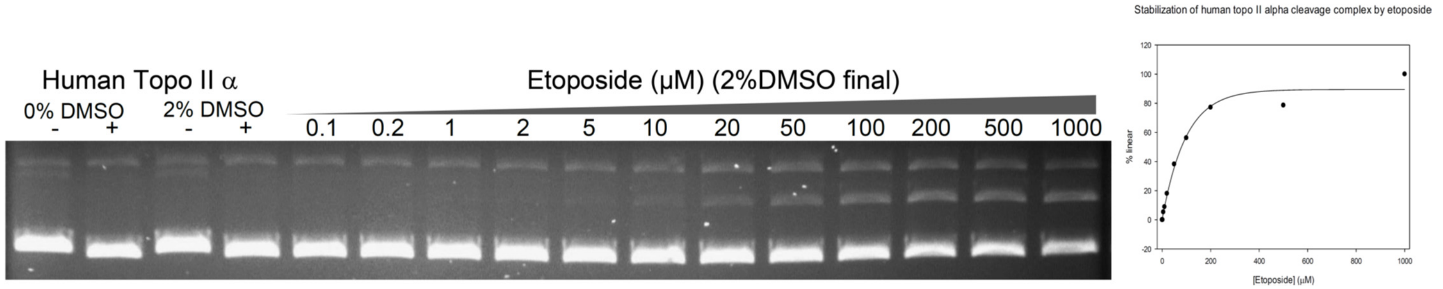

Figure S103. Representative gel image of human Top2α cleavage assay with etoposide in the presence of ethidium bromide and the corresponding quantification (the uncropped gel can be found in Source Data file).

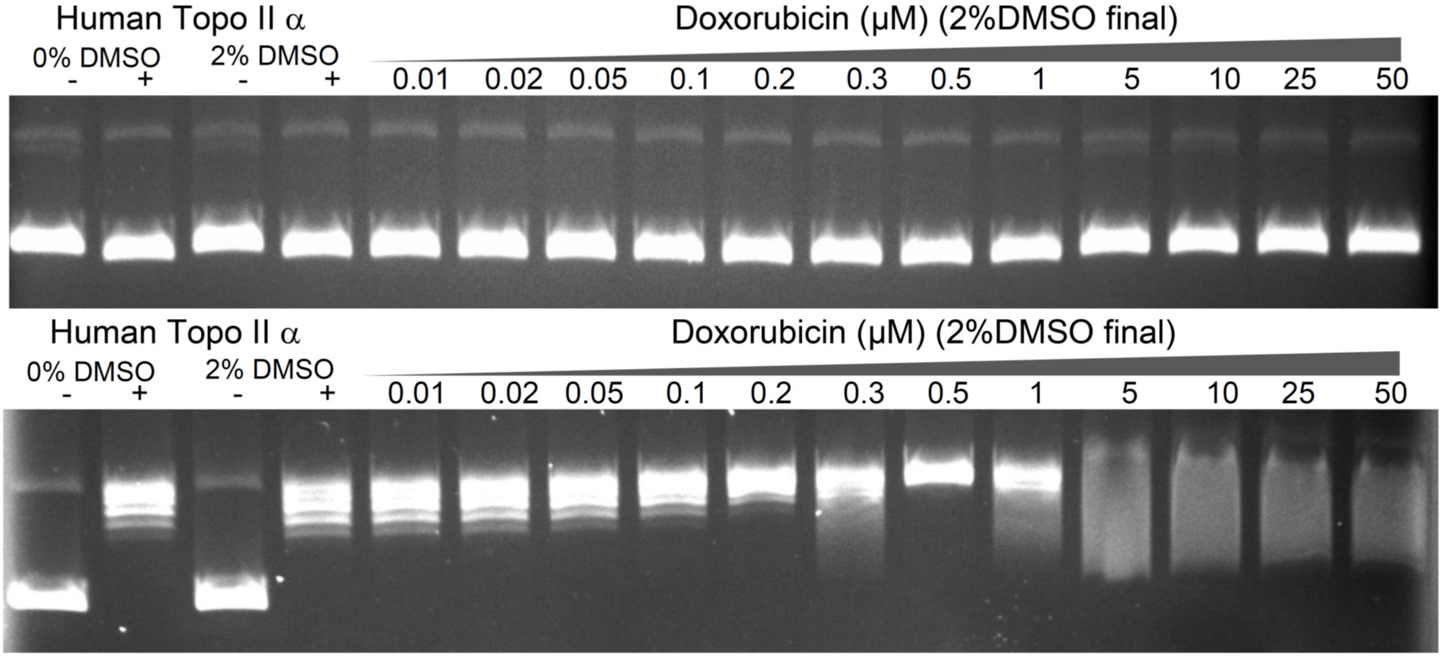

Figure S104. Gel image of human Top2α cleavage assay with doxorubicin in the presence of ethidium bromide (top) and in the absence of ethidium bromide (bottom). The uncropped gels can be found in Source Data file.

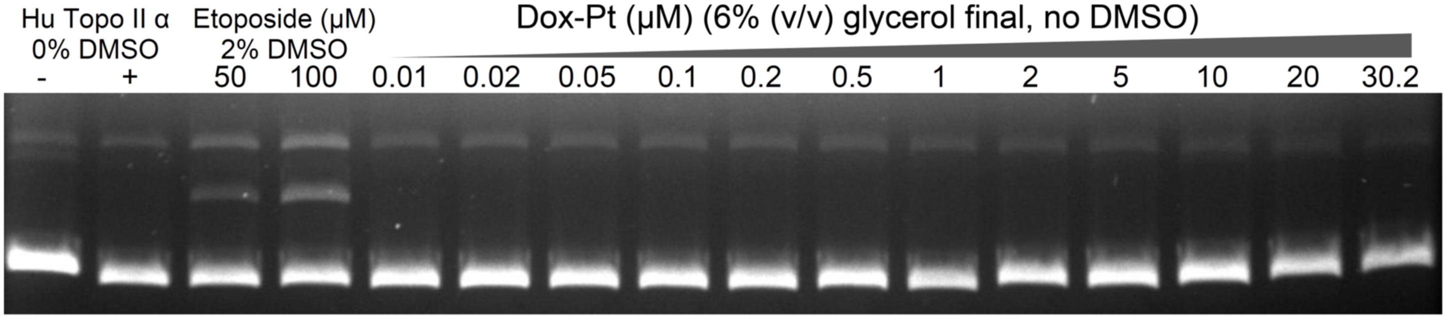

Figure S105. Representative gel image of human Top2α cleavage assay with DoxPt in the presence of ethidium bromide (the uncropped gel can be found in Source Data file).

S14. Investigation of the mode of action of DoxPt with wheat germ Top1 intercalation (unwinding) assay

The mode of action of DoxPt was studied with a wheat germ Top1 unwinding assay (Inspiralis Limited). All experiments were conducted according to the following procedure provided by Inspiralis Limited. For this assay, wheat germ Top1 (2 U) was incubated with supercoiled or relaxed plasmid DNA (pBR322, 0.5 μg) at pH 7.9 and 37 °C for 30 min in a 30 μL reaction mixture containing 50 mM Tris·HCl, 50 mM NaCl, 1.0 mM EDTA, 1.0 mM DTT, and 20% glycerol. After each reaction was stopped, the anticancer agent was removed before electrophoresis by adding a sat. aq. solution of *n*-butanol (50 μL). The samples were vortexed and the top *n*-butanol layer was removed before the addition of chloroform/isoamyl alcohol (24:1, 30 μL) and Stop Dye (30 μL). Samples were resolved on a 1.0% TAE gel run at 90 V for 2 h. Bands were visualized by ethidium bromide staining for 10 min and de-stained for 10 min in water. The images were quantified from band data obtained using gel scanning software (GeneTools, Syngene, Cambridge, UK).

The control compound doxorubicin at a concentration of 0.5 μM and above resulted in a change in mobility of the pBR322 towards (positively) supercoiled form irrespective of the topology of the substrate used. At concentrations of 20 μM and above, its binding interferes with the DNA migration in the gel. DoxPt shows intercalation at 5 μM. The intercalation reached a maximum at 10 μM as the compound started to interfere with DNA migration at this concentration and above. The abnormal DNA migration at higher agent concentrations may be attributed to the incomplete removal of anticancer agents or enzyme from the DNA during the extractions. As expected, the doxorubicin control exhibits intercalating effects at 0.5 μM under the tested conditions. In contrast, DoxPt also acted as a relatively weaker DNA intercalator, showing the effect at about 5 μM.

The master mix (16 samples) for Dox with supercoiled DNA and amsacrine (m-AMSA, 100 μM) contained water (164.8 μL), 2× assay buffer (240 μL), supercoiled DNA - pBR322 (1 μg/μL, 8 μL), and a glycerol/water mixture (1:1, v/v, 9.6 μL).

The master mix for DoxPt and Dox controls with supercoiled DNA (16 samples) contained water (164.8 μL), 2× assay buffer (240 μL), and supercoiled DNA - pBR322 (1 μg/μL, 8 μL).

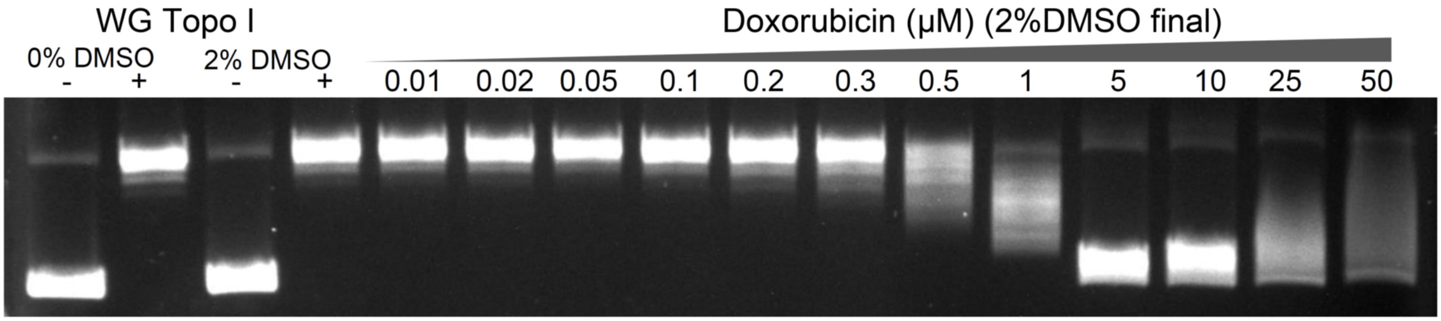

Figure S106. Representative gel image of wheat germ Top1 supercoiled DNA unwinding assay with doxorubicin (the uncropped gel can be found in Source Data file).

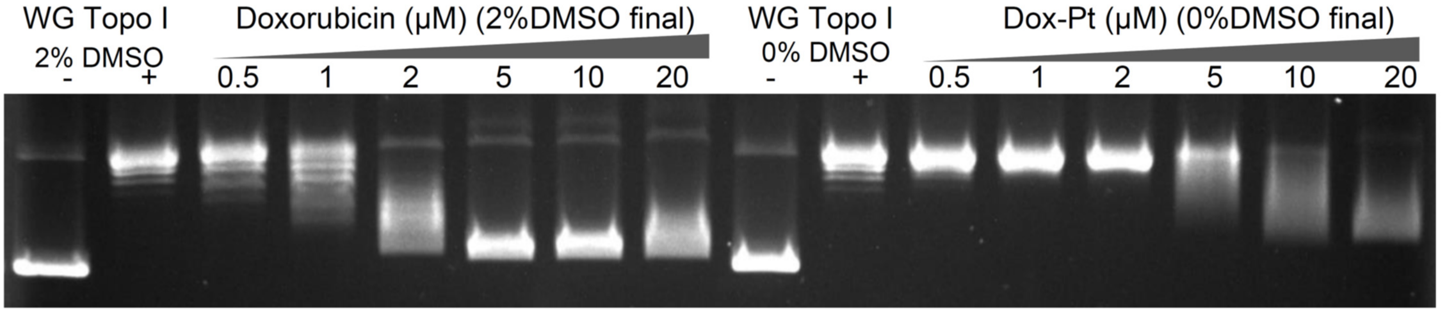

Figure S107. Representative gel image of wheat germ Top1 supercoiled DNA unwinding assay with doxorubicin (control) and DoxPt. The uncropped gel can be found in Source Data file.

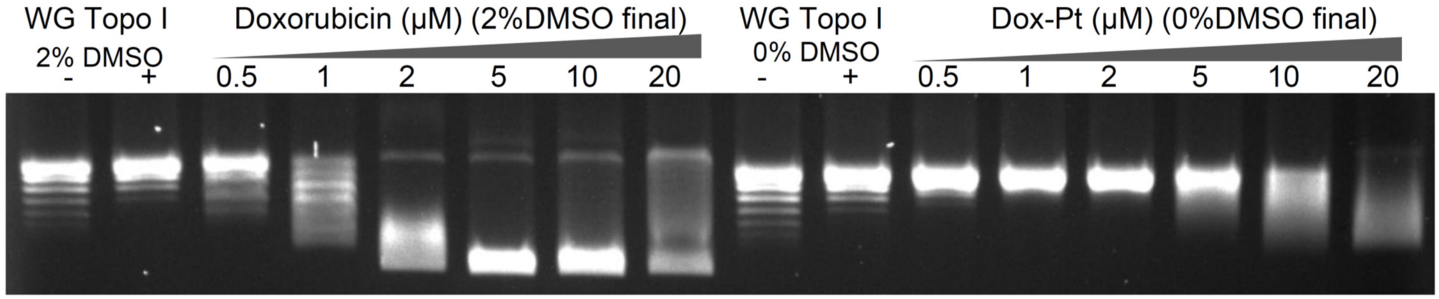

Figure S108. Representative gel image of wheat germ Top1 relaxed DNA unwinding assay with doxorubicin (control) and DoxPt (the uncropped gel can be found in Source Data file).

S15. HPLC Chromatograms of purified compounds.

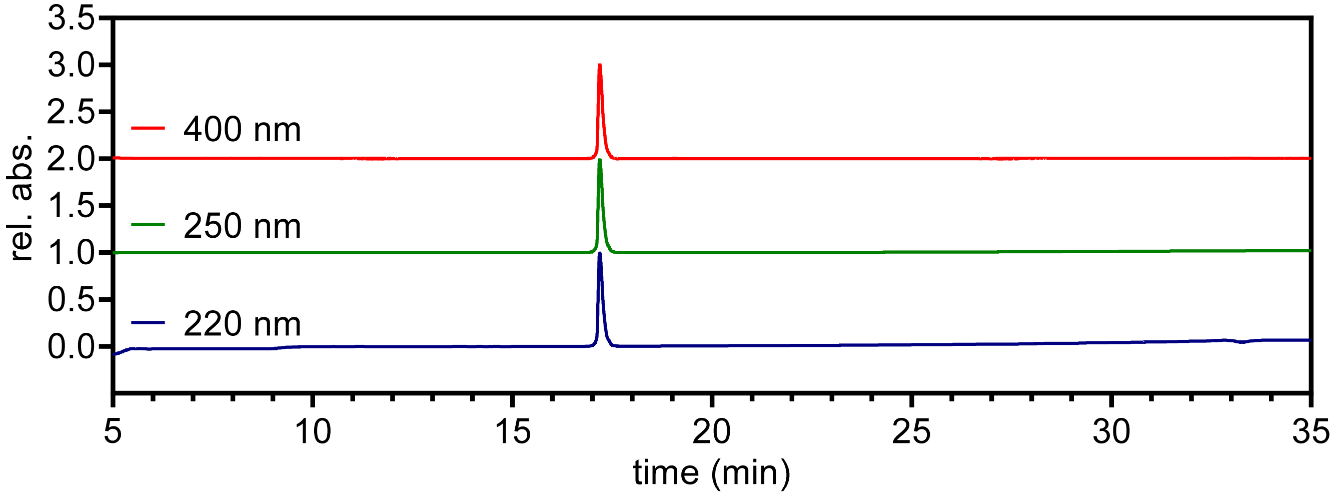

Figure S109. HPLC chromatogram of purified DoxNH_2NH_2. Solvent gradient is given in the text.

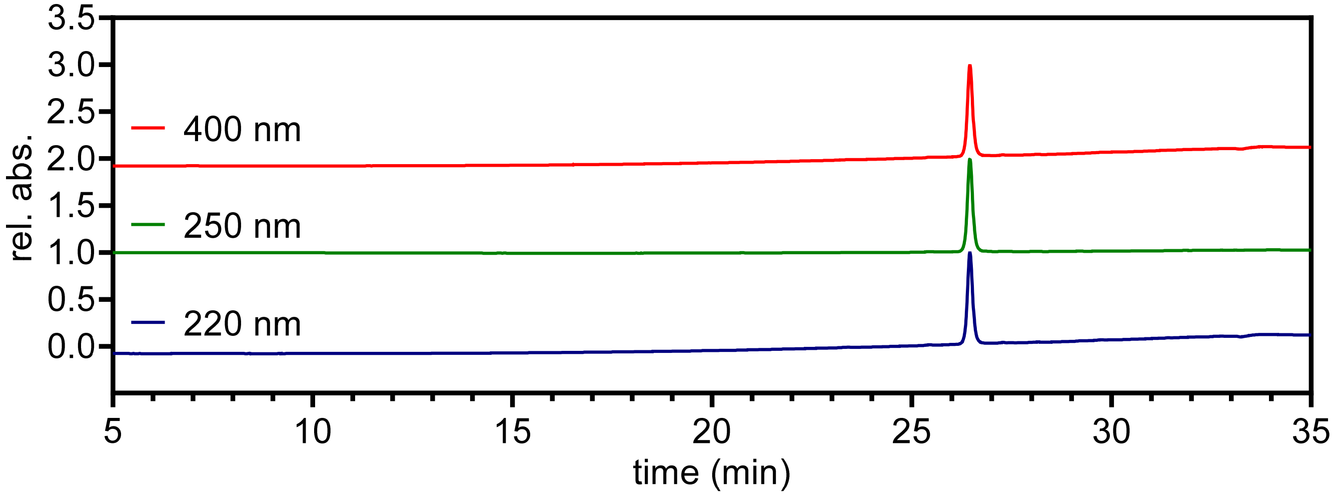

Figure S110. HPLC chromatogram of purified DoxPt. Solvent gradient is given in the text.

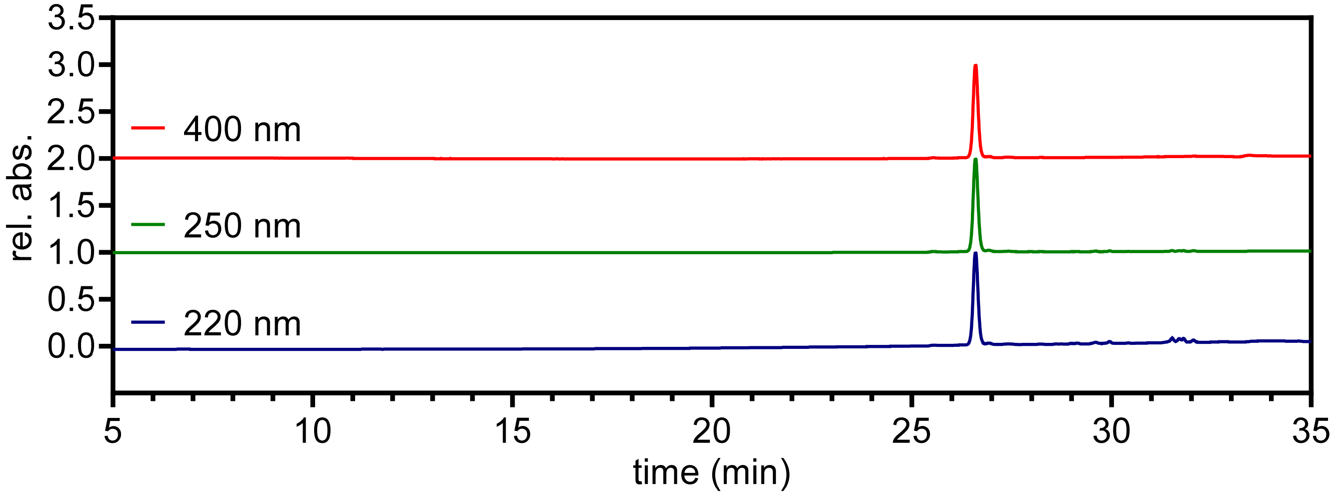

Figure S111. HPLC chromatogram of purified DoxPt2. Solvent gradient is given in the text.

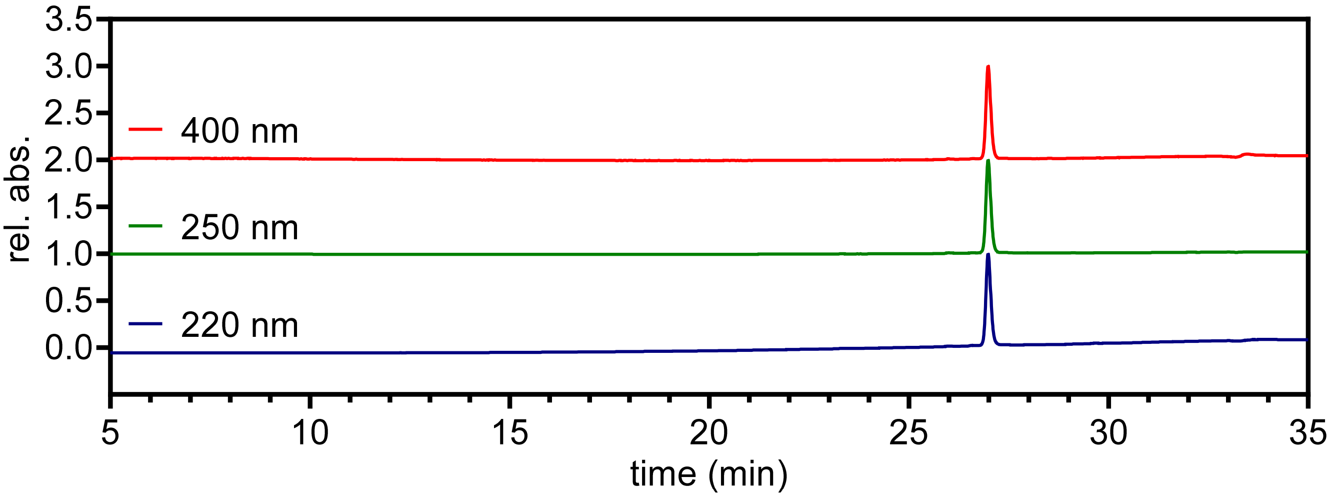

Figure S112. HPLC chromatogram of purified DoxPt3. Solvent gradient is given in the text.

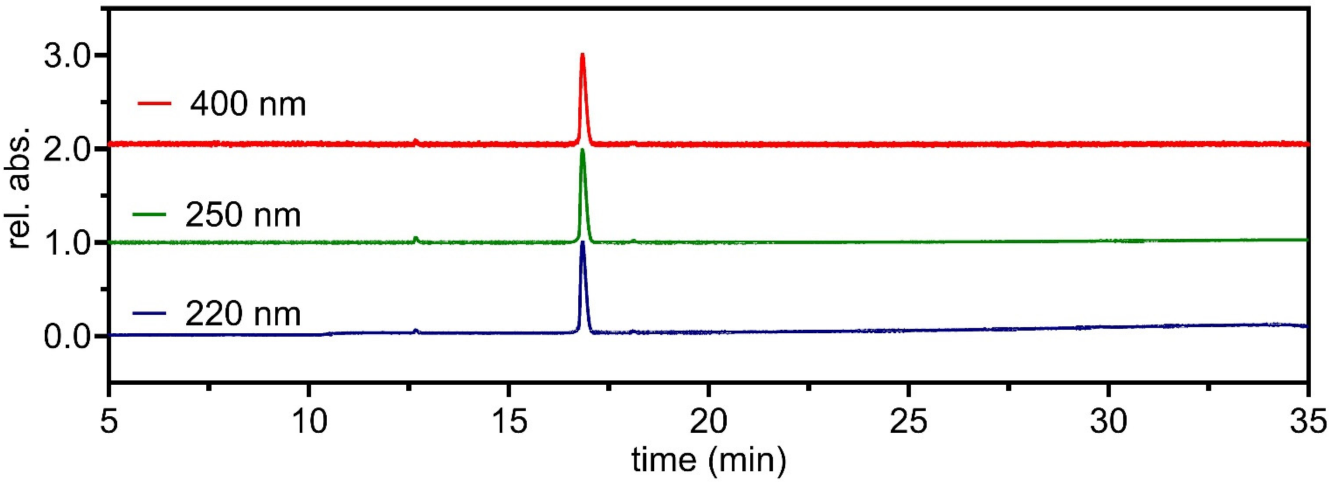

Figure S113. HPLC chromatogram of DoxPt4. Solvent gradient is given in the text.

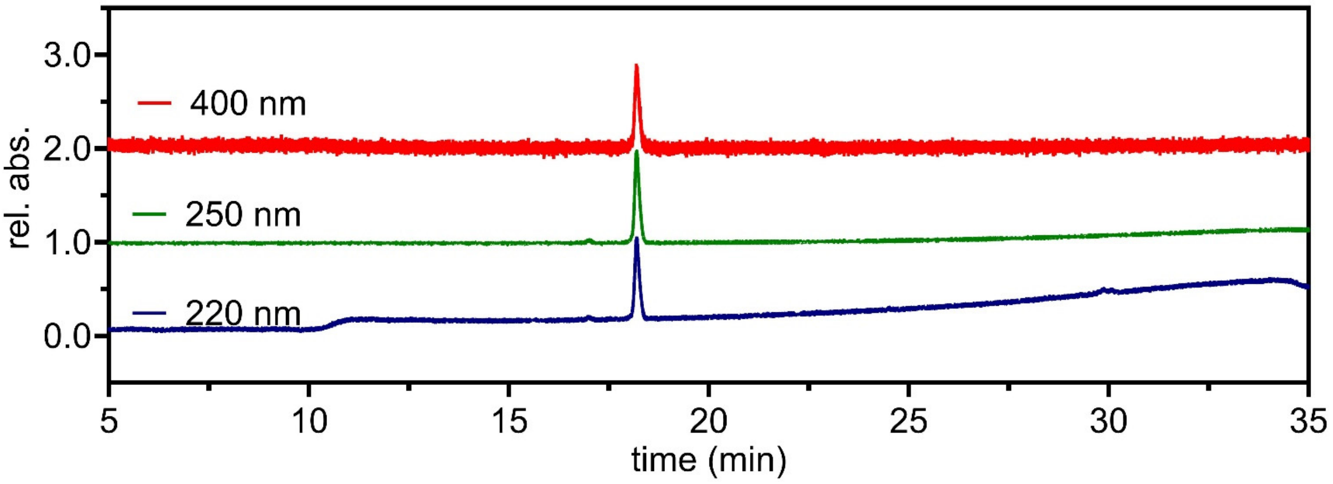

Figure S114. HPLC chromatogram of DoxPt5 HPLC fraction before lyophilization. Solvent gradient is given in the text.

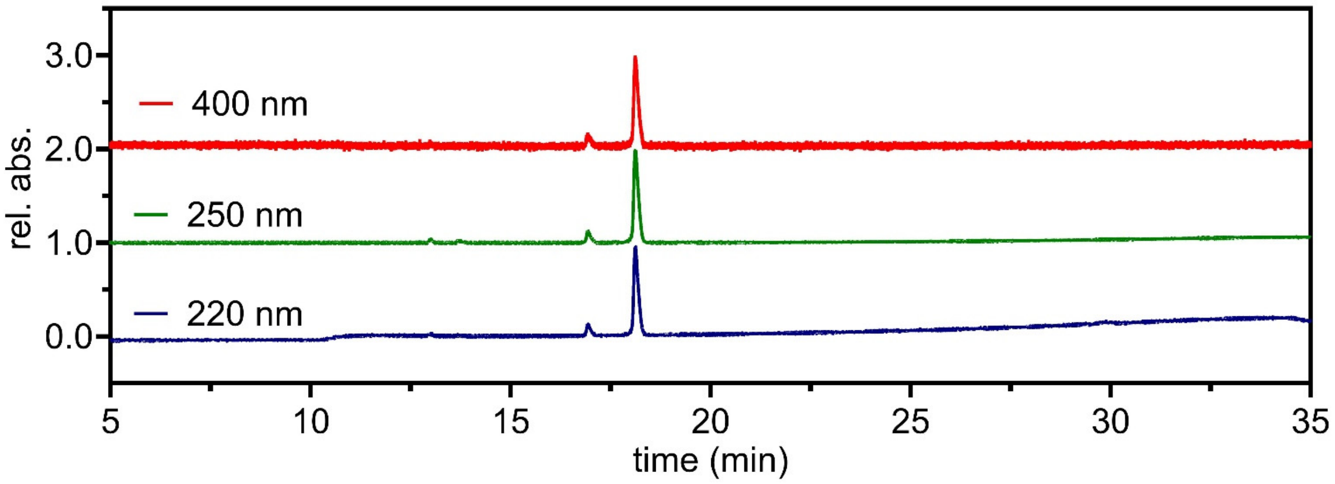

Figure S115. HPLC chromatogram of a freshly prepared aqueous solution of lyophilized DoxPt5. Solvent gradient is given in the text.

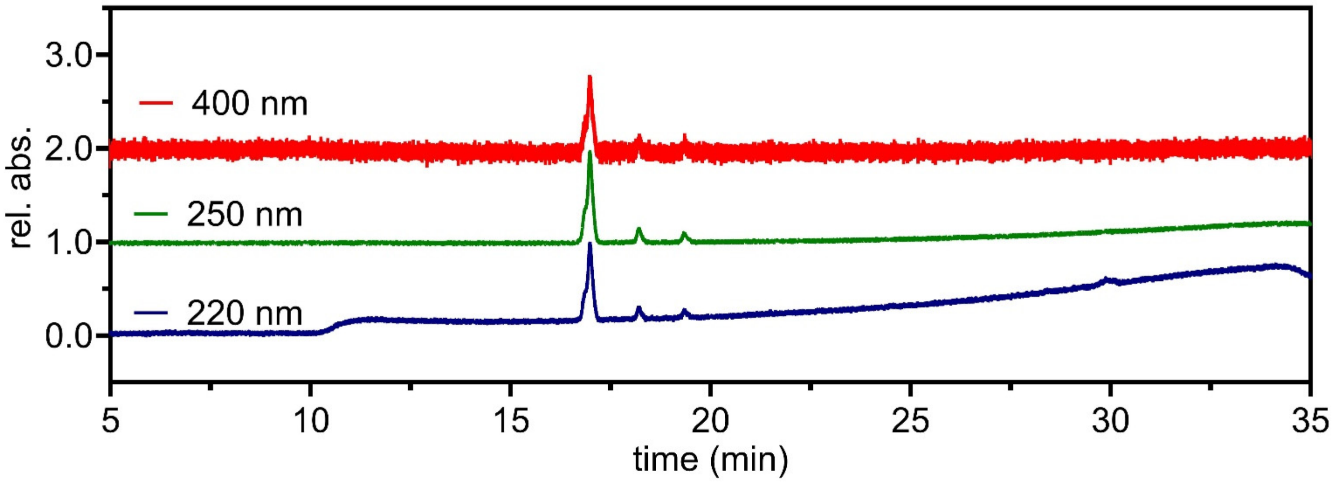

Figure S116. HPLC chromatogram of DoxPt5 in water stored at rt for 24 h, suggesting the conversion into DoxPt4. Solvent gradient is given in the text.

S16. NMR Spectra.

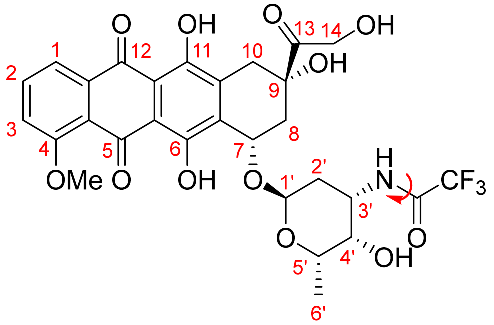

Figure S117. Numbering of *N*-trifluoroacetyl doxorubicin (**6**). The red arrow indicates the slow rotation on the ^{19}F NMR timescale.

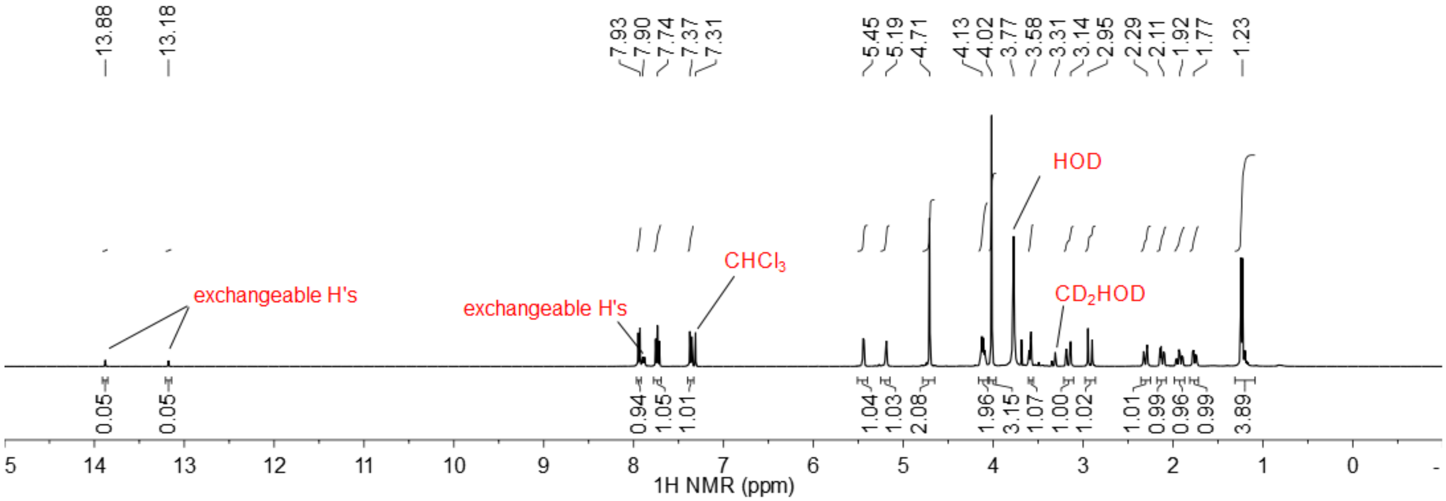

Figure S118. ^{1}H NMR spectrum of *N*-trifluoroacetyl doxorubicin (**6**) in $CDCl_3$-CD_3OD (6:1, *v*:*v*).

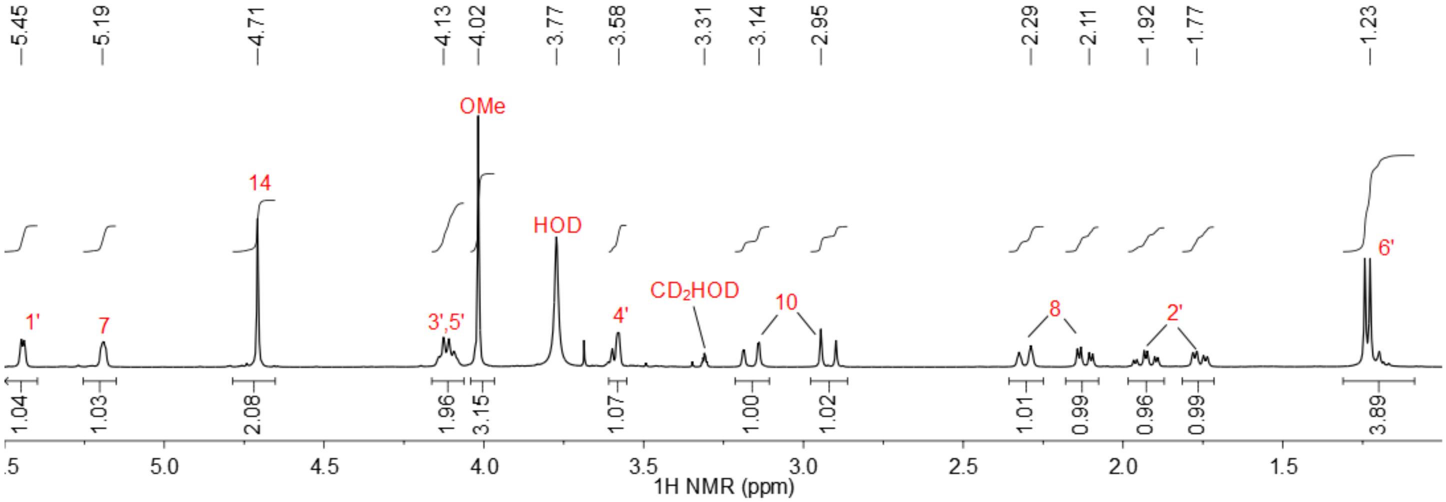

Figure S119. Expansion of ^{1}H NMR spectrum of *N*-trifluoroacetyl doxorubicin (**6**) in $CDCl_3$-CD_3OD (6:1, *v*:*v*) from 1.1 to 5.5 ppm.

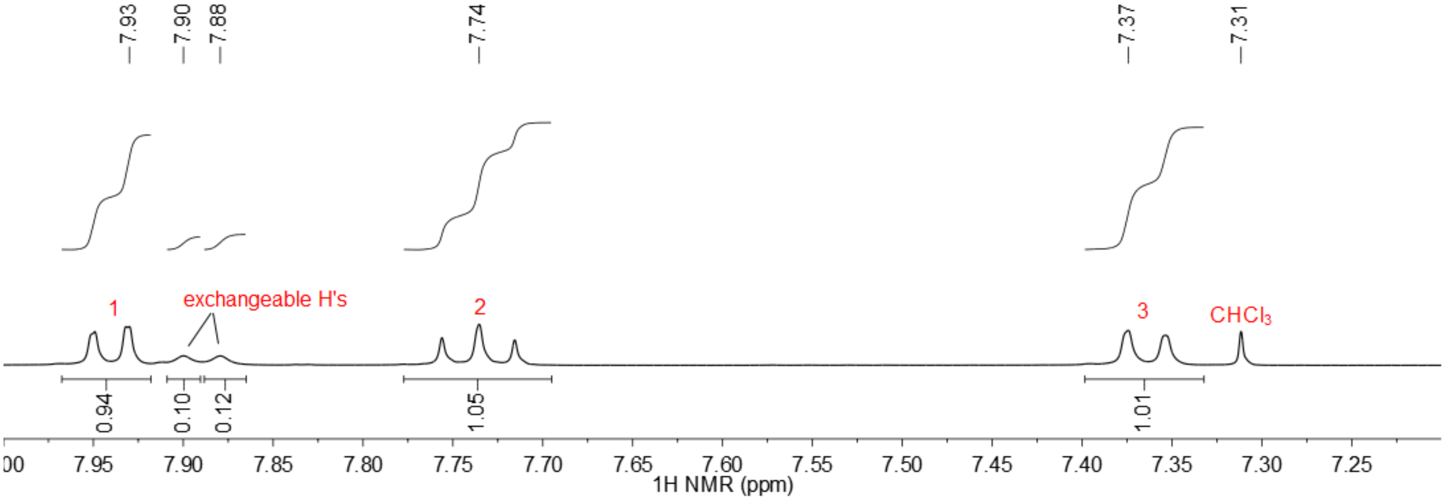

Figure S120. Expansion of ^{1}H NMR spectrum of *N*-trifluoroacetyl doxorubicin (**6**) in $CDCl_3$-CD_3OD (6:1, *v*:*v*) from 7.2 to 8.0 ppm.

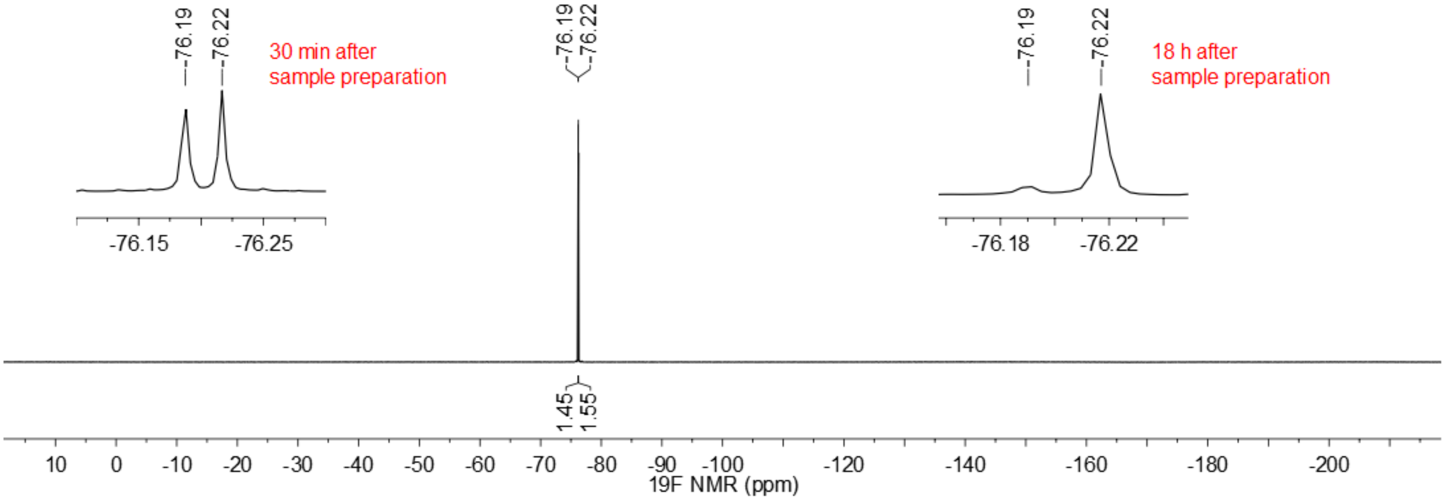

Figure S121. $^{19}F\{^1H\}$ NMR spectra of *N*-trifluoroacetyl doxorubicin (**6**) in $CDCl_3$-CD_3OD (6:1, *v*:*v*) acquired 30 min and 18 h after sample preparation.

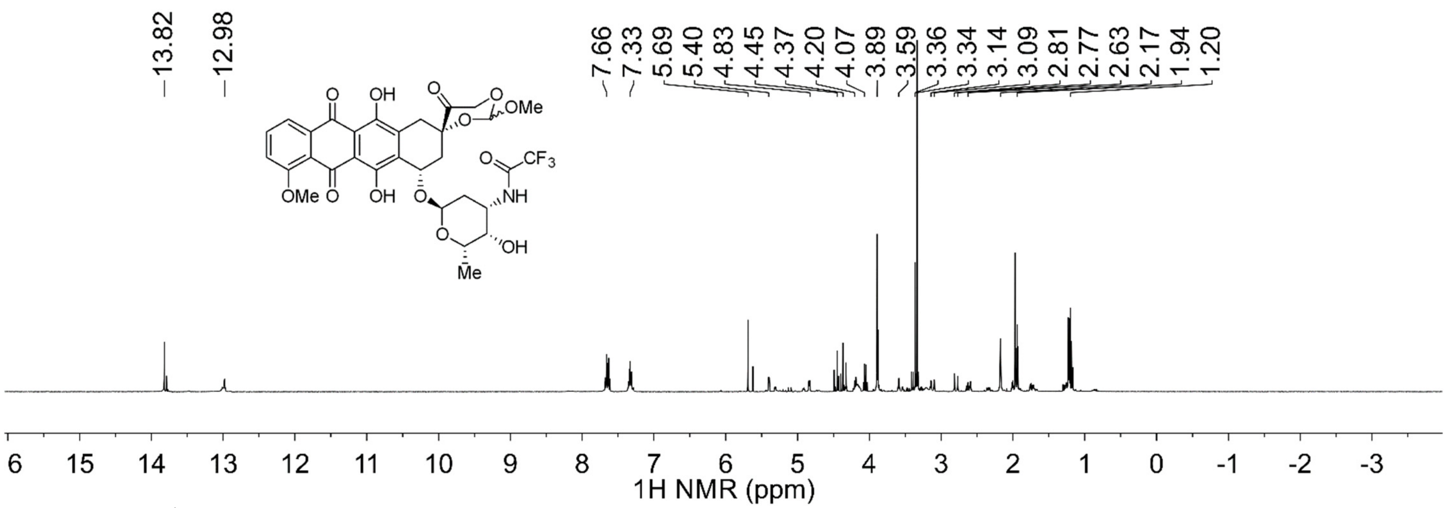

Figure S122. ^{1}H NMR spectrum of *N*-trifluoroacetyl doxorubicin 9,14-cyclic methyl orthoester (**7**) in CD_3CN.

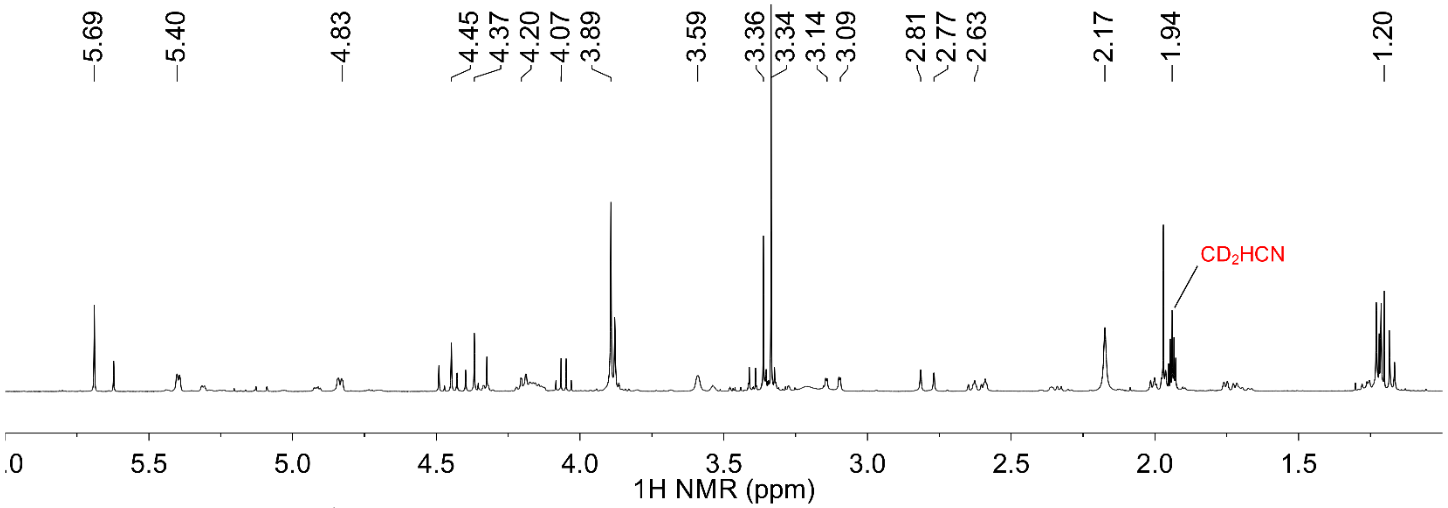

Figure S123. Expansion of ^{1}H NMR spectrum of *N*-trifluoroacetyl doxorubicin 9,14-cyclic methyl orthoester (**7**) in CD_3CN from 1.0 to 6.0 ppm.

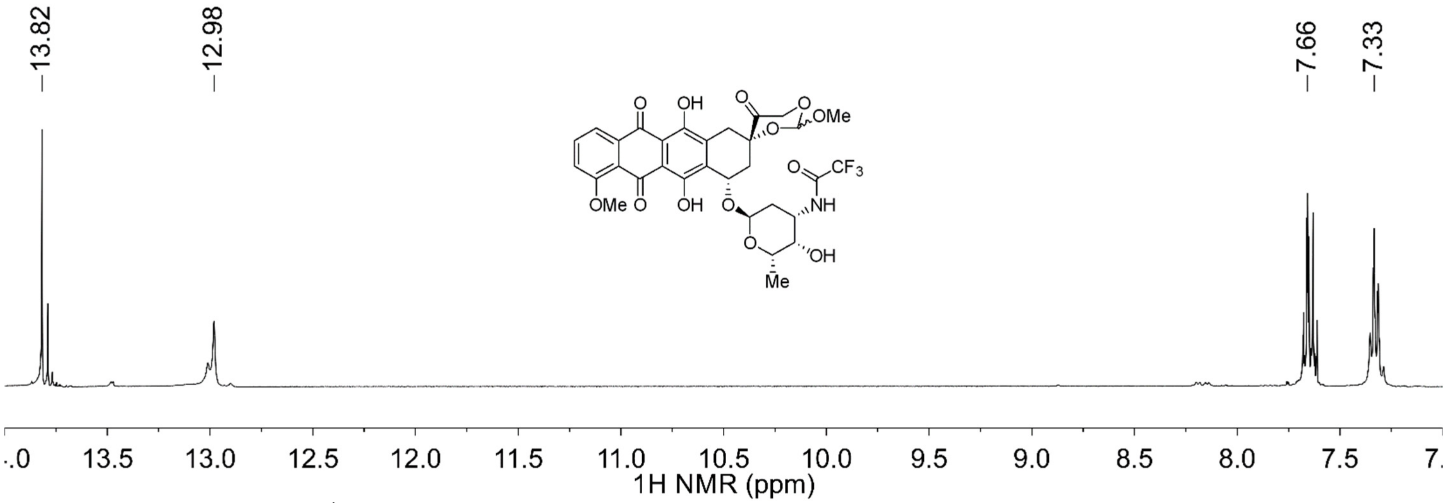

Figure S124. Expansion of ^{1}H NMR spectrum of *N*-trifluoroacetyl doxorubicin 9,14-cyclic methyl orthoester (**7**) in CD_3CN from 7.0 to 14.0 ppm.

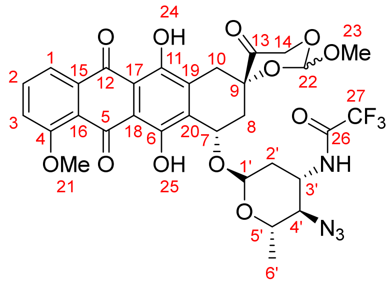

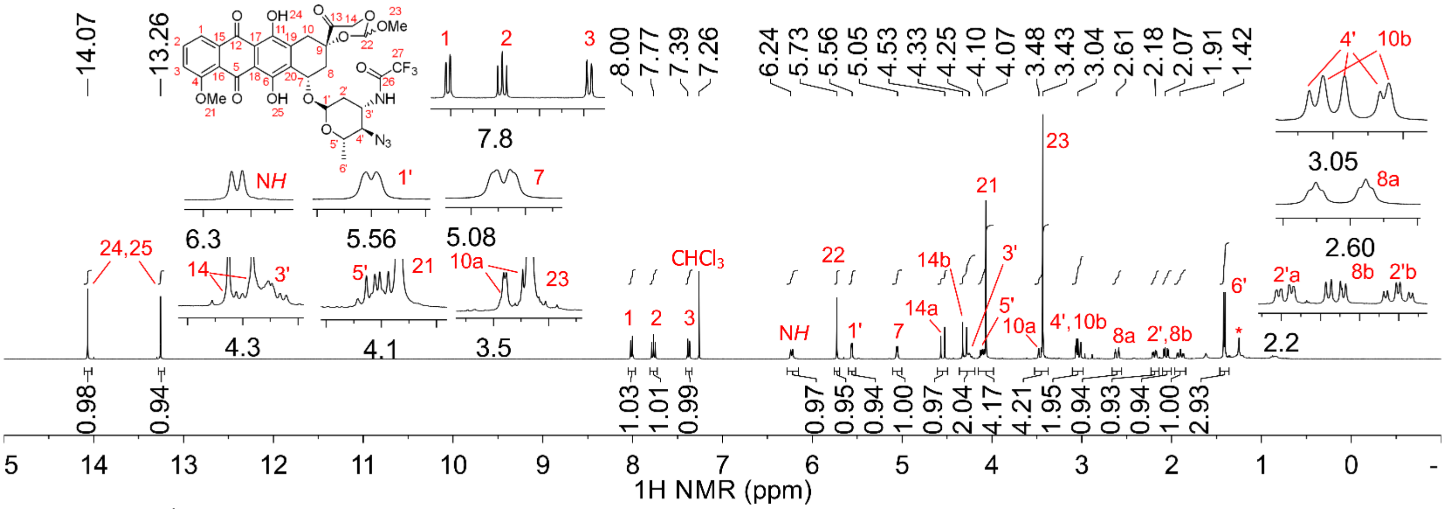

Figure S125. ^{1}H NMR spectrum of (4’*R*)-azido doxorubicin 9,14-cyclic methyl orthoester (**9**) in $CDCl_3$.

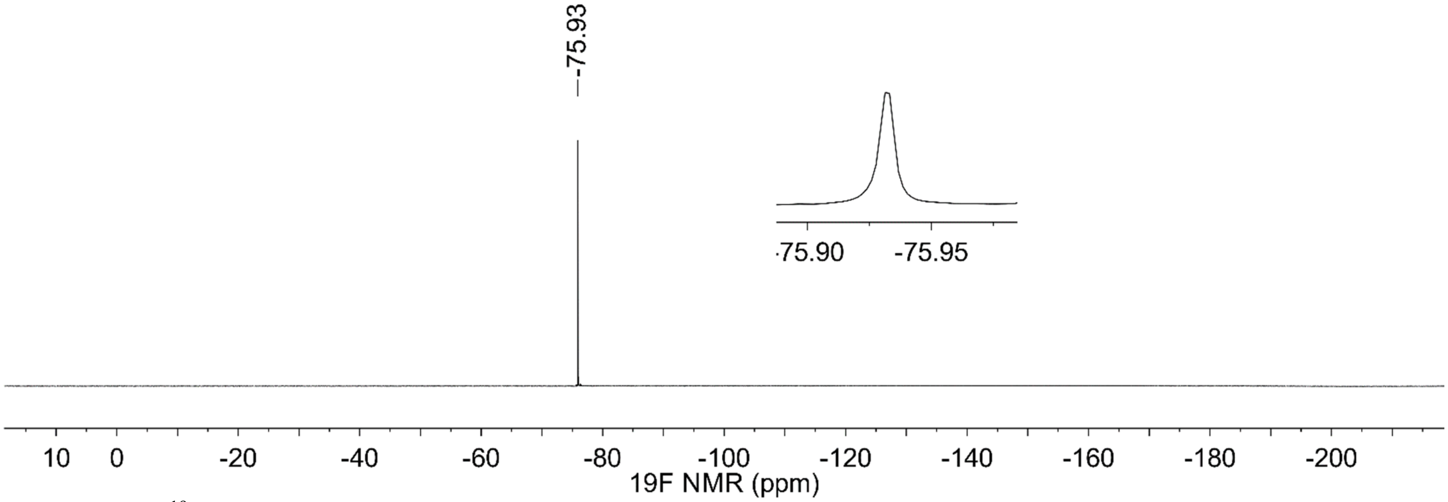

Figure S126. ^{19}F NMR spectrum of (4’*R*)-azido doxorubicin 9,14-cyclic methyl orthoester (**9**) in $CDCl_3$.

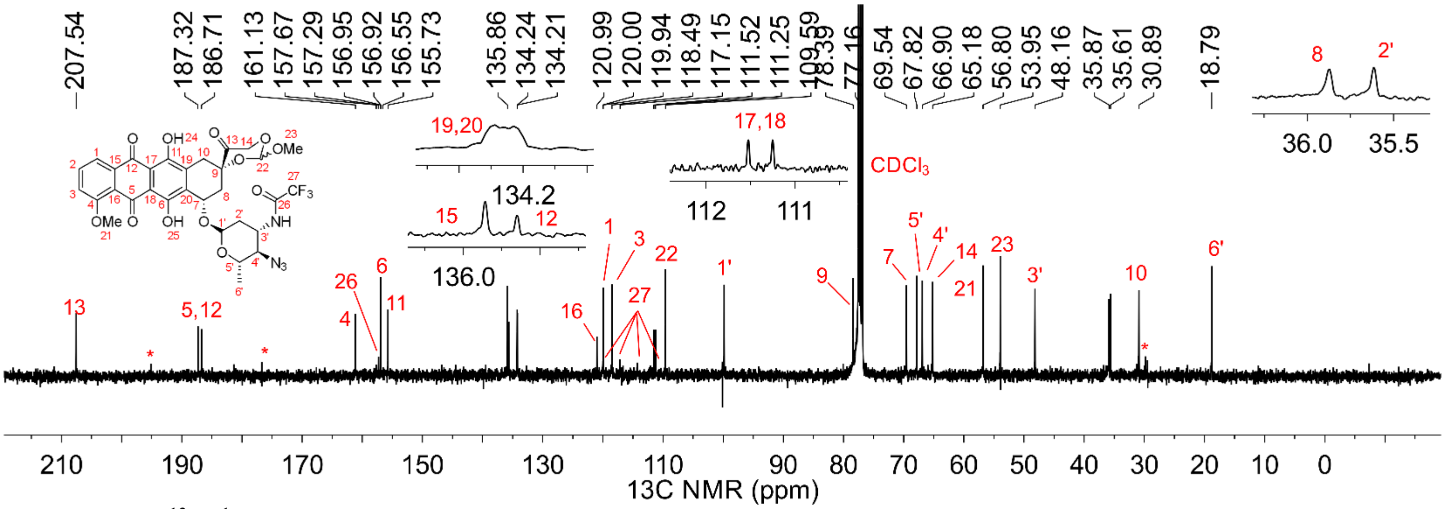

Figure S127. ^{13}C{^{1}H} NMR spectrum of (4’*R*)-azido doxorubicin 9,14-cyclic methyl orthoester (**9**) in $CDCl_3$.

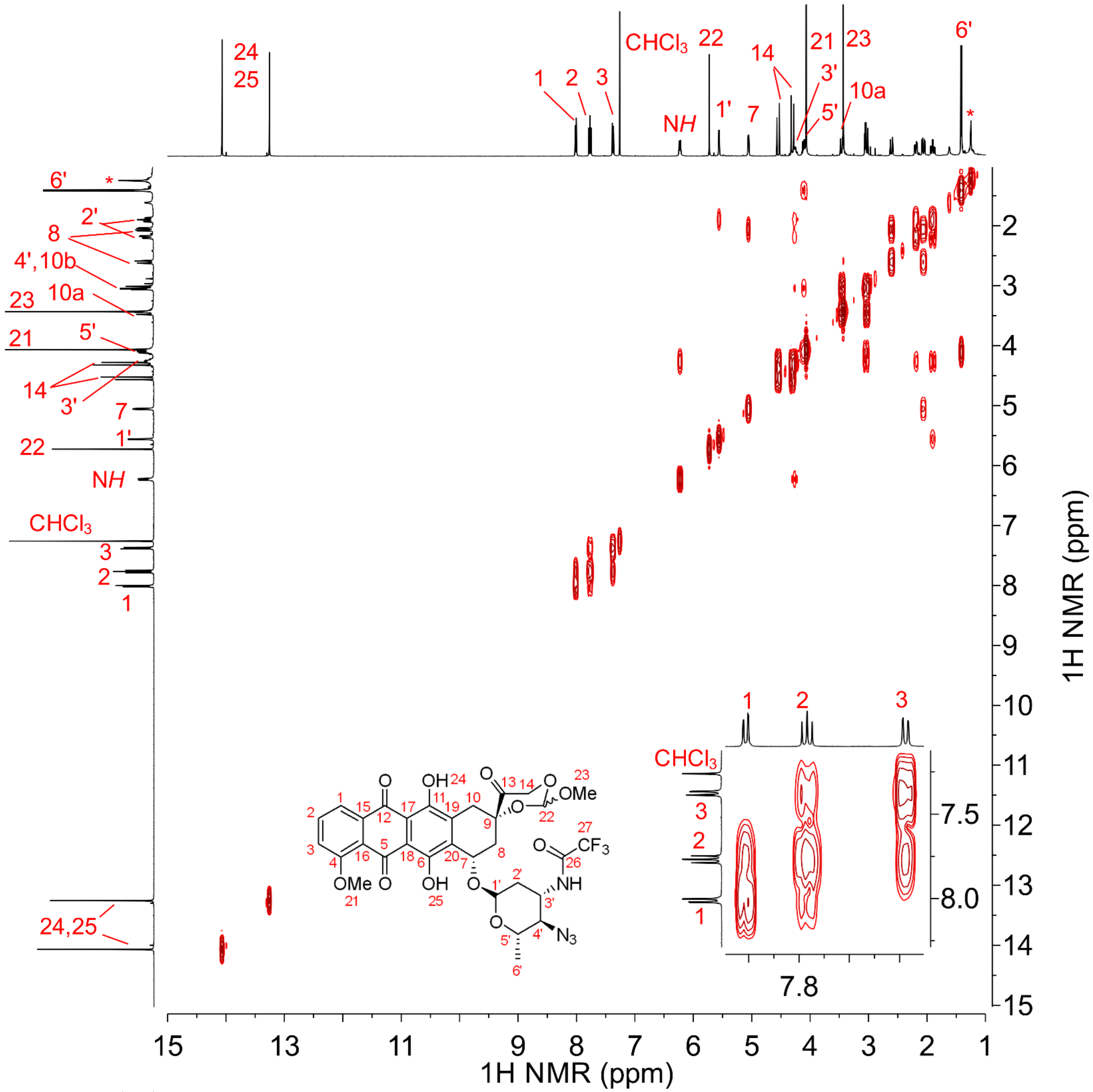

Figure S128. ^{1}H-^{1}H COSY spectrum of (4'*R*)-azido doxorubicin 9,14-cyclic methyl orthoester (**9**) in $CDCl_3$.

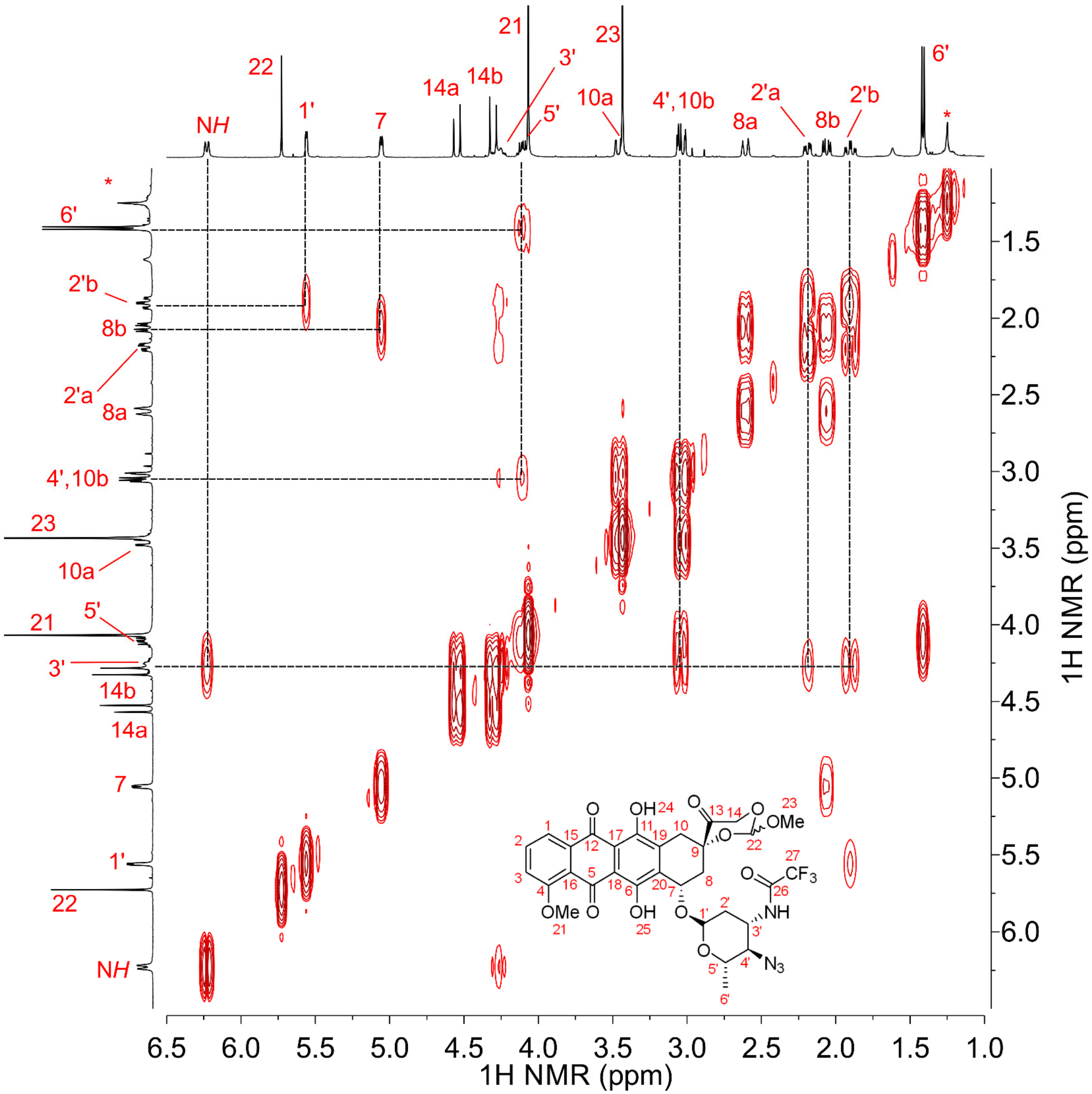

Figure S129. Expansion of ^{1}H-^{1}H COSY spectrum of (4'*R*)-azido doxorubicin 9,14-cyclic methyl orthoester (**9**) in $CDCl_3$ from 1.00 to 6.50 ppm (^{1}H) and 1.00 to 6.50 ppm (^{1}H).

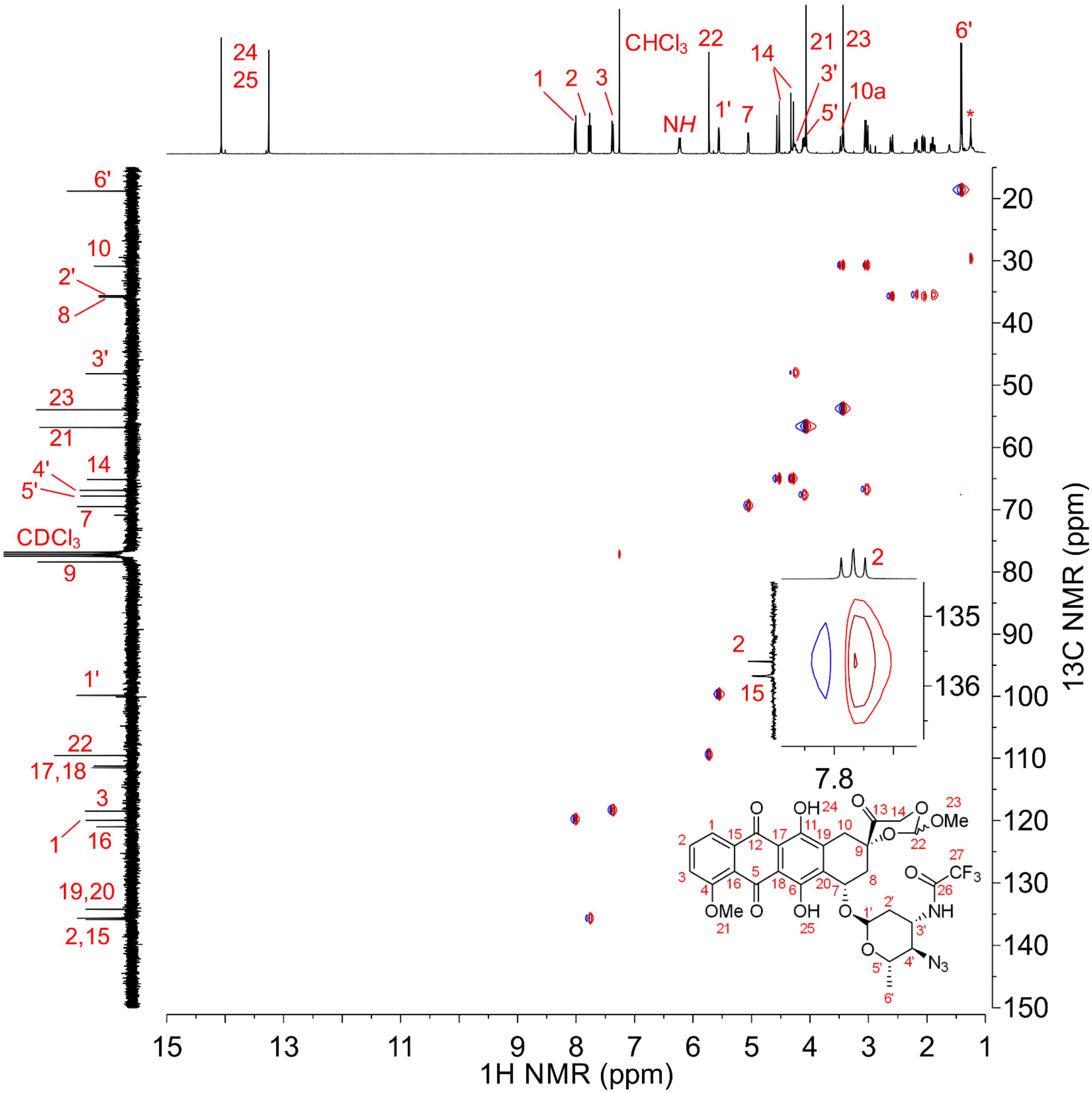

Figure S130. ^{1}H-^{13}C HSQC spectrum of (4'*R*)-azido doxorubicin 9,14-cyclic methyl orthoester (**9**) in $CDCl_3$.

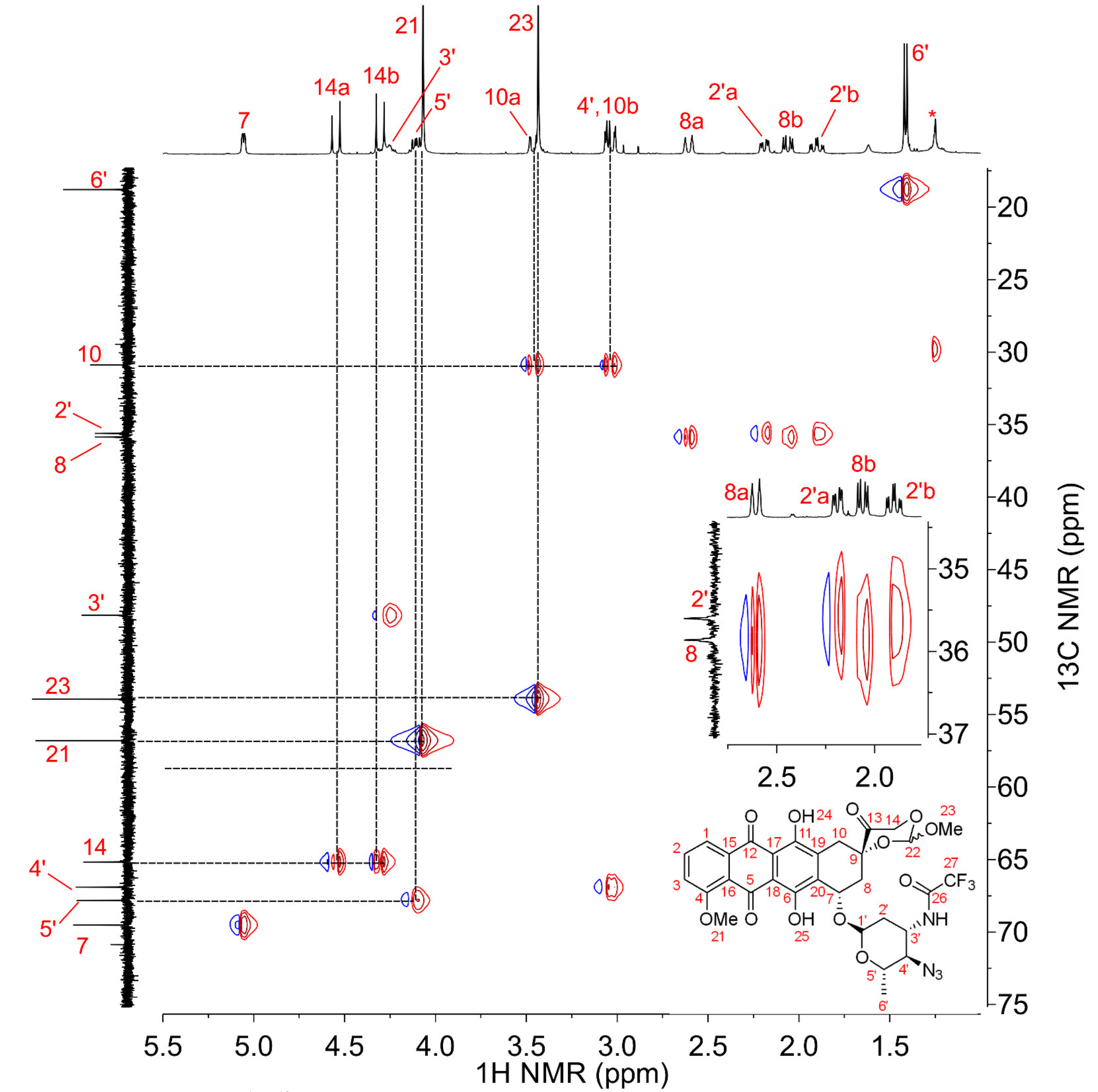

Figure S131. Expansion of ^{1}H-^{13}C HSQC spectrum of (4'*R*)-azido doxorubicin 9,14-cyclic methyl orthoester (**9**) from 1.00 to 5.50 ppm (^{1}H) and 17.5 to 75.0 ppm (^{13}C).

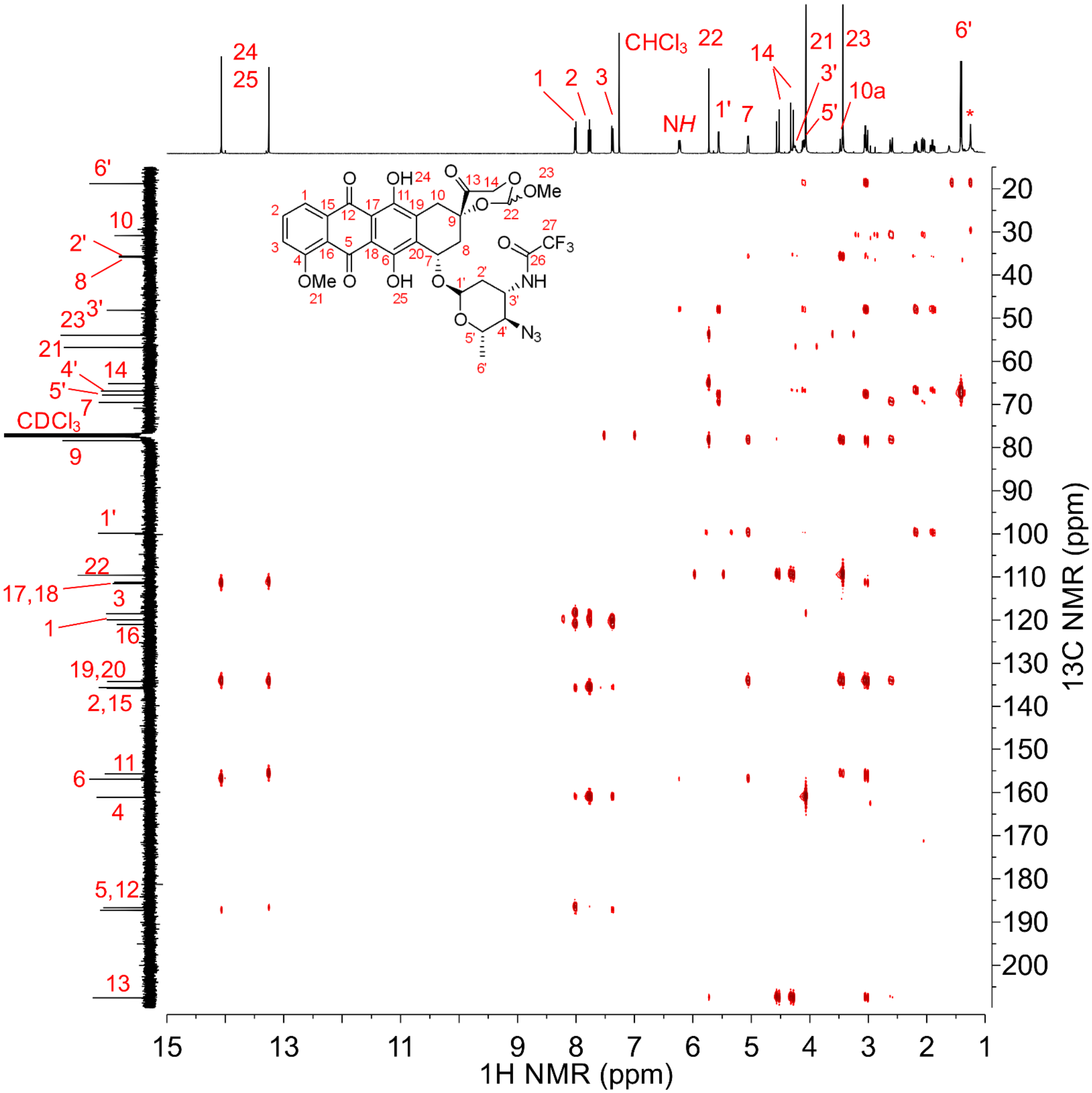

Figure S132. ^{1}H-^{13}C HMBC spectrum of (4'*R*)-azido doxorubicin 9,14-cyclic methyl orthoester (**9**) in $CDCl_3$.

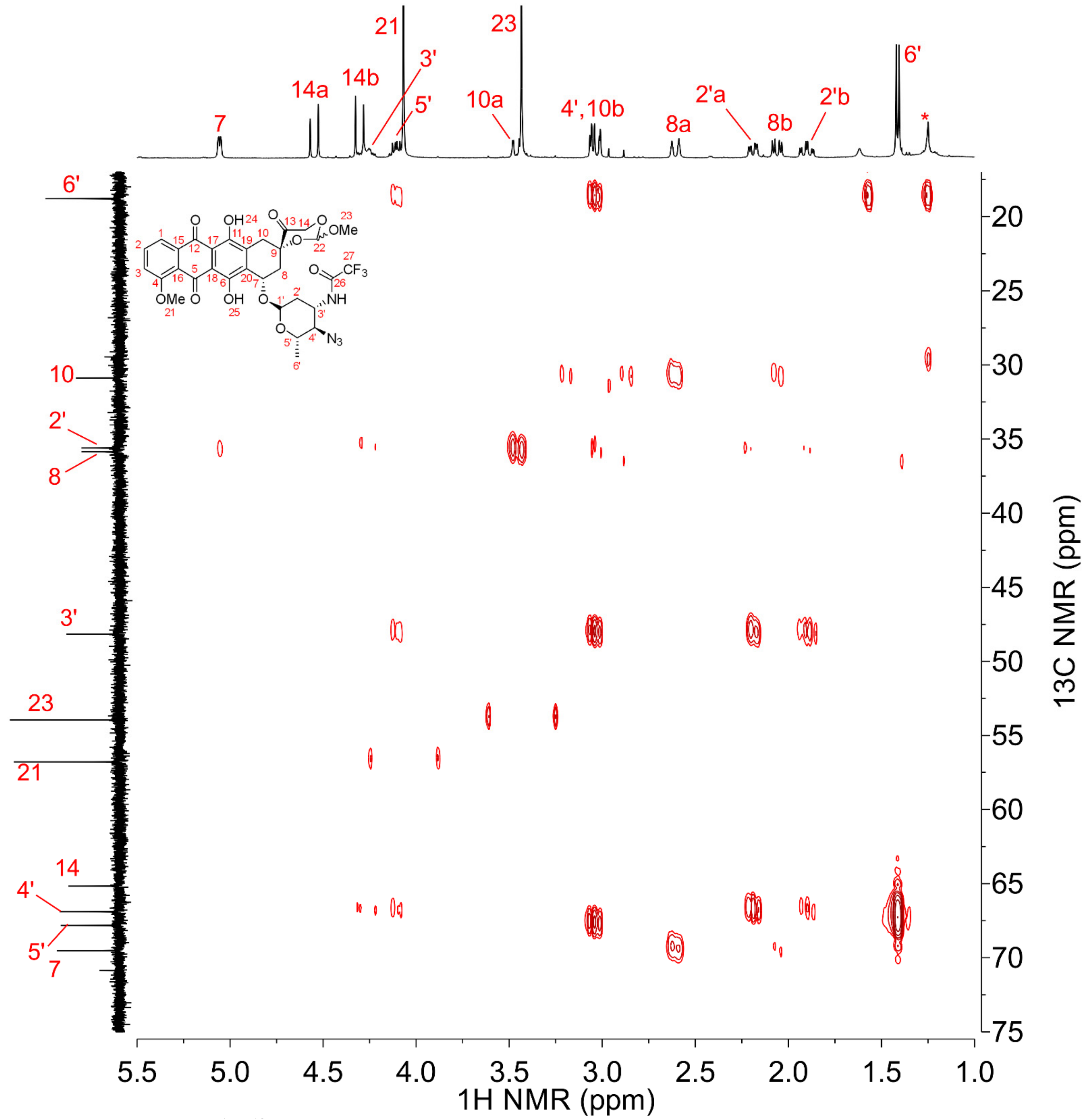

Figure S133. Expansion of ^{1}H-^{13}C HMBC spectrum of (4'*R*)-azido doxorubicin 9,14-cyclic methyl orthoester (**9**) from 1.00 to 5.50 ppm (^{1}H) and 17.5 to 75.0 ppm (^{13}C).

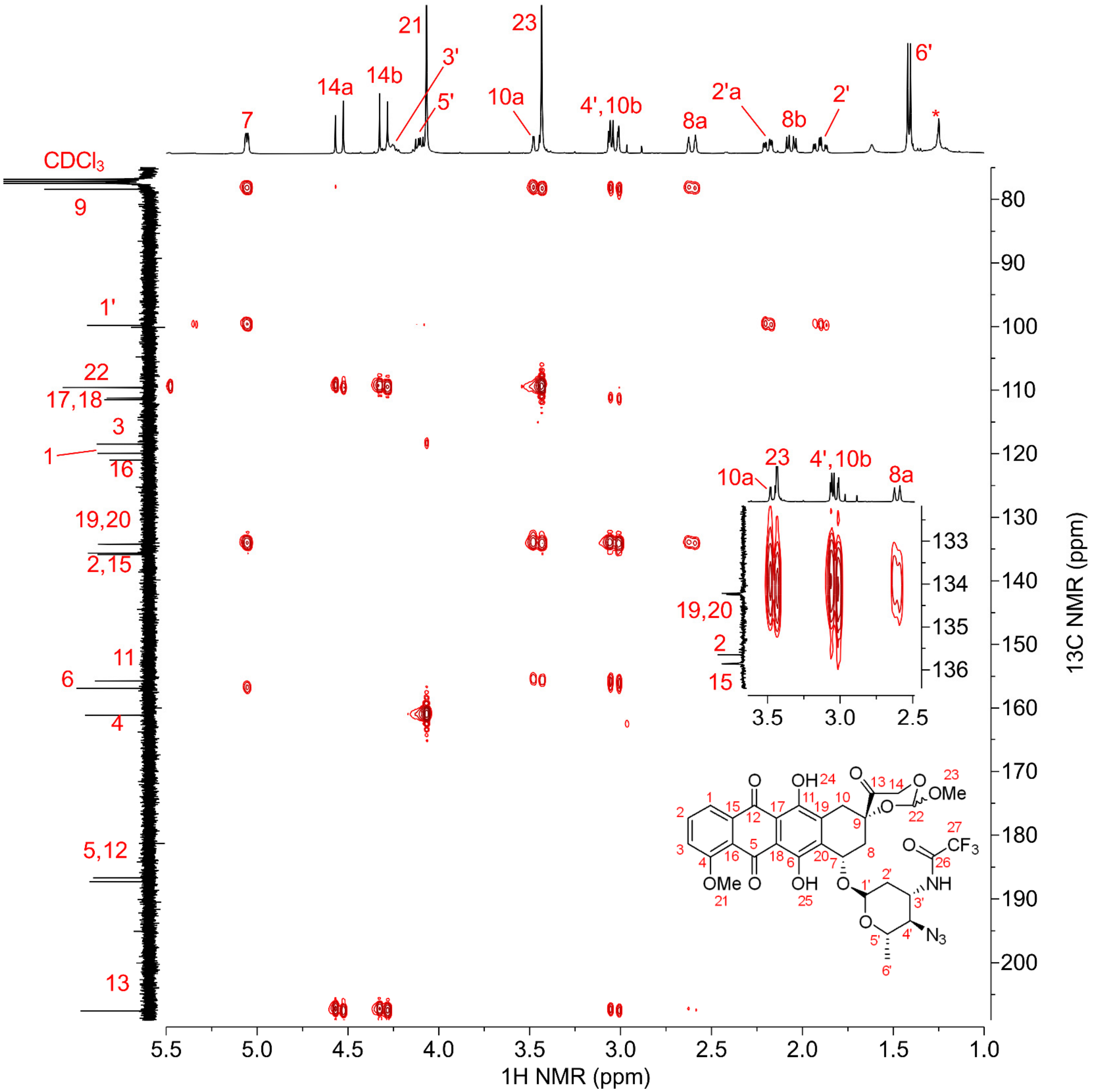

Figure S134. Expansion of ^{1}H-^{13}C HMBC spectrum of (4'*R*)-azido doxorubicin 9,14-cyclic methyl orthoester (**9**) from 1.00 to 5.50 ppm (^{1}H) and 75.0 to 210 ppm (^{13}C).

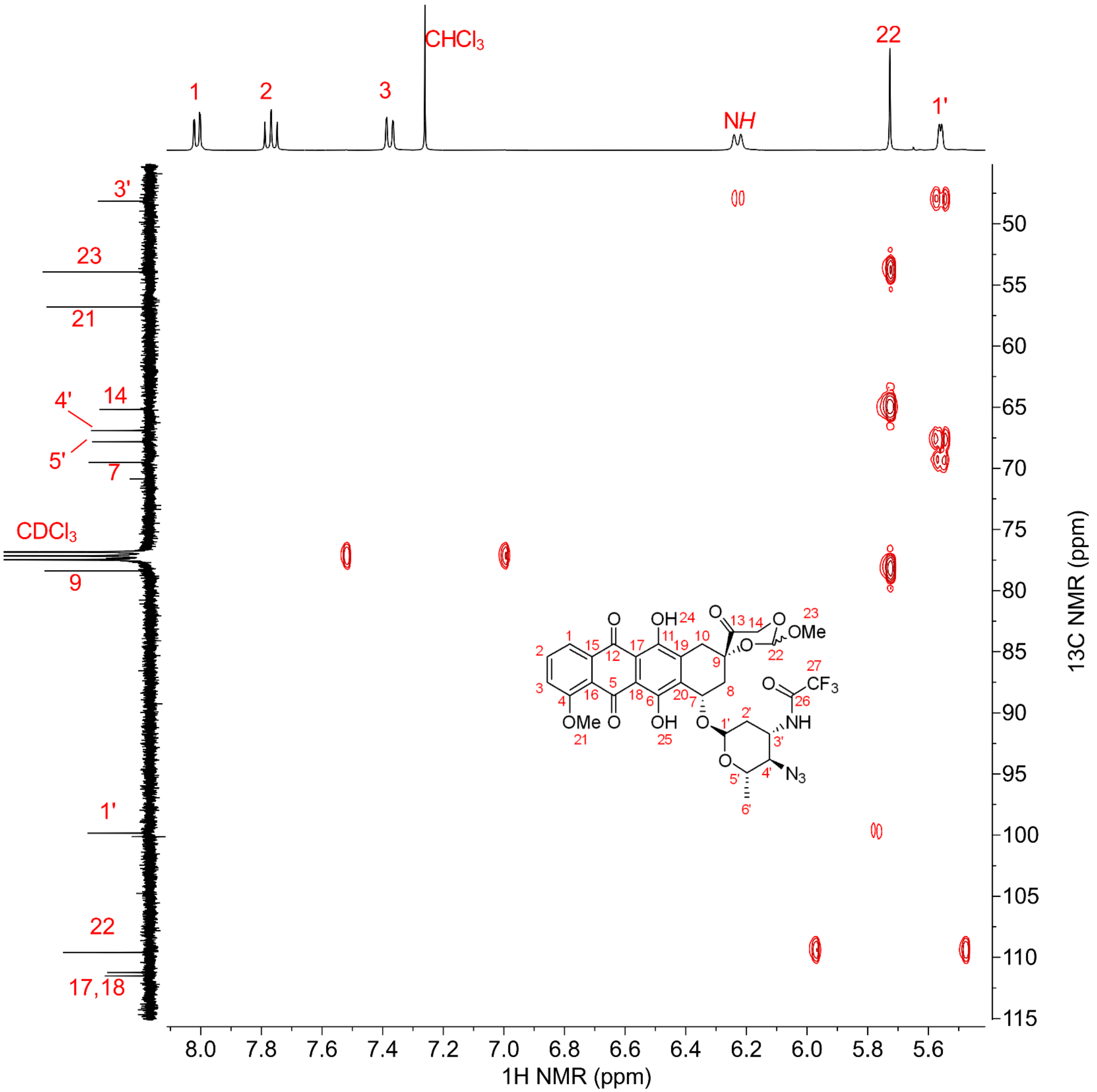

Figure S135. Expansion of ^{1}H-^{13}C HMBC spectrum of (4'*R*)-azido doxorubicin 9,14-cyclic methyl orthoester (**9**) from 5.40 to 8.10 ppm (^{1}H) and 45.0 to 115 ppm (^{13}C).

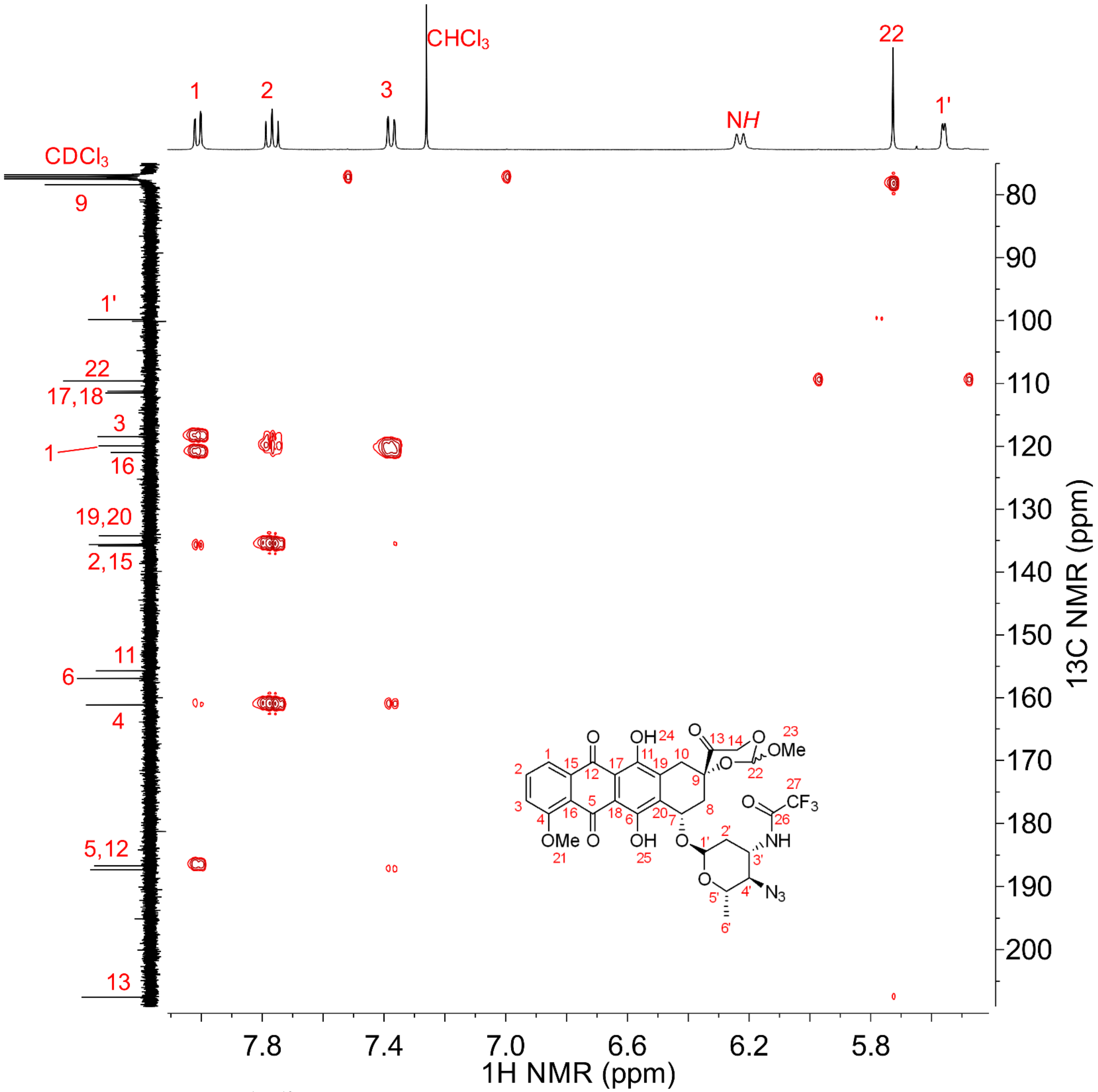

Figure S136. Expansion of ^{1}H-^{13}C HMBC spectrum of (4'*R*)-azido doxorubicin 9,14-cyclic methyl orthoester (**9**) from 5.60 to 8.10 ppm (^{1}H) and 75.0 to 210 ppm (^{13}C).

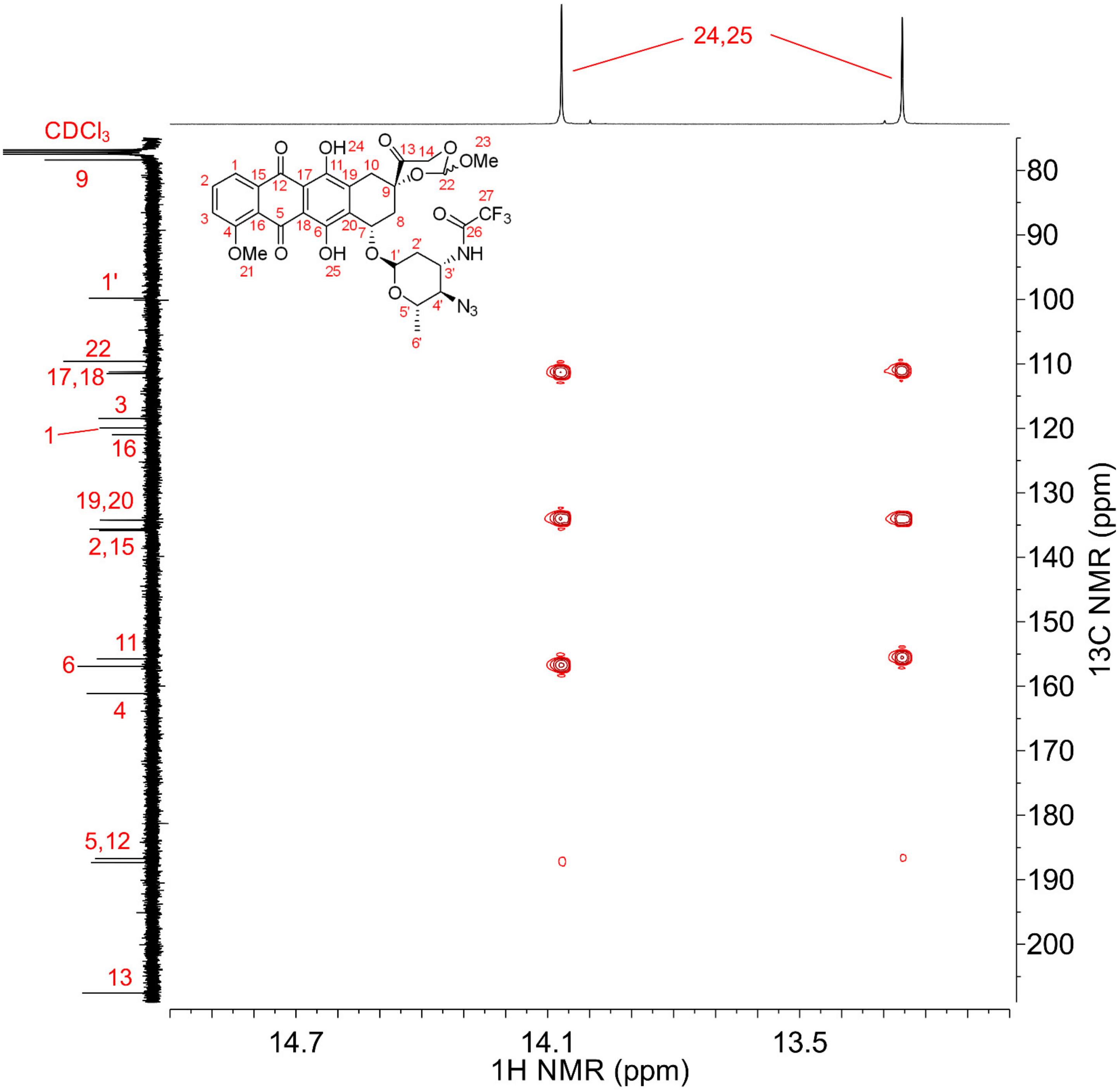

Figure S137. Expansion of ^{1}H-^{13}C HMBC spectrum of (4'*R*)-azido doxorubicin 9,14-cyclic methyl orthoester (**9**) from 13.00 to 15.00 ppm (^{1}H) and 75.0 to 210 ppm (^{13}C).

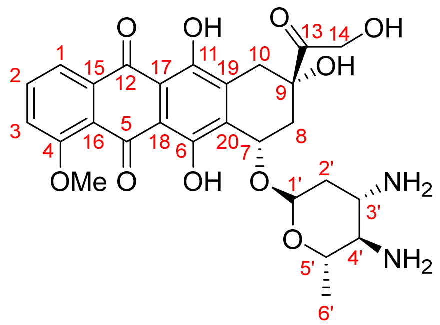

Figure S138. Numbering of DoxNH2NH2 (**1**).

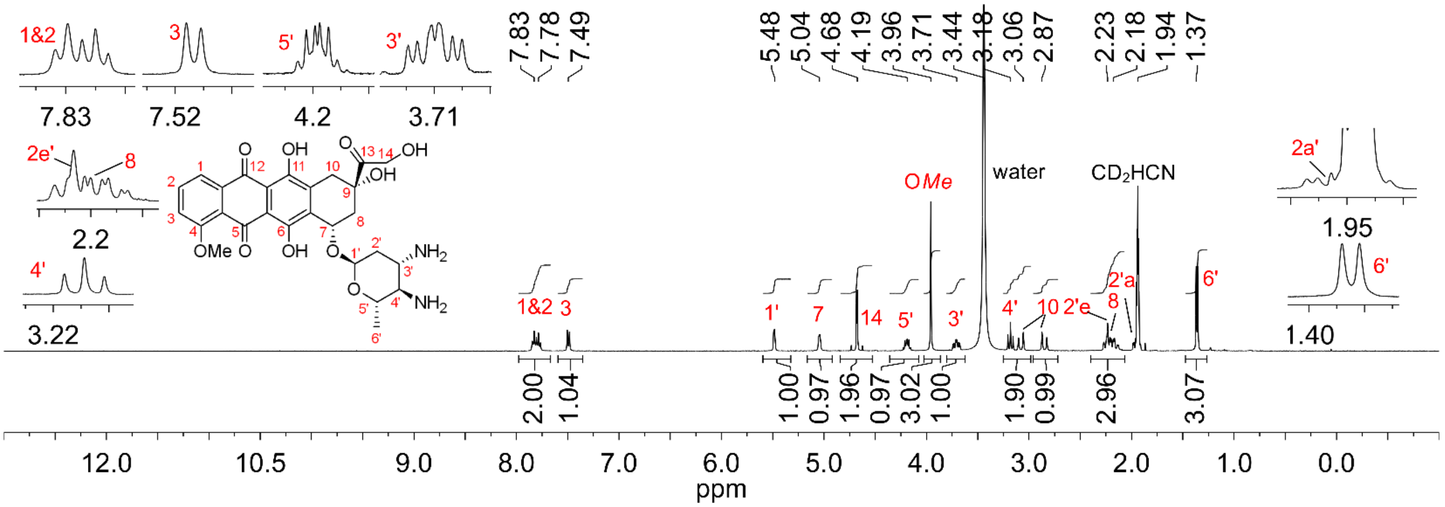

Figure S139. ^{1}H NMR spectrum of DoxNH2NH2 (**6**) in a CD_3CN-D_2O mixture.

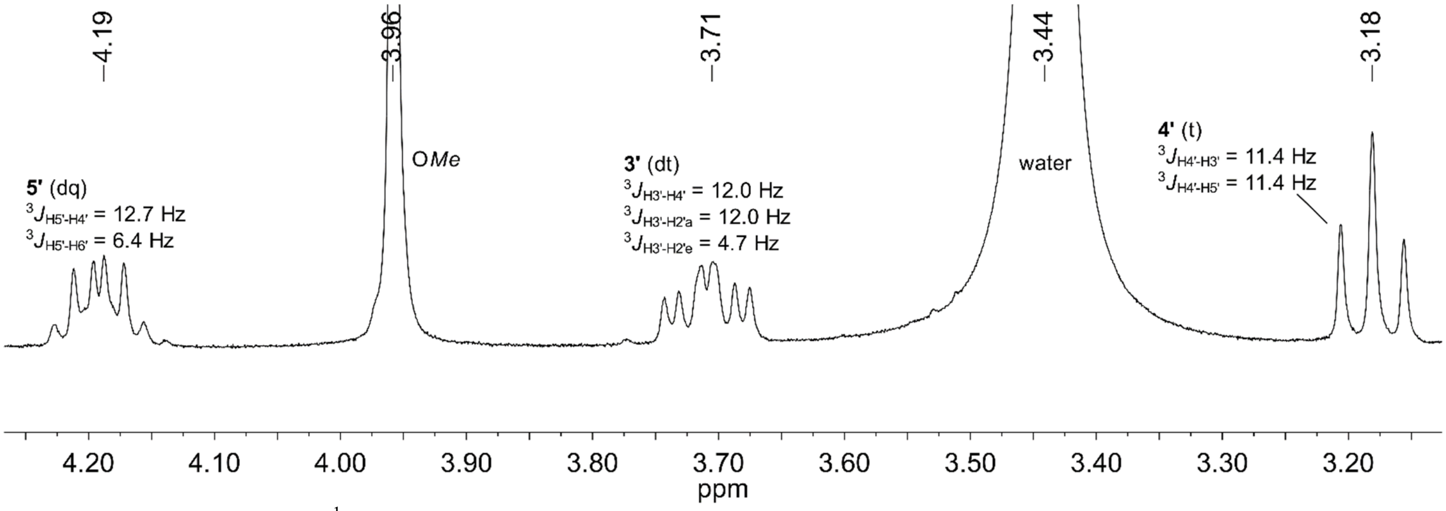

Figure S140. Expansion of ^{1}H NMR spectrum of DoxNH2NH2 (**1**) in a CD_3CN-D_2O mixture from 3.1 to 4.3 ppm.

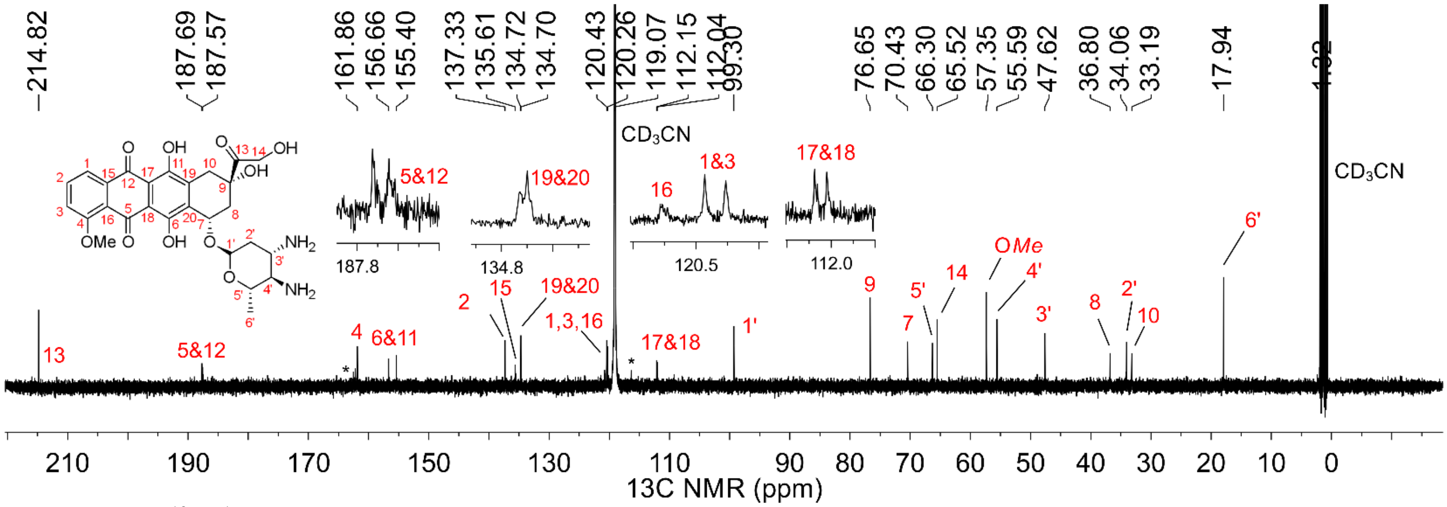

Figure S141. $^{13}C\{^1H\}$ NMR spectrum of $DoxNH_2NH_2$ (**1**) in a CD_3CN-D_2O mixture.

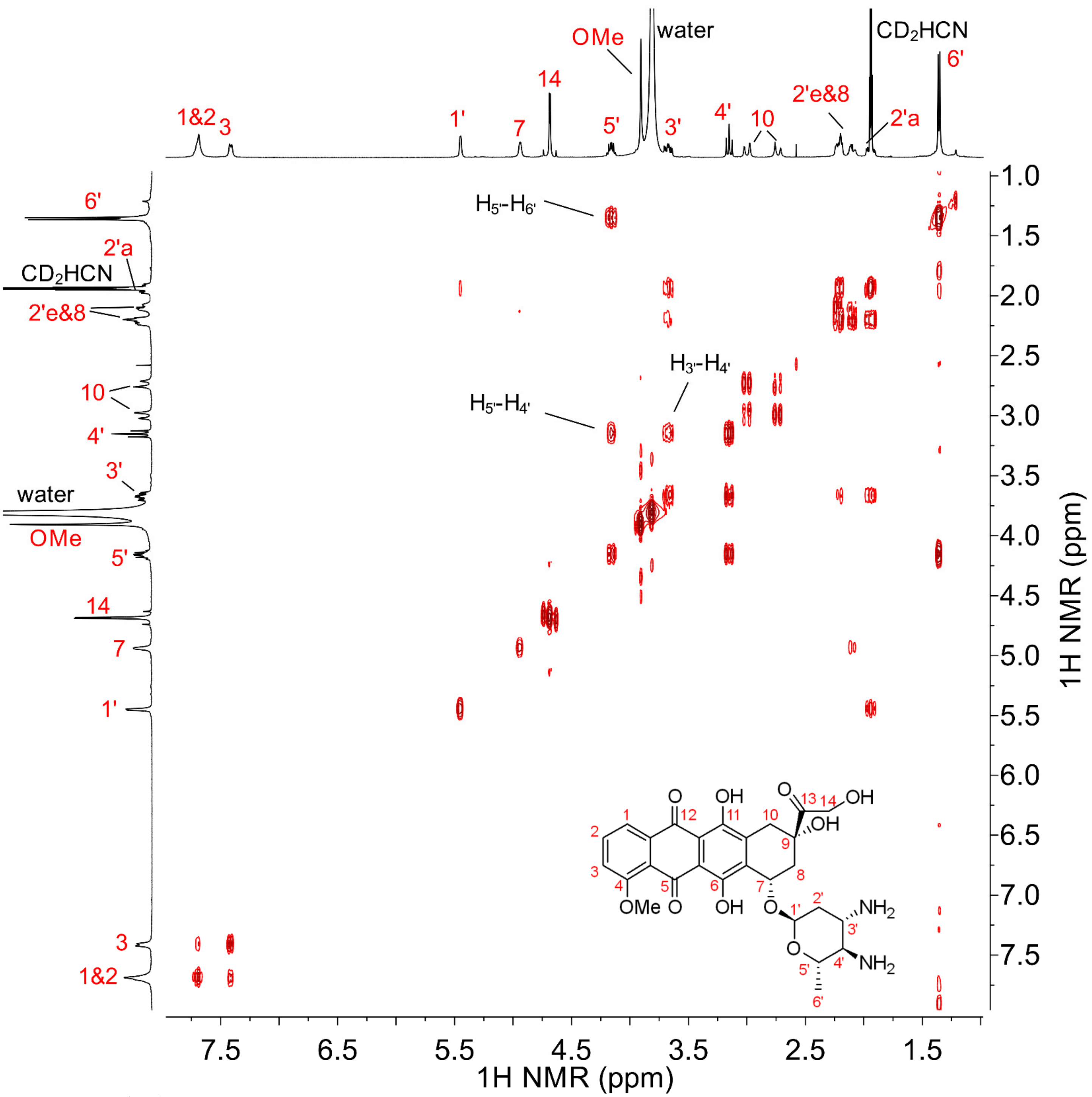

Figure S142. ^1H-^1H COSY spectrum of $DoxNH_2NH_2$ (**1**) in a CD_3CN-D_2O mixture.

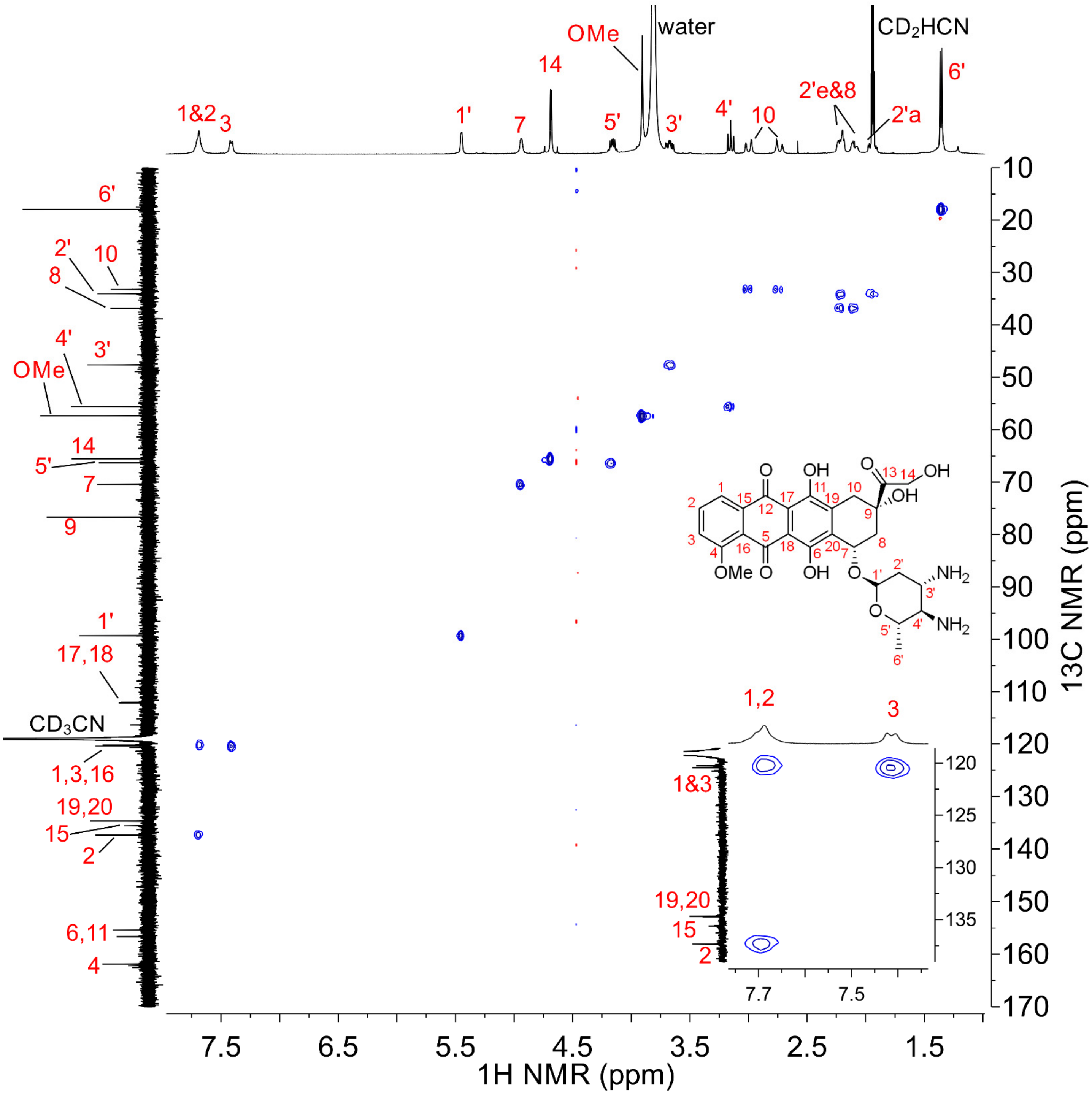

Figure S143. ^{1}H-^{13}C HSQC spectrum of $DoxNH_2NH_2$ (**1**) in a CD_3CN-D_2O mixture.

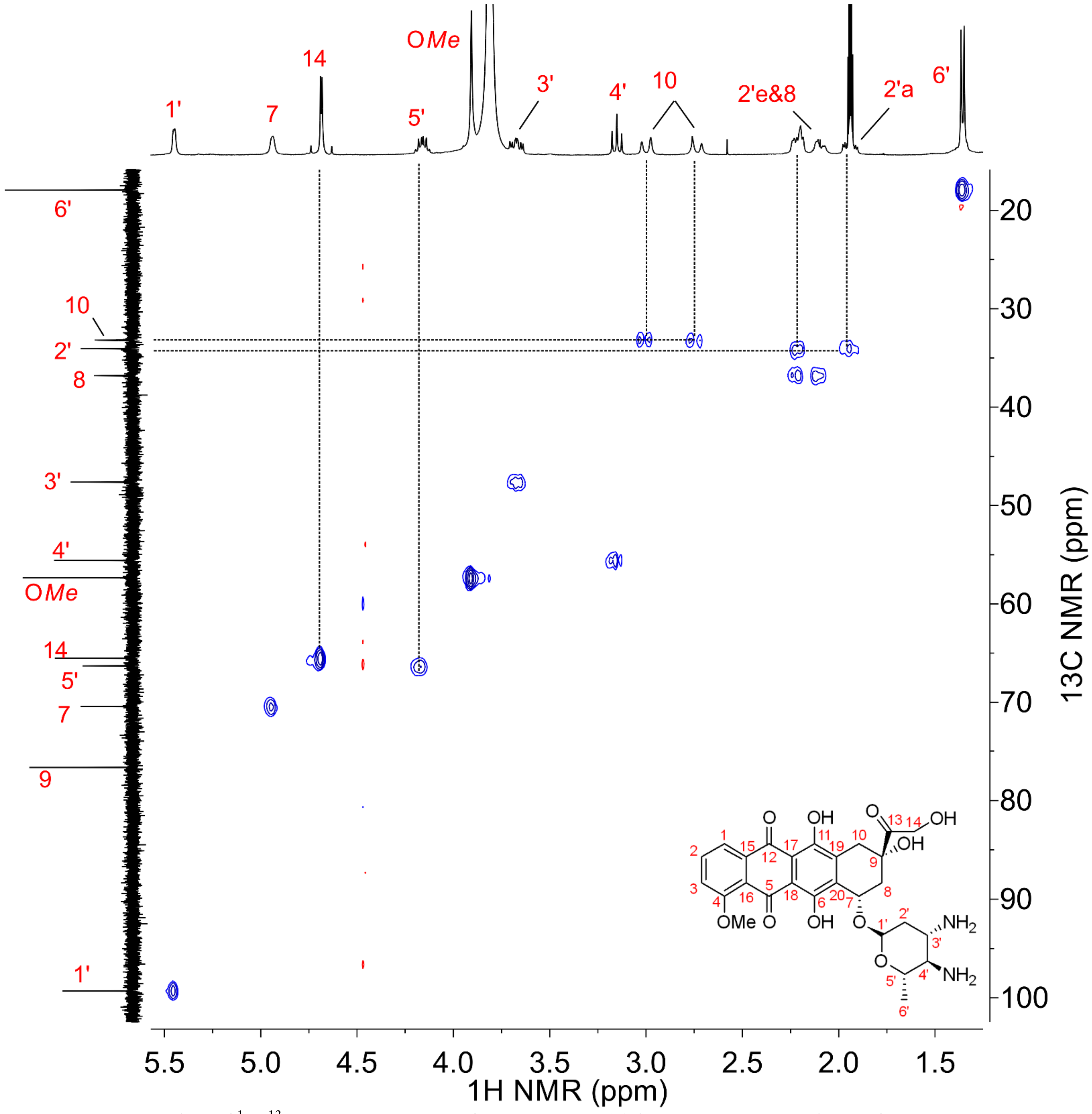

Figure S144. Expansion of ^{1}H-^{13}C HSQC spectrum of $DoxNH_2NH_2$ (**1**) in a CD_3CN-D_2O mixture from 1.25 to 5.50 ppm (^{1}H) and 15.0 to 105 ppm (^{13}C).

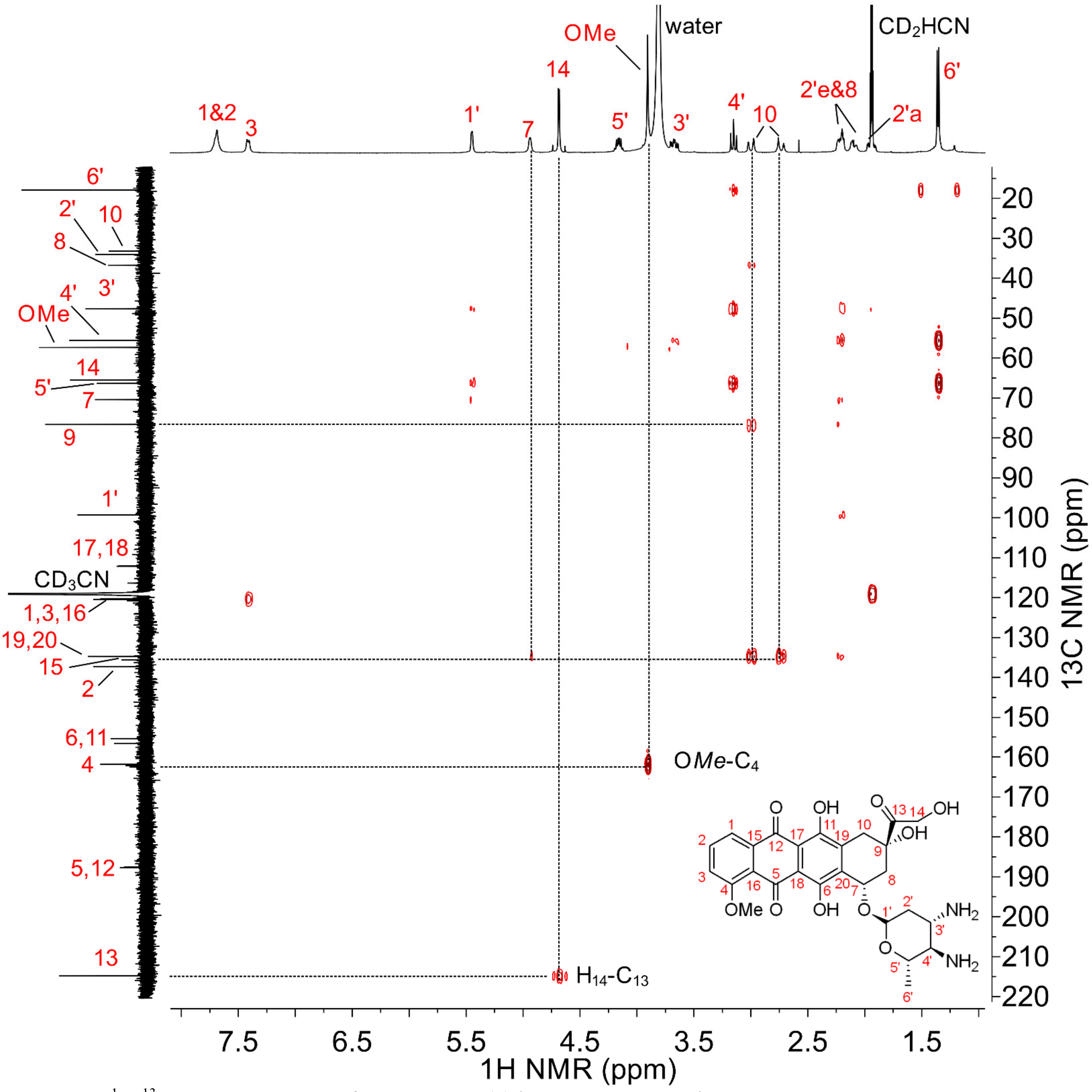

Figure S145. ^{1}H-^{13}C HMBC spectrum of $DoxNH_2NH_2$ (**1**) in a CD_3CN-D_2O mixture.

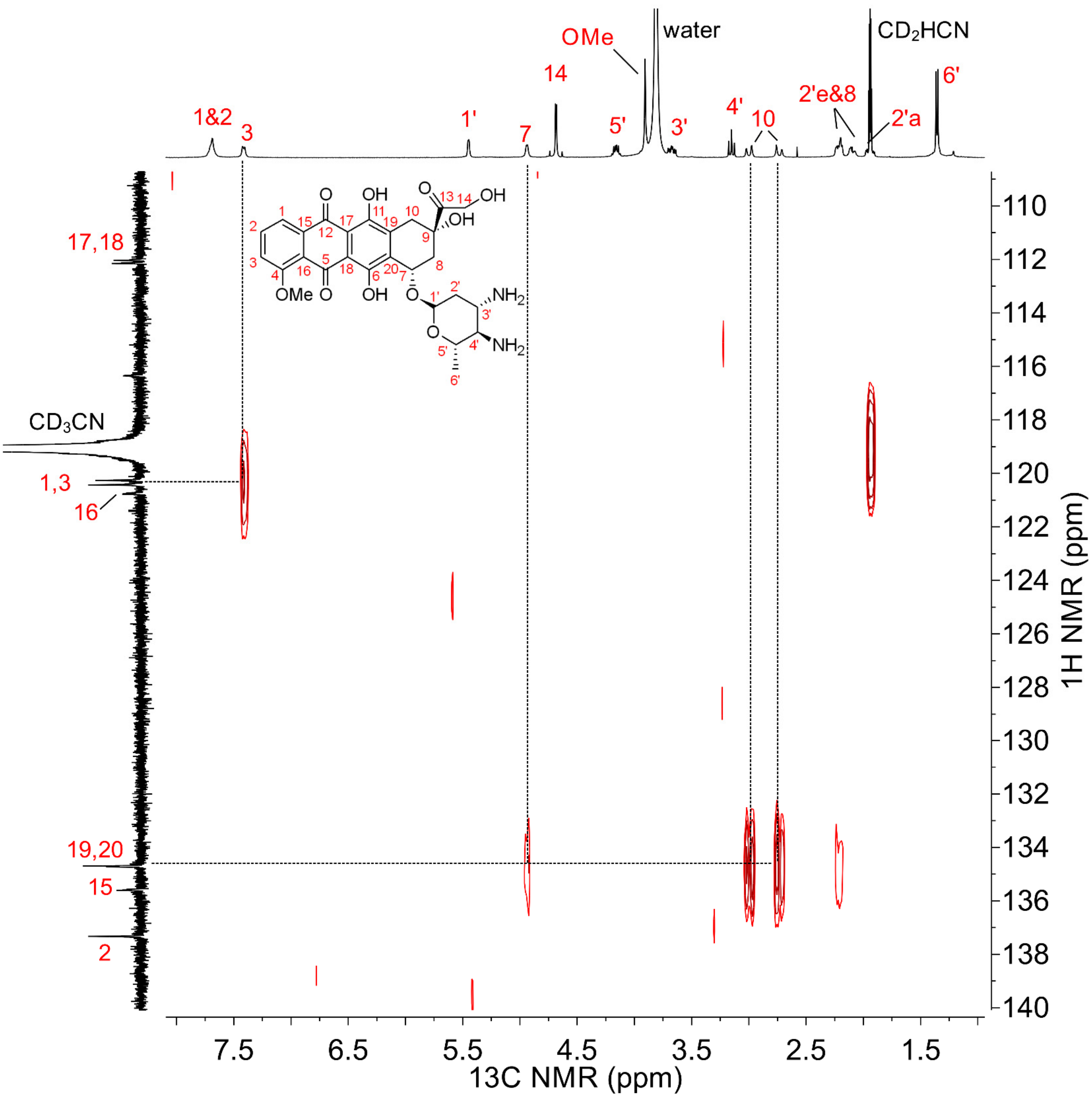

Figure S146. Expansion of ^{1}H-^{13}C HMBC spectrum of DoxNH$_2$NH$_2$ (**1**) in a CD$_3$CN-D$_2$O mixture from 1.0 to 8.0 ppm (^{1}H) and 110 to 140 ppm (^{13}C).

Conformer A

^{1}H NMR

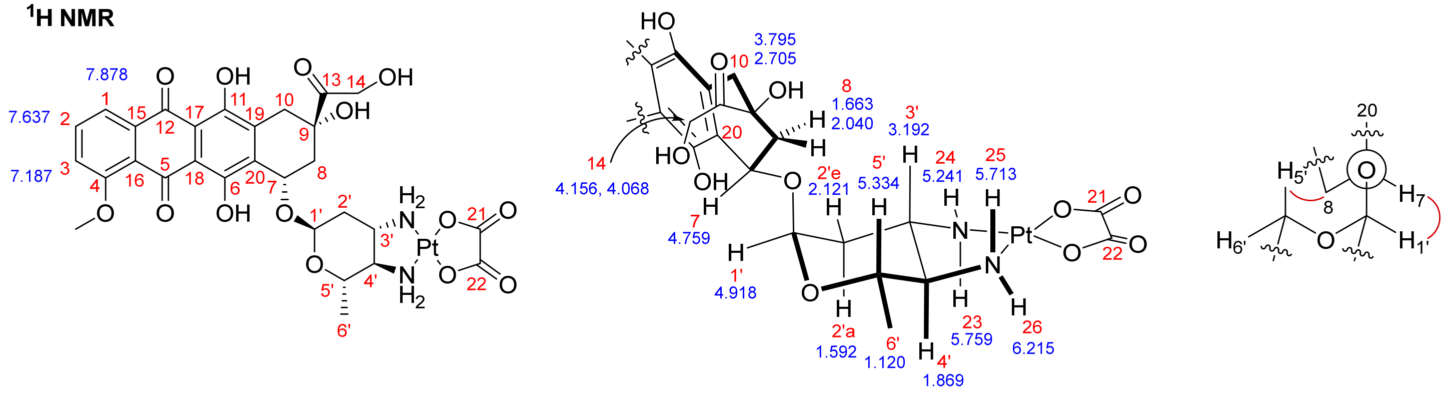

Conformer B

^{1}H NMR

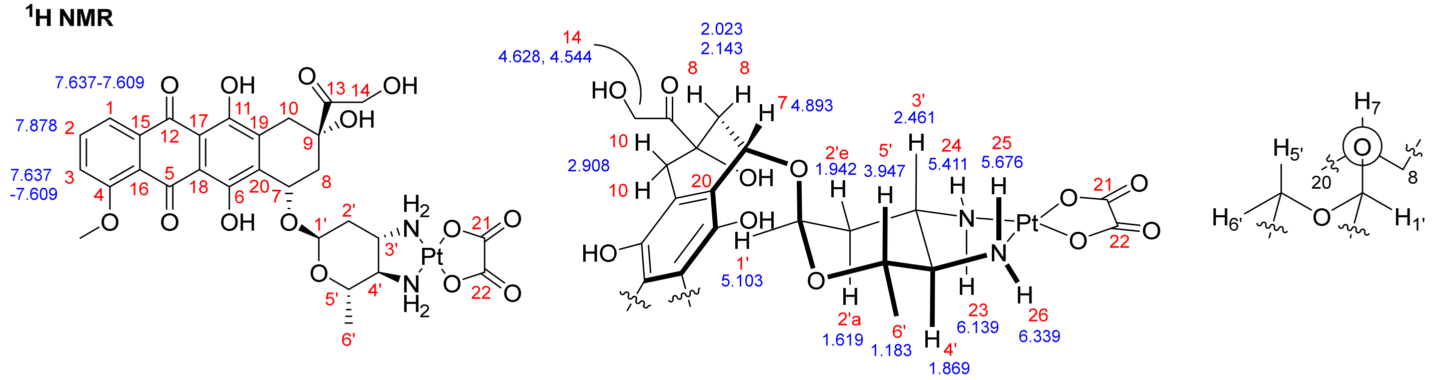

Figure S147. Numbering of two conformers of doxaliplatin (DoxPt) in DMSO-d_6 detected by NMR spectroscopy. The red curves indicate observed NOE signals crucial for determining the conformations. The ^{1}H NMR chemical shift of each proton was indicated in blue.

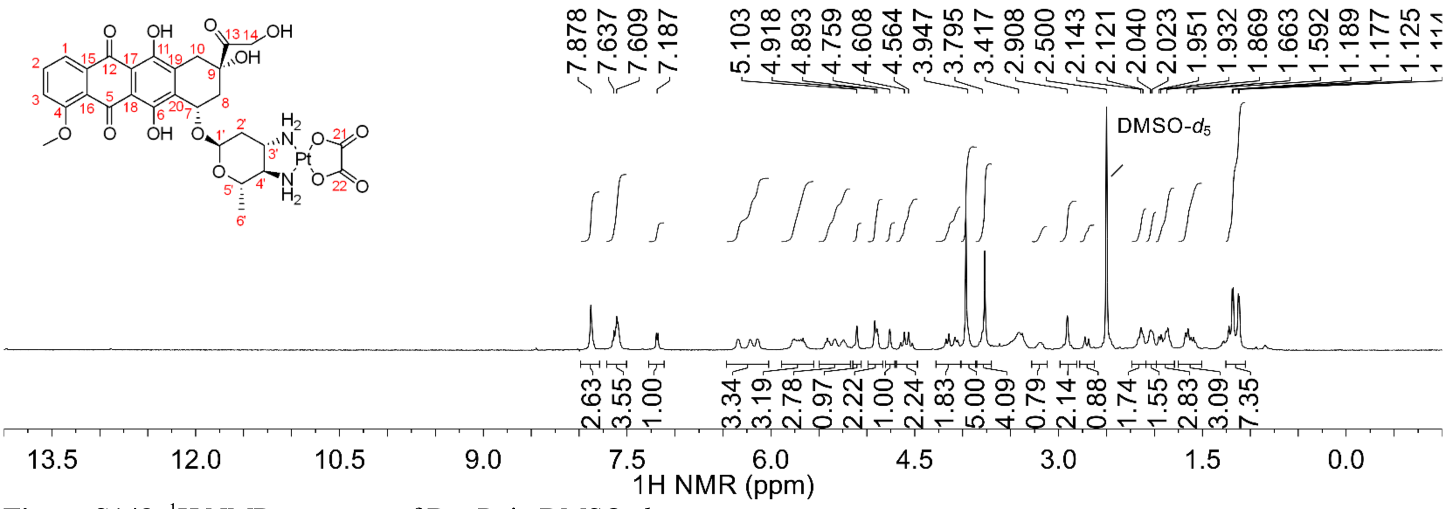

Figure S148. ^{1}H NMR spectrum of DoxPt in DMSO-d_6.

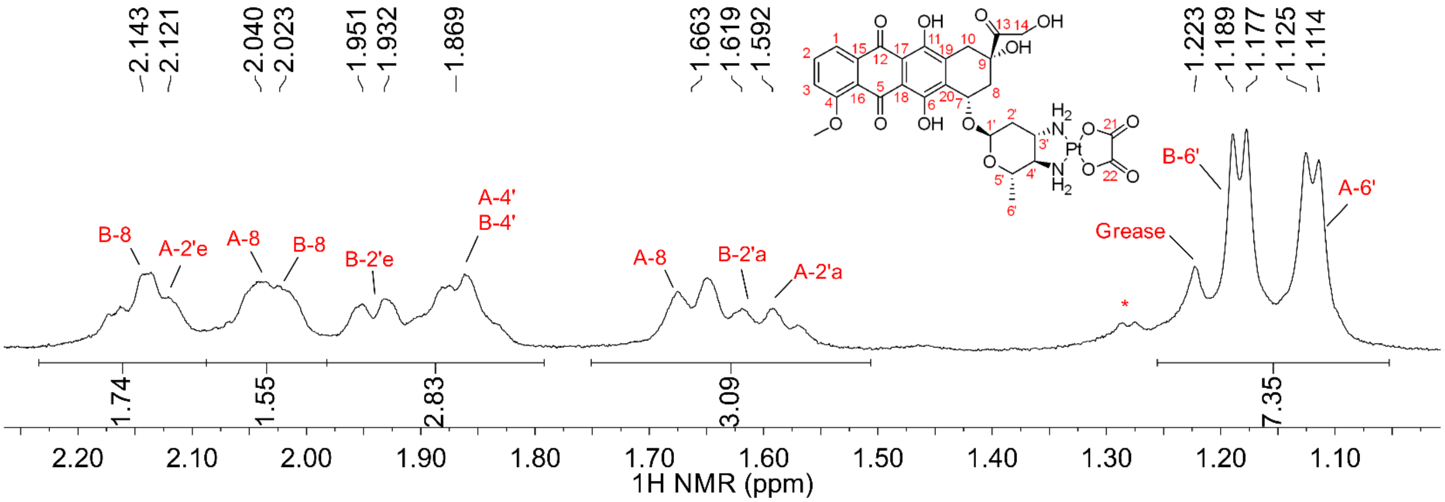

Figure S149. Expansion of ^{1}H NMR spectrum of DoxPt in DMSO-d_6 from 1.00 to 2.25 ppm.

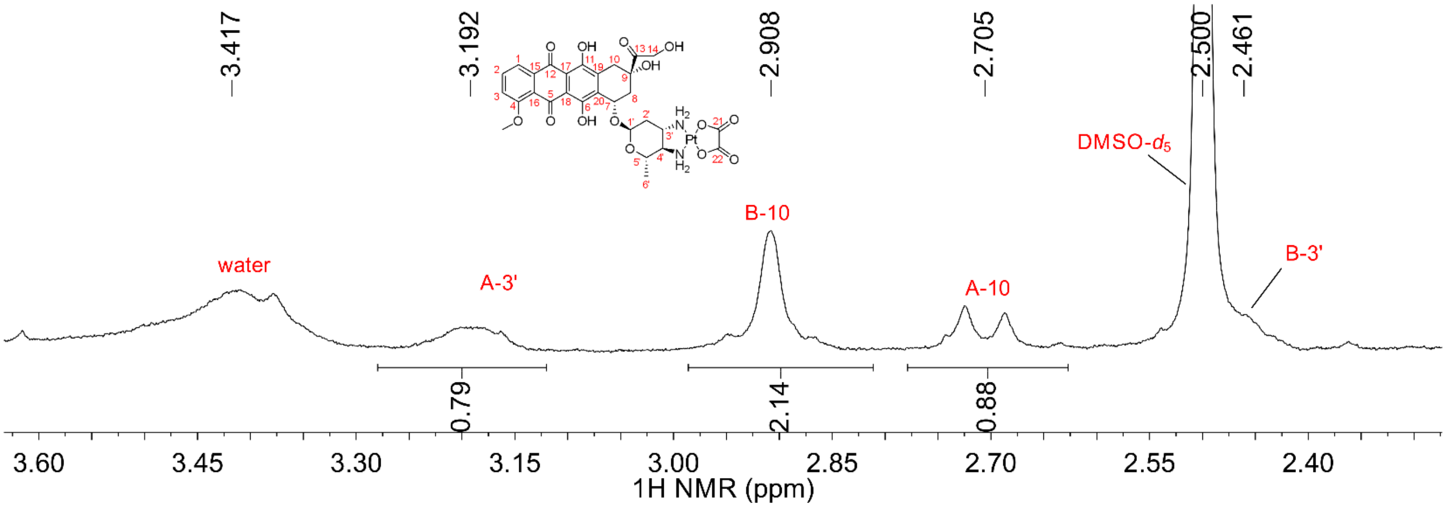

Figure S150. Expansion of ^{1}H NMR spectrum of DoxPt in DMSO-d_6 from 2.30 to 3.60 ppm.

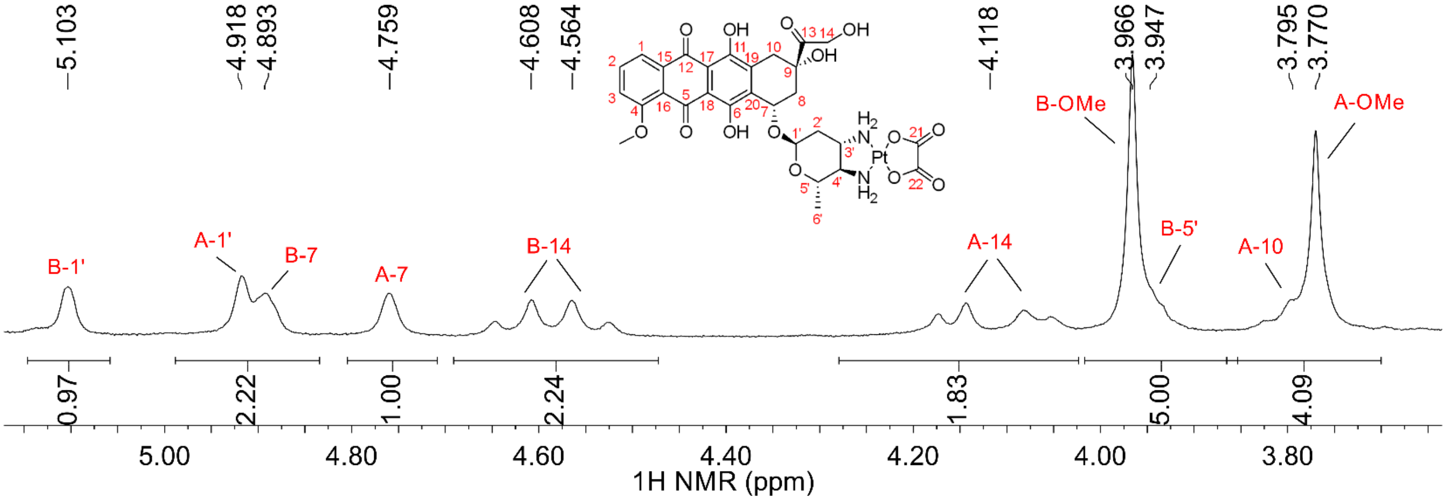

Figure S151. Expansion of ^{1}H NMR spectrum of DoxPt in DMSO-d_6 from 3.65 to 5.15 ppm.

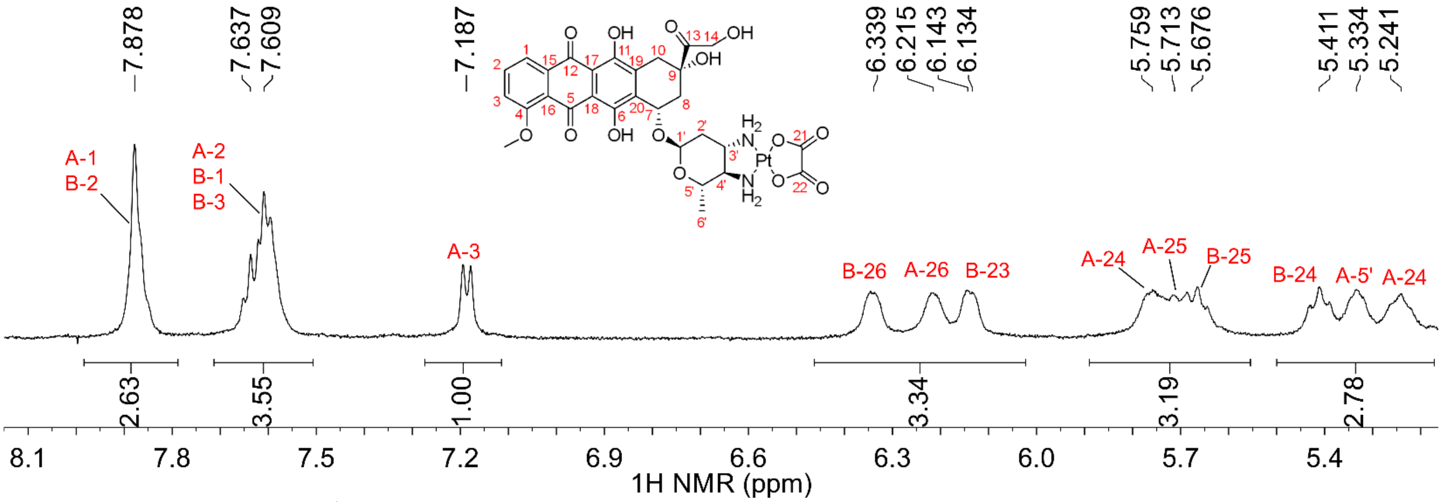

Figure S152. Expansion of ^{1}H NMR spectrum of DoxPt in DMSO-d_6 from 5.15 to 8.15 ppm.

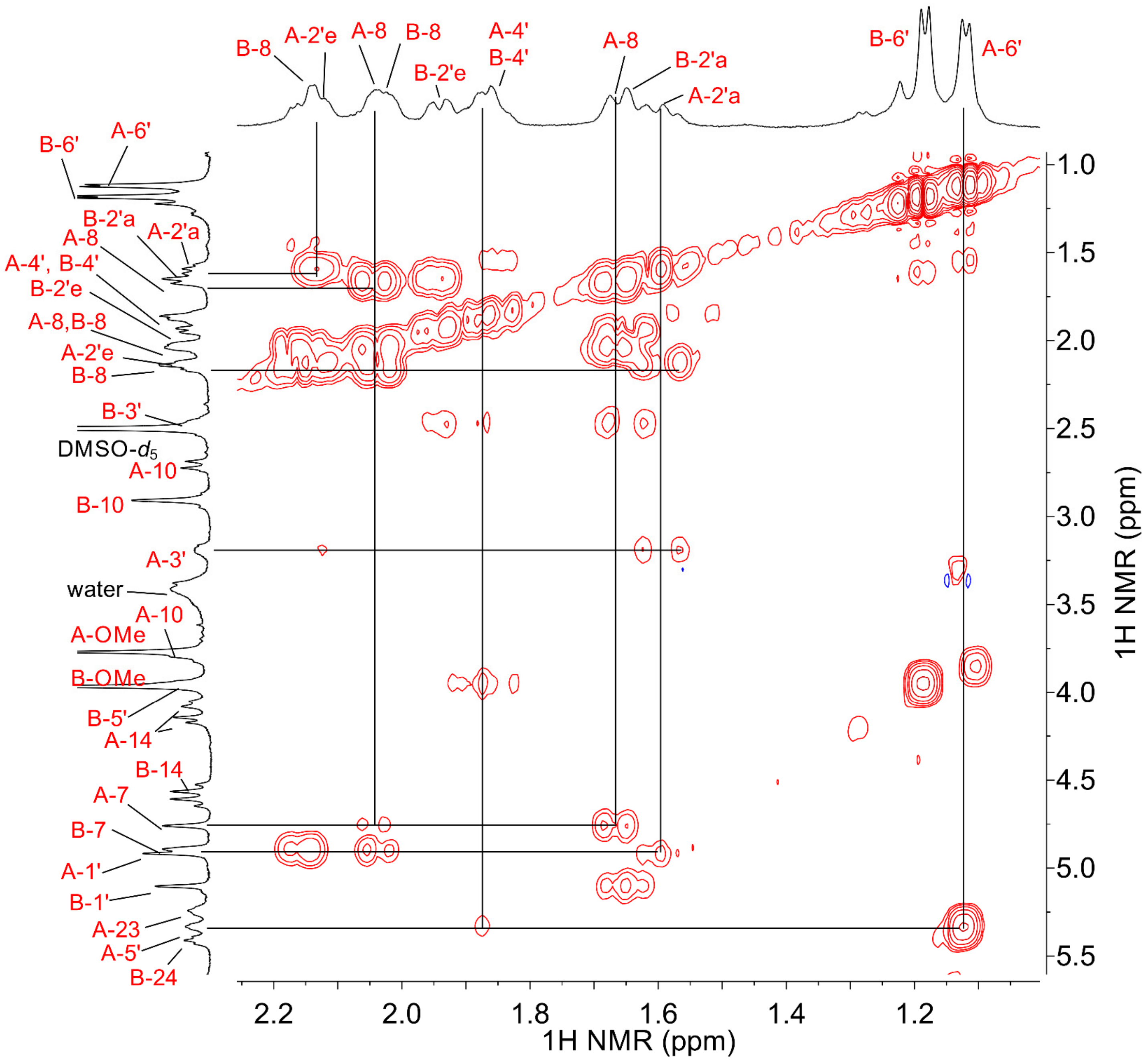

Figure S153. Expansion of ^{1}H-^{1}H COSY spectrum of DoxPt in DMSO-d_6 from 1.0 to 5.5 ppm (f1) and 1.0 to 2.3 ppm (f2). Crucial correlations related to Conformer A are indicated.

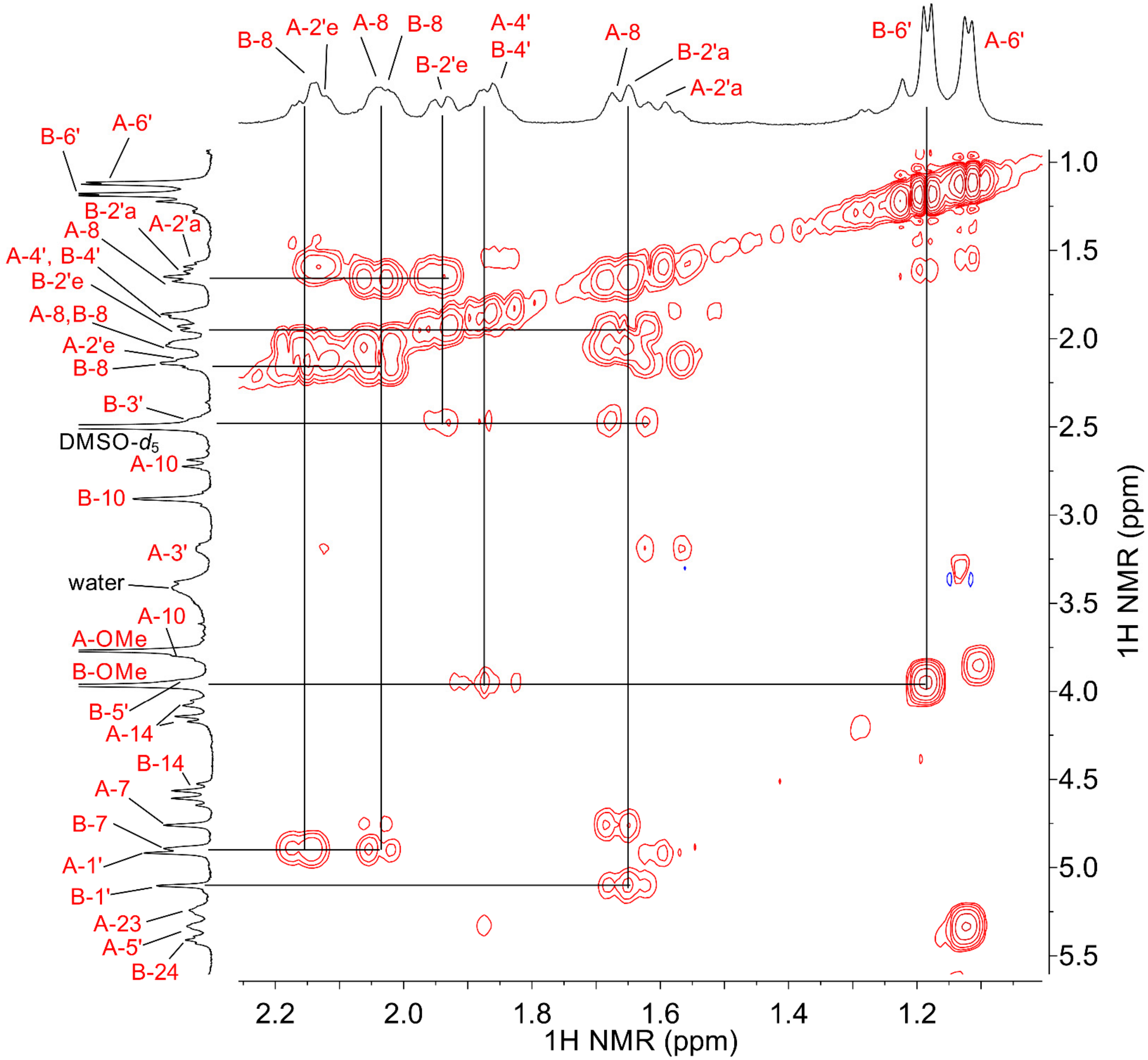

Figure S154. Expansion of ^{1}H-^{1}H COSY spectrum of DoxPt in DMSO-d_6 from 1.0 to 5.5 ppm (f1) and 1.0 to 2.3 ppm (f2). Crucial correlations related to Conformer B are indicated.

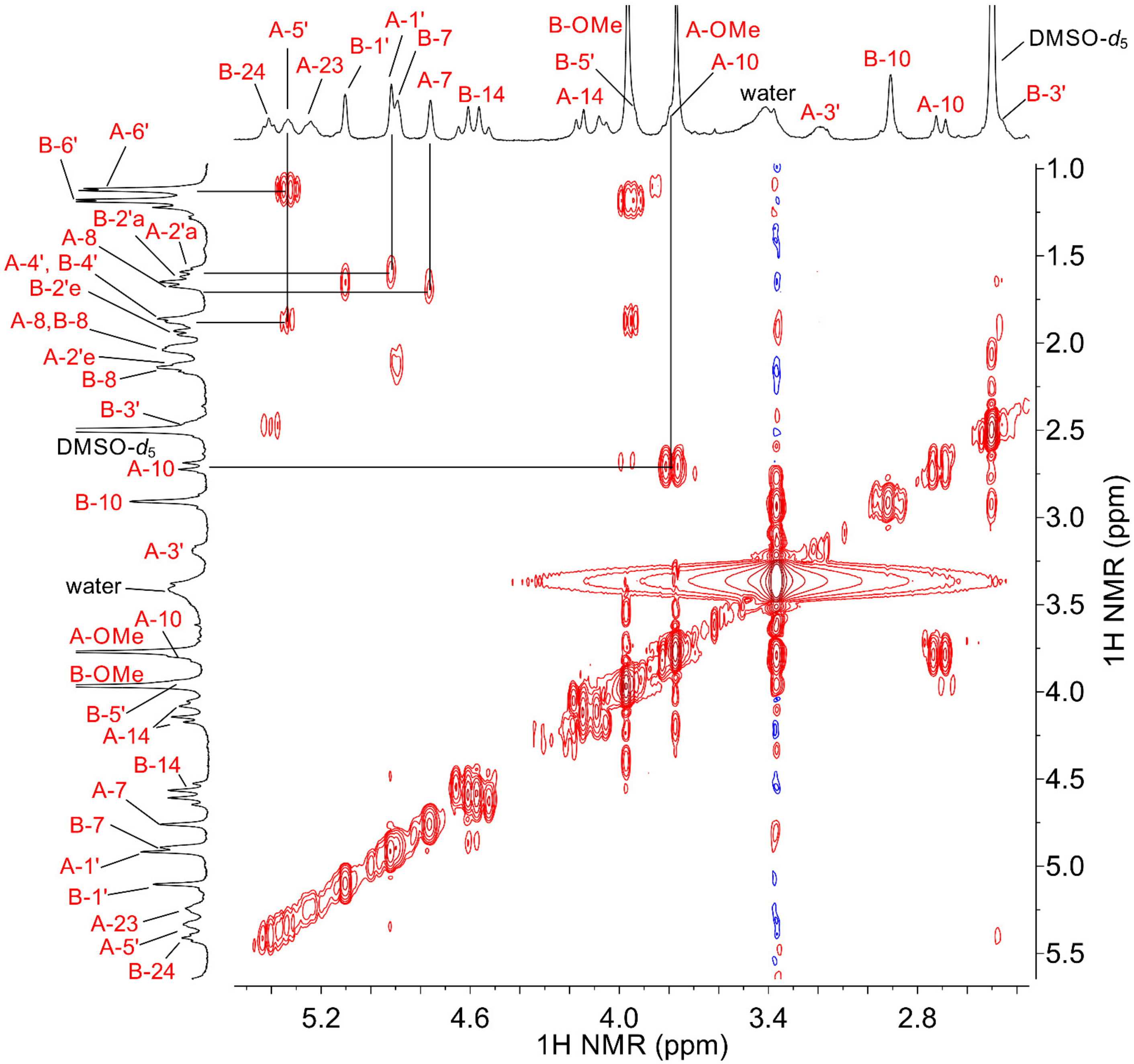

Figure S155. Expansion of ^1H-^1H COSY spectrum of DoxPt in DMSO-d_6 from 1.0 to 5.5 ppm (f1) and 2.4 to 5.6 ppm (f2). Crucial correlations related to Conformer A are indicated.

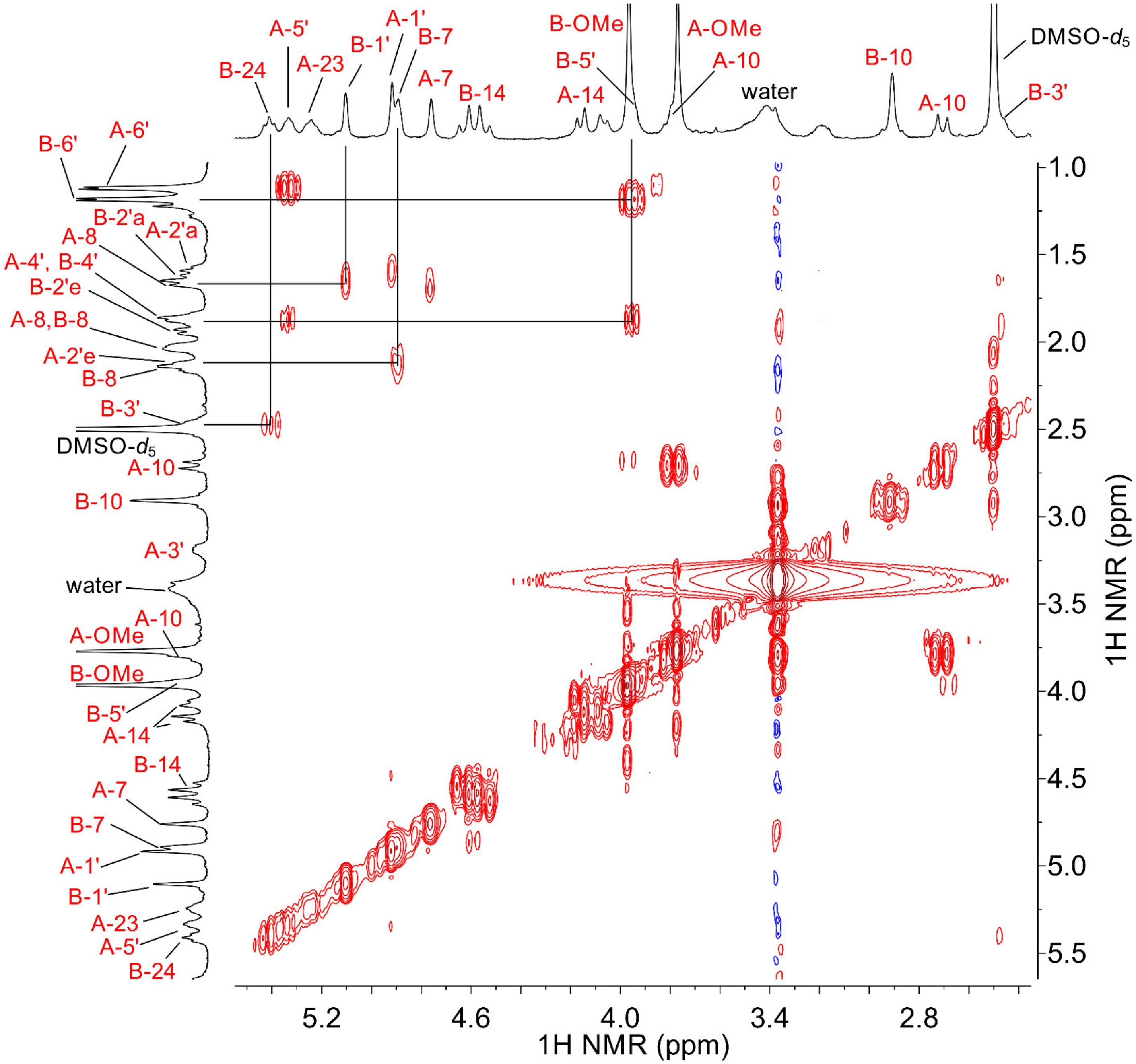

Figure S156. Expansion of ^{1}H-^{1}H COSY spectrum of DoxPt in DMSO-d_6 from 1.0 to 5.5 ppm (f1) and 2.4 to 5.6 ppm (f2). Crucial correlations related to Conformer B are indicated.

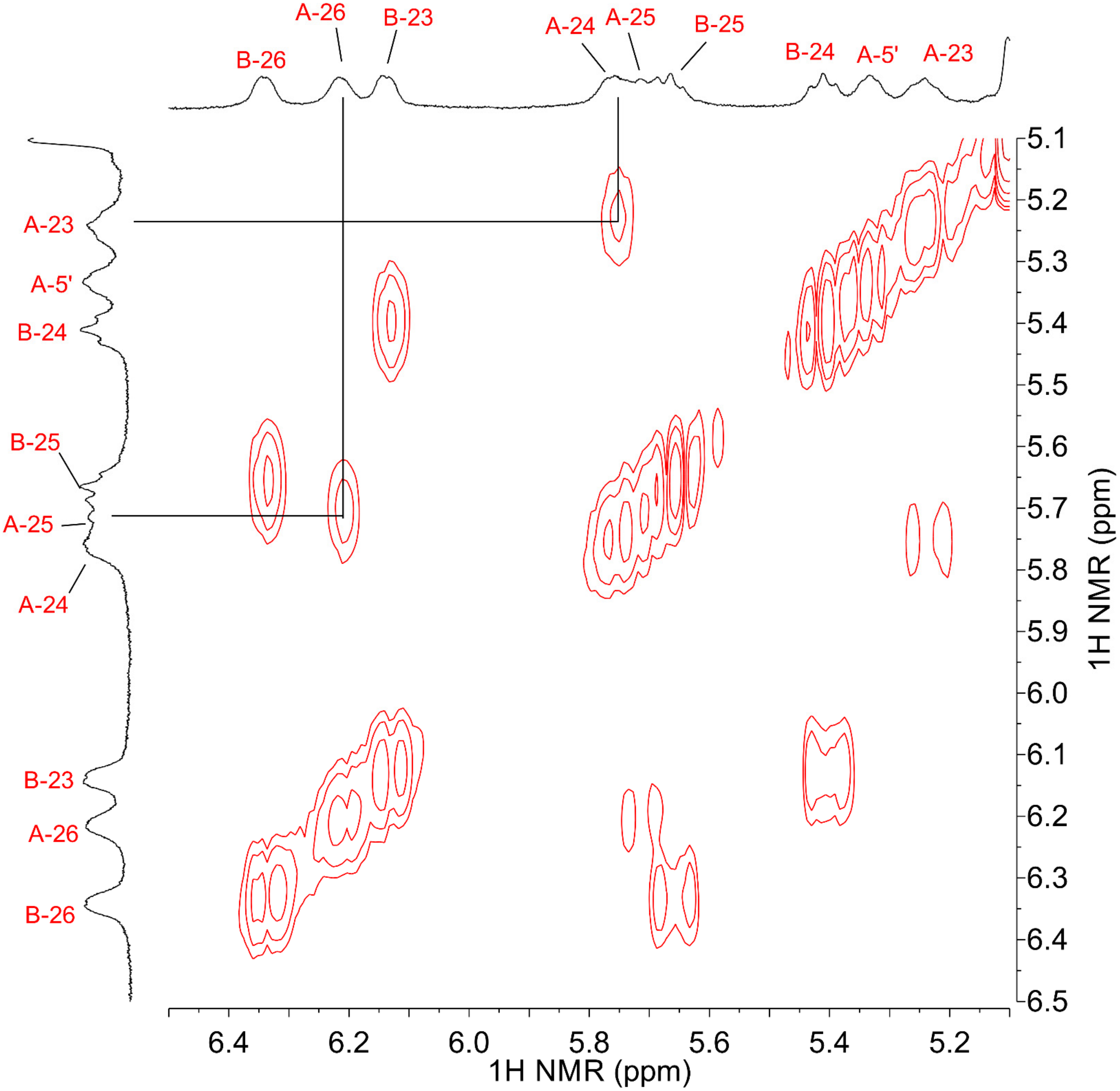

Figure S157. Expansion of ^{1}H-^{1}H COSY spectrum of DoxPt in DMSO-d_6 from 5.1 to 6.5 ppm (f1) and 5.1 to 6.5 ppm (f2). Crucial correlations related to Conformer A are indicated.

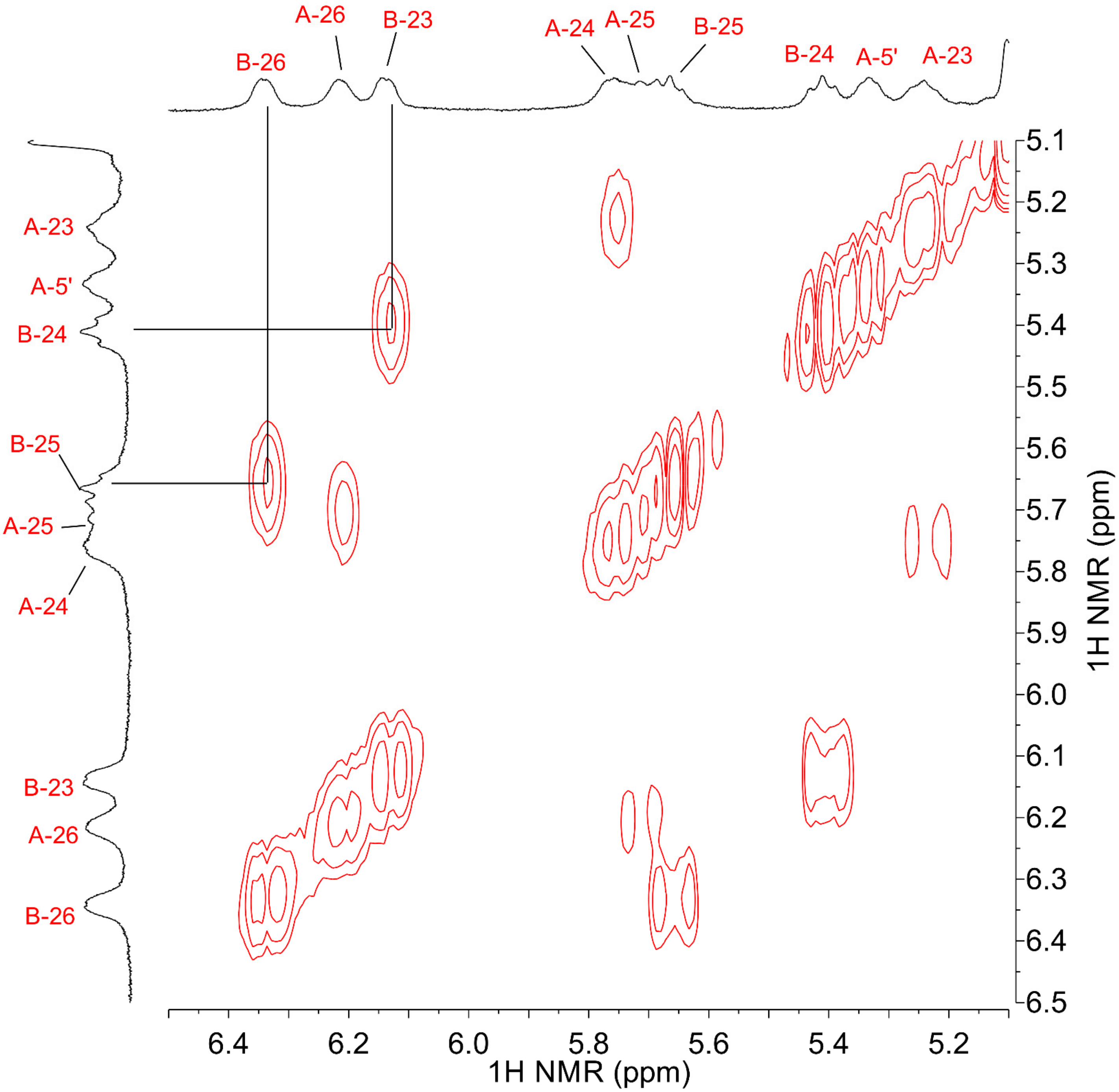

Figure S158. Expansion of ^{1}H-^{1}H COSY spectrum of DoxPt in DMSO-d_6 from 5.1 to 6.5 ppm (f1) and 5.1 to 6.5 ppm (f2). Crucial correlations related to Conformer B are indicated.

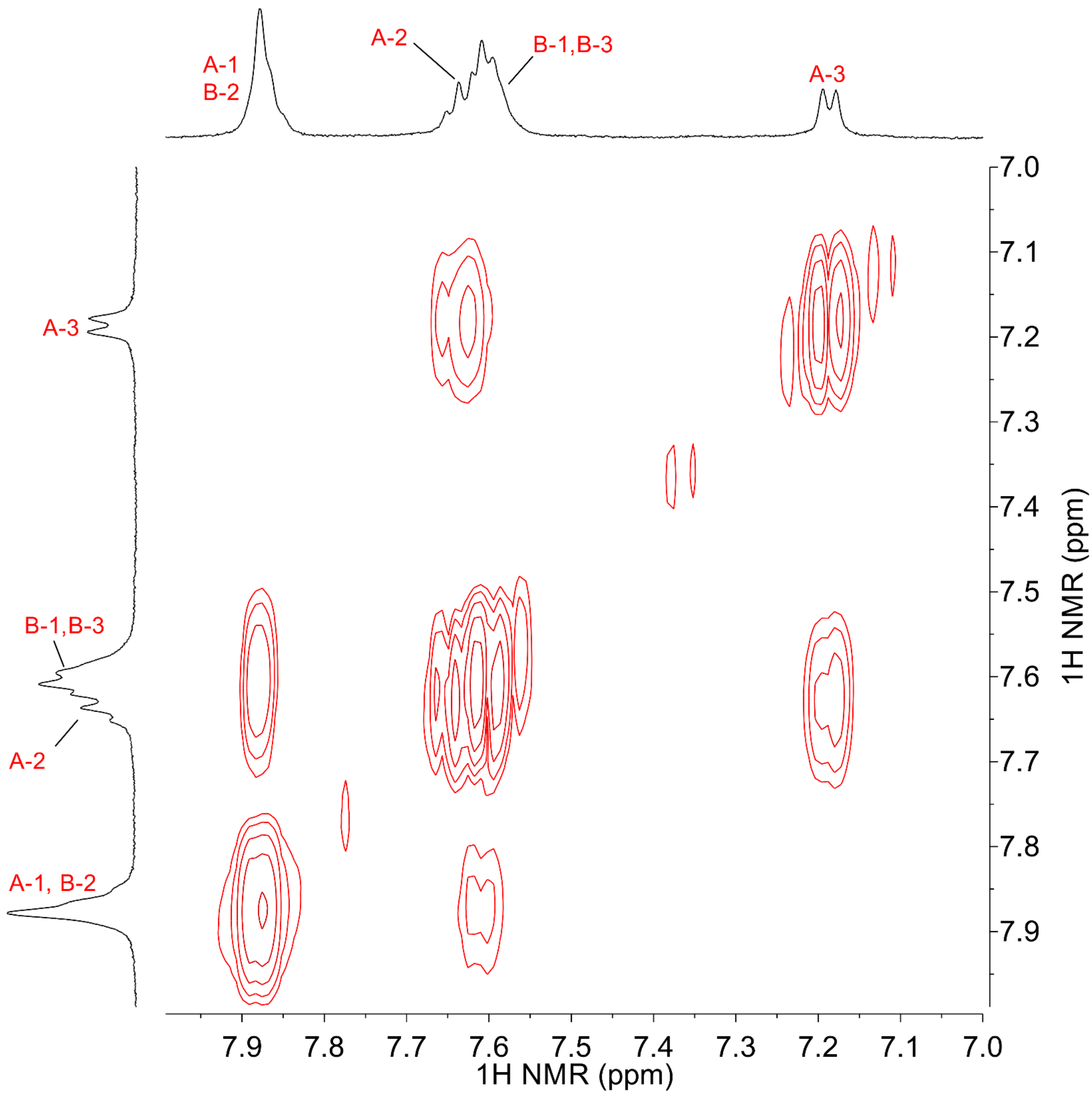

Figure S159. Expansion of ^{1}H-^{1}H COSY spectrum of DoxPt in DMSO-d_6 from 7.0 to 8.0 ppm.

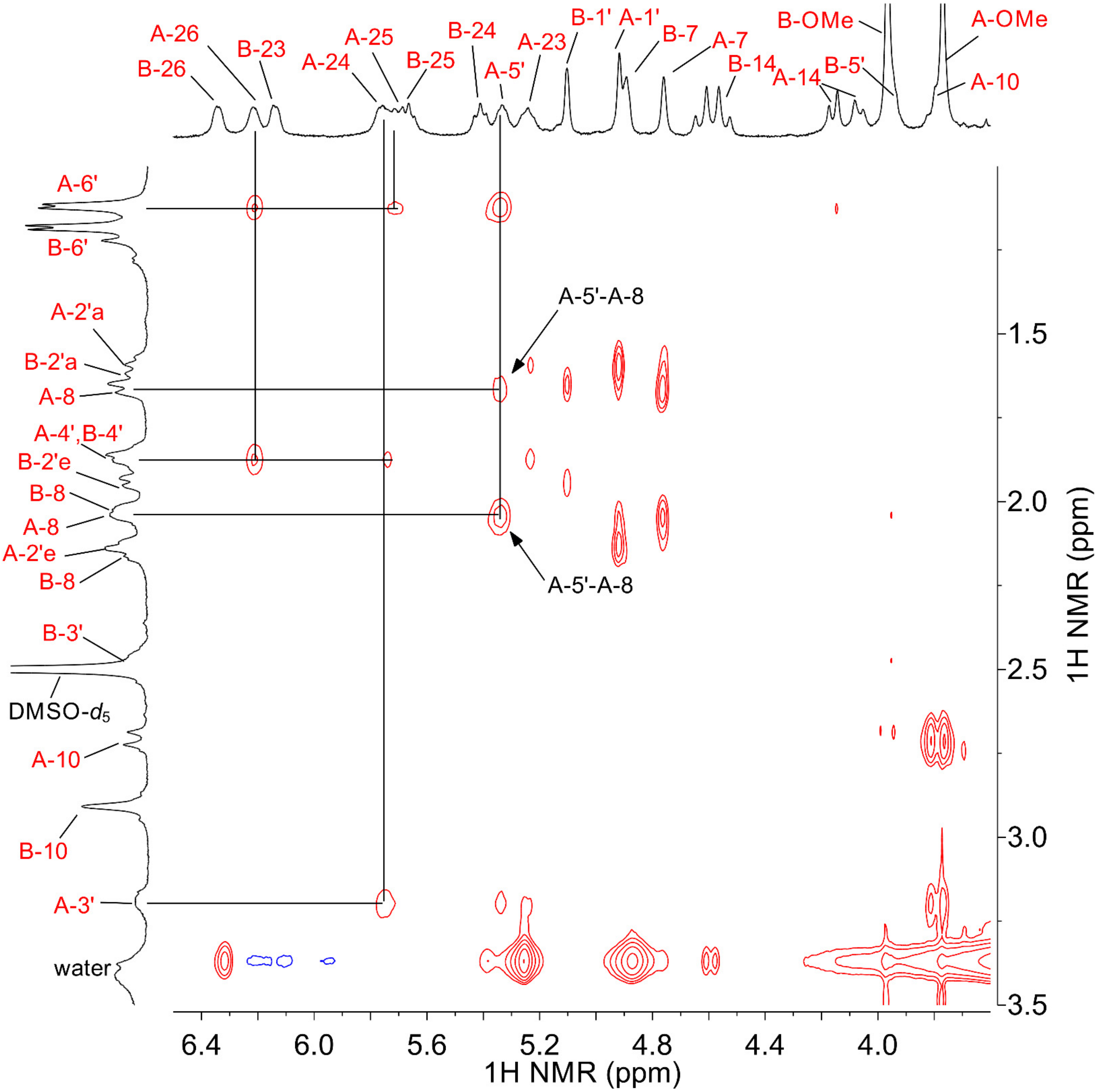

Figure S160. Expansion of ^{1}H-^{1}H NOESY spectrum of DoxPt in DMSO-d_6 from 1.0 to 3.5 ppm (f1) and 3.6 to 6.4 ppm (f2). Crucial correlations for identifying Conformer A are indicated.

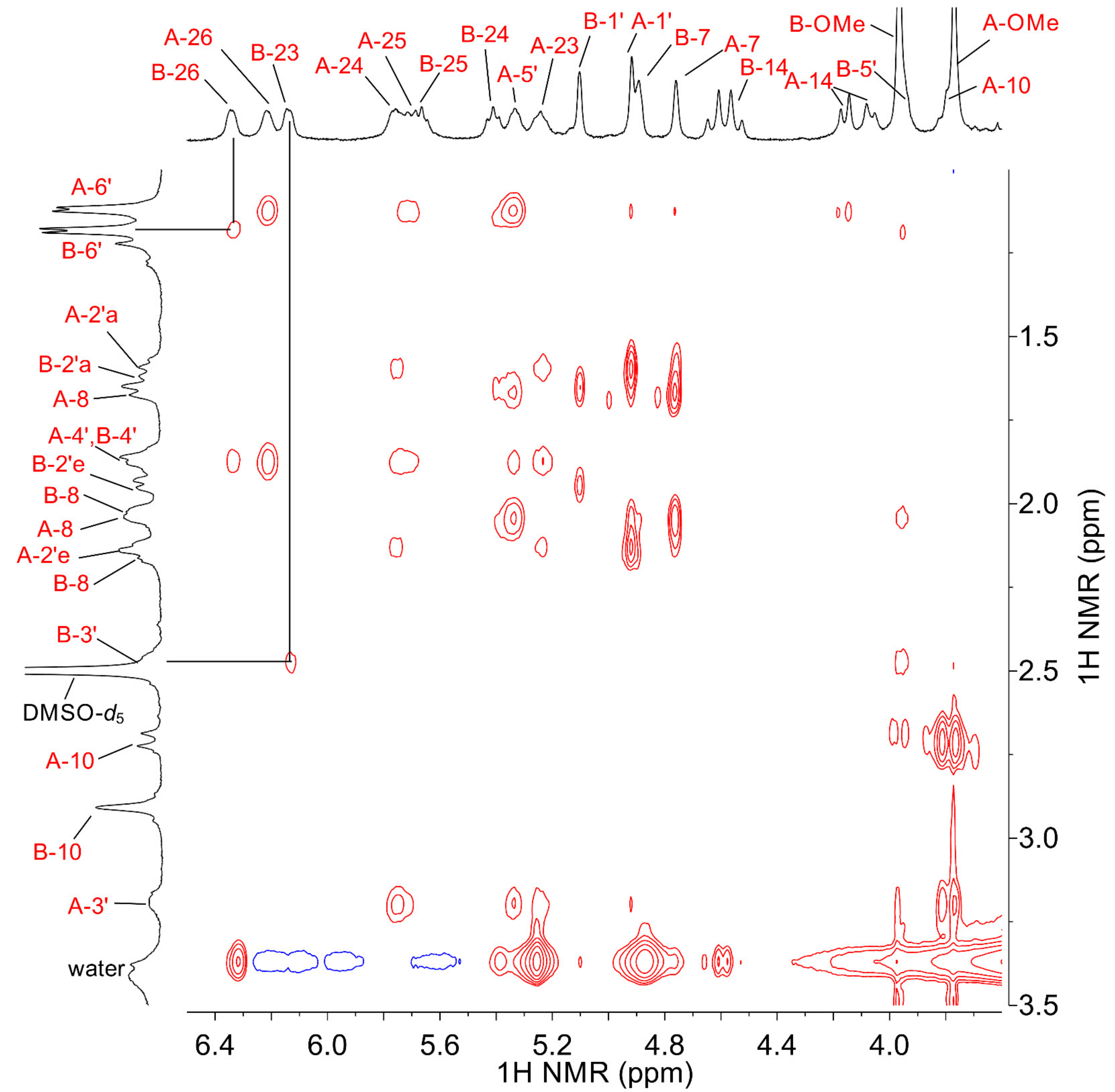

Figure S161. Expansion of ^1H-^1H NOESY spectrum of DoxPt in DMSO-d_6 from 1.0 to 3.5 ppm (f1) and 3.6 to 6.4 ppm (f2). Crucial correlations for identifying Conformer B are indicated.

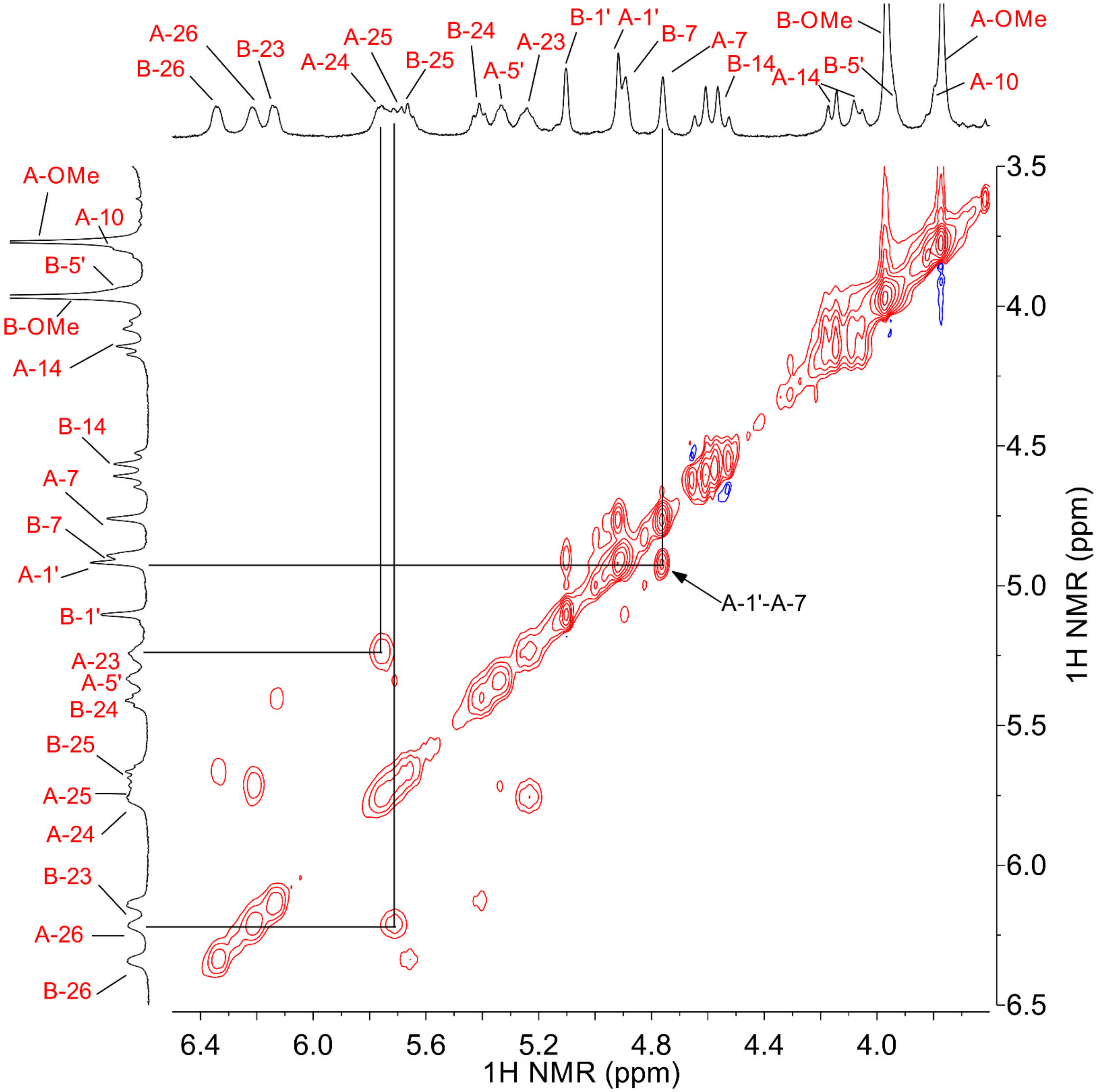

Figure S162. Expansion of ^{1}H-^{1}H NOESY spectrum of DoxPt in DMSO-d_6 from 3.5 to 6.5 ppm (f1) and 3.6 to 6.4 ppm (f2). Crucial correlations for identifying Conformer A are indicated.

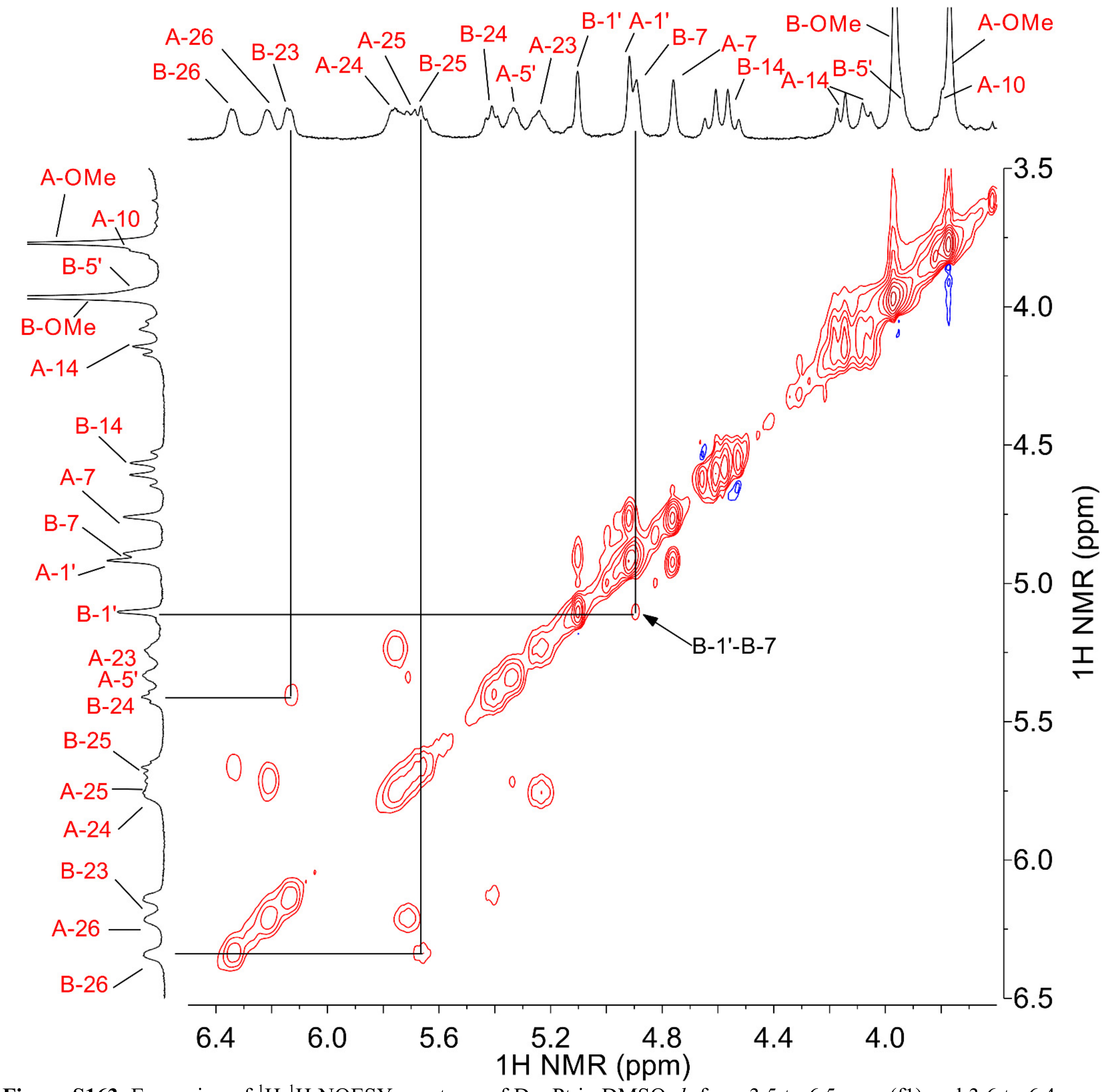

Figure S163. Expansion of ^{1}H-^{1}H NOESY spectrum of DoxPt in DMSO-d_6 from 3.5 to 6.5 ppm (f1) and 3.6 to 6.4 ppm (f2). Crucial correlations for identifying Conformer B are indicated.

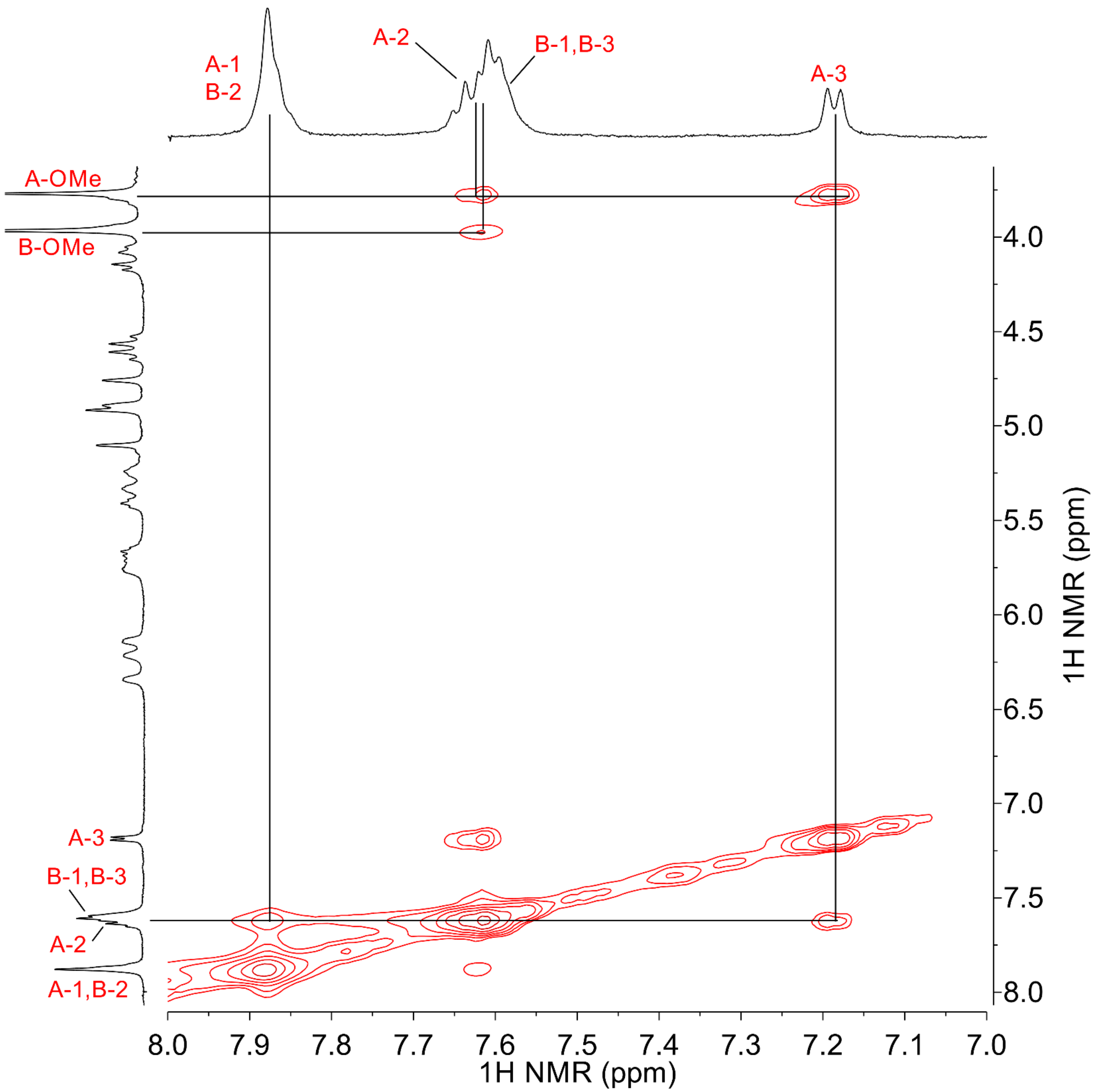

Figure S164. Expansion of ^{1}H-^{1}H NOESY spectrum of DoxPt in DMSO-d_6 from 3.75 to 8.00 ppm (f1) and 7.00 to 8.00 ppm (f2). Crucial correlations for identifying conformers are indicated.

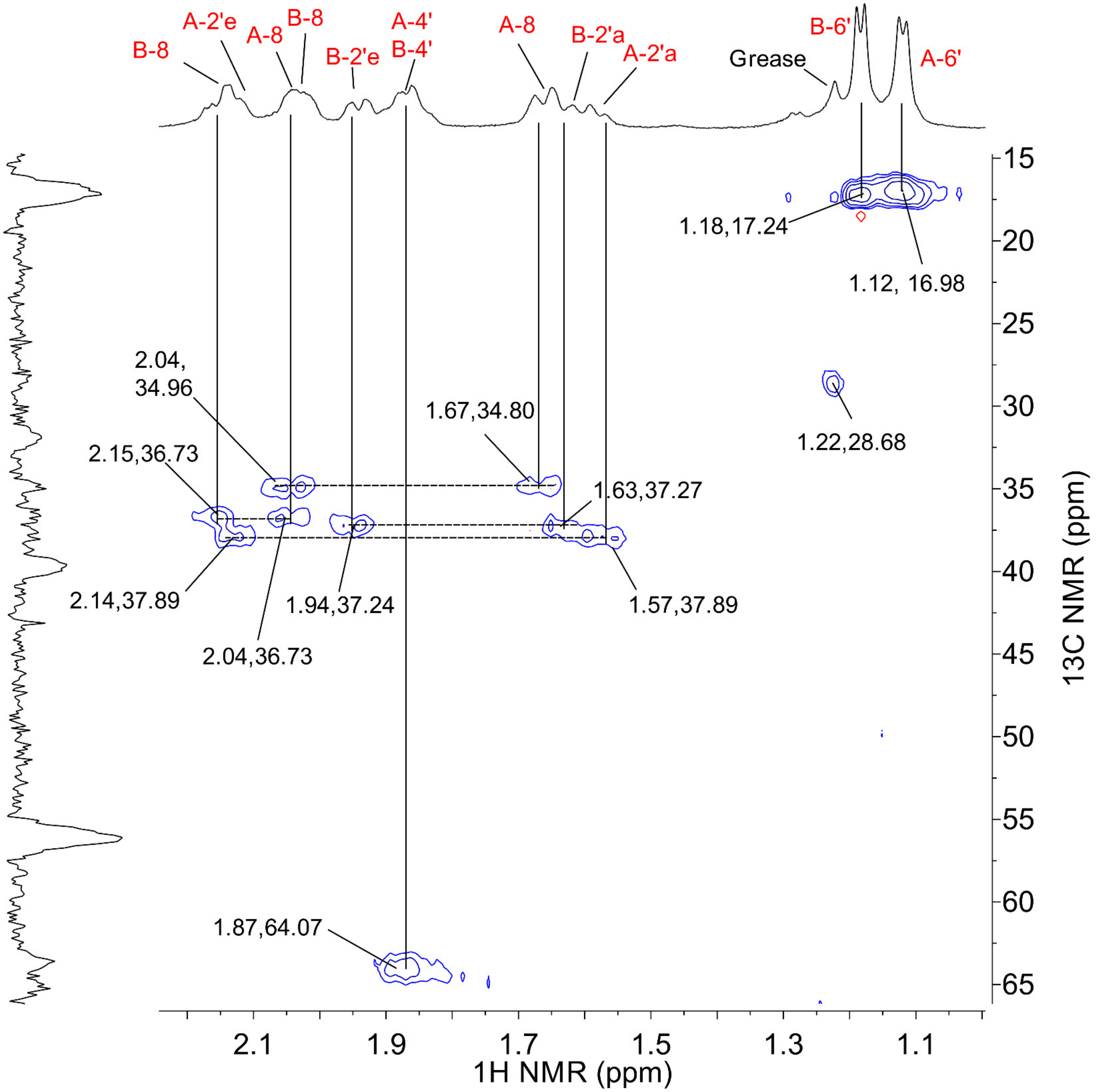

Figure S165. Expansion of ^{1}H-^{13}C HSQC spectrum of DoxPt in DMSO-d_6 from 1.0 to 2.2 ppm (^{1}H) and 15 to 65 ppm (^{13}C).

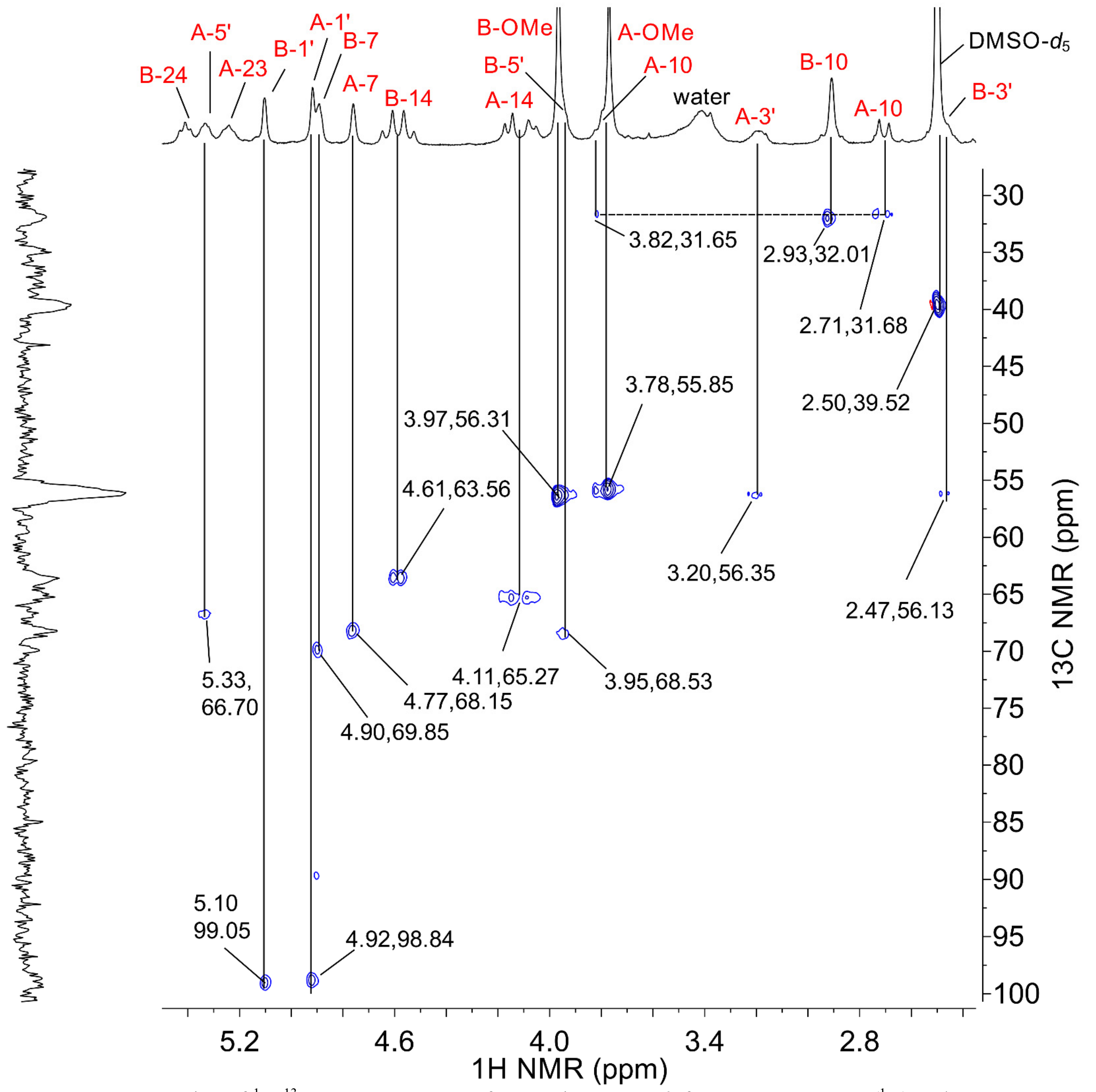

Figure S166. Expansion of ^{1}H-^{13}C HSQC spectrum of DoxPt in DMSO-d_6 from 2.4 to 5.4 ppm (^{1}H) and 30 to 100 ppm (^{13}C).

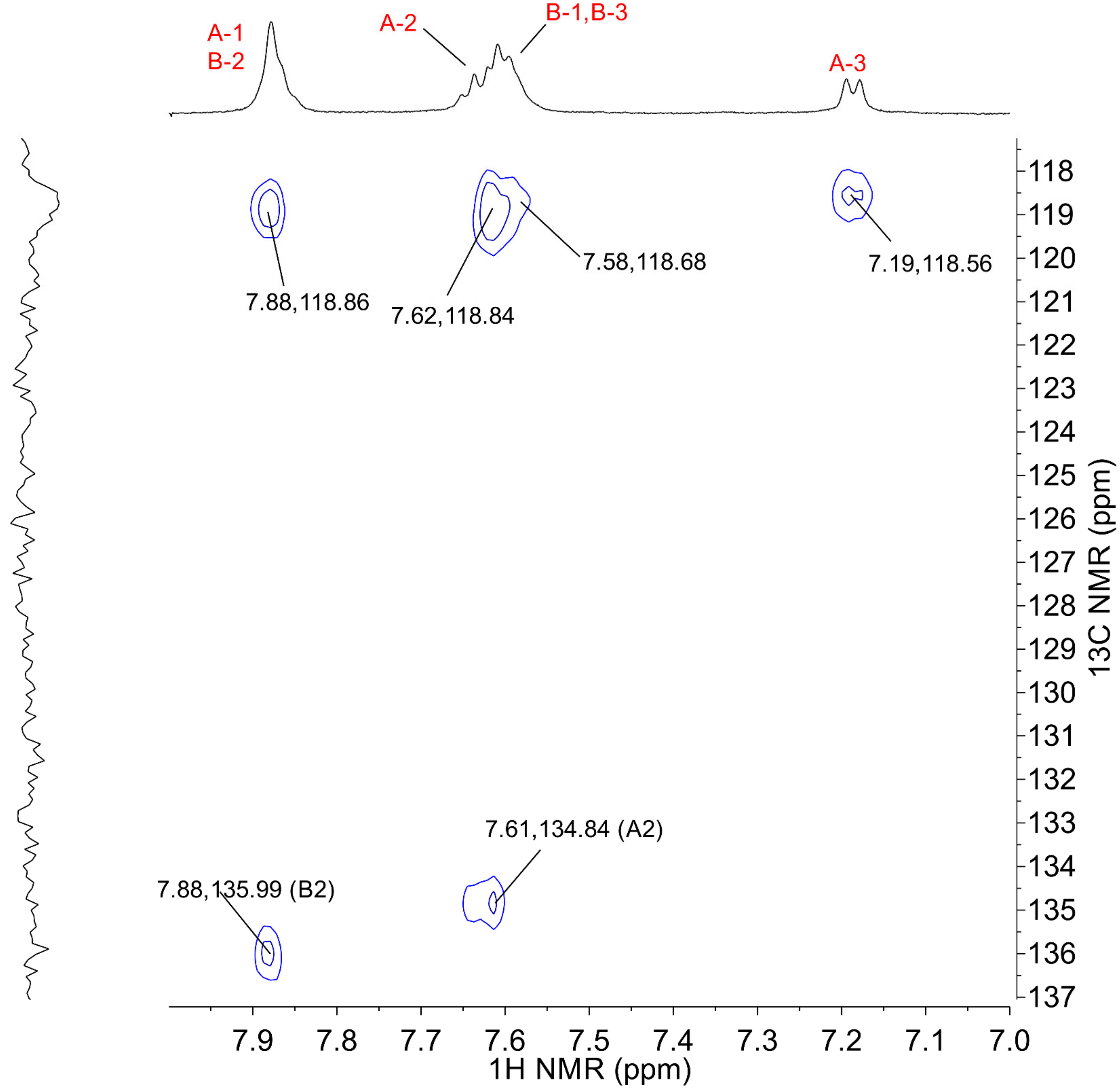

Figure S167. Expansion of ^{1}H-^{13}C HSQC spectrum of DoxPt in DMSO-d_6 from 7.0 to 8.0 ppm (^{1}H) and 117 to 137 ppm (^{13}C).

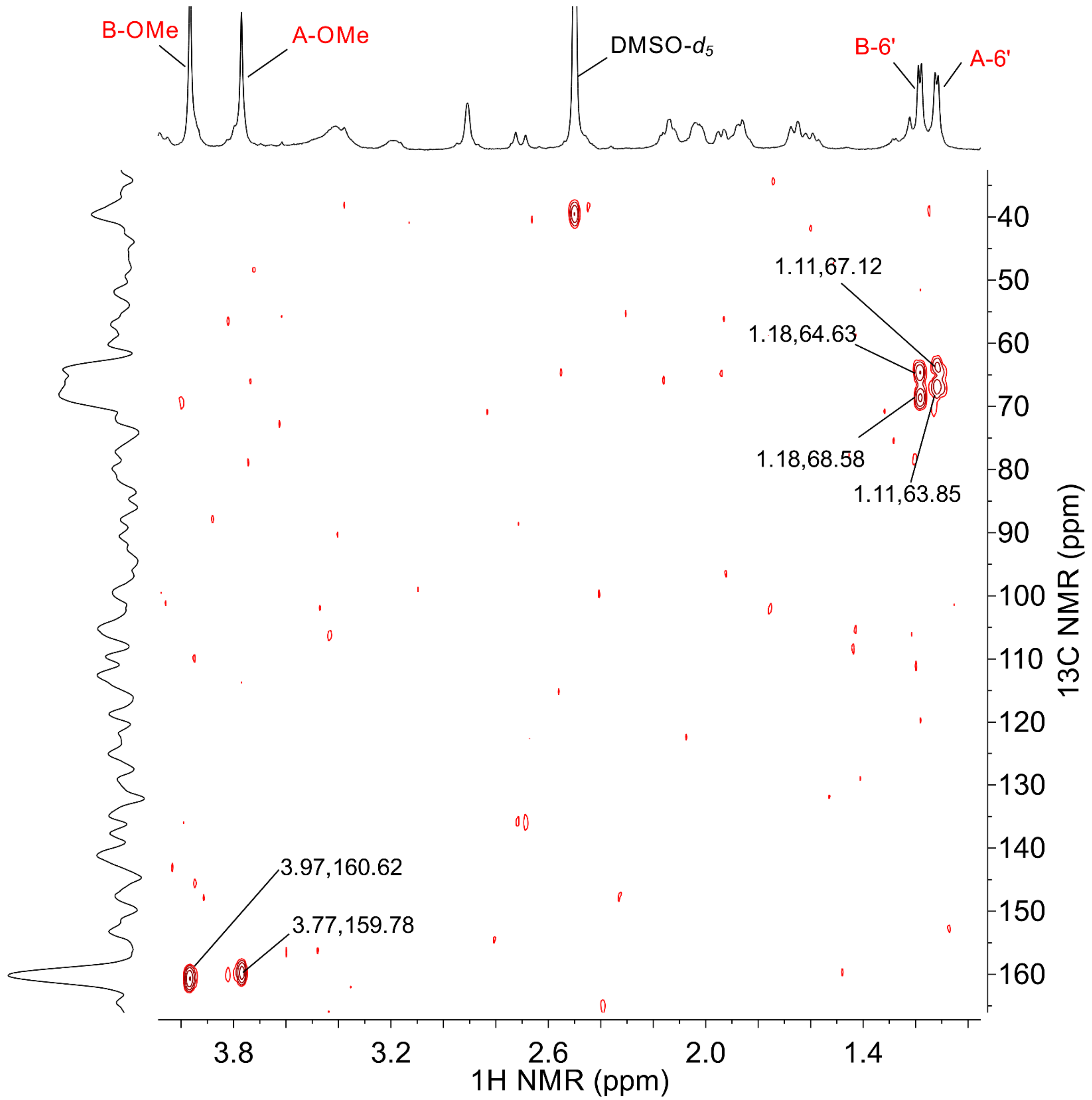

Figure S168. Expansion of ^1H-^{13}C HMBC spectrum of DoxPt in DMSO-d_6 from 1.0 to 4.0 ppm (^1H) and 35 to 165 ppm (^{13}C).

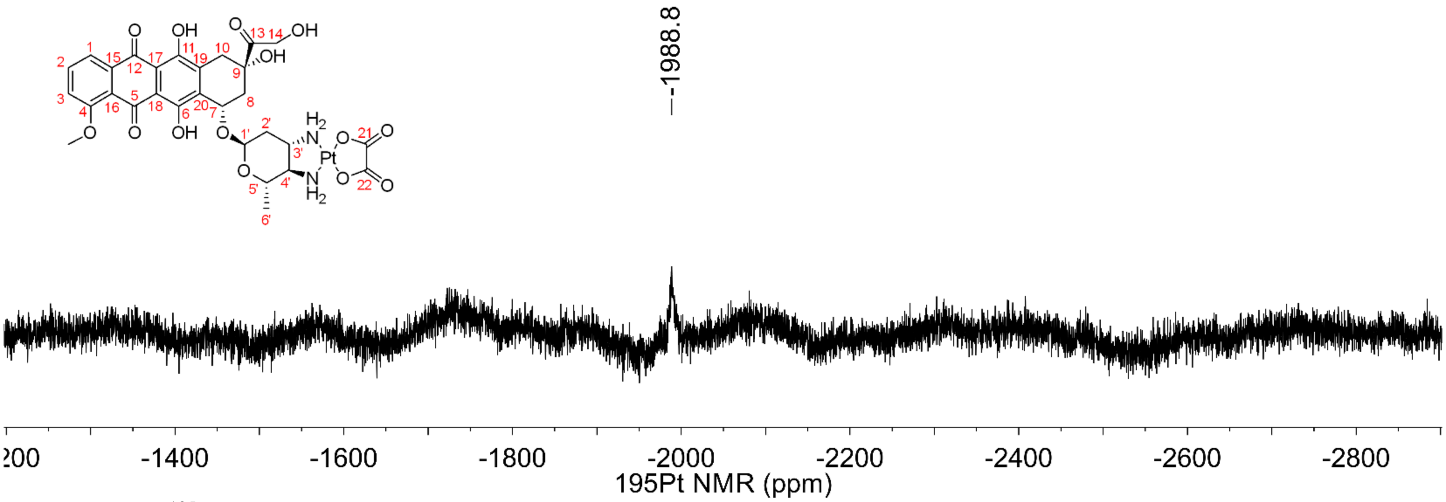

Figure S169. ^{195}Pt NMR spectrum of DoxPt in DMSO-d_6.

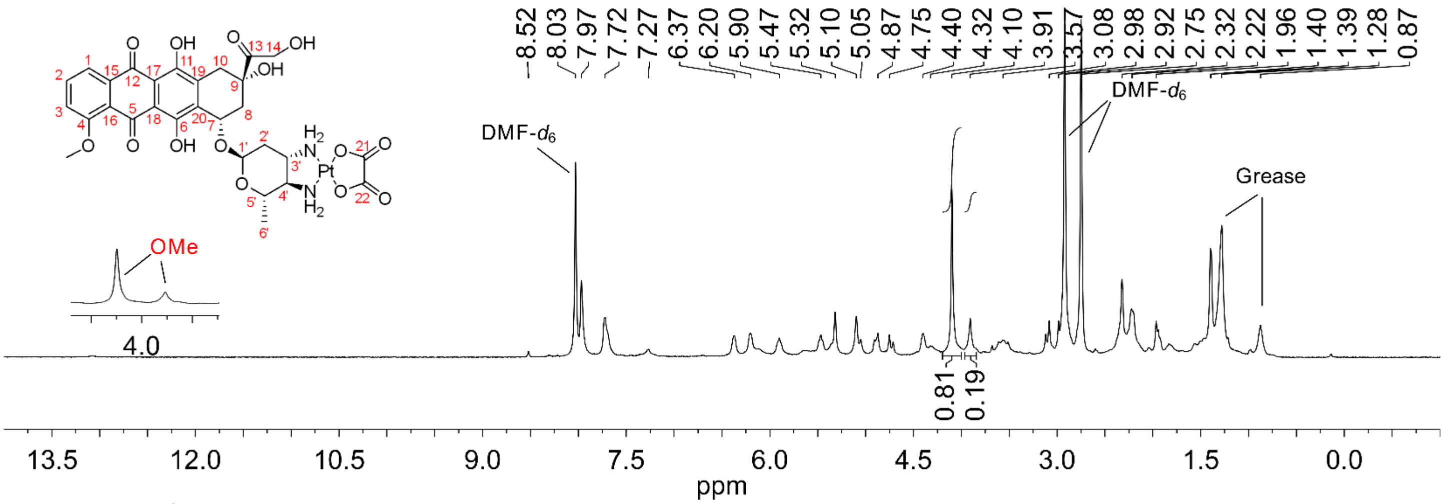

Figure S170. ^{1}H NMR spectrum of DoxPt in DMF-d_7. This spectrum is challenging to interpret due to the significant line broadening. The two signals at around 4 ppm are assigned to the methoxy group of two conformers of DoxPt, which are in an 81:19 ratio.

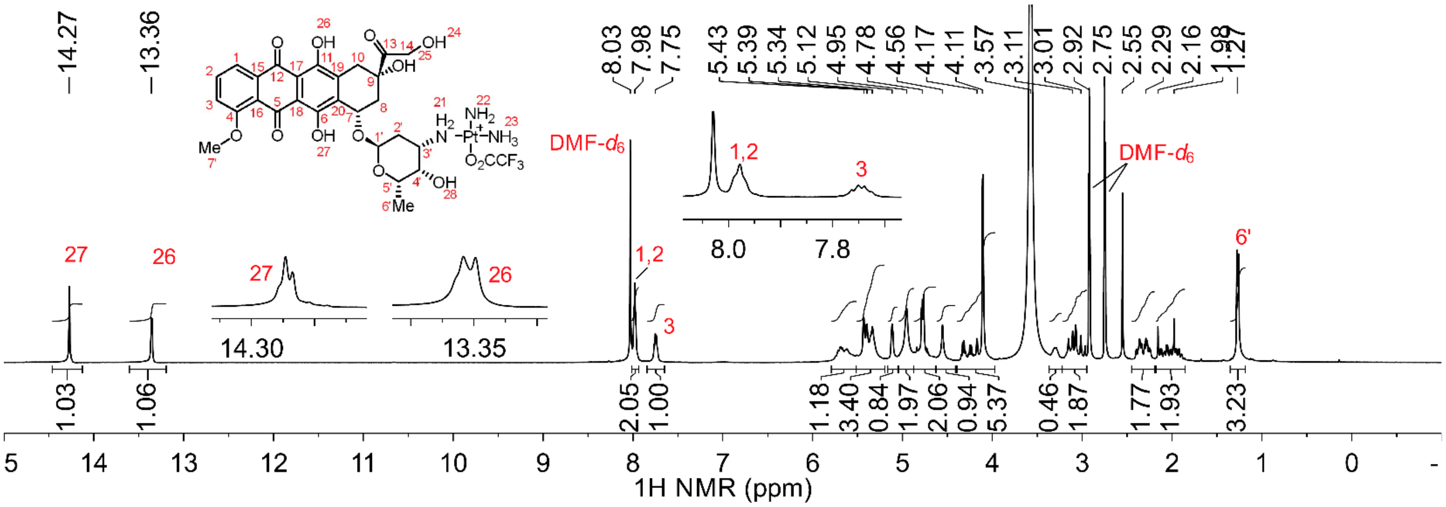

Figure S171. ^{1}H NMR spectrum of DoxPt4 in DMF-d_7. The free trifluoroacetate anion is omitted for clarity.

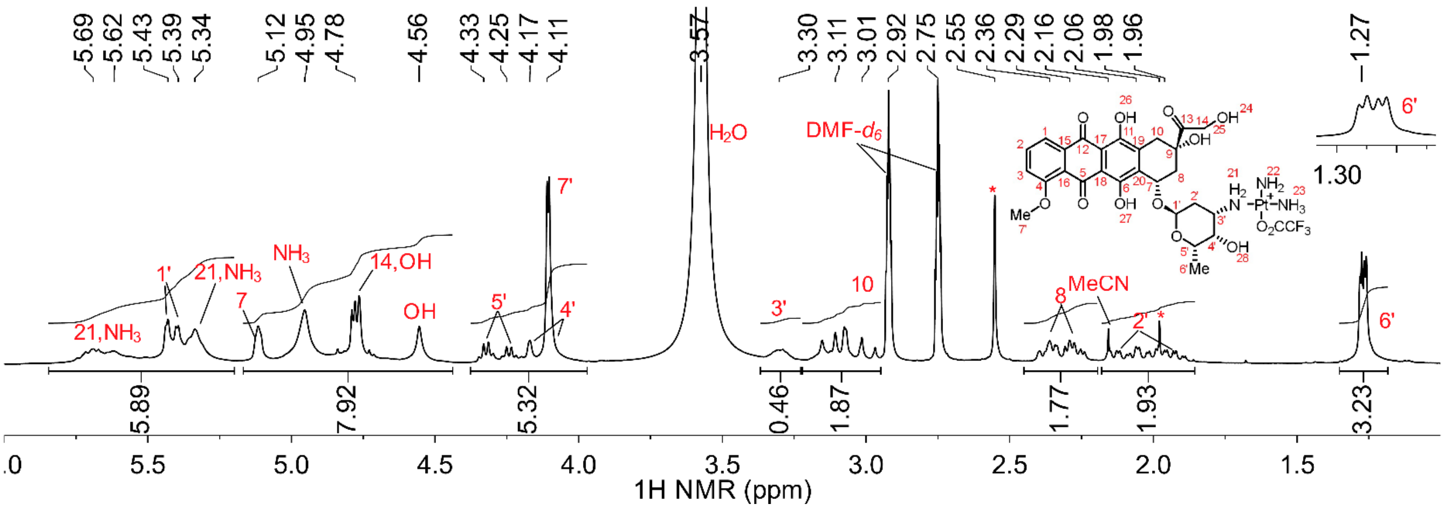

Figure S172. Expansion of ^{1}H NMR spectrum of DoxPt4 in DMF-d_7 from 1.0 to 6.0 ppm. The free trifluoroacetate anion is omitted for clarity.

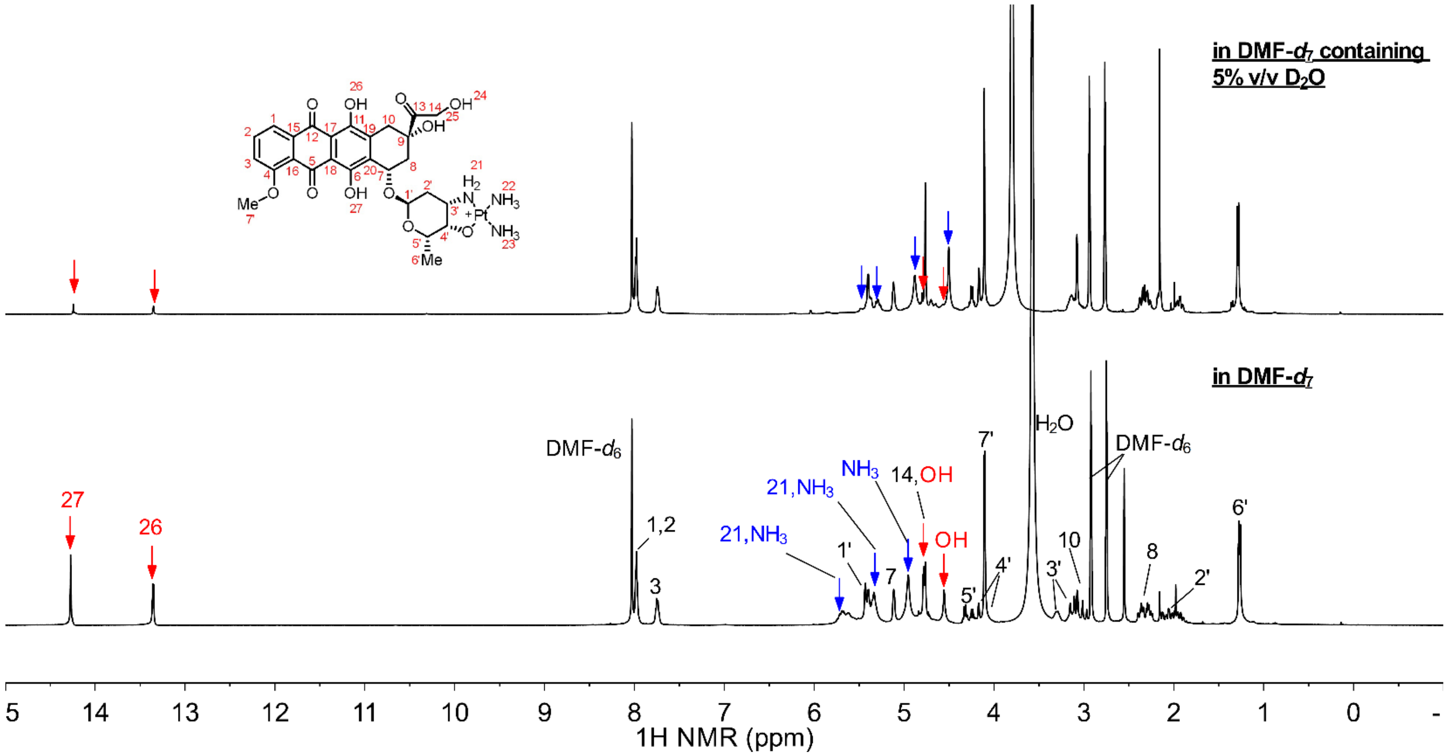

Figure S173. Comparison of the ^{1}H NMR spectrum of DoxPt4 in DMF-*d*$_7$ and in DMF-*d*$_7$/D_2O (95:5, v/v). Exchangable protons of the OH groups are indicated with red arrows. Protons of the N–H groups are indicated with blue arrows. The free trifluoroacetate anion is omitted for clarity.

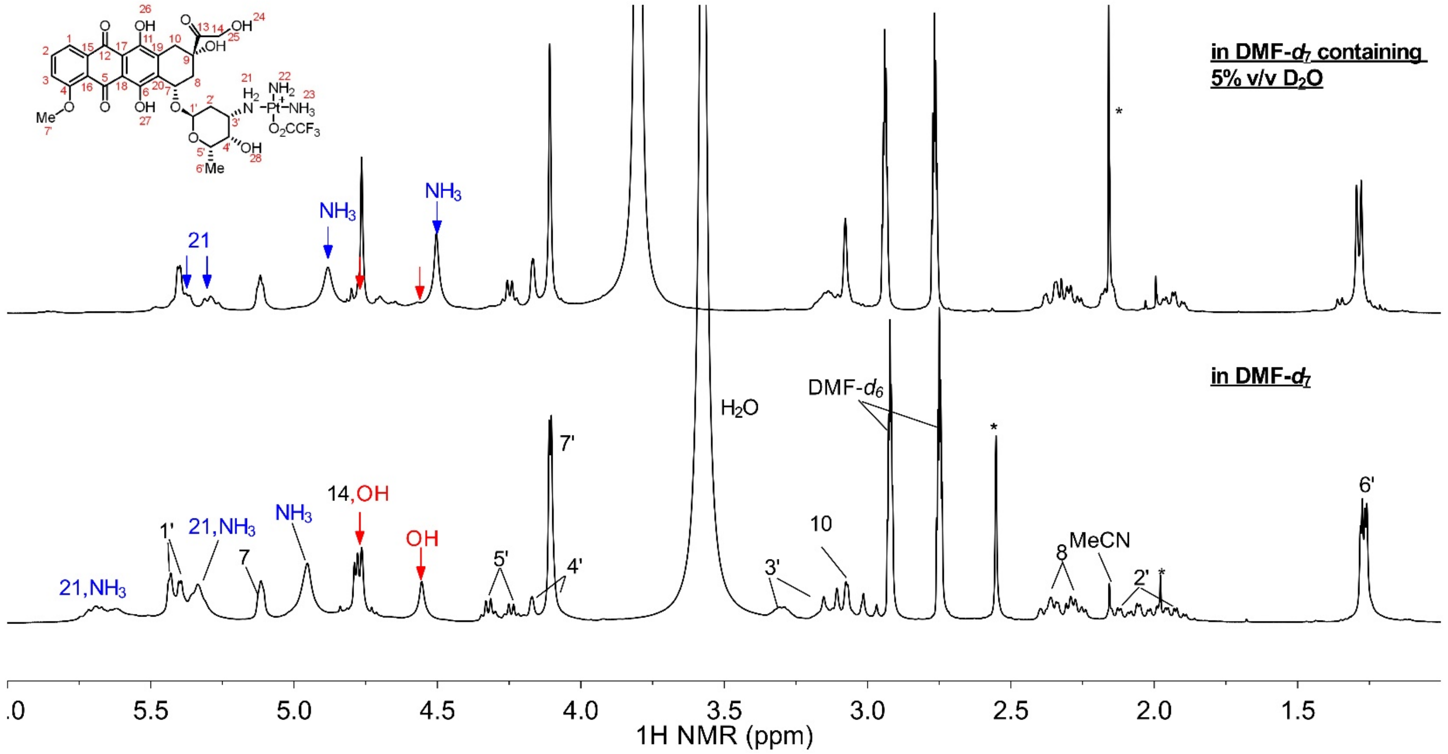

Figure S174. Comparison of the ^{1}H NMR spectrum of DoxPt4 in DMF-*d*$_7$ and in DMF-*d*$_7$/D_2O (95:5, v/v) in the range from 1.0 to 6.0 ppm. Exchangable protons of the OH groups are indicated with red arrows. Protons of the N–H groups are indicated with blue arrows. The free trifluoroacetate anion is omitted for clarity.

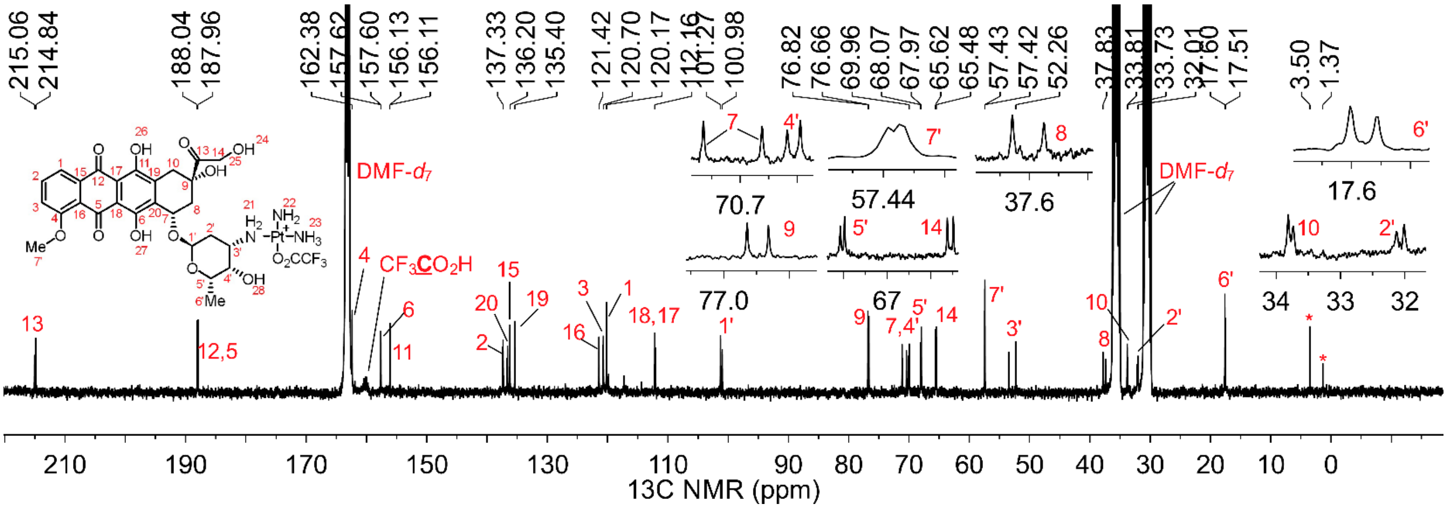

Figure S175. $^{13}C\{^1H\}$ NMR spectrum of DoxPt4 in DMF-d_7. The free trifluoroacetate anion is omitted for clarity.

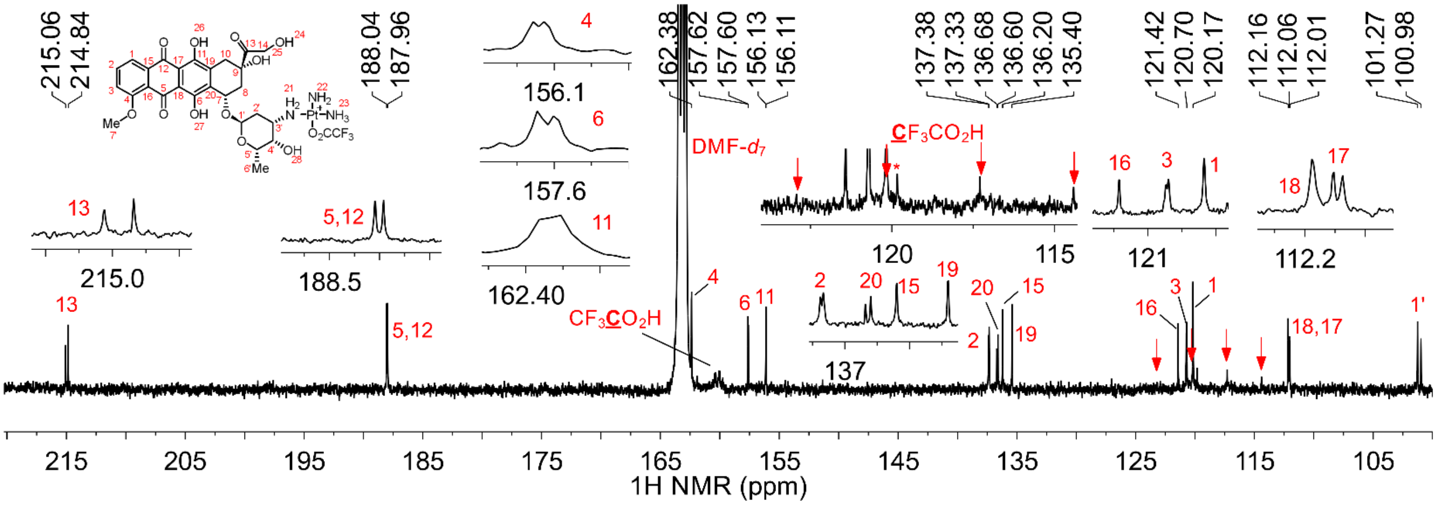

Figure S176. Expansion of $^{13}C\{^1H\}$ NMR spectrum of DoxPt4 in DMF-d_7 from 100 to 220 ppm. The free trifluoroacetate anion is omitted for clarity.

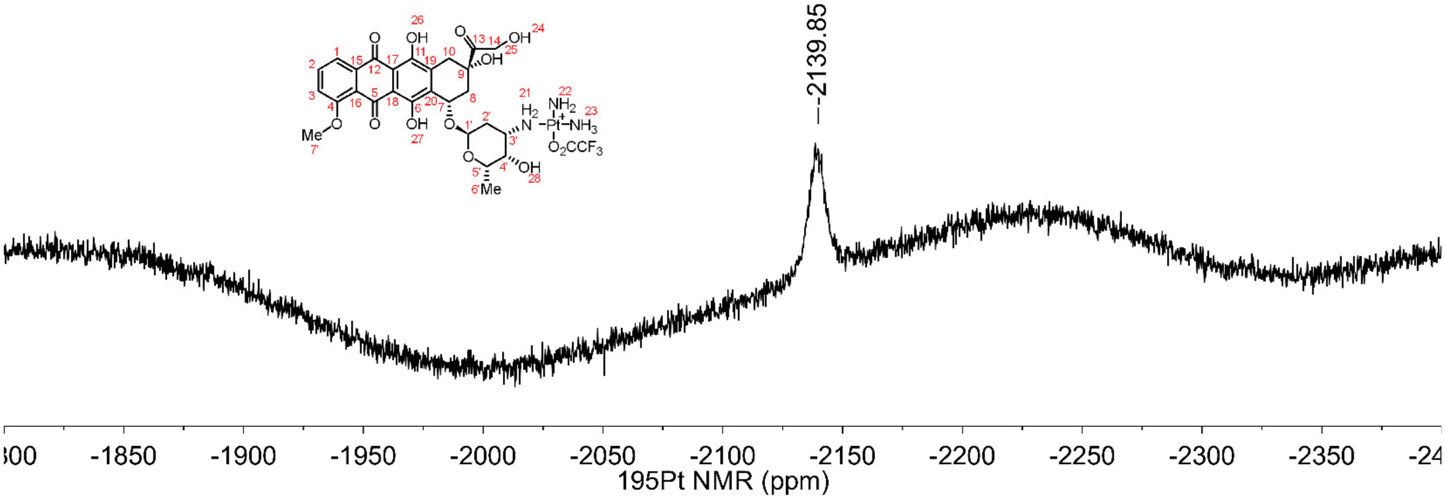

Figure S177. ^{195}Pt NMR spectrum of DoxPt4 in DMF-d_7. The free trifluoroacetate anion is omitted for clarity.

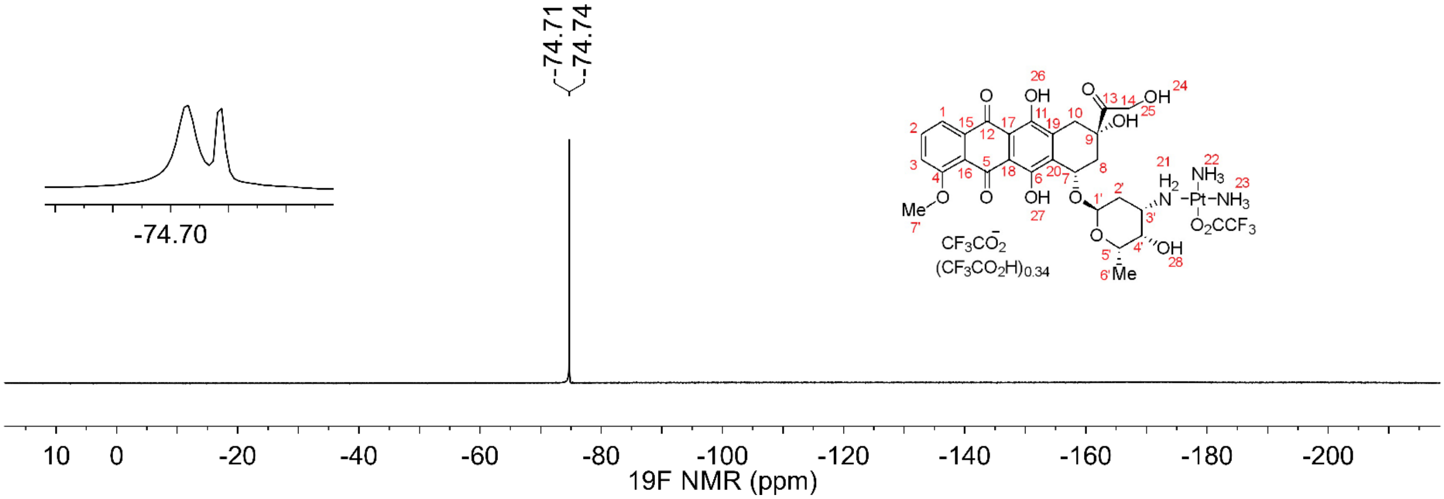

Figure S178. ^{19}F NMR spectrum of DoxPt4 in DMF-d_7.

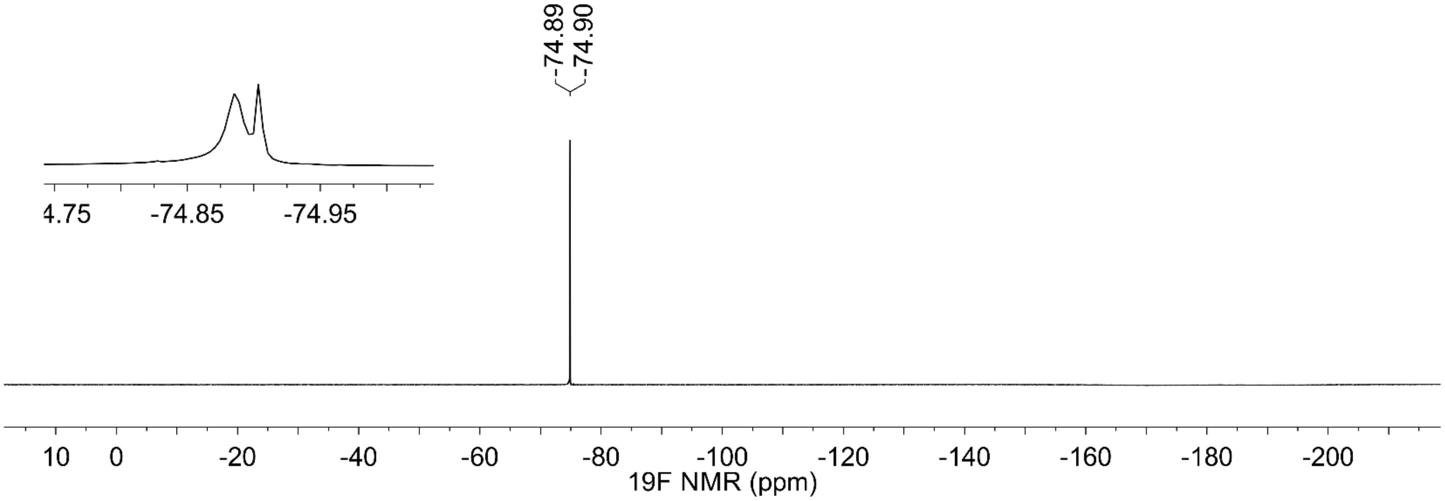

Figure S179. ^{19}F NMR spectrum of DoxPt4 in DMF-d_7/D_2O (95:5, v/v).

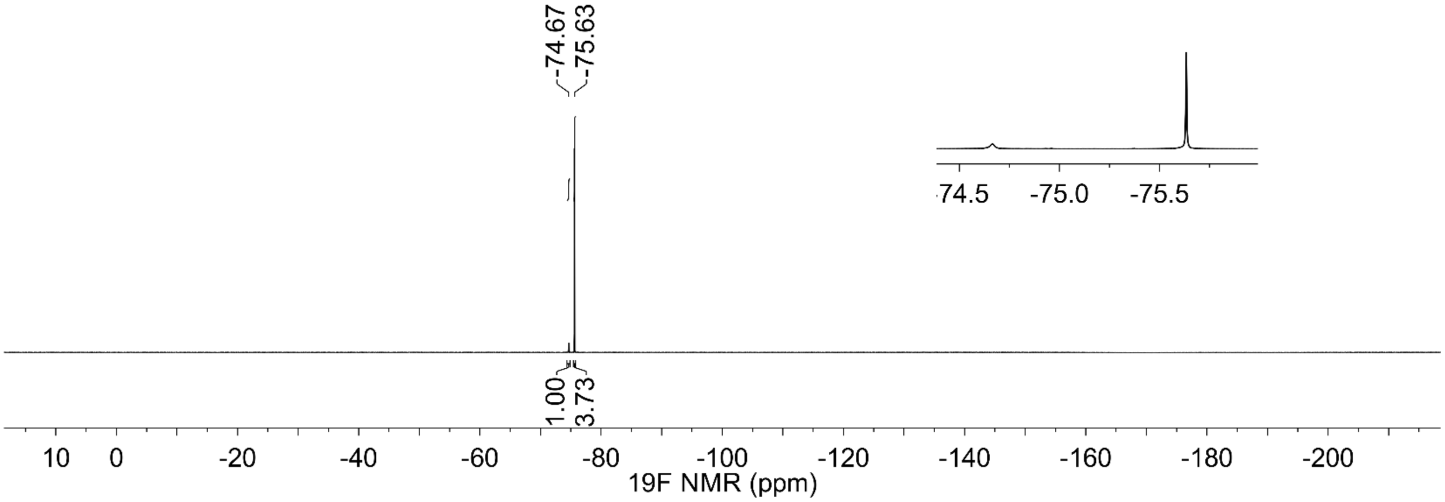

Figure S180. ^{19}F NMR spectrum of DoxPt4 in D_2O.

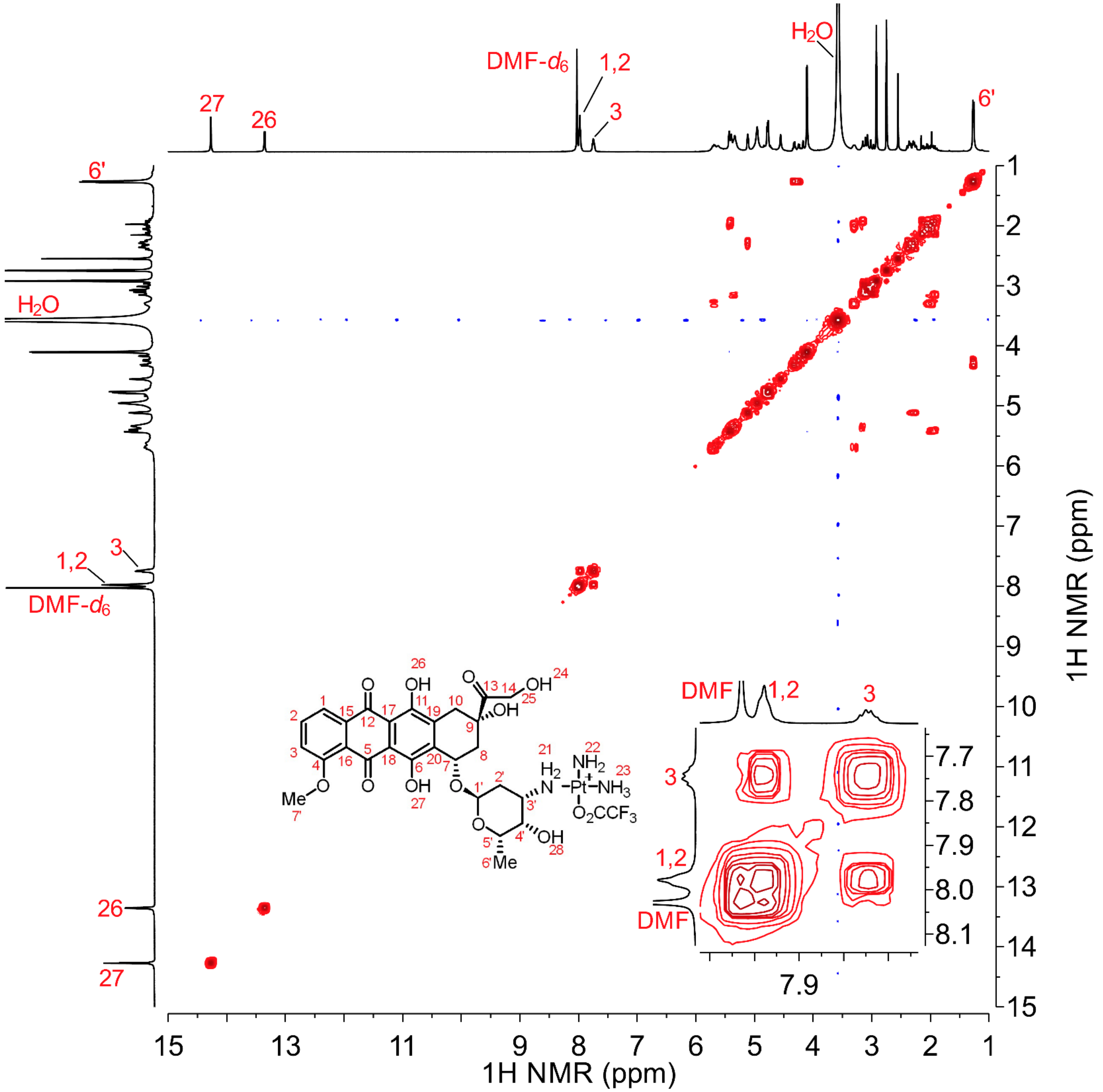

Figure S181. ^1H-^1H COSY spectrum of DoxPt4 in DMF-d_7. The free trifluoroacetate anion is omitted for clarity.

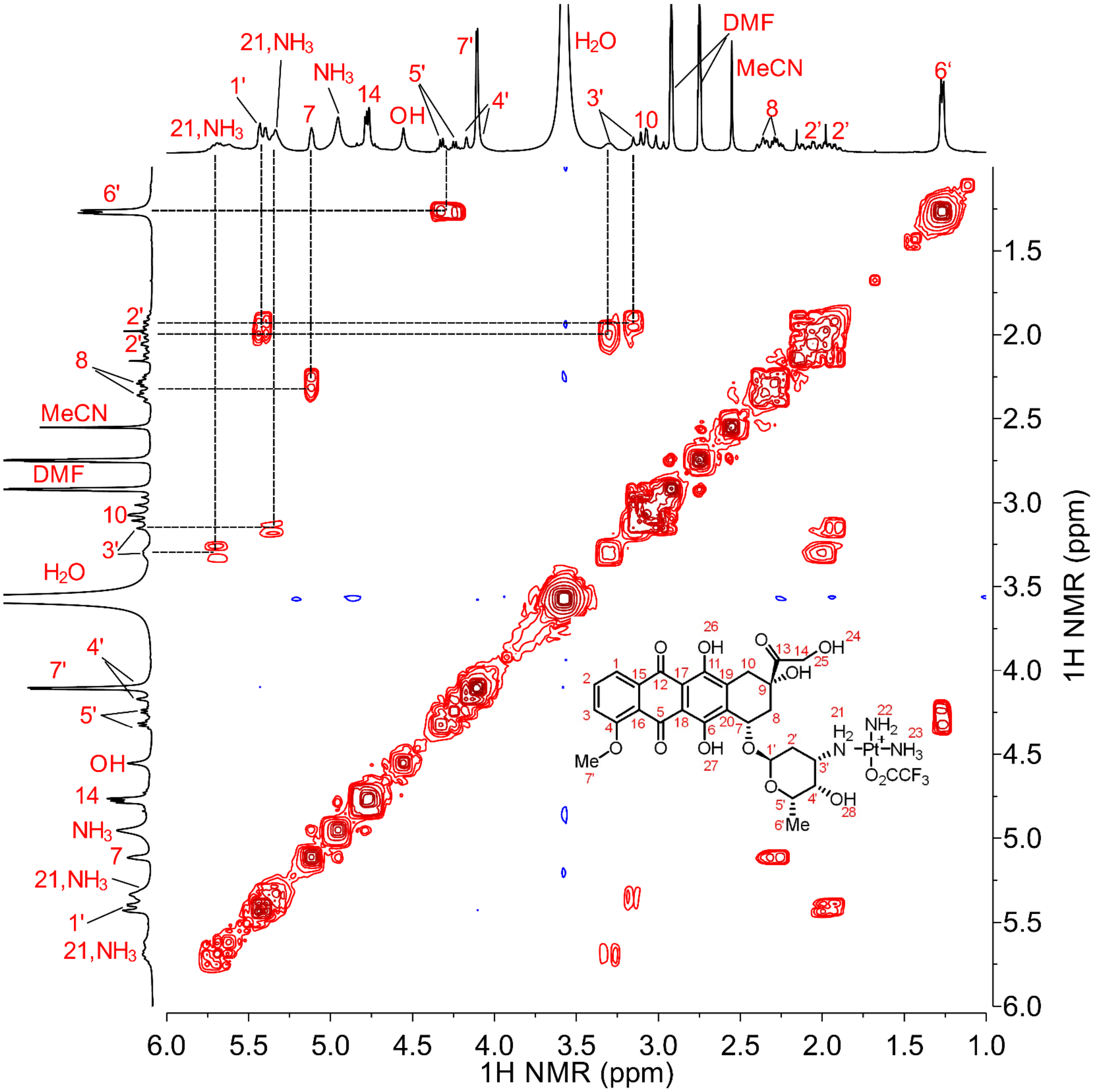

Figure S182. Expansion of ^{1}H-^{1}H COSY spectrum of DoxPt4 in DMF-d_7 from 1.0 to 6.0 ppm. The free trifluoroacetate anion is omitted for clarity.

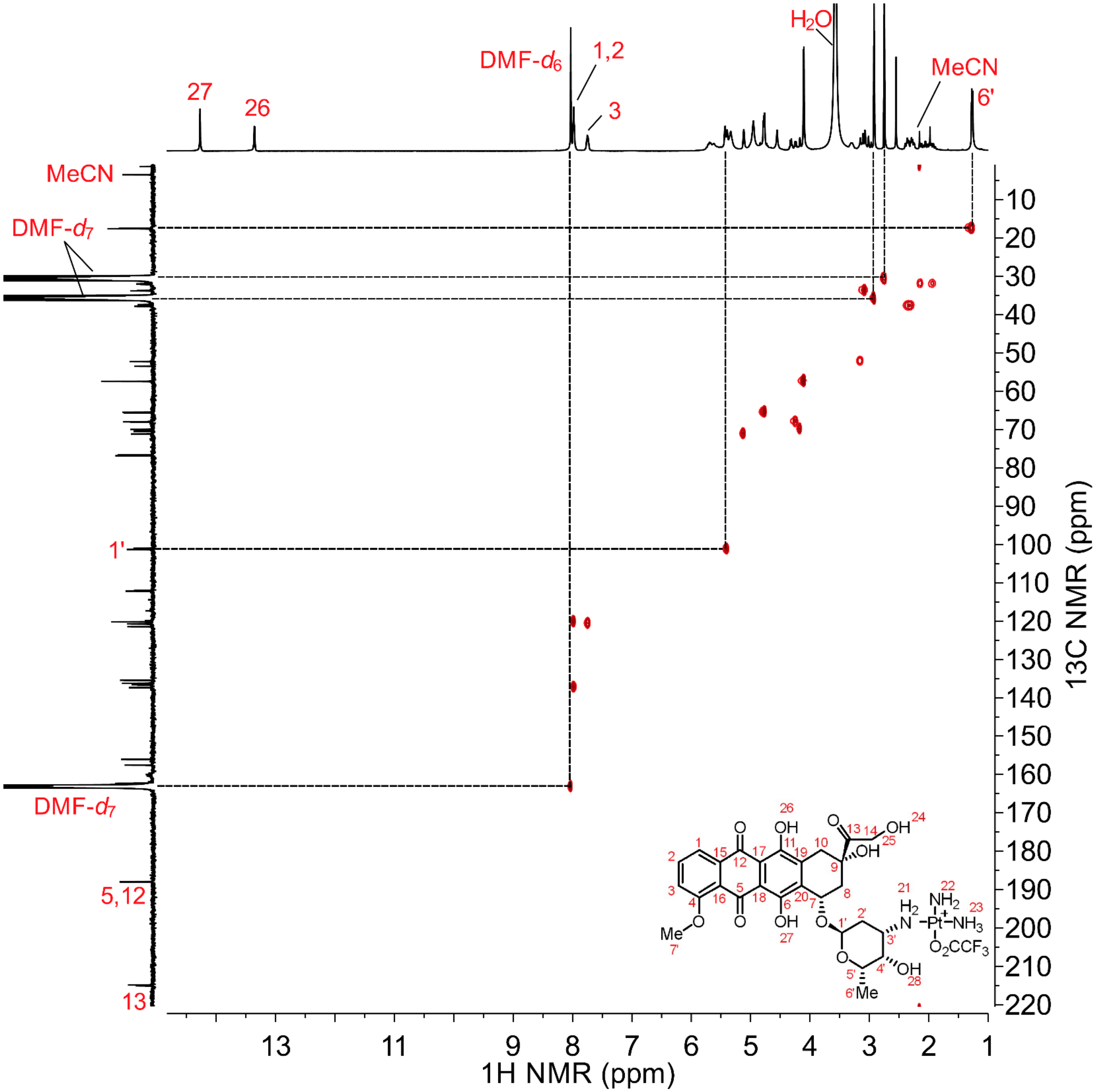

Figure S183. ^{1}H-^{13}C HSQC spectrum of DoxPt4 in DMF-d_7. The free trifluoroacetate anion is omitted for clarity.

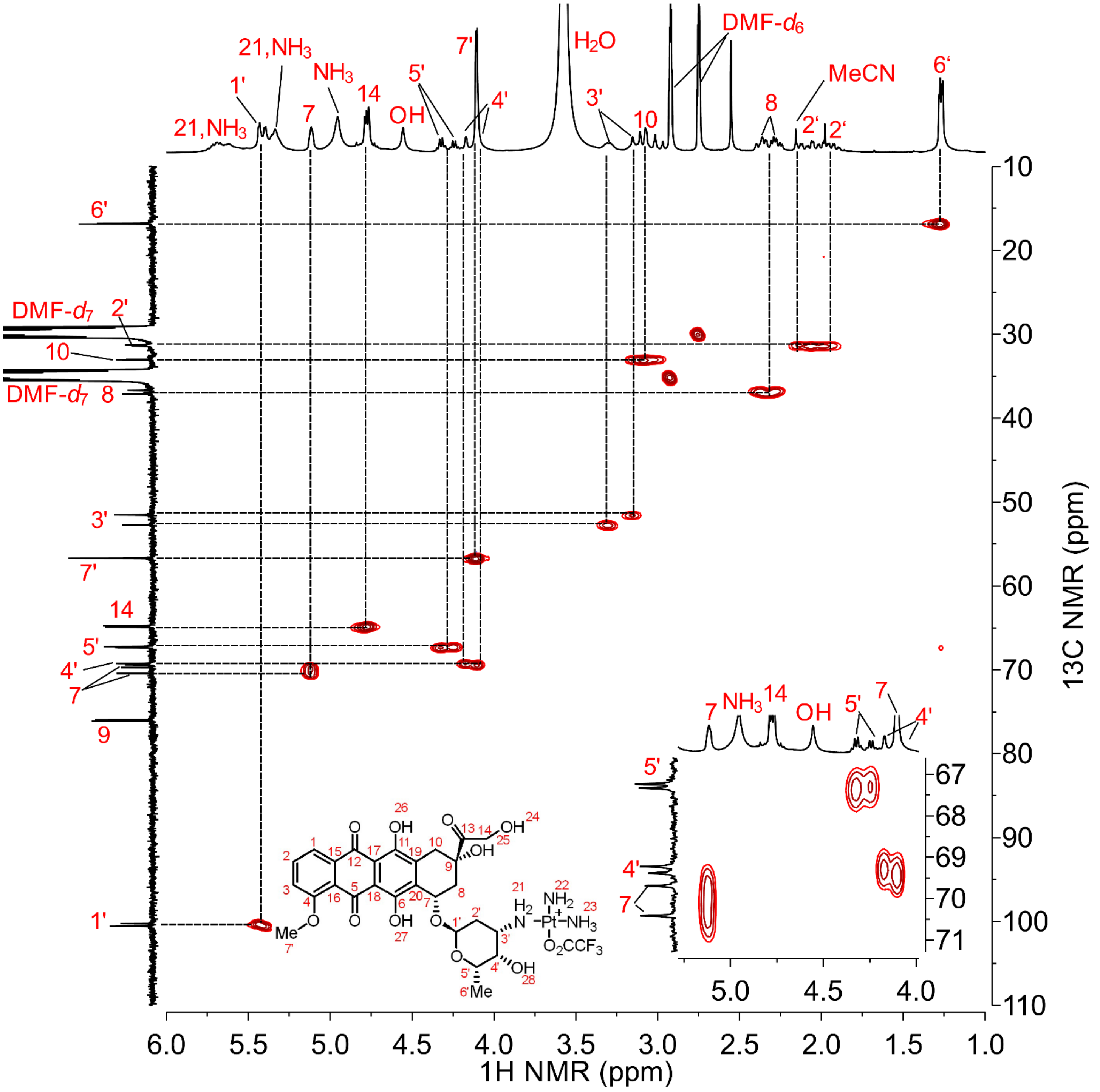

Figure S184. Expansion of ^1H-^{13}C HSQC spectrum of DoxPt4 in DMF-d_7 from 1.0 to 6.0 ppm (^1H) and 10 to 110 ppm (^{13}C). The free trifluoroacetate anion is omitted for clarity.

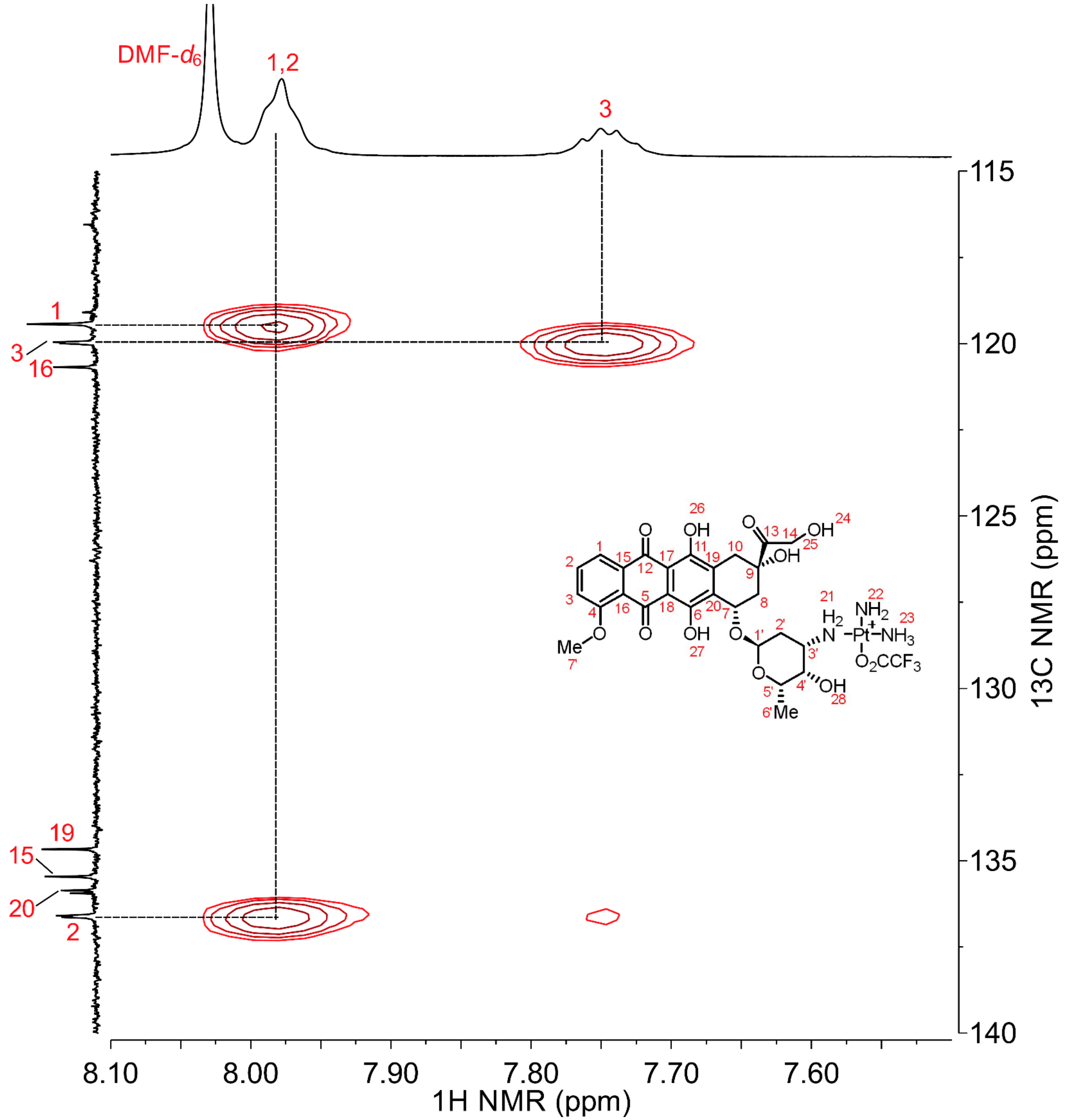

Figure S185. Expansion of ^{1}H-^{13}C HSQC spectrum of DoxPt4 in DMF-d_7 from 7.4 to 8.1 ppm (^{1}H) and 115 to 140 ppm (^{13}C). The free trifluoroacetate anion is omitted for clarity.

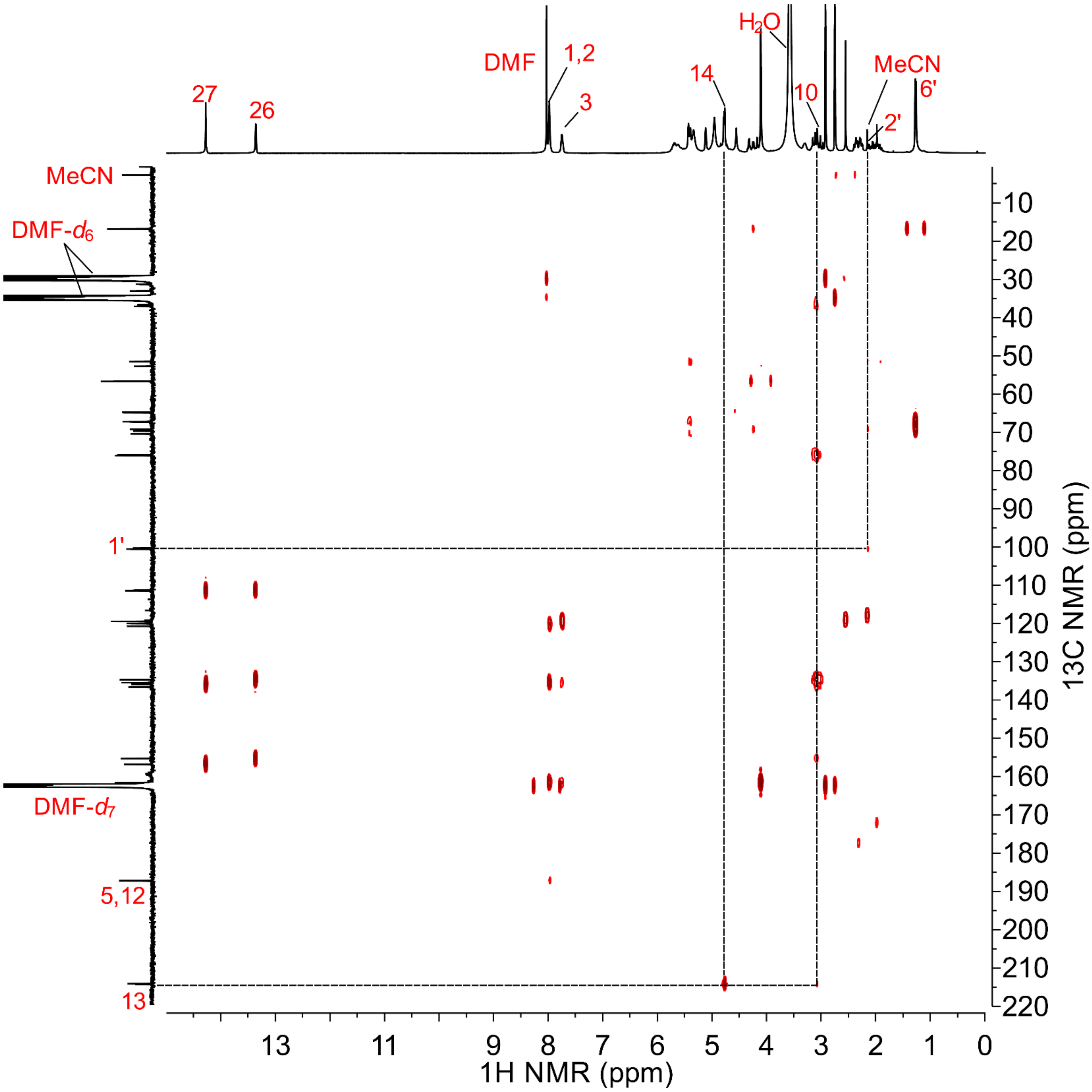

Figure S186. ^{1}H-^{13}C HMBC spectrum of DoxPt4 in DMF-d_7. The free trifluoroacetate anion is omitted for clarity.

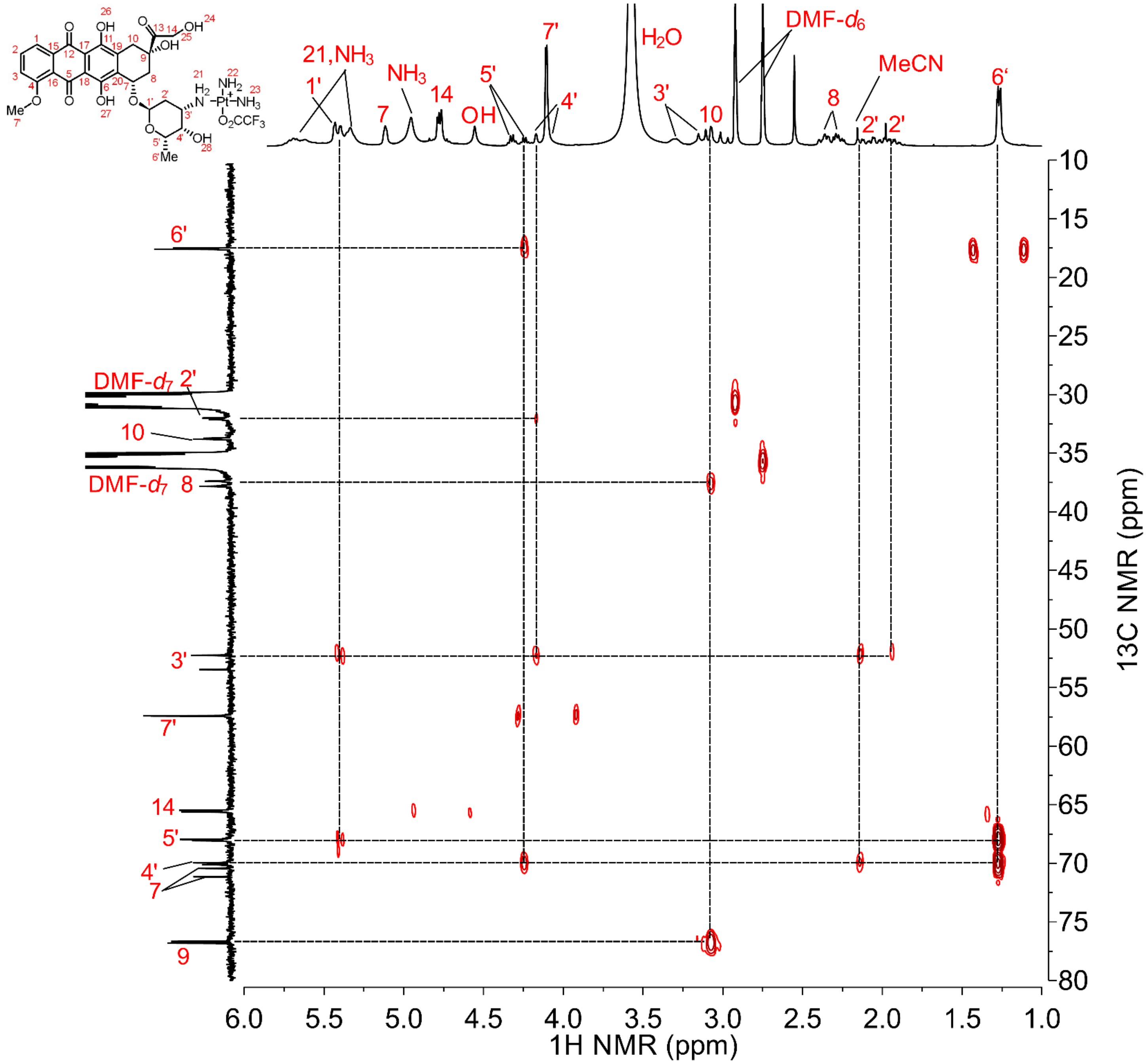

Figure S187. Expansion of ^{1}H-^{13}C HMBC spectrum of DoxPt4 in DMF-d_7 from 1.0 to 6.0 ppm (^{1}H) and 10 to 80 ppm (^{13}C). The free trifluoroacetate anion is omitted for clarity.

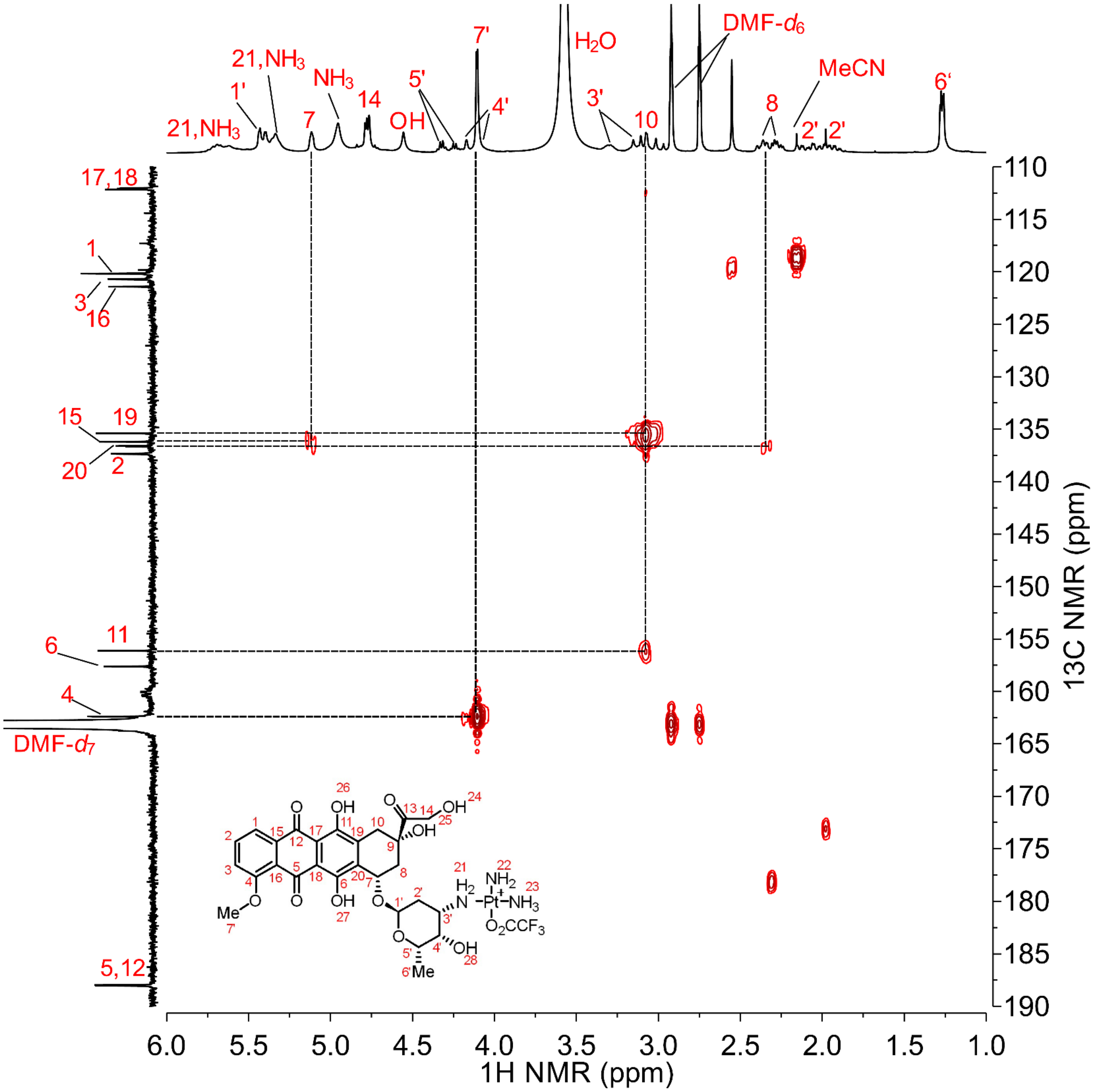

Figure S188. Expansion of ^{1}H-^{13}C HMBC spectrum of DoxPt4 in DMF-d_7 from 1.0 to 6.0 ppm (^{1}H) and 110 to 190 ppm (^{13}C). The free trifluoroacetate anion is omitted for clarity.

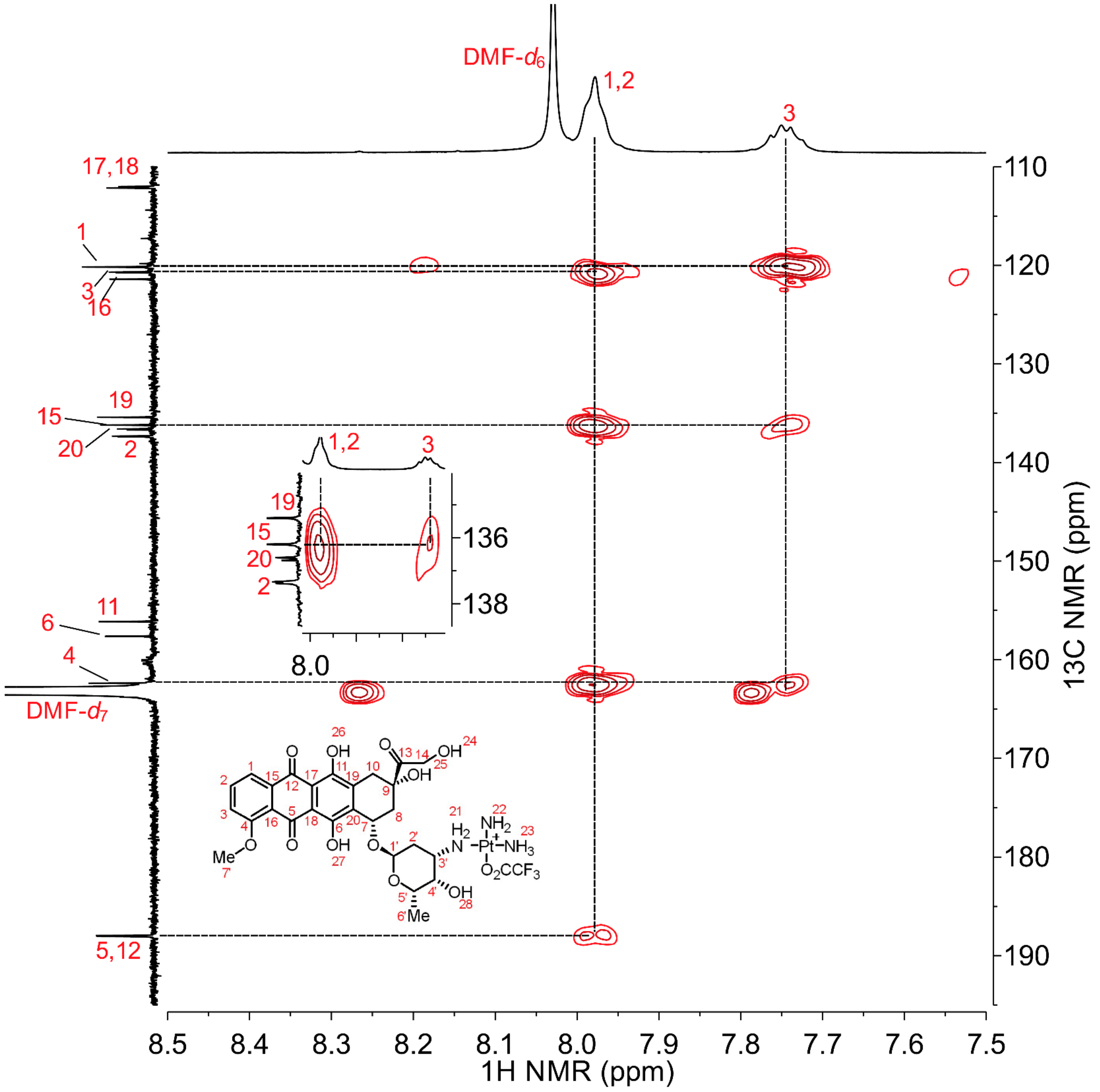

Figure S189. Expansion of ^{1}H-^{13}C HMBC spectrum of DoxPt4 in DMF-d_7 from 7.5 to 8.5 ppm (^{1}H) and 110 to 195 ppm (^{13}C). The free trifluoroacetate anion is omitted for clarity.

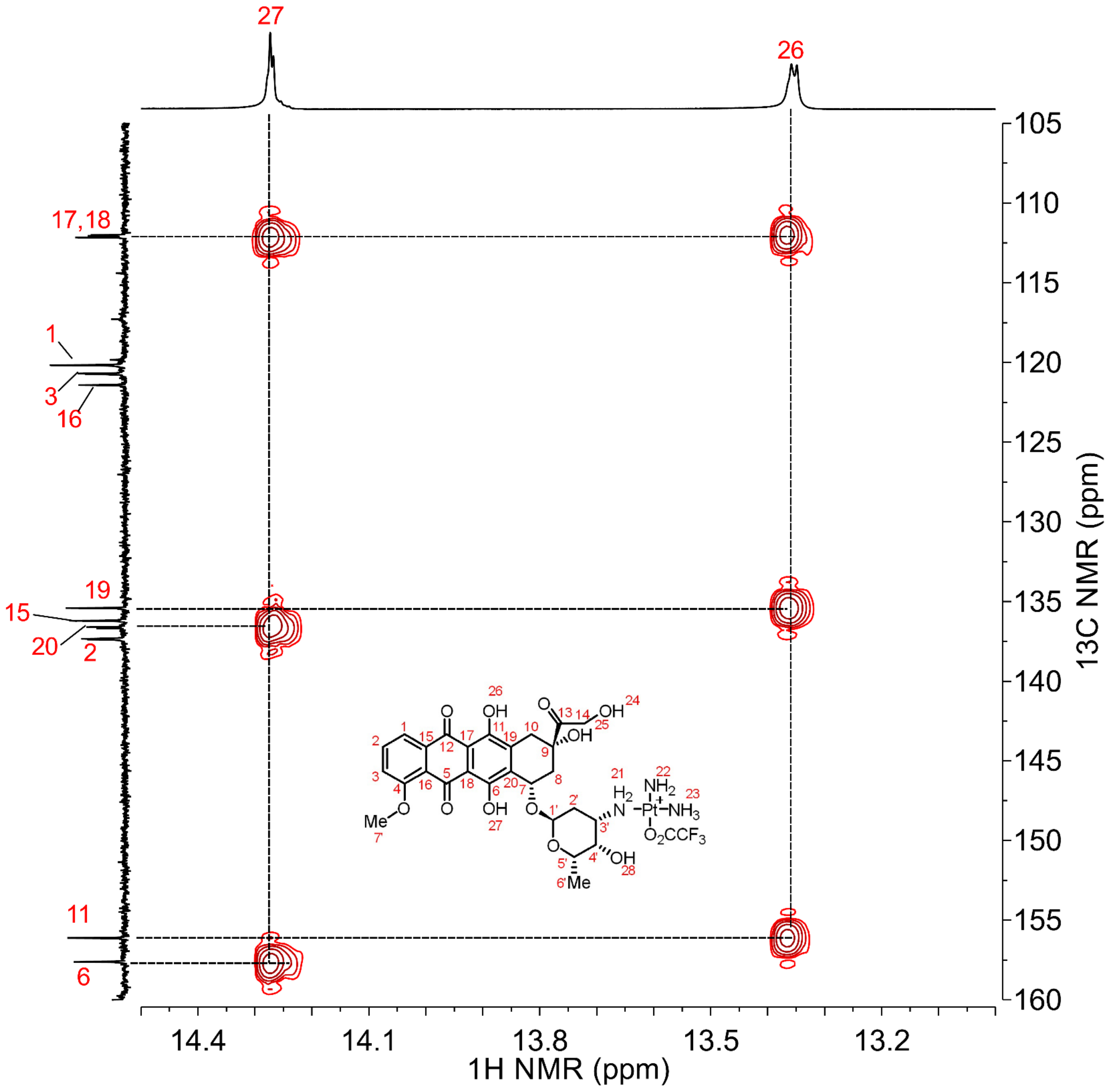

Figure S190. Expansion of ^{1}H-^{13}C HMBC spectrum of DoxPt4 in DMF-d_7 from 13.0 to 14.5 ppm (^{1}H) and 105 to 160 ppm (^{13}C). The free trifluoroacetate anion is omitted for clarity.

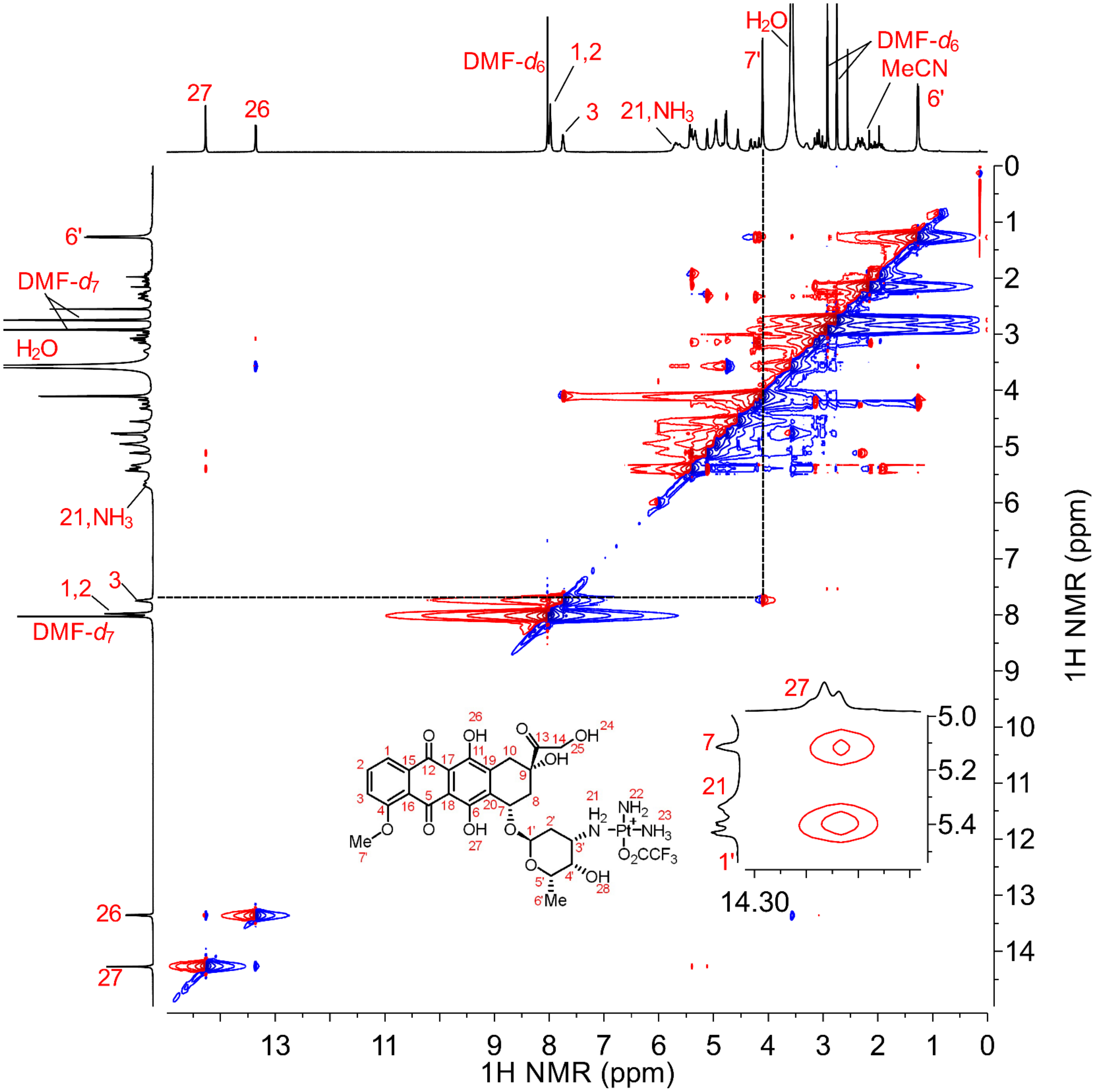

Figure S191. ^{1}H-^{1}H ROESY spectrum of DoxPt4 in DMF-d_7. The free trifluoroacetate anion is omitted for clarity.

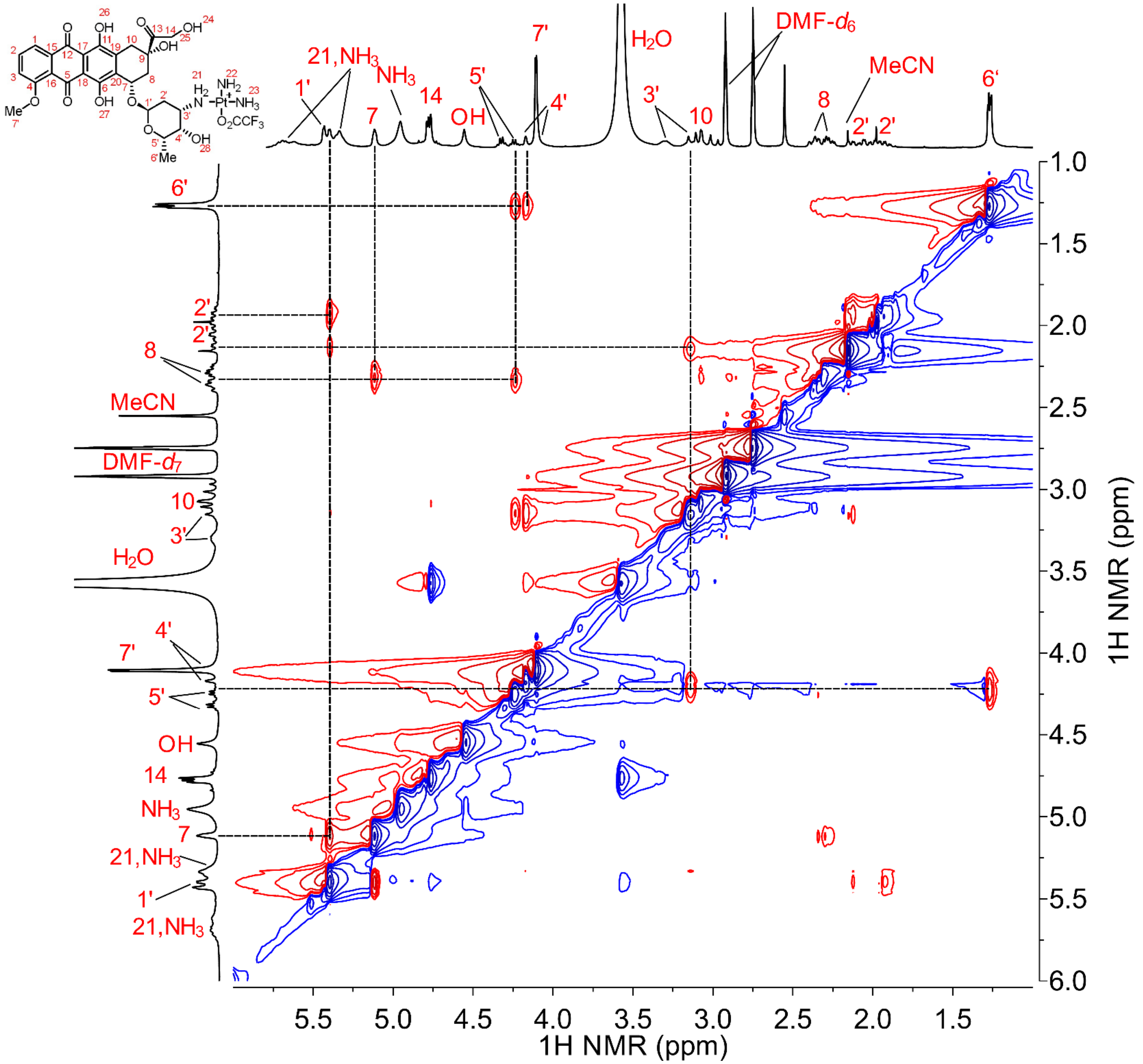

Figure S192. Expansion of ^1H-^1H ROESY spectrum of DoxPt4 in DMF-d_7 from 1.0 to 6.0 ppm. The free trifluoroacetate anion is omitted for clarity.

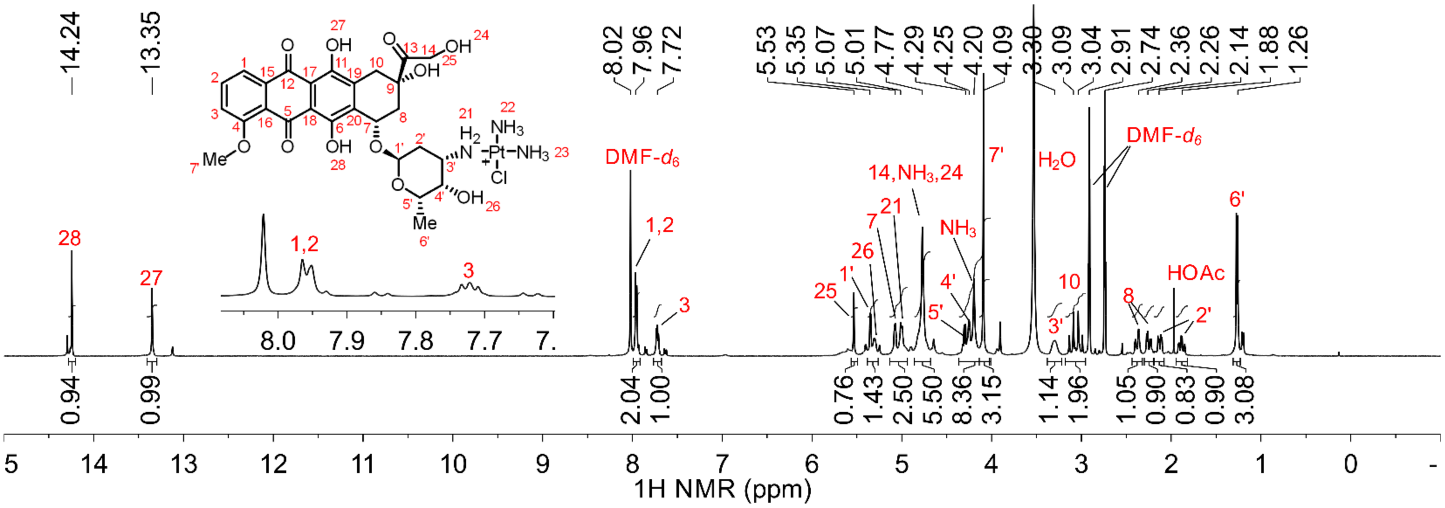

Figure S193. ^{1}H NMR spectrum of DoxPt5 in DMF-d_7. The trifluoroacetate anion is omitted for clarity.

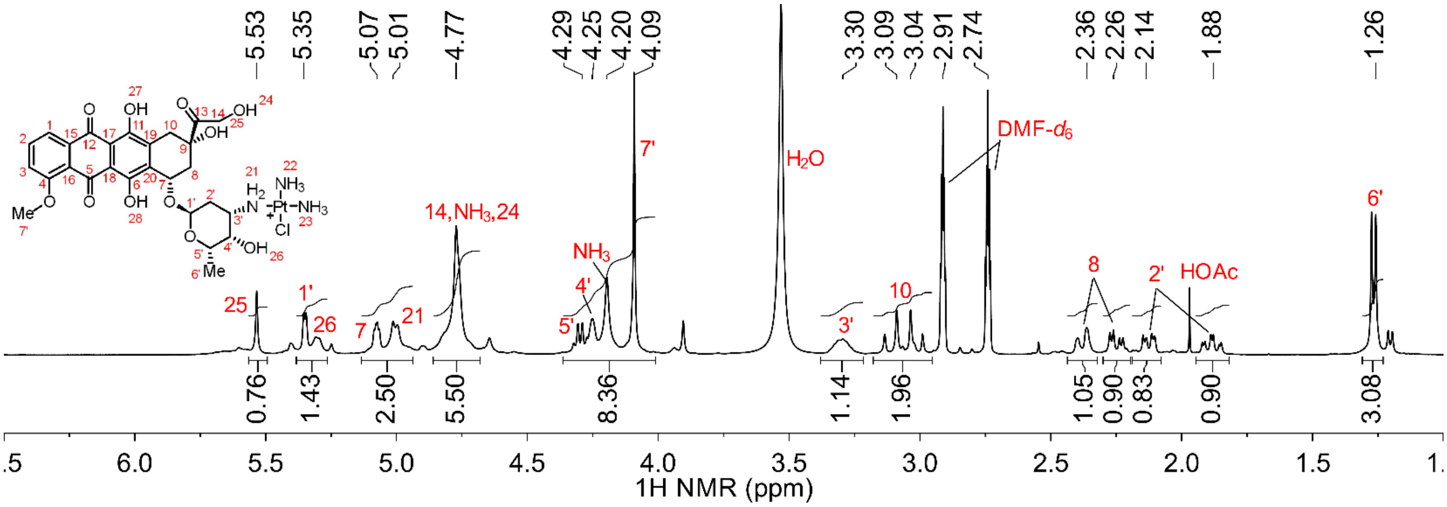

Figure S194. Expansion of ^{1}H NMR spectrum of DoxPt5 in DMF-d_7 from 1.0 to 6.5 ppm. The trifluoroacetate anion is omitted for clarity.

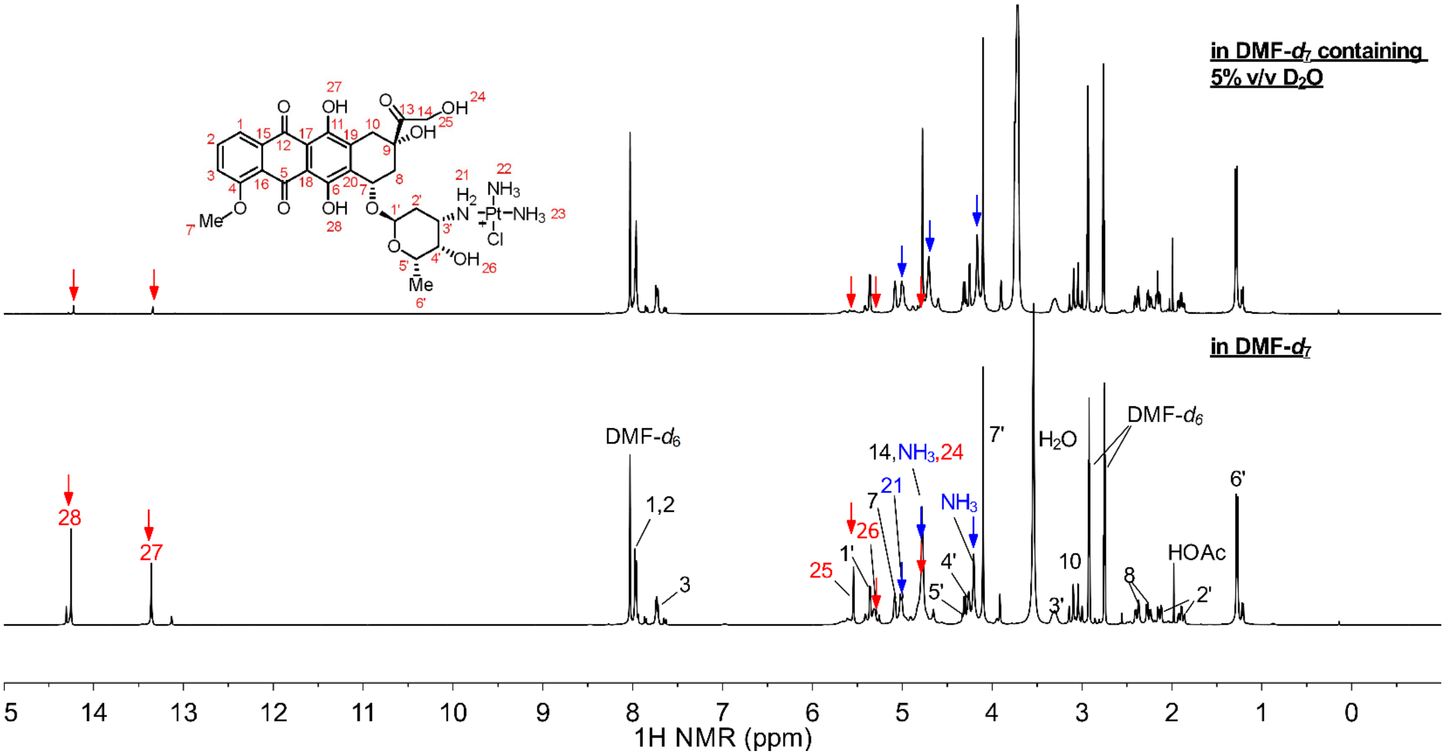

Figure S195. Comparison of ^{1}H NMR spectrum of DoxPt5 in DMF-d_7 and in DMF-d_7/D_2O (95:5, v/v). Exchangable protons of the OH groups are indicated with red arrows. Protons of the N–H groups are indicated with blue arrows. The trifluoroacetate anion is omitted for clarity.

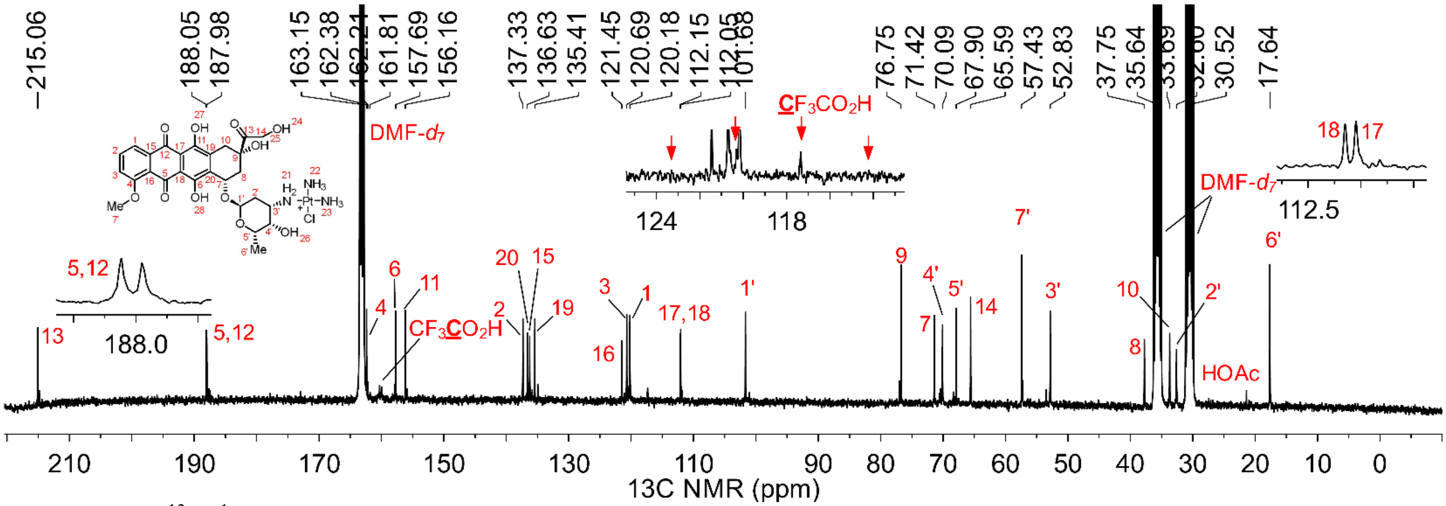

Figure S196. $^{13}C\{^{1}H\}$ NMR spectrum of DoxPt5 in DMF-d_7. The trifluoroacetate anion is omitted for clarity.

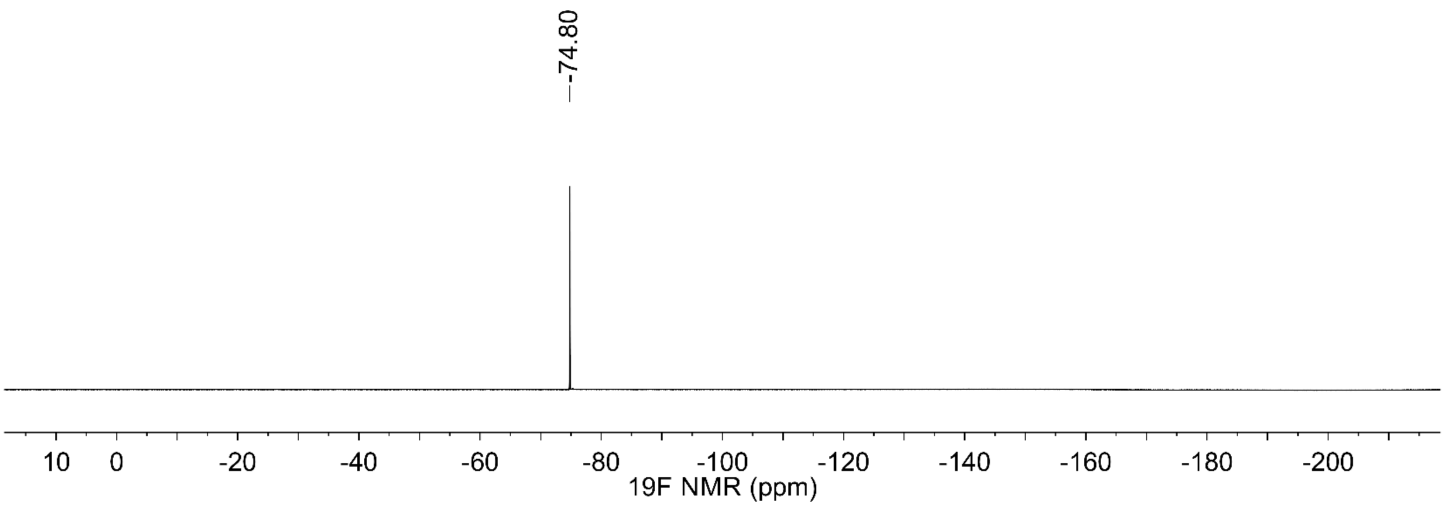

Figure S197. ^{19}F NMR spectrum of DoxPt5 in DMF-d_7.

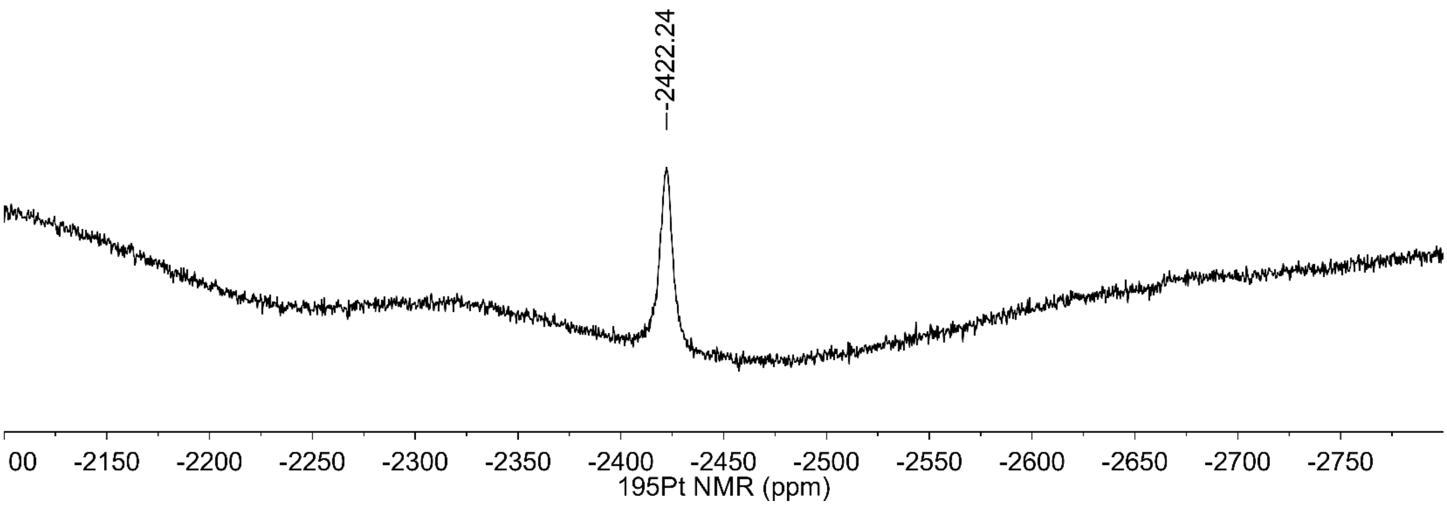

Figure S198. ^{195}Pt NMR spectrum of DoxPt5 in DMF-d_7.

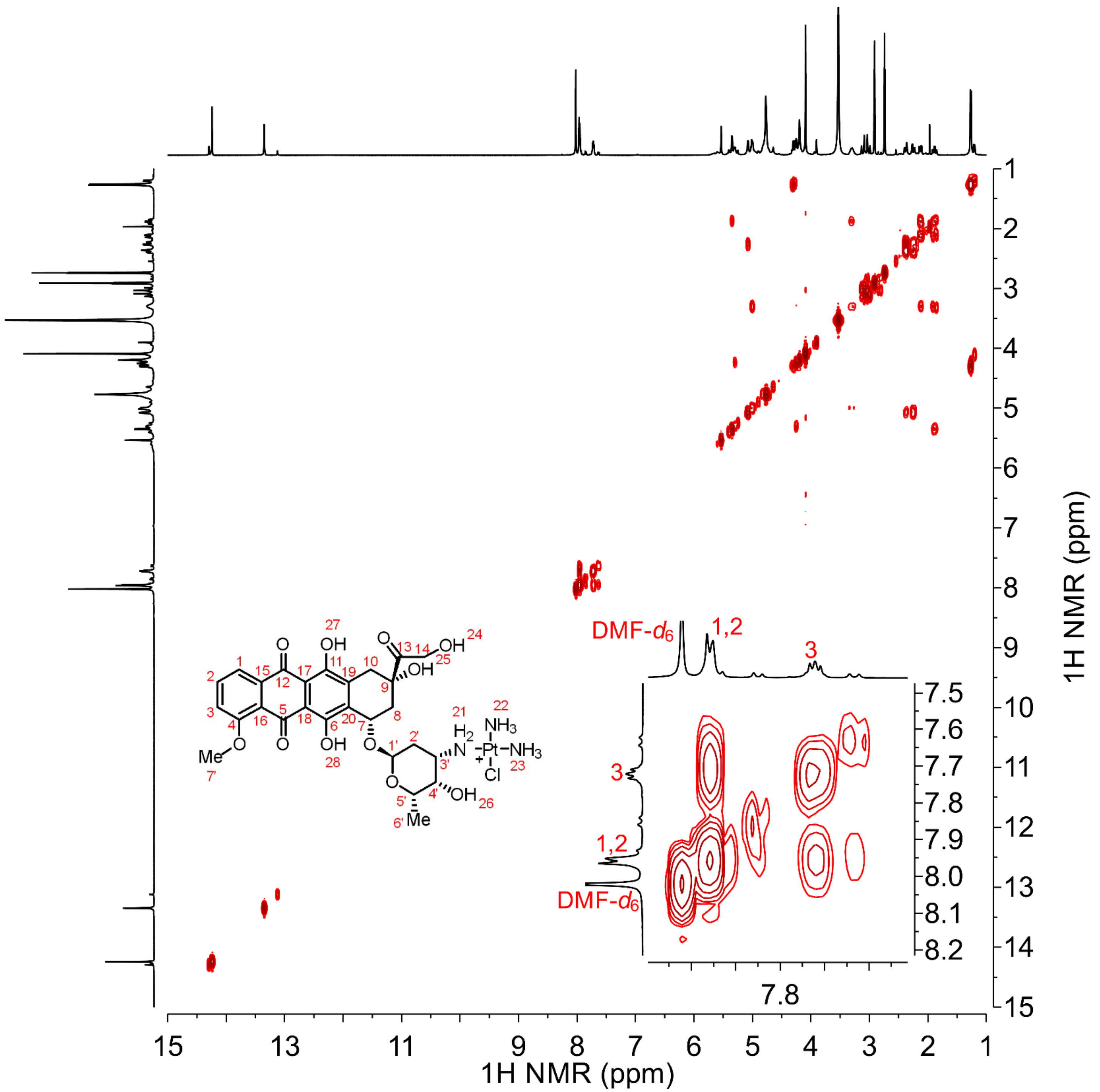

Figure S199. ^{1}H-^{1}H COSY spectrum of DoxPt5 in DMF-d_7. The trifluoroacetate anion is omitted for clarity.

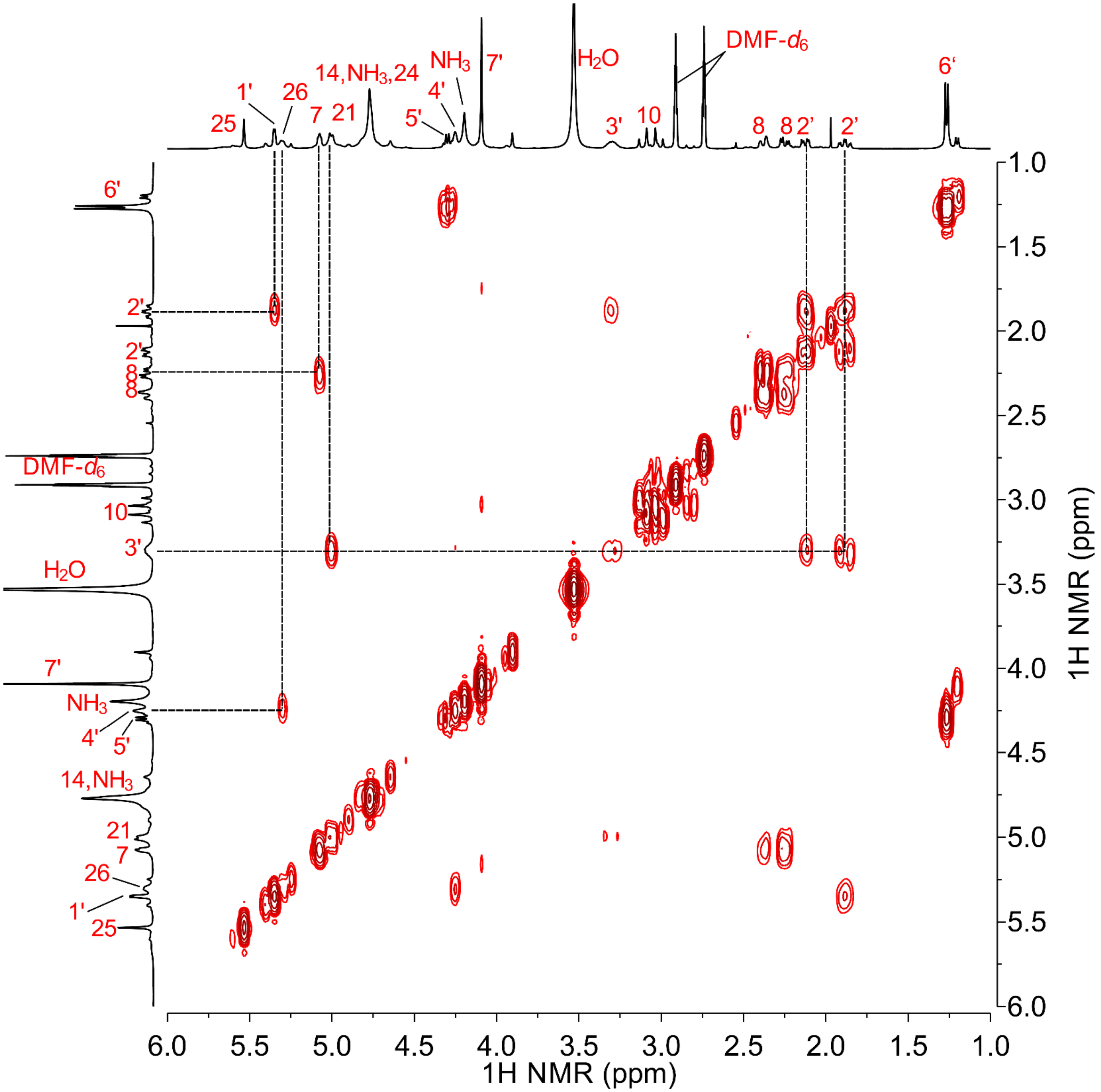

Figure S200. Expansion of ^{1}H-^{1}H COSY spectrum of DoxPt5 in DMF-d_7 from 1.0 to 6.0 ppm.

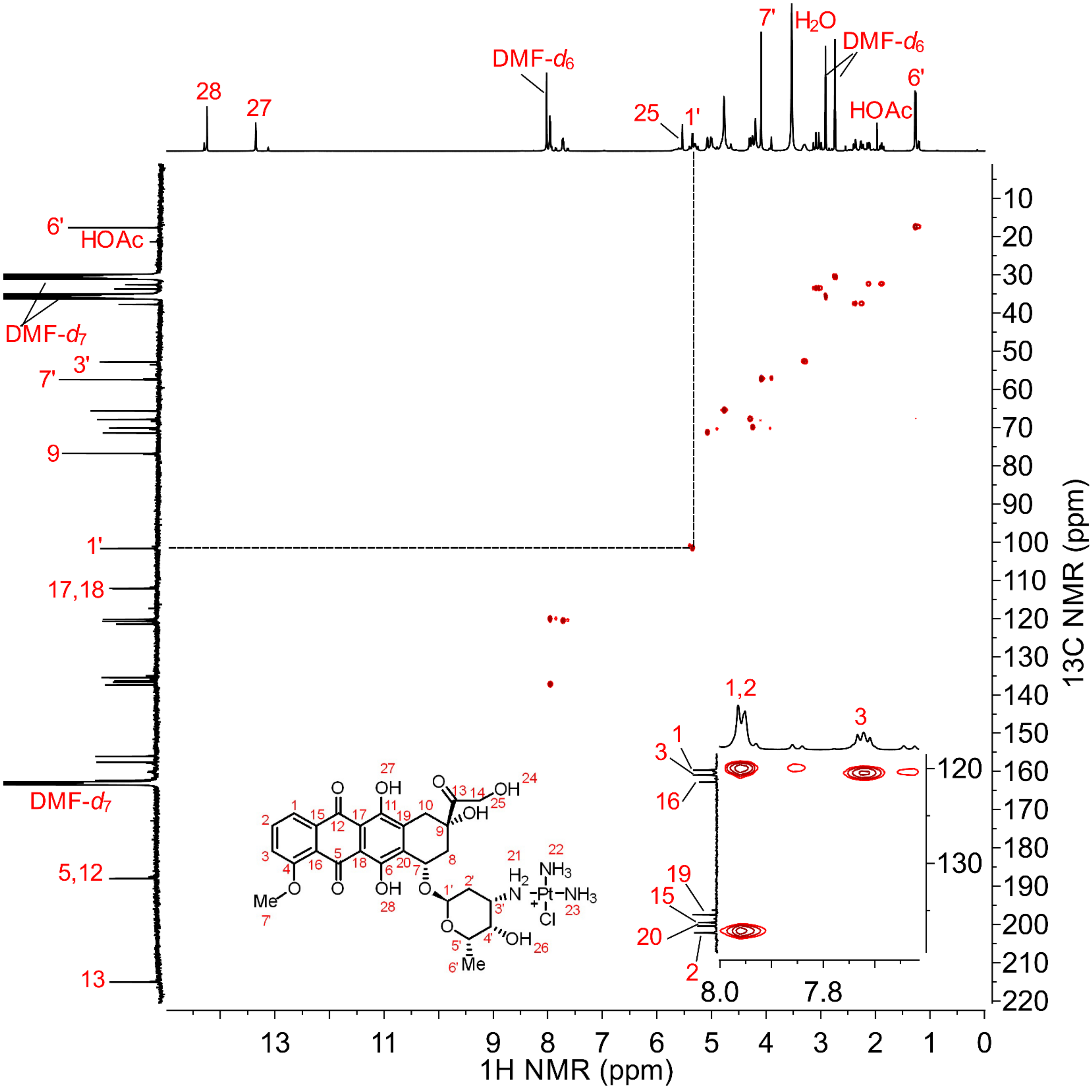

Figure S201. ^{1}H-^{13}C HSQC spectrum of DoxPt5 in DMF-d_7. The trifluoroacetate anion is omitted for clarity.

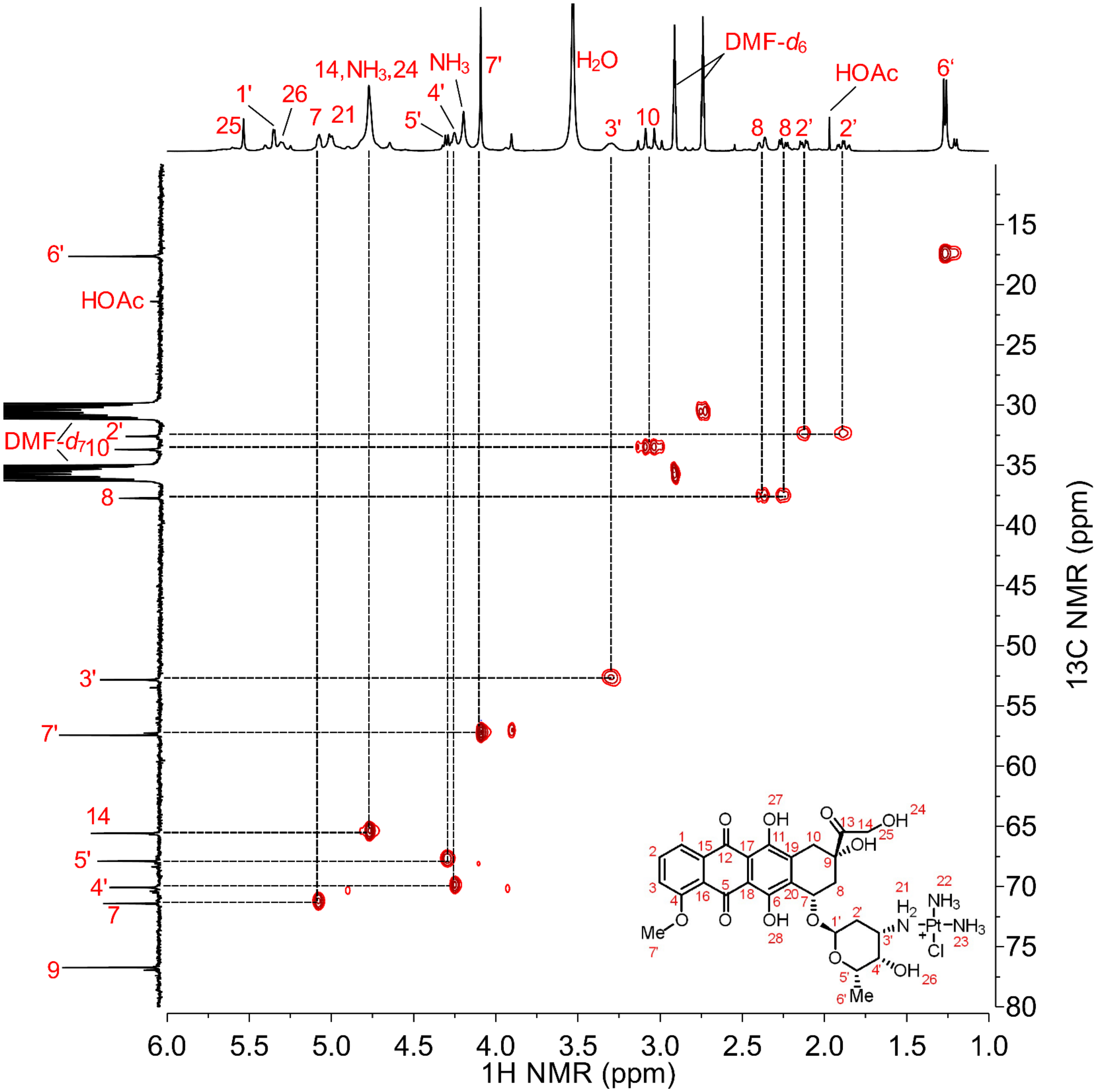

Figure S202. Expansion of ^{1}H-^{13}C HSQC spectrum of DoxPt5 in DMF-d_7 from 1.0 to 6.0 ppm (^{1}H) and 10 to 80 ppm (^{13}C). The trifluoroacetate anion is omitted for clarity.

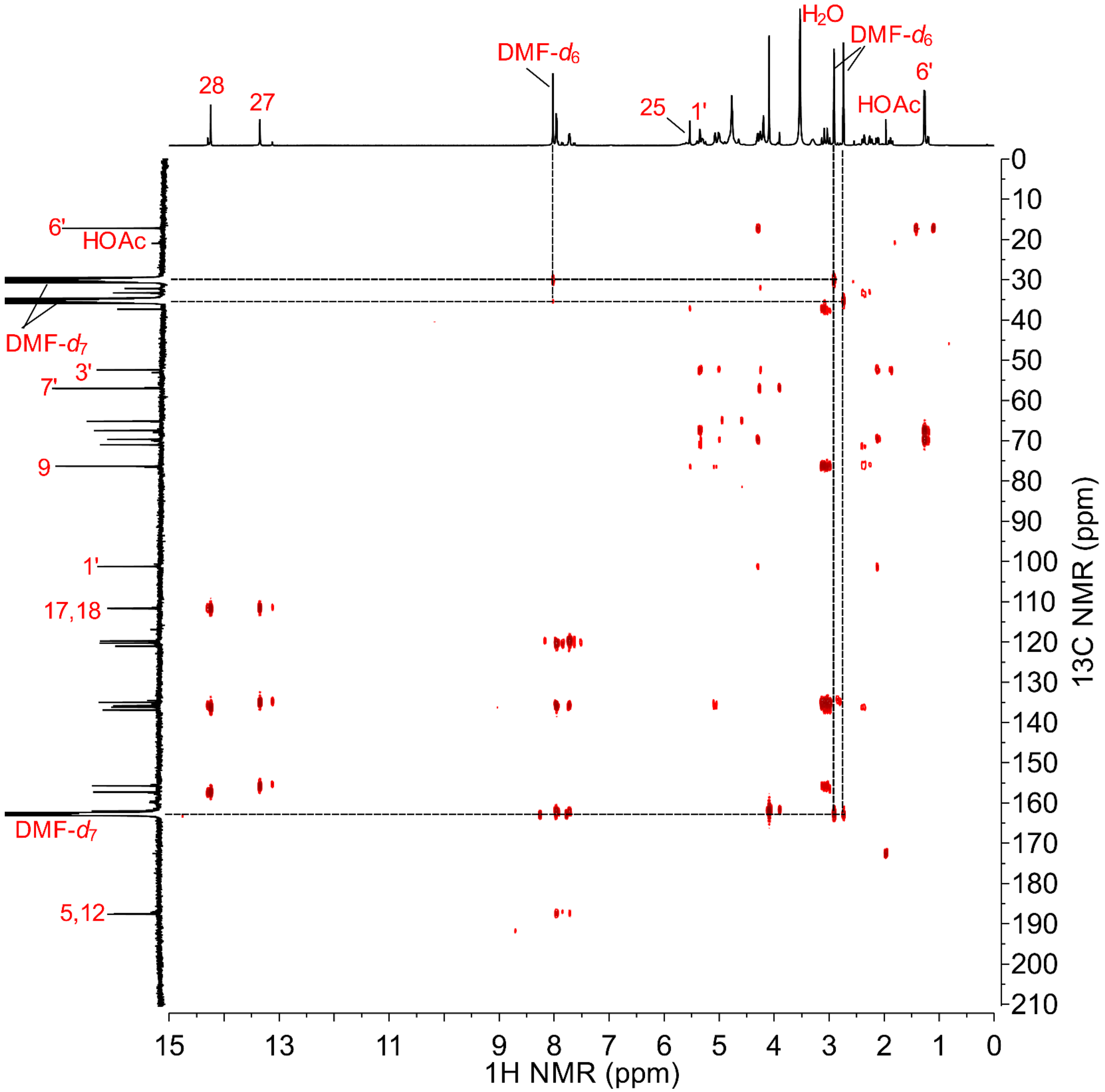

Figure S203. ^{1}H-^{13}C HMBC spectrum of DoxPt5 in DMF-d_7. The trifluoroacetate anion is omitted for clarity.

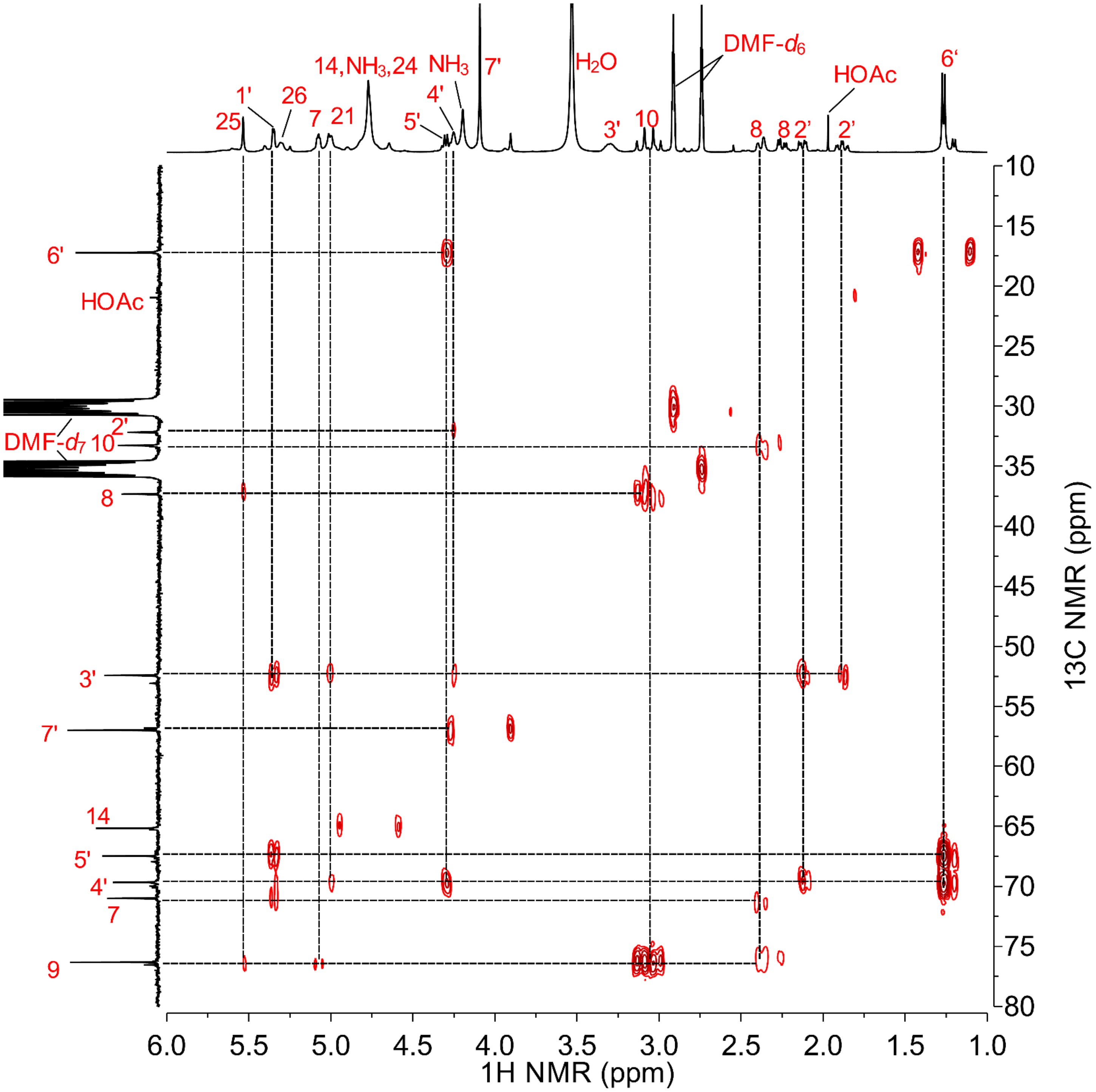

Figure S204. Expansion of ^{1}H-^{13}C HMBC spectrum of DoxPt5 in DMF-d_7 from 1.0 to 6.0 ppm (^{1}H) and 10 to 80 ppm (^{13}C).

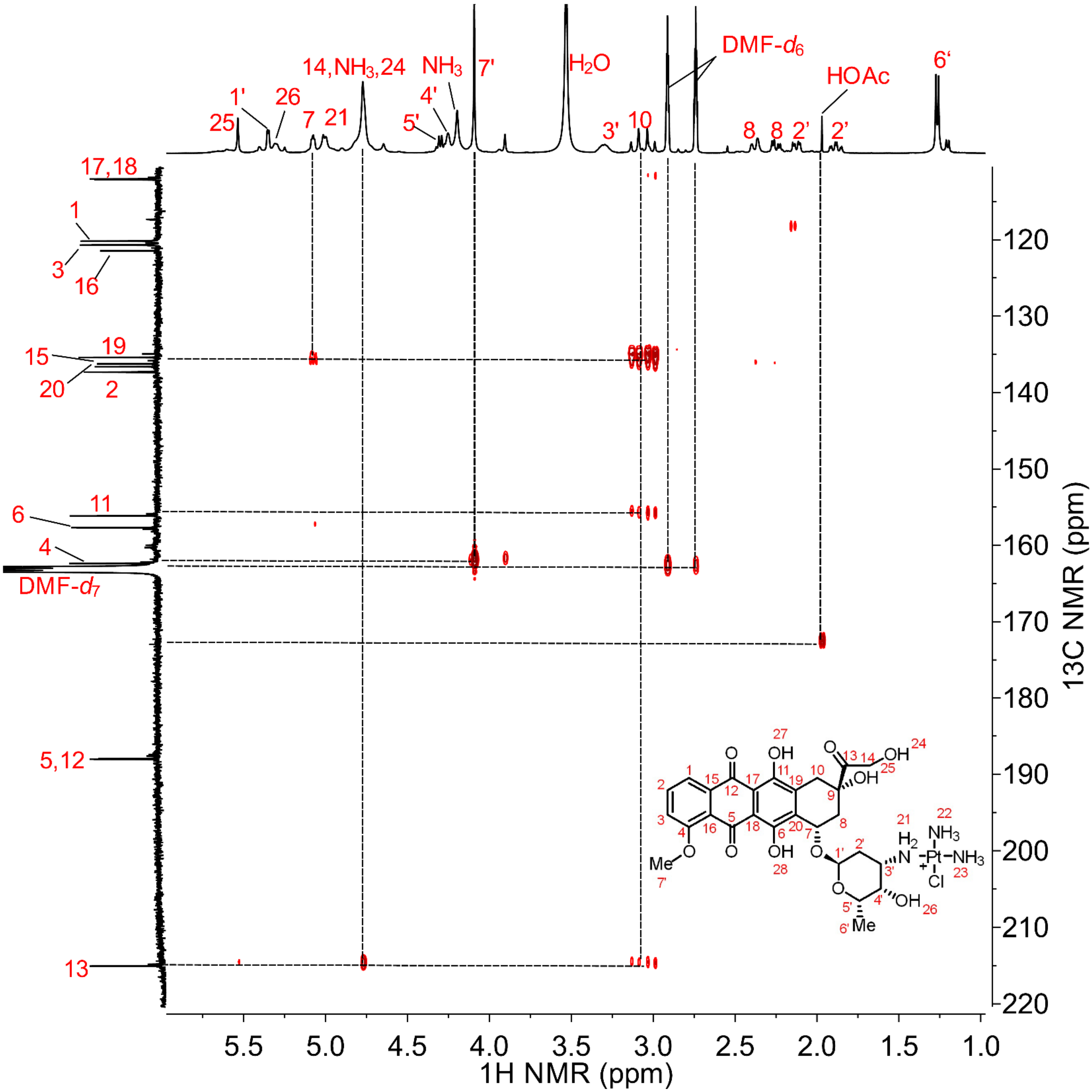

Figure S205. Expansion of ^{1}H-^{13}C HMBC spectrum of DoxPt5 in DMF-d_7 from 1.0 to 6.0 ppm (^{1}H) and 110 to 220 ppm (^{13}C). The free trifluoroacetate anion is omitted for clarity.

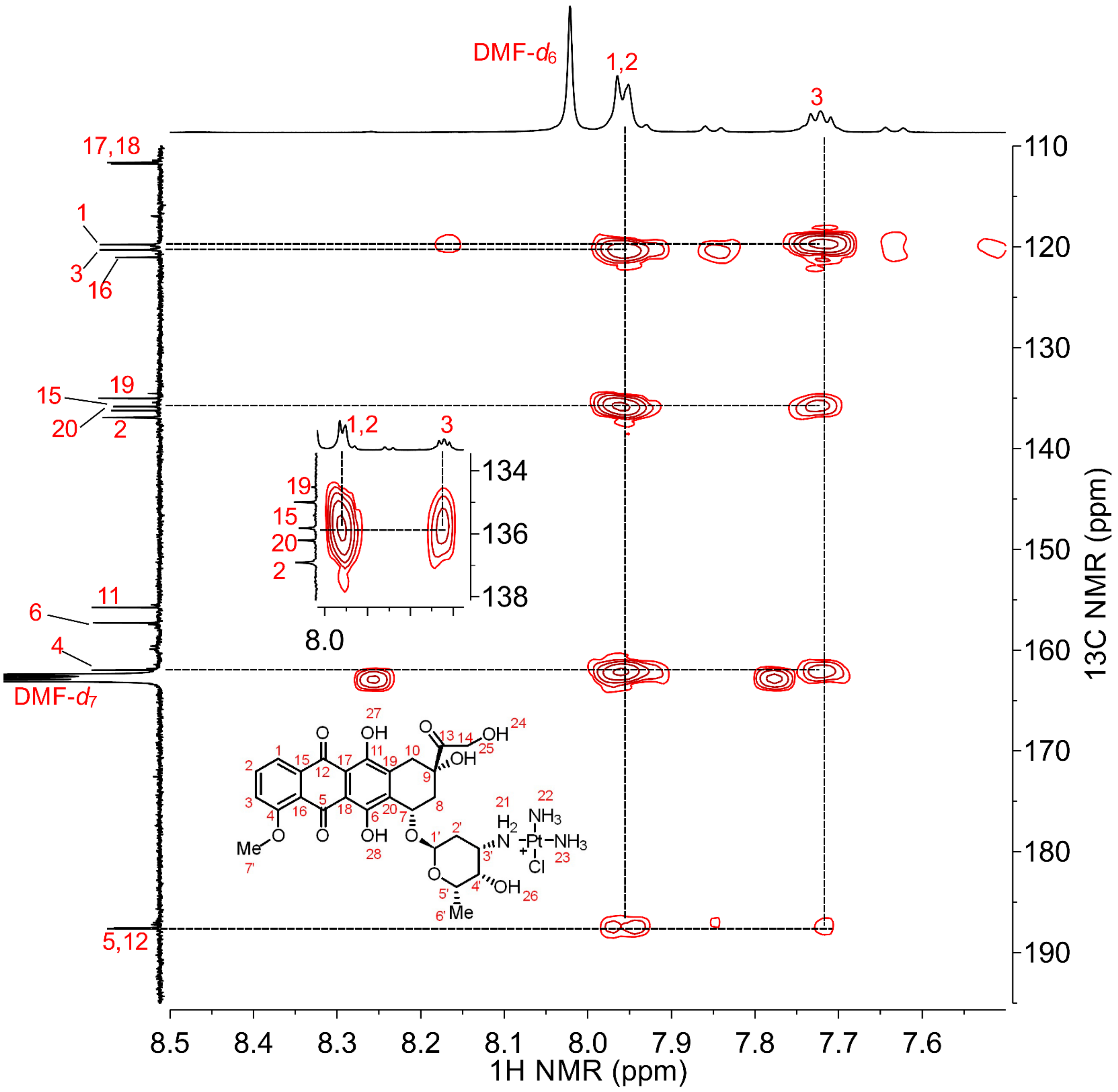

Figure S206. Expansion of ^{1}H-^{13}C HMBC spectrum of DoxPt5 in DMF-d_7 from 7.5 to 8.5 ppm (^{1}H) and 110 to 195 ppm (^{13}C). The free trifluoroacetate anion is omitted for clarity.

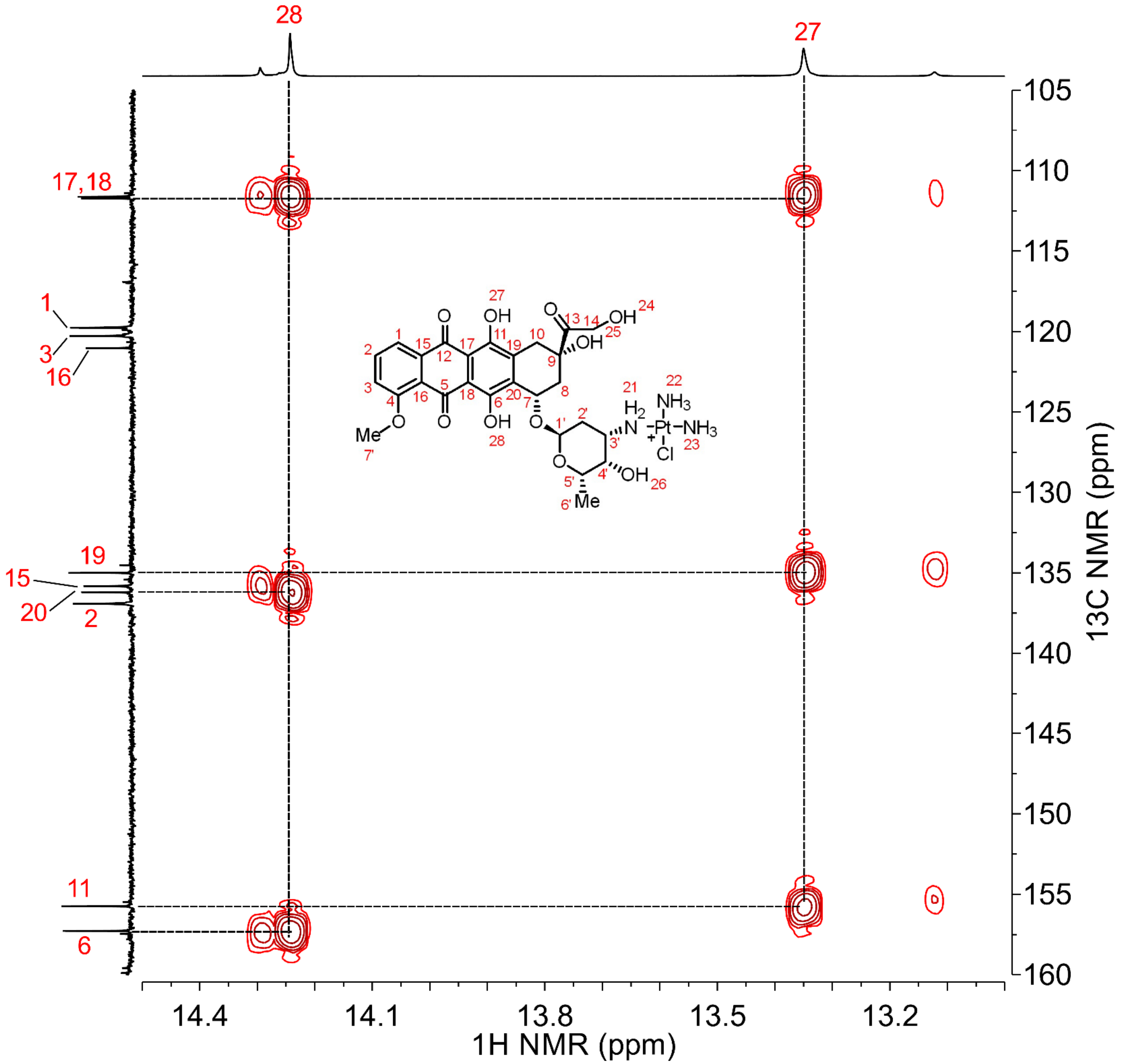

Figure S207. Expansion of ^{1}H-^{13}C HMBC spectrum of DoxPt5 in DMF-d_7 from 13.0 to 14.5 ppm (^{1}H) and 105 to 160 ppm (^{13}C). The free trifluoroacetate anion is omitted for clarity.

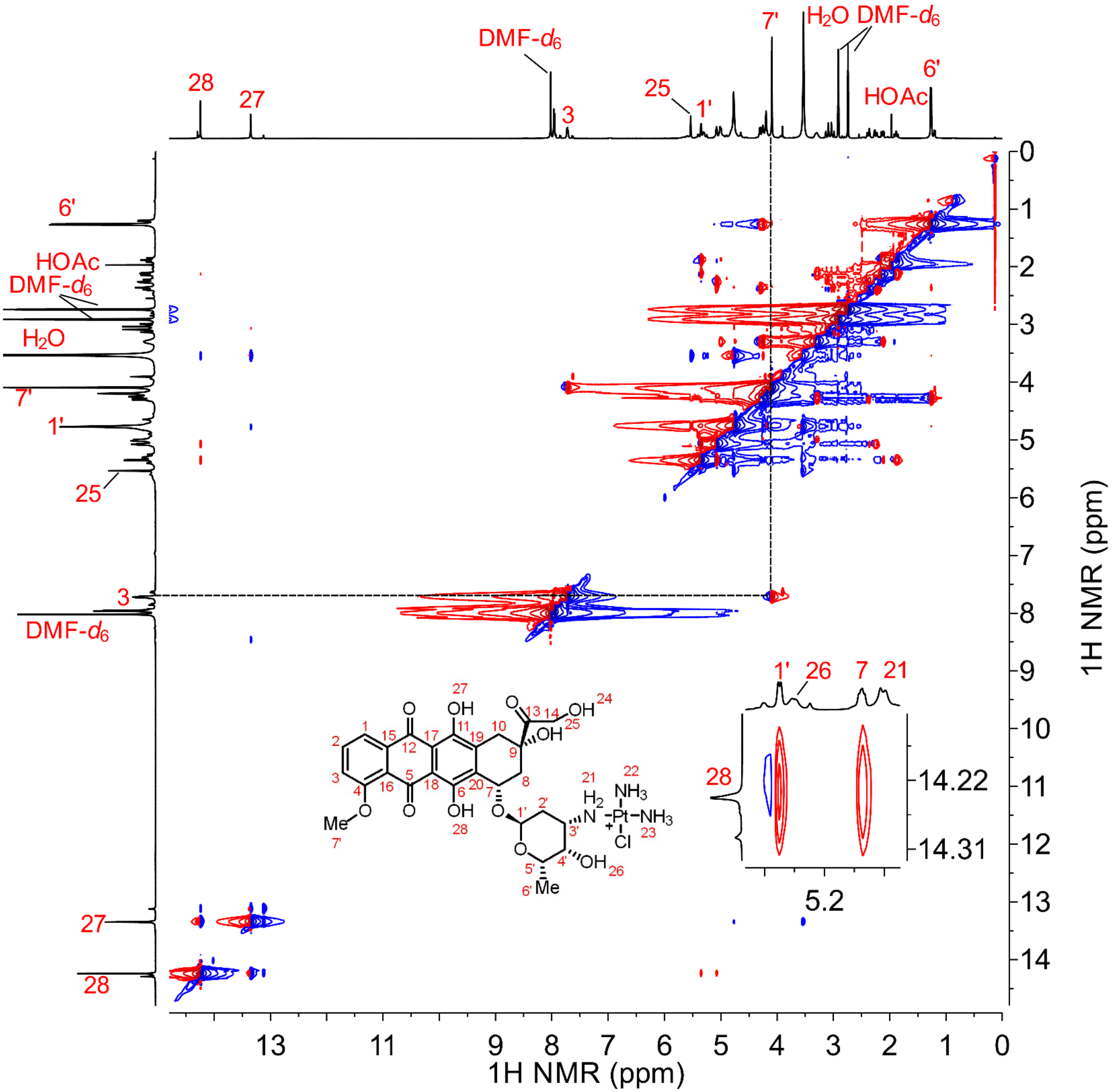

Figure S208. ^{1}H-^{1}H ROESY spectrum of DoxPt5 in DMF-d_7. The free trifluoroacetate anion is omitted for clarity.

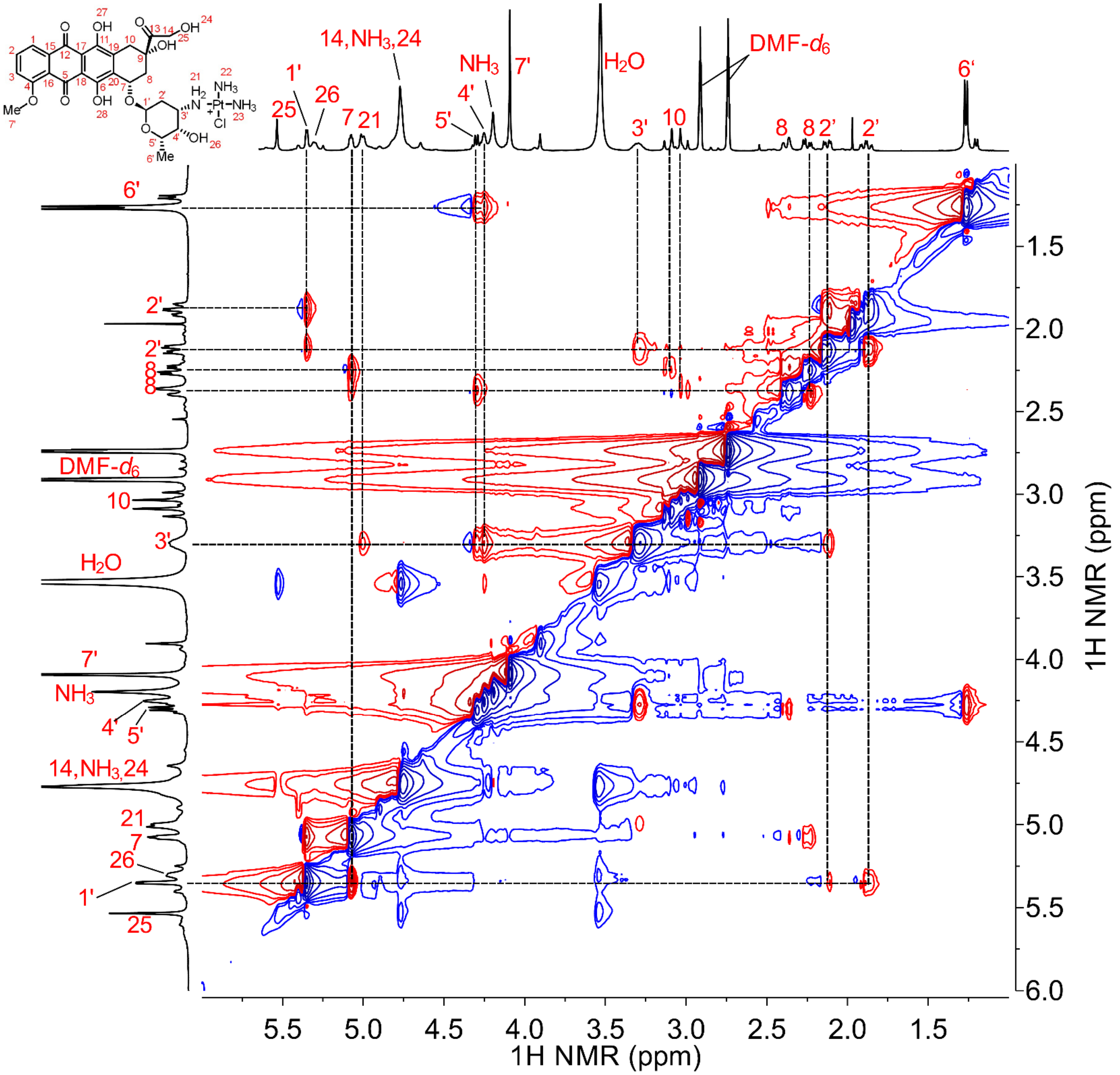

Figure S209. Expansion of ^{1}H-^{1}H ROESY spectrum of DoxPt5 in DMF-d_7 from 1.0 to 6.0 ppm. The free trifluoroacetate anion is omitted for clarity.

S17. References.

1 Fulmer, G. R. *et al.* NMR Chemical Shifts of Trace Impurities: Common Laboratory Solvents, Organics, and Gases in Deuterated Solvents Relevant to the Organometallic Chemist. *Organometallics* **29**, 2176-2179 (2010).

2 Di Veroli, G. Y. *et al.* An automated fitting procedure and software for dose-response curves with multiphasic features. *Sci. Rep.* **5**, 14701 (2015).

3 Cotterill, I. C. & Rich, J. O. Chemoenzymatic Synthesis of *N*-Trifluoroacetyl Doxorubicin-14-Valerate (Valrubicin). *Org. Process Res. Dev.* **9**, 818-821 (2005).

4 Acton, E. M. & Tong, G. L. Synthesis and preliminary antitumor evaluation of 5-iminodoxorubicin. *J. Med. Chem.* **24**, 669-673 (1981).

5 Varbanov, H. P. *et al.* Oxaliplatin reacts with DMSO only in the presence of water. *Dalton Trans.* **46**, 8929-8932 (2017).

6 Jerremalm, E. *et al.* Alkaline Hydrolysis of Oxaliplatin—Isolation and Identification of the Oxalato Monodentate Intermediate. *J. Pharm. Sci.* **91**, 2116-2121 (2002).

7 Chou, T.-C. Drug Combination Studies and Their Synergy Quantification Using the Chou-Talalay Method. *Cancer Res.* **70**, 440-446 (2010).

8 Sánchez-Rivera, F. J. *et al.* Rapid modelling of cooperating genetic events in cancer through somatic genome editing. *Nature* **516**, 428-431 (2014).

9 Jiang, H., Pritchard, J. R., Williams, R. T., Lauffenburger, D. A. & Hemann, M. T. A mammalian functional-genetic approach to characterizing cancer therapeutics. *Nat. Chem. Biol.* **7**, 92-100 (2011).

10 Dickins, R. A. *et al.* Probing tumor phenotypes using stable and regulated synthetic microRNA precursors. *Nat. Genet.* **37**, 1289-1295 (2005).

11 Zuber, J. *et al.* Toolkit for evaluating genes required for proliferation and survival using tetracycline-regulated RNAi. *Nat. Biotechnol.* **29**, 79-83 (2011).

12 Pritchard, J. R., Bruno, P. M., Hemann, M. T. & Lauffenburger, D. A. Predicting cancer drug mechanisms of action using molecular network signatures. *Mol. BioSyst.* **9**, 1604-1619 (2013).

13 Bruno, P. M. *et al.* A subset of platinum-containing chemotherapeutic agents kills cells by inducing ribosome biogenesis stress. *Nat. Med.* **23**, 461 (2017).

14 Shoemaker, R. H. The NCI60 Human Tumour Cell Line Anticancer Drug Screen. *Nat. Rev. Cancer* **6**, 813 (2006).

15 Reinhold, W. C., Sunshine, M., Varma, S., Doroshow, J. H. & Pommier, Y. Using CellMiner 1.6 for Systems Pharmacology and Genomic Analysis of the NCI-60. *Clin. Cancer. Res.* **21**, 3841-3852 (2015).
