# Supplementary figures and images for: Leveraging platinum-protein interactions to overcome chemoresistance
Source: Nat Commun. 2025 Oct 20;16:9263. doi: 10.1038/s41467-025-64295-0 (PMC12537935; doi:10.1038/s41467-025-64295-0)

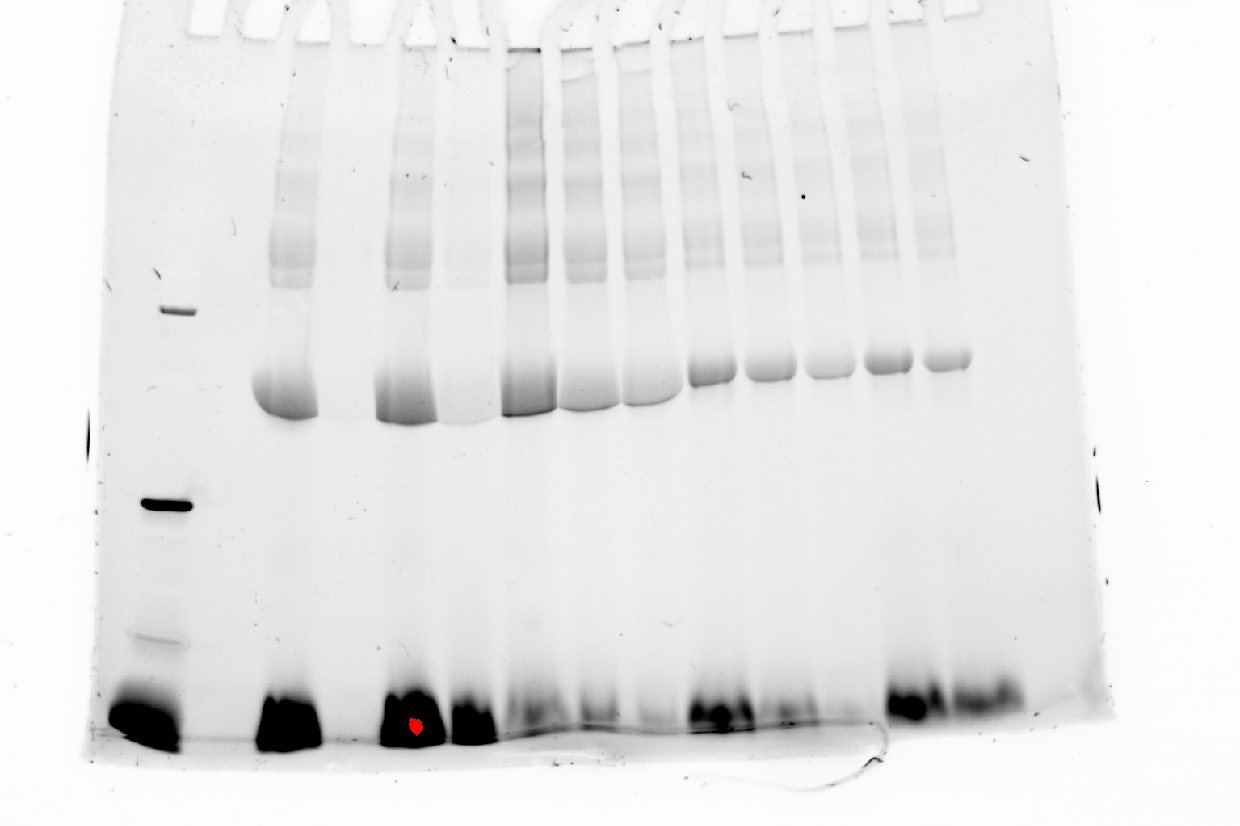

Supplement: Supplementary file 4 — Source data [file 41467_2025_64295_MOESM4_ESM.zip › Excel files/Source Data Figure 38.tif]

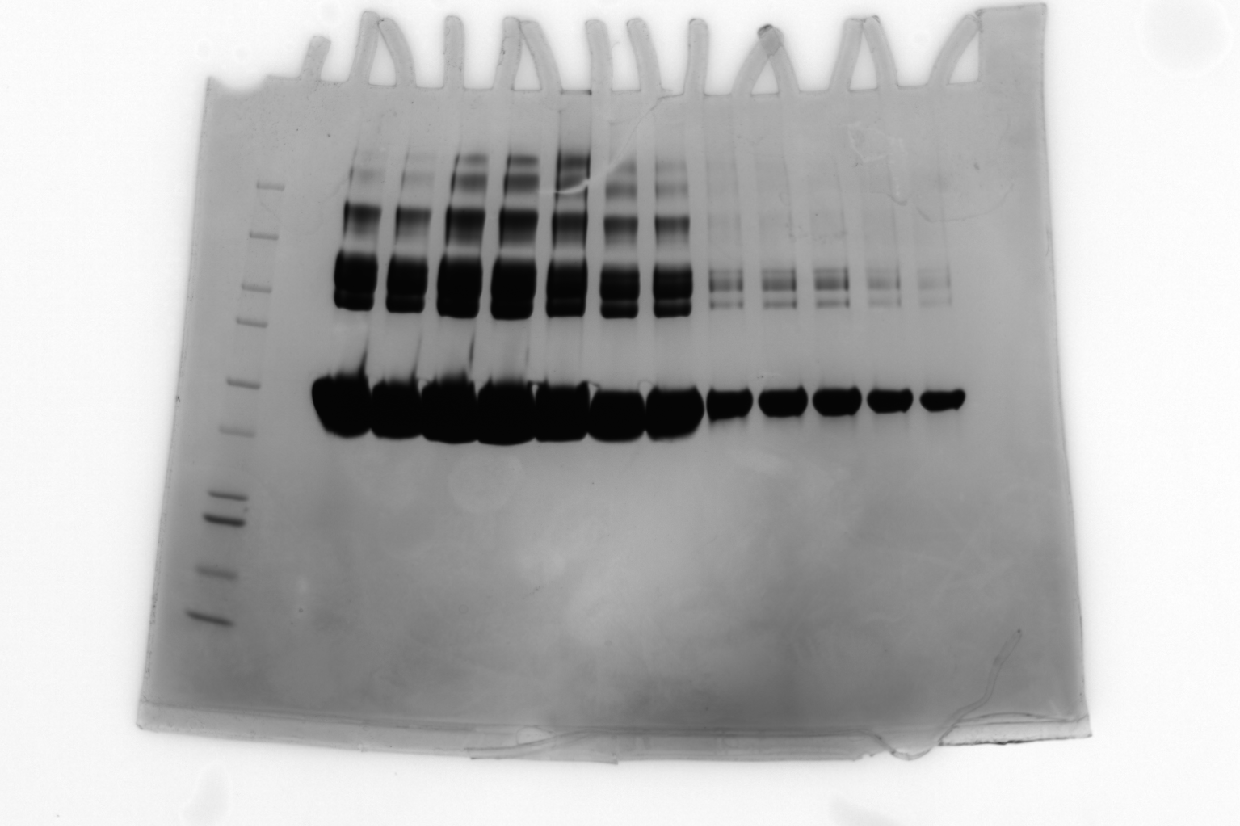

Supplement: Supplementary file 4 — Source data [file 41467_2025_64295_MOESM4_ESM.zip › Excel files/Source Data Figure 39.tif]

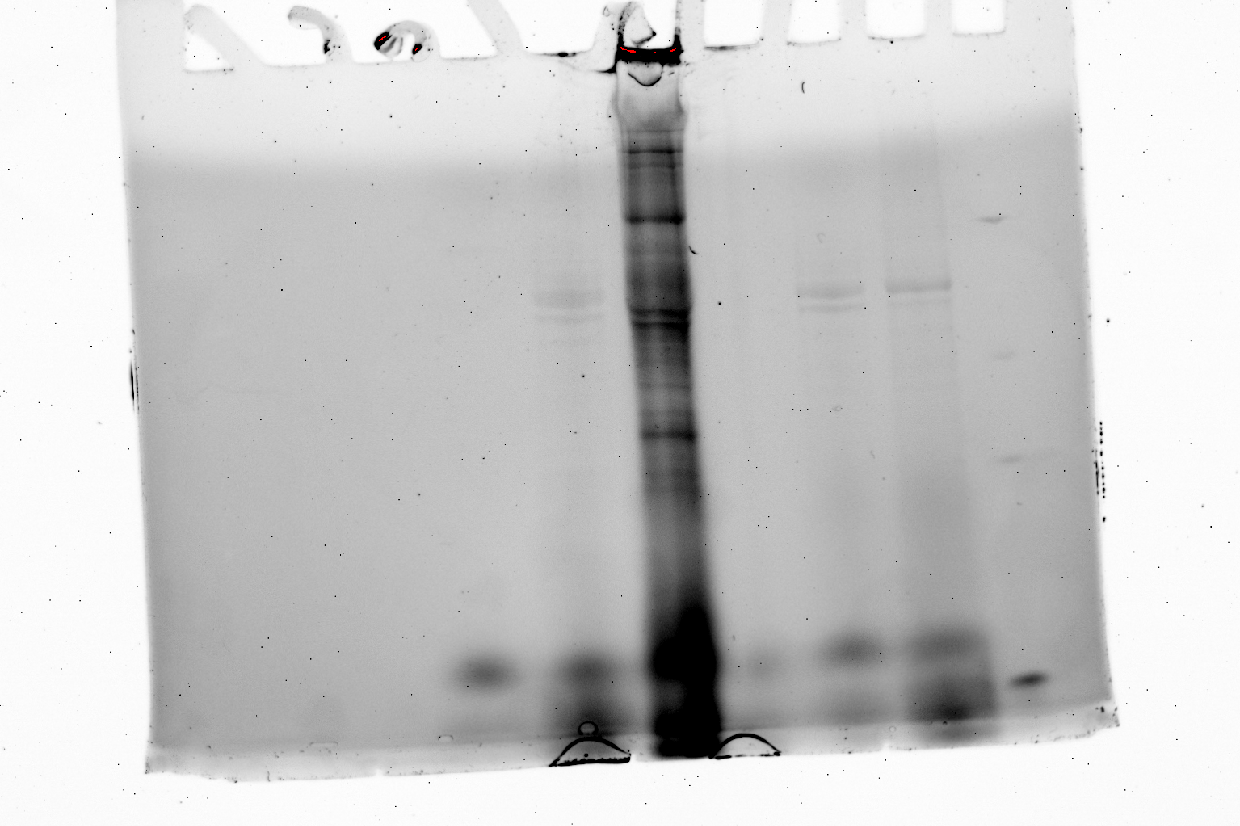

Supplement: Supplementary file 4 — Source data [file 41467_2025_64295_MOESM4_ESM.zip › Excel files/Source Data Figure 3j.tif]

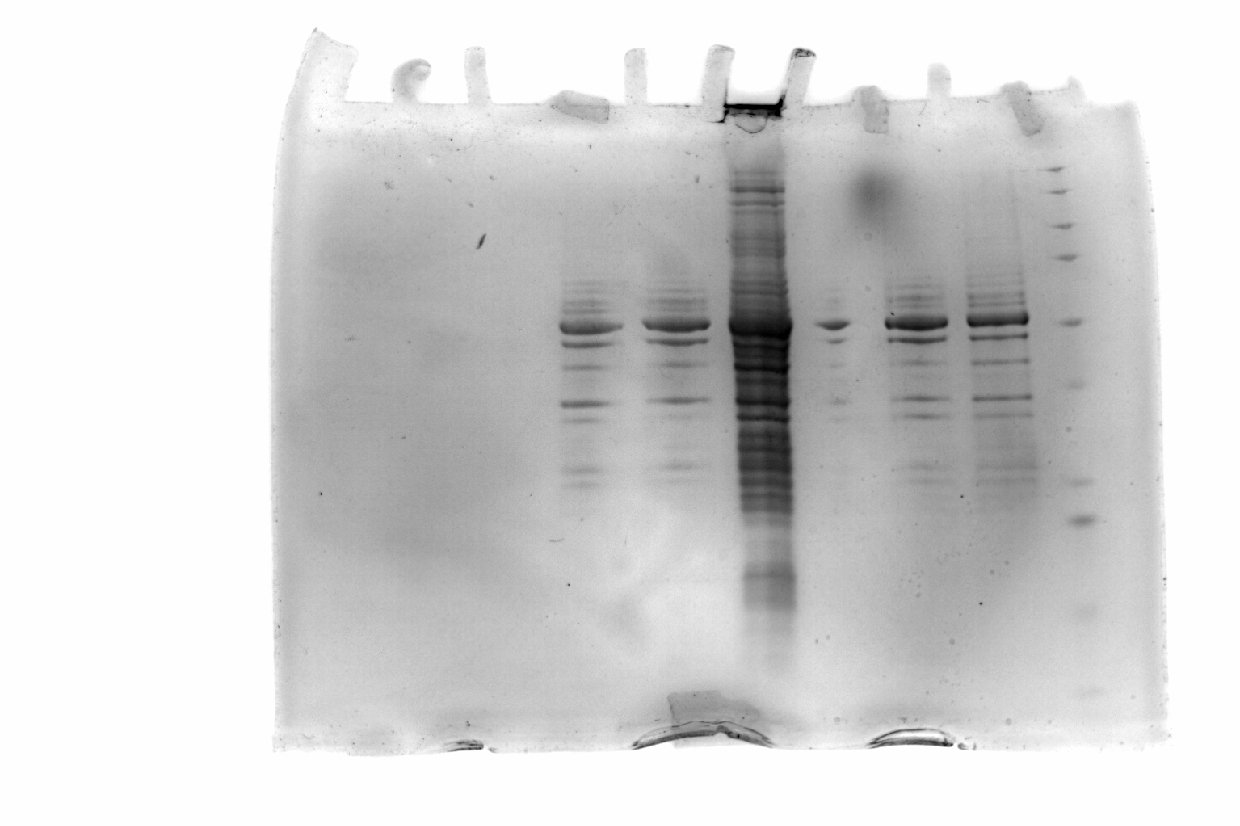

Supplement: Supplementary file 4 — Source data [file 41467_2025_64295_MOESM4_ESM.zip › Excel files/Source Data Figure 41.tif]

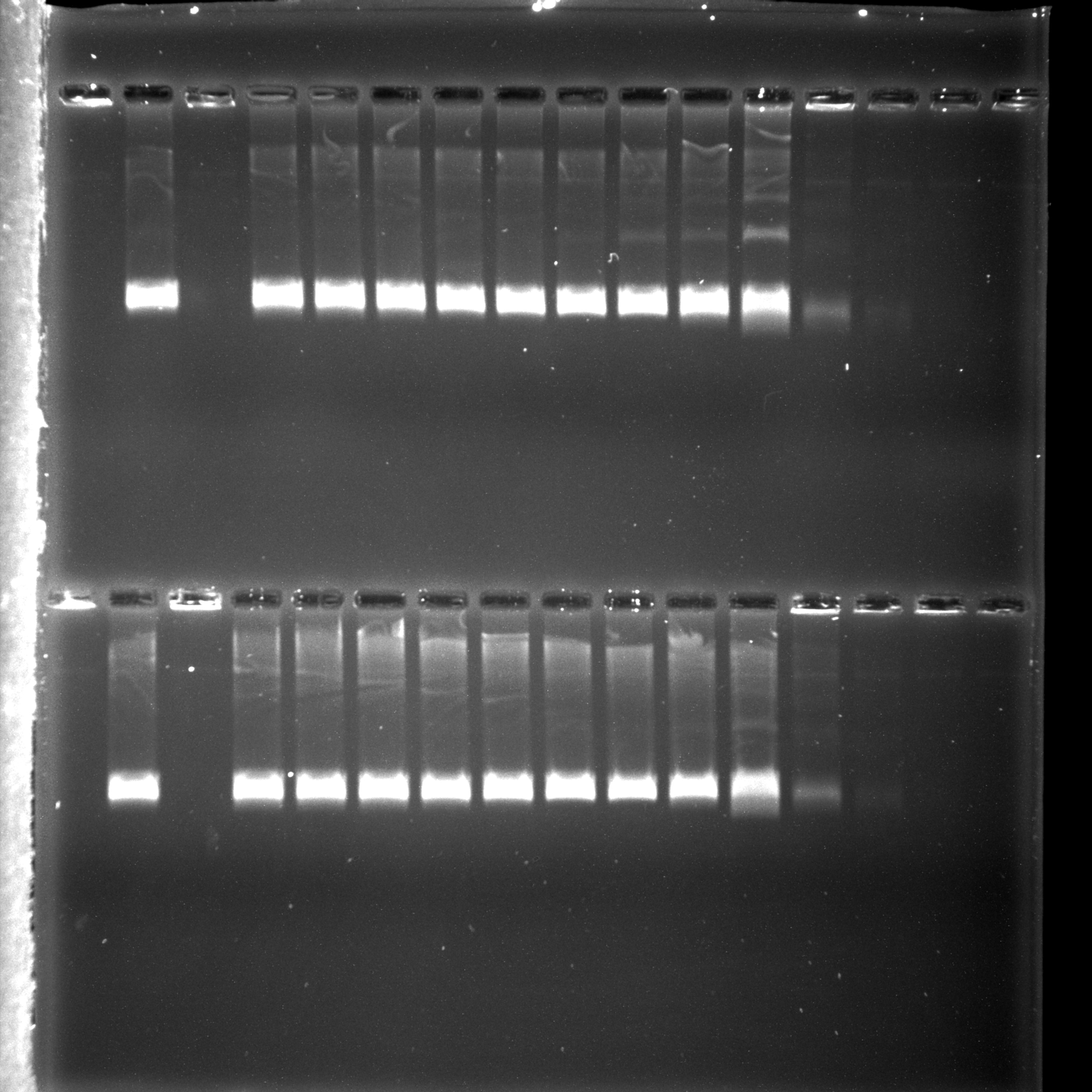

Supplement: Supplementary file 4 — Source data [file 41467_2025_64295_MOESM4_ESM.zip › Excel files/Source Data Figure S100 Gel.jpg]

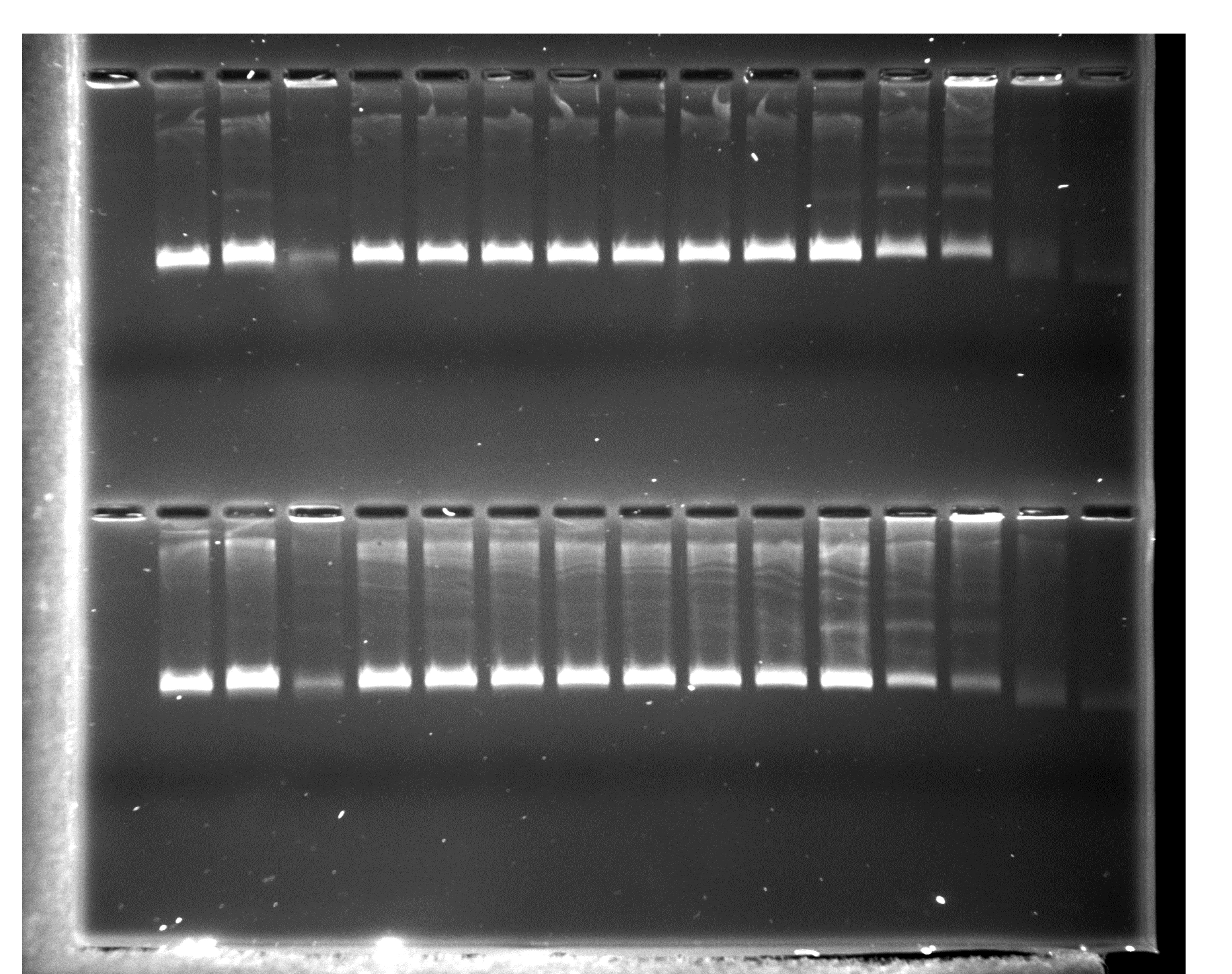

Supplement: Supplementary file 4 — Source data [file 41467_2025_64295_MOESM4_ESM.zip › Excel files/Source Data Figure S101 Gel.jpg]

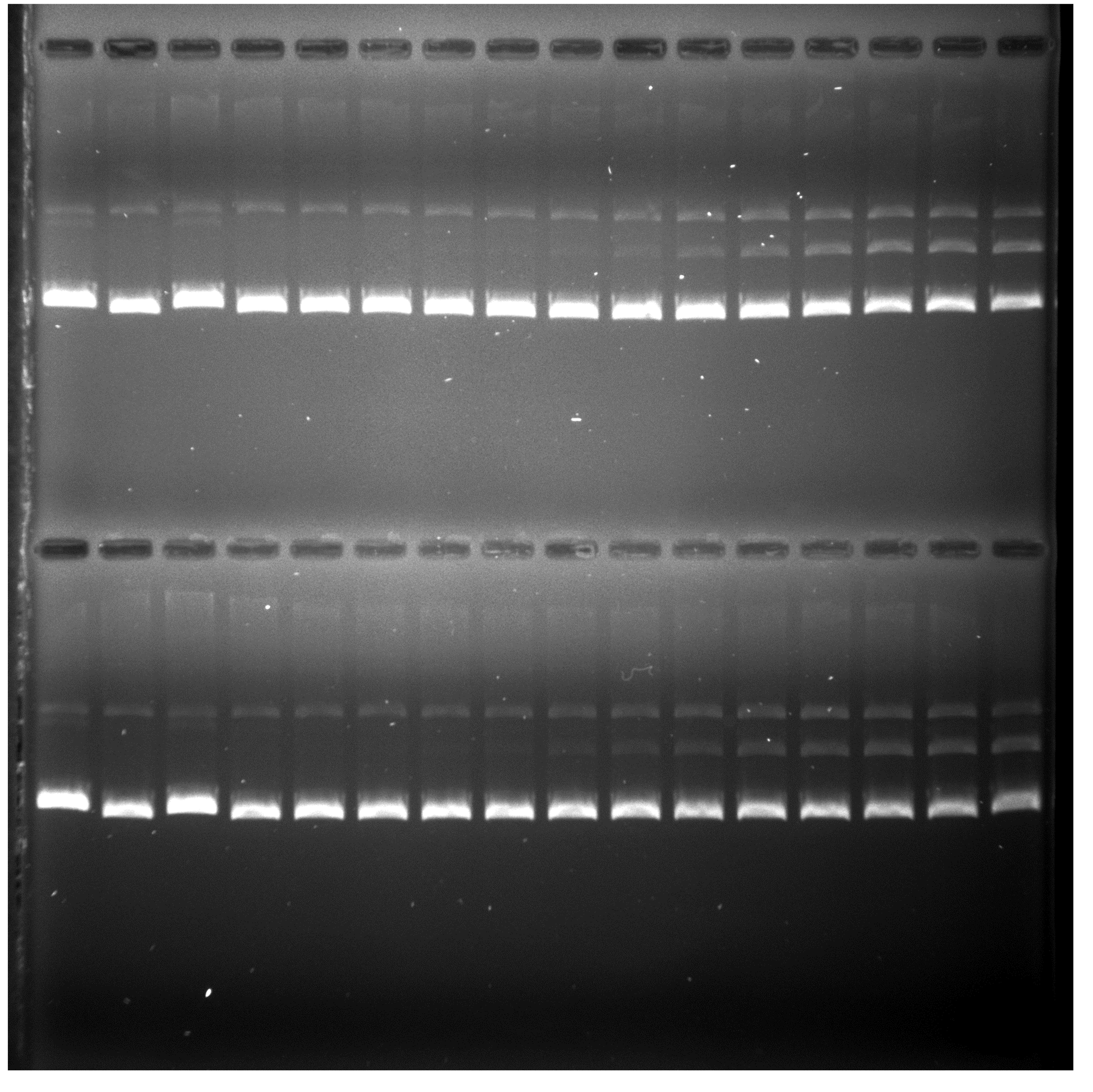

Supplement: Supplementary file 4 — Source data [file 41467_2025_64295_MOESM4_ESM.zip › Excel files/Source Data Figure S102 Gel.jpg]

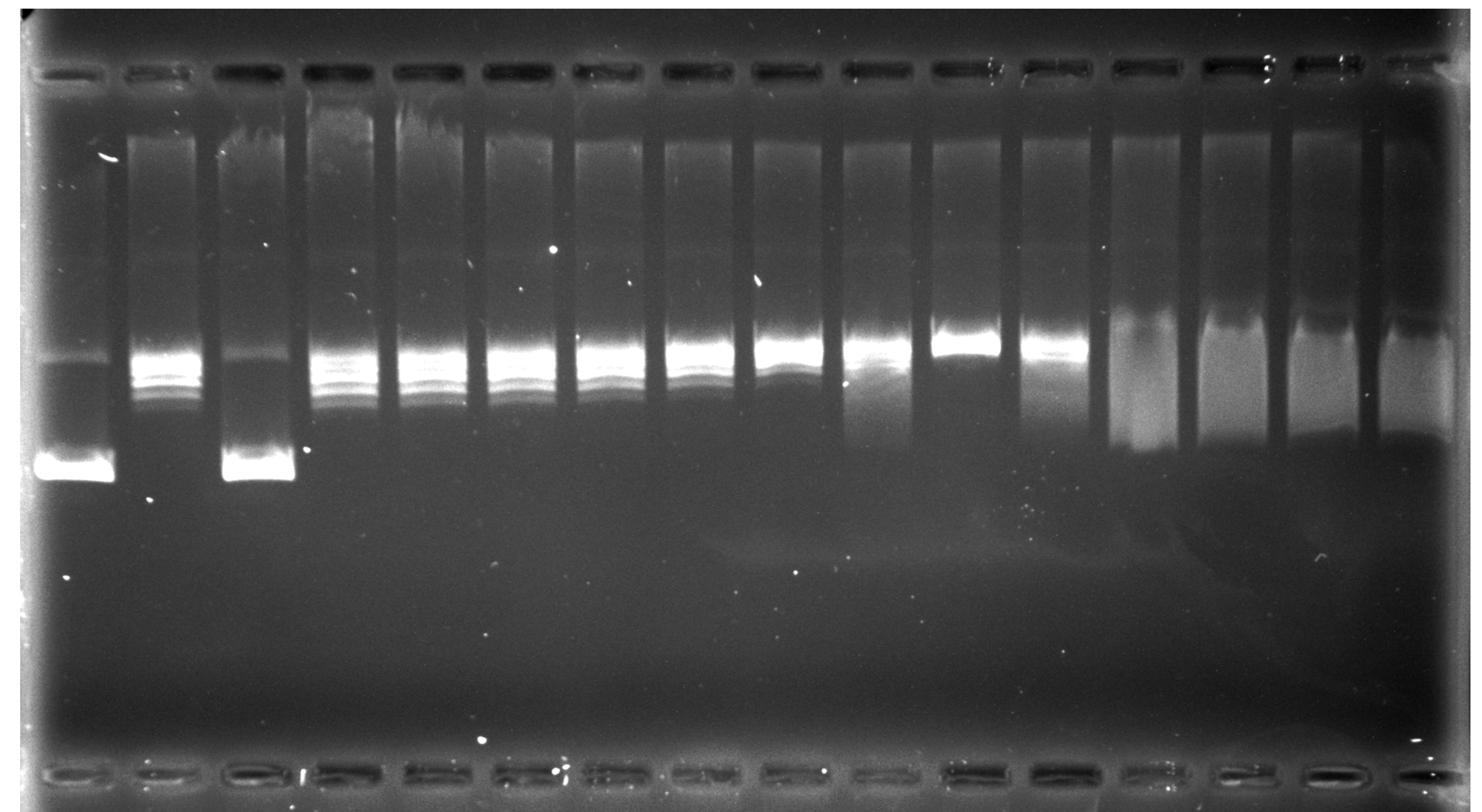

Supplement: Supplementary file 4 — Source data [file 41467_2025_64295_MOESM4_ESM.zip › Excel files/Source Data Figure S103 Bottom Gel.jpg]

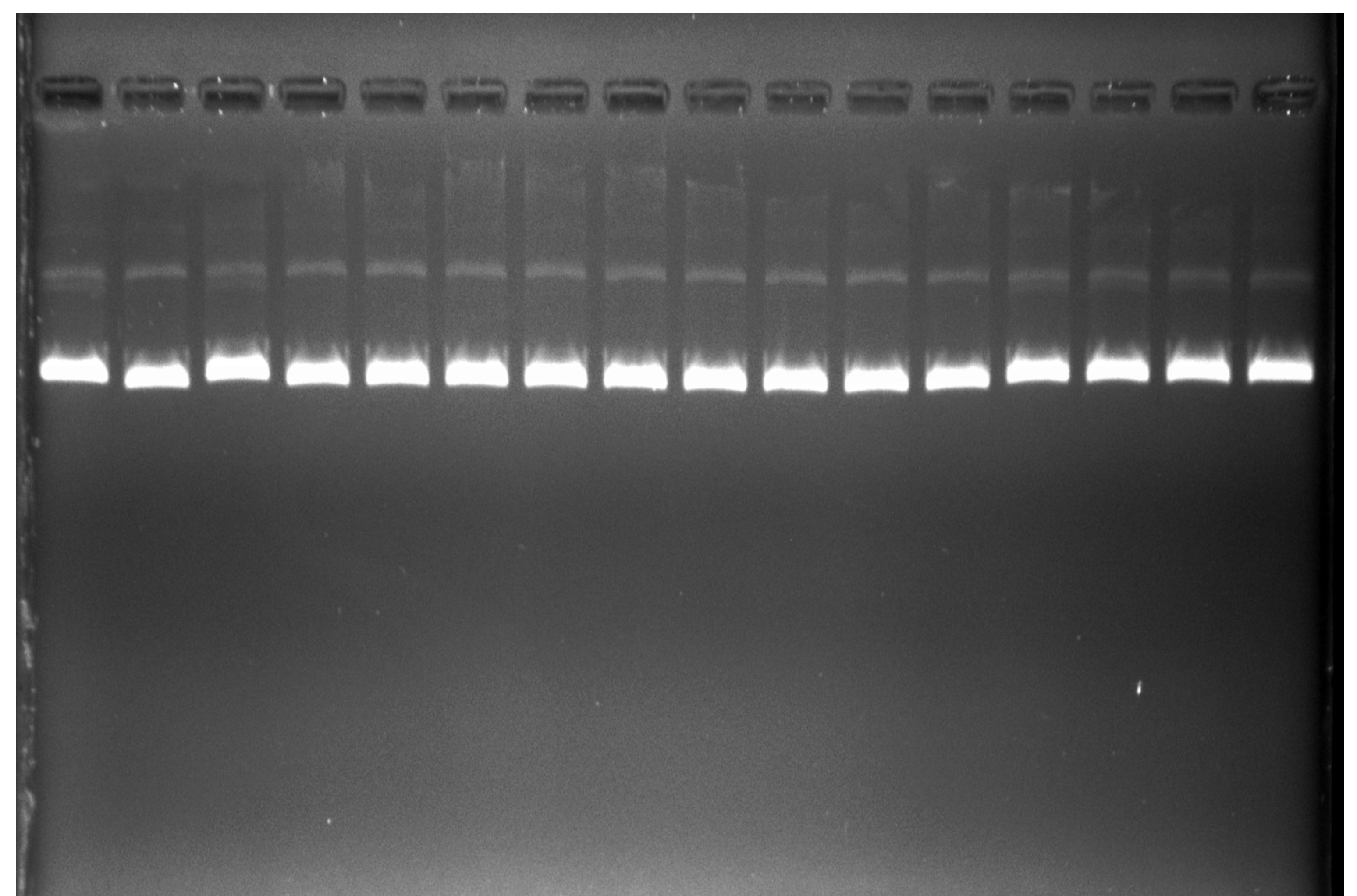

Supplement: Supplementary file 4 — Source data [file 41467_2025_64295_MOESM4_ESM.zip › Excel files/Source Data Figure S103 Top Gel.jpg]

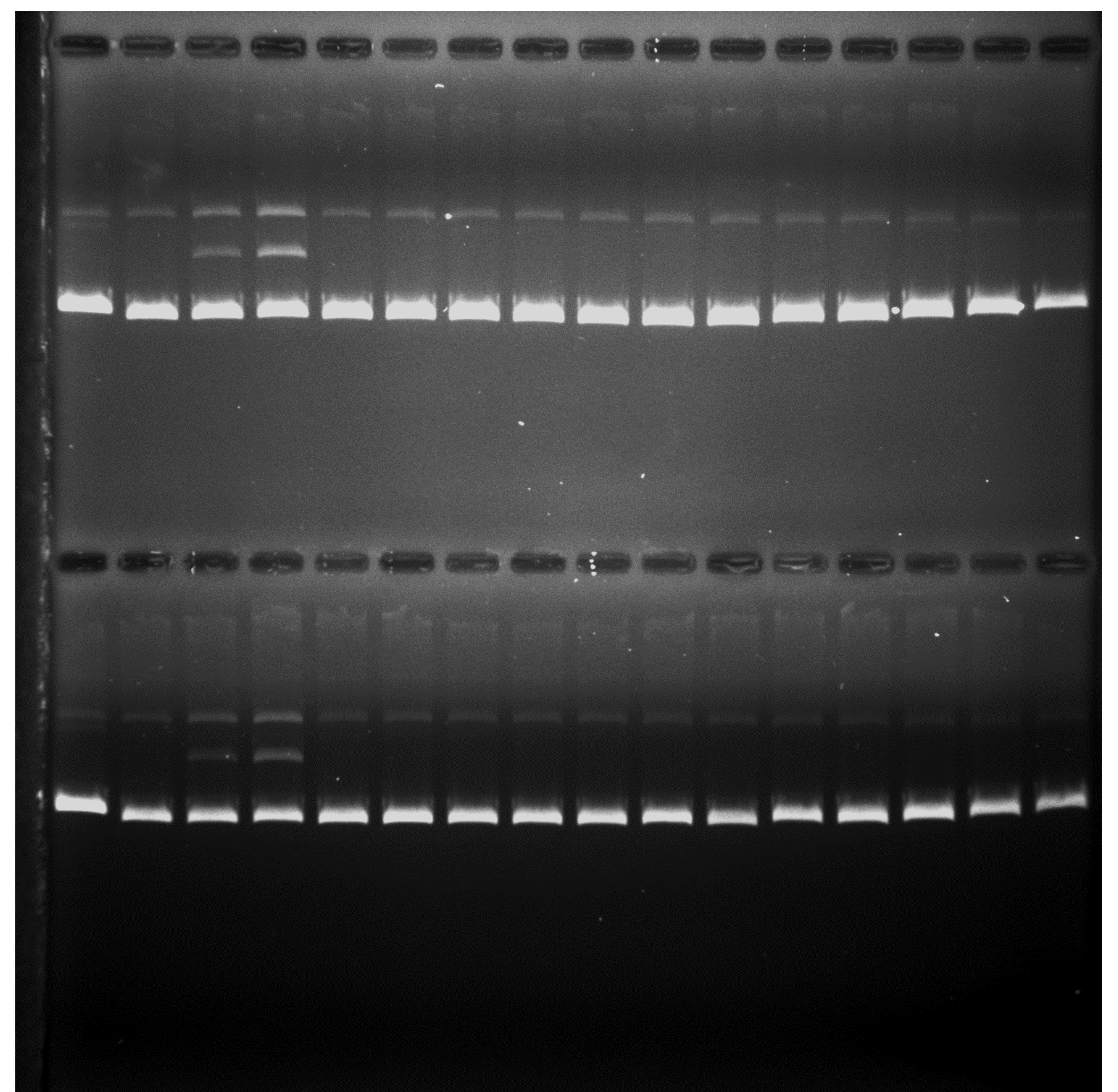

Supplement: Supplementary file 4 — Source data [file 41467_2025_64295_MOESM4_ESM.zip › Excel files/Source Data Figure S104 Gel.jpg]

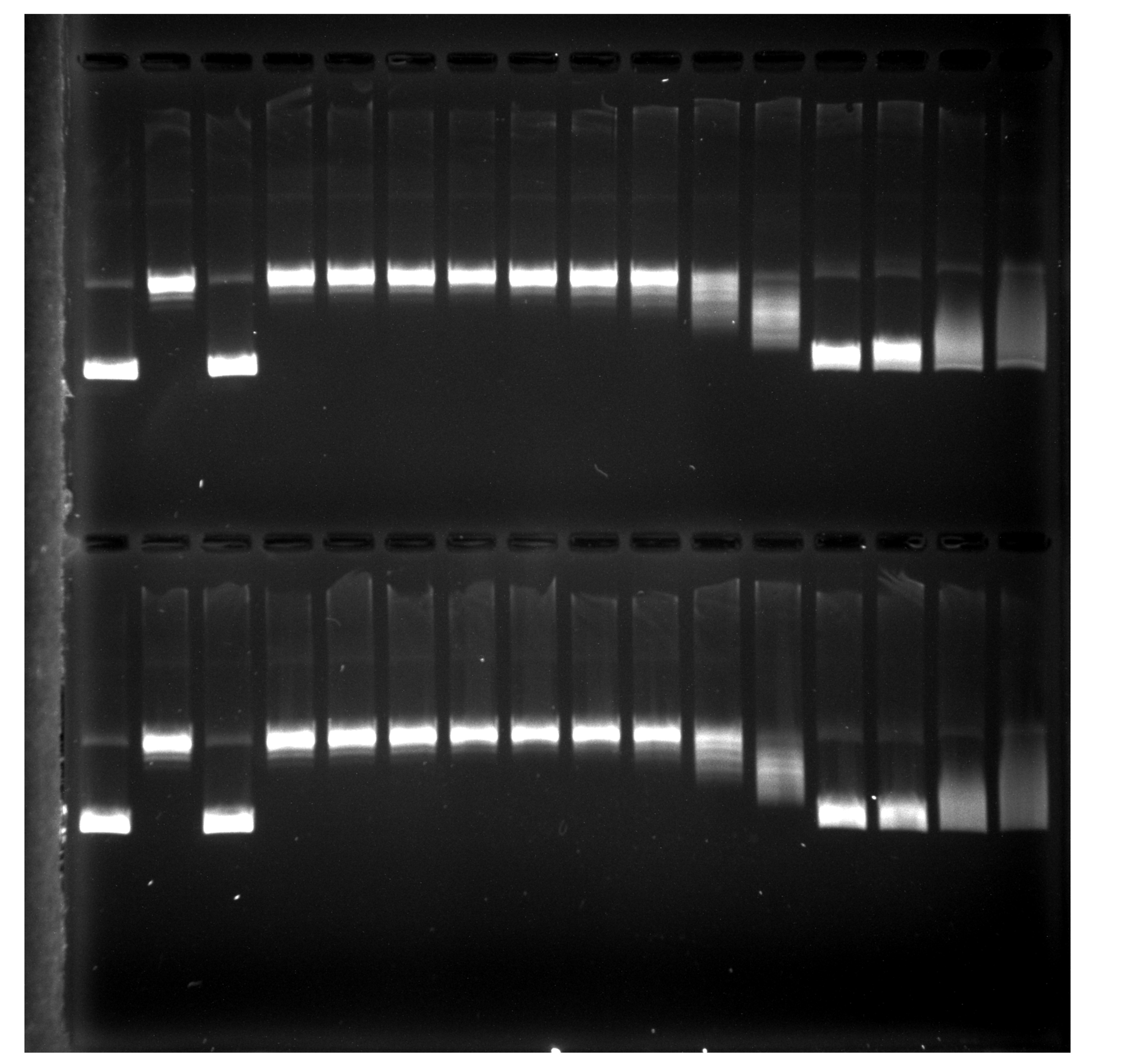

Supplement: Supplementary file 4 — Source data [file 41467_2025_64295_MOESM4_ESM.zip › Excel files/Source Data Figure S105 Gel.jpg]

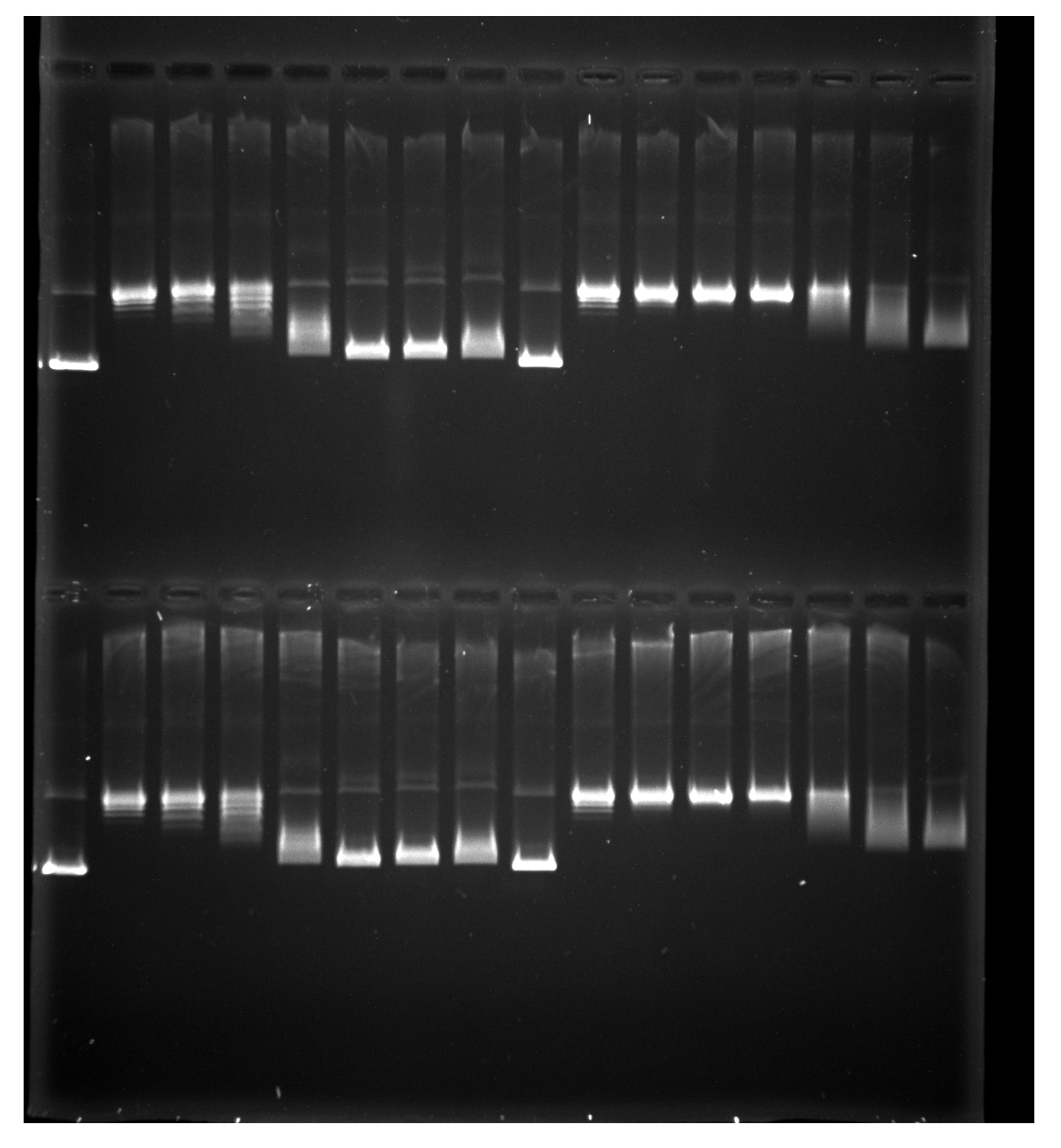

Supplement: Supplementary file 4 — Source data [file 41467_2025_64295_MOESM4_ESM.zip › Excel files/Source Data Figure S106 Gel.jpg]

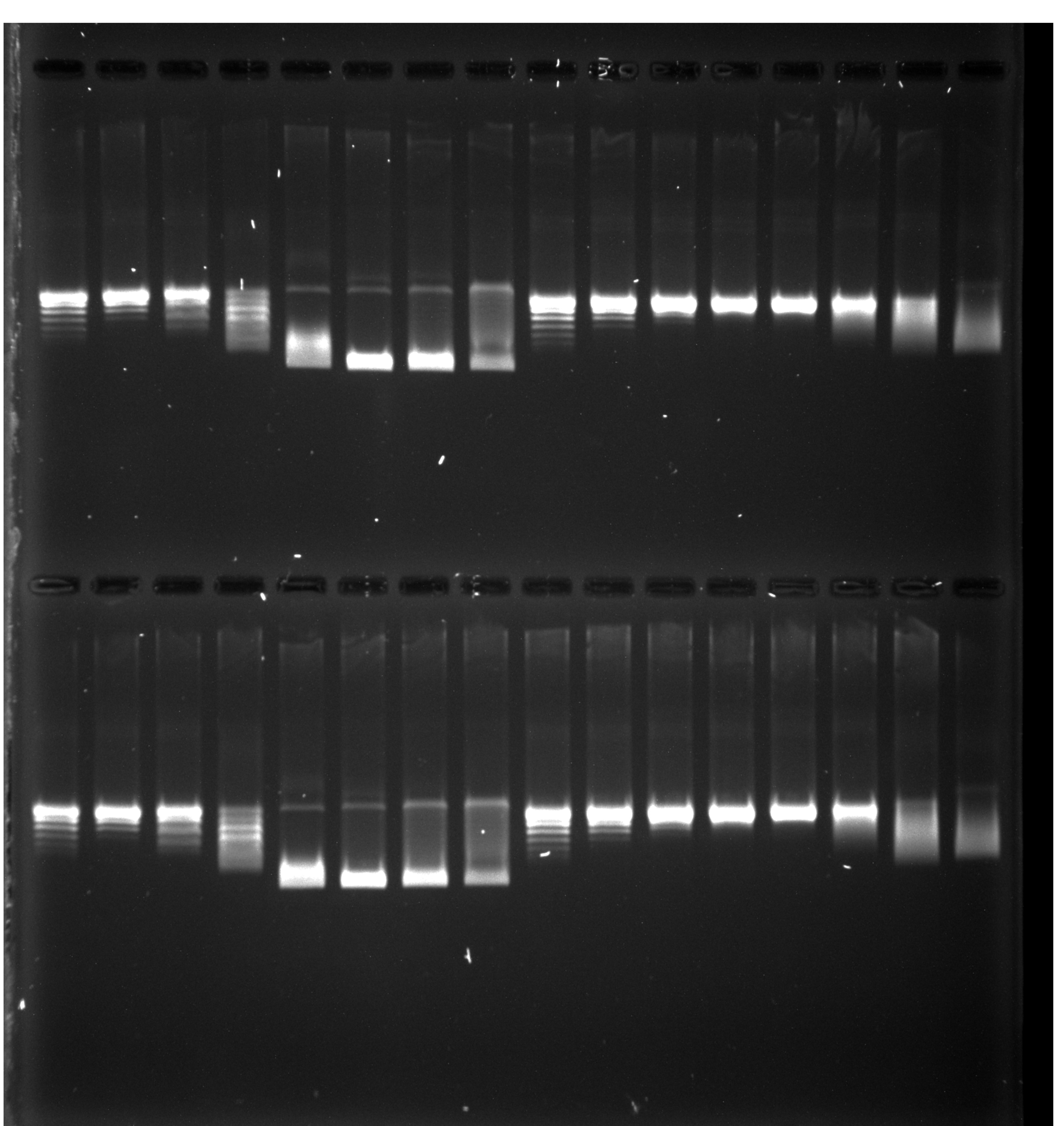

Supplement: Supplementary file 4 — Source data [file 41467_2025_64295_MOESM4_ESM.zip › Excel files/Source Data Figure S107 Gel.jpg]
